# Novel Eukaryotic Double Histone Fold Motifs Sharing High Sequence Identity with Histones H3 and H4 in Single-Domain Proteins and in Proteins Featuring Remarkable Histone Fold Multiplets

**DOI:** 10.1021/acsomega.6c01090

**Published:** 2026-08-10

**Authors:** Anna Ranaudo, Toshiko Miyake, Haidi Shehi, Ugo Cosentino, Marco Orlando, Marco Nardini, Claudio Greco

**Affiliations:** † Department of Earth and Environmental Sciences, 9305 University of Milano-Bicocca, Piazza della Scienza 1, 20126 Milan, Italy; ‡ Department of Pharmaceutical and Pharmacological Sciences, University of Padova, via Marzolo, 5, 35131 Padova, Italy; § Department of Biosciences, University of Milano, via Celoria 26, 20133 Milan, Italy; ∥ Laboratory for Environmental and Life Sciences, 119110 University of Nova Gorica, Rožna Dolina, SI-5000 Nova Gorica, Slovenia

## Abstract

Histone proteins play a central role in chromatin organization. In eukaryotes, the fundamental units of DNA packaging—the nucleosomal cores—are assembled from histone dimers. The double histone fold (DHF) refers to a protein architecture in which two adjacent regions, each containing a histone fold, associate to form a histone pseudodimer. In the present study, by targeted sequence searches in protein databases and subsequent structural and phylogenetic investigations, we identified a large number of DHF proteins featuring a high or very high degree of identity with the amino acid sequences of both histones H3 and H4, which constitute a new class of eukaryotic DHF proteins. Strikingly—somehow in analogy with recently identified proteins encoded in some giant viruses—we found, as well, triplets of various kinds (i.e., proteins showing regions of homology with histones H3, histone H4, and an additional histone fold). We were also able to evidence the existence of unprecedented quadruplets encompassing two distinct DHF domains, as well as multiplets that include not only region of homology to nucleosome core histones, but also to the linker histone H1. Focus was put on the evolutionary scenarios for the origin of the newly identified proteins, as well as on the conservation of residues relevant for dimerization and DNA binding. Implications of our findings in fundamental areas of biochemistry are illustrated, and perspectives for future research directions are discussed.

## Graphical abstract

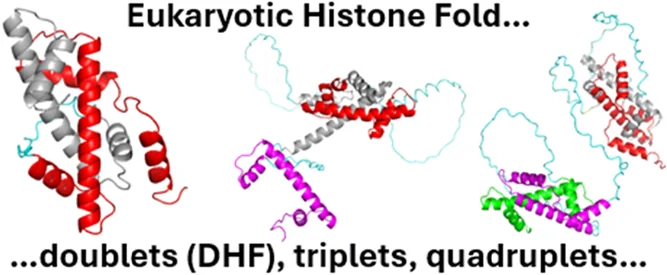

## Introduction

Histones are fundamental components of chromatin and form the protein core around which DNA is wrapped in eukaryotic nuclei. The canonical nucleosome consists of an octamer of core histones—two copies each of H2A, H2B, H3, and H4—organized as heterodimers, which assemble into a stable particle around approximately 146 bp of DNA.^1^ Structurally, this organization is based on the histone fold, a conserved motif composed of three α-helices connected by two loops, which mediates dimerization through extensive hydrophobic interactions (see Figure 1).^1^

**Figure 1:**
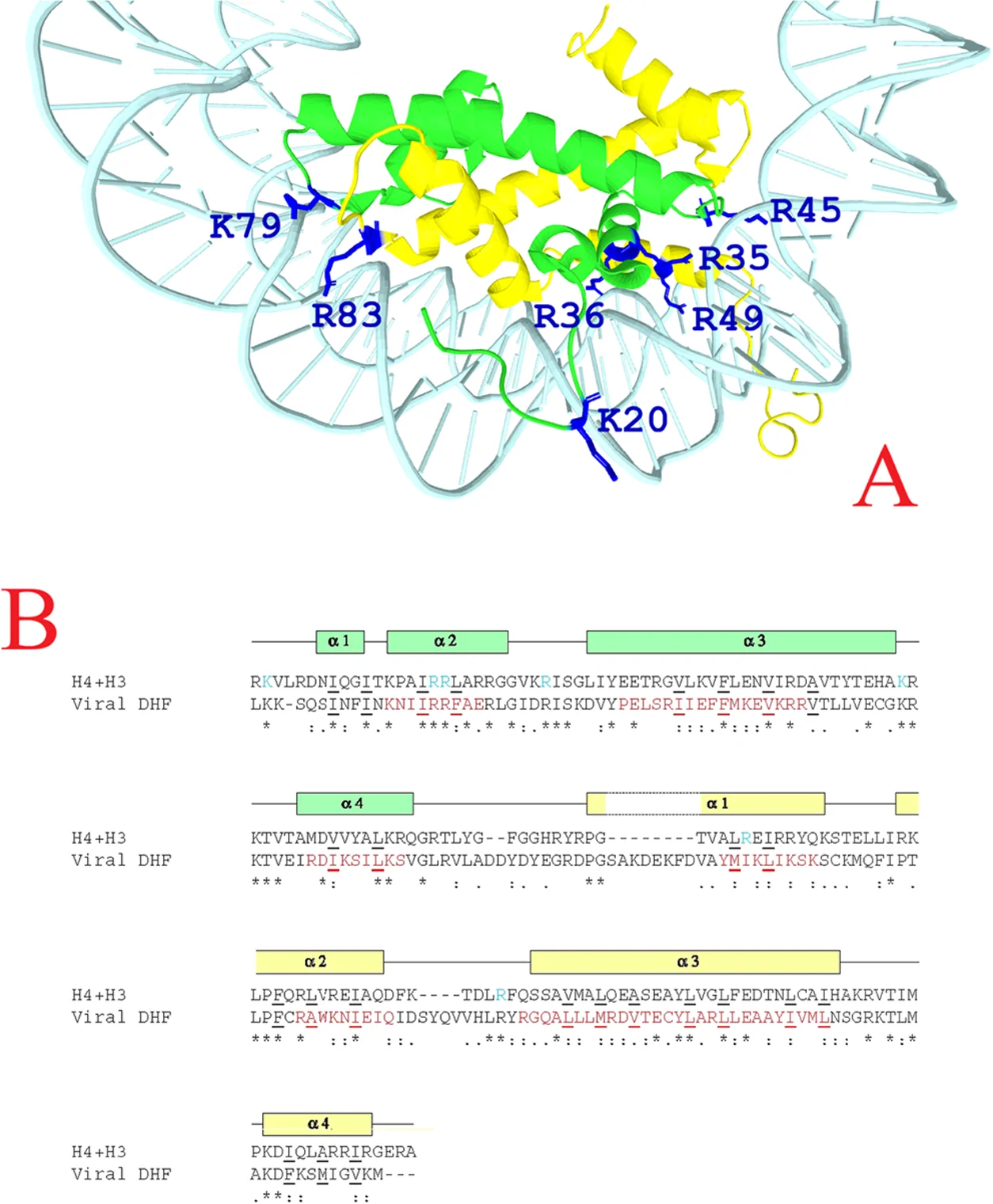
(a) Relative positioning of two dimerizing histones, i.e., histone H3 (in yellow), histone H4 (in green), and DNA in the nucleosome core particle; the positively charged amino acid residues in direct contact with DNA are highlighted in blue and labeled. (b) Alignment between the primary amino acid structure of human histones H4 and H3 and the amino acid sequence of the double histone fold from *Heliothis zea* nudivirus 1 (previously known as Hz-1 virus).^6^ Positions of α-helices in histone H4 and histone H3 are evidenced by green and yellow rectangles, respectively. Red letters indicate regions predicted to assume α-helical structure;^6^ blue letters indicate residues mediating contacts between histone dimer and DNA. Underlined residues belong to the pattern of residues whose hydrophobicity is conserved. The lower panel of the picture was reprinted from ref 6. Copyright 2005, Greco et al.

Although historically associated with chromatin in eukaryotes, the histone fold is actually much more widespread than was previously thought. Viral histones,^2,3^ archaeal histones,^2,4^ bacterial histone-like proteins,^2,5^ and numerous histone fold-containing domains in nonchromatin proteins illustrate how this structural motif has been repeatedly reused throughout evolution. Among these variants, the double histone fold (DHF) is a particularly notable example. In this configuration, two histone folds are encoded in succession within a single polypeptide chain and fold intramolecularly to form a structure that closely resembles that of a histone heterodimer. As an example, Figure 1 shows the amino acid sequence of a viral double histone fold protein that exhibits regions of homology to nucleosomal histones H3 and H4, forming a DHF predicted to function as a DNA binding protein.^6^

The first experimentally characterized examples of a double histone fold came from an archaeal histone expressed by *Methanopyrus kandleri* ^7^ and from the N-terminal regulatory domain of the human Son of Sevenless 1 (hSos1) protein, a Ras guanine nucleotide exchange factor.^8^ These findings challenged the assumption that histone folds function exclusively as obligate intermolecular dimers and suggested that histone-like architectures could be embedded within multidomain proteins with noncanonical roles.

Over the past two decades, experimental and computational investigations have identified additional DHF-containing proteins in eukaryotes, viruses, and bacteria, progressively reshaping our understanding of the spread and functional versatility of this fold.^2,9–13^ Specifically, in the case of viruses, early studies indicated the presence of a DHF in the *H. zea* nudivirus 1 (Figure 1) and recent large-scale analyses indicated that DHF is encoded in species belonging to the Nucleo-Cytoplasmic Large DNA Viruses (NCLDV) group.^6,11^ Notably, the genetic material of the latter encodes not only proteins including a doublet of histone folds (either H2A-like + H2B-like DHF, or H3-like + H4-like DHF), but also proteins featuring triplets and quadruplets.^13^ As far as the DHF of *H. zea* nudivirus 1, it is a single-domain protein that features a H3-like histone fold preceding an H4-like one (Figure 1).^6^ Beyond the case of viruses, some preliminary results on double histone folds featuring regions of homology with histones H3 and H4 were recently reported in the case of eukaryotic polypeptides annotated as TATA box binding protein-associated factor (TAF) histone-like fold domain containing proteins.^14^

Here, we investigate further the issue of DHF proteins containing regions of homology to histones H3 and H4 by means of targeted bioinformatic analyses supported by AlphaFold structural attribution of new DHF proteins and their phylogenetic analysis.

## Results and Discussion

As illustrated below, by targeted sequence searches in protein databases and subsequent structural investigations based on AlphaFold,^15^ we identified a large number of different DHF proteins featuring a high degree of identity with the amino acid sequences of both histones H3 and H4. Notably, we found that such proteins belong to different, newly identified classes of eukaryotic DHF proteins. In fact, apart from the case of proteins containing a doublet of histone folds featuring the region of homology to histone H3 positioned before the region of homology with histone H4 along the protein sequence—in analogy with the above-mentioned, previously identified TAF-annotated proteins—we found doublets featuring such two histone folds in swapped relative positions; we also found triplets of various kinds (i.e., proteins showing regions of homology with histones H3, histone H4, and an additional histone fold), quadruplets, as well as multiplets. Moreover, we found molecular evidence for these combinations not to be the result of progressive acquisitions, but rather a mosaic of domain combinations that evolved many times and resulted in many different domain architectures. Implications of such findings are discussed at the end of the present contribution.

Our investigation was carried out by means of searches that used combinations of known dimerizing human histone sequences as queries (*vide infra*); searches were carried out by using protein BLAST,^16^ as described in the Supporting Information. For our initial sequence search, we employed a ‘chimeric’ query by joining together the full sequences of human histone H3 and H4, in such relative order (H3H4 query sequence reported in the Supporting Information).

The first outcome of such a search corresponds to evidencing the existence of a group of proteins similar to each other in terms of length (ca. 240 aa) and expressed in various eukaryotic species, which feature a tandem of sequences homologous to histones H3 and H4 that shares with the query an overall high sequence identity, ≥88% (see Table 1). A structural model of one of the proteins of such group—the one expressed by *Heterocephalus glaber*—obtained with AlphaFold, is shown in Figure 2 (protein with code EHB13139.1 within NCBI RefSeq; notice that, in the following, NCBI RefSeq codes will be used to identify all the proteins here discussed). The AlphaFold model in Figure 2 highlights the packing of the two histone folds against each other to form a pseudodimer, which is predicted at a high confidence level. Therefore, AlphaFold outcomes show that this close homologue of nucleosome histones H3 and H4 features a double histone fold structure, and highlight that it is composed of a single domain, in full analogy to the above-mentioned cases of viral DHF proteins in NCLDV that are known to be involved in DNA packaging.^11^ A prerequisite for DHF formation is that the linker region between the two histone folds be long enough to allow their intramolecular packing; notably, also the other proteins reported in Table 1, which have similar or longer amino acid sequences with respect to the *H. glaber* DHF, appear to have stretches of linker sequences that permit pseudodimerization (see sequence alignments in the Supporting Information). Accordingly, residues involved in H3–H4 dimerization (Table S1) and DNA binding (Table S2) are highly conserved across all HF doublets reported in Table 1.

**Figure 2:**
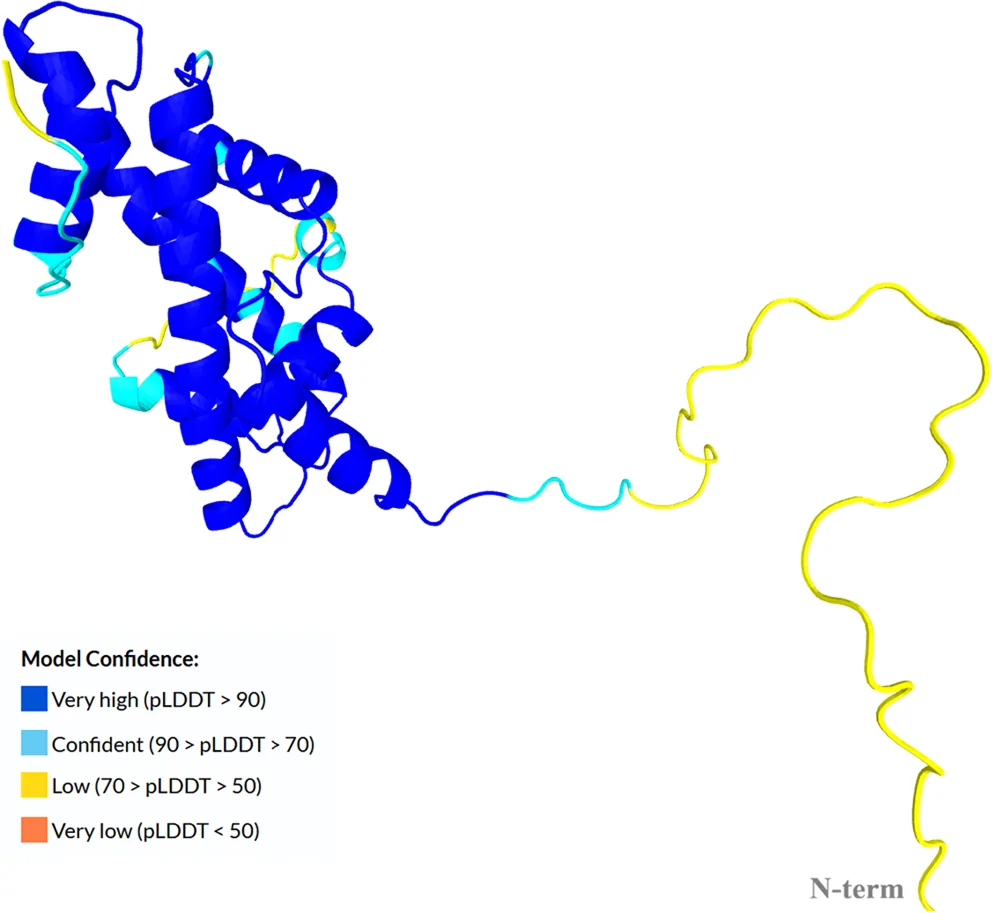
Double histone fold from *H. glaber* (NCBI RefSeq protein code EHB13139.1). The structure is colored according to the AlphaFold predicted local distance difference test (pLDDT; see the legend within the picture), a per-residue measure of local confidence of structural prediction.

**Table 1:** Proteins Featuring Doublets of Histone Folds (HF), Retrieved Using a Chimeric Sequence Obtained by Joining Human Histones H3 (136 Amino Acids) and H4 (103 Amino Acids) Sequences (H3H4 Query)^c^

protein sequence ID^a^ (amino acid length)	organism	HF domain organization	overall query % identity	HF domain % identity	% identity of dimerization residues	% identity of DNA binding residues
XP_057322000.1 (218)	*Microplitis mediator*	H3–H4	88	81–99	82–100	90–100
XP_015988254.2 (218)	*Rousettus aegyptiacus*	H3–H4	88	79–100	82–100	90–100
XP_032717653.1 (219)	*Lontra canadensis*	H3–H4	90	82–100	82–100	90–100
EHB13139.1^b^ (222)	*Heterocephalus glaber*	H3–H4	91	94–87	100–100	100–100
KAK2892181.1 (228)	*Channa argus*	H3–H4	91	88–95	100–100	100–100
KAK2913306.1 (233)	*Cirrhinus molitorella*	H3–H4	95	90–100	100–100	100–100
XDA87243.1 (242)	*Ovis aries*	H3–H4	98	97–100	100–100	100–100
XP_014913934.1 (251)	*Poecilia latipinna*	H3–H4	88	78–100	82–100	90–100
KAH9426658.1 (257)	*Dermatophagoides pteronyssinus*	H3–H4	95	96–92	100–100	100–100
XP_037744148.1 (259)	*Chelonia mydas*	H3–H4	98	96–100	100–100	100–100
XP_061655078.1 (259)	*Phyllopteryx taeniolatus*	H3–H4	90	83–100	85–100	95–100
XP_044528527.1 (263)	*Gracilinanus agilis*	H3–H4	90	82–100	82–100	90–100
XP_062289628.1 (268)	*Scomber scombrus*	H3–H4	90	83–100	82–100	90–100
XP_054346924.1 (269)	*Pongo pygmaeus*	H3–H4	98	97–100	100–100	100–100

a NCBI RefSeq collection protein code

b For this protein, the AlphaFold model is reported in Figure 2.

c The domain organization along the sequence is shown, together with the percentage of amino acid sequence identity relative to the query and to the isolated H3 and H4 domains. The percentages of sequence identity for residues involved in HF dimerization (see Table S1) and DNA binding (see Table S2) are reported in the last two columns.

Another interesting outcome of our BLAST search regards the occurrence of protein sequences featuring multiplets of histone folds in eukaryotes. The first case of this kind regards triplets, a selection of which is reported in Table S3. In fact, Table S3 reports proteins with lengths up to 450 amino acids in which a couple of regions of homology to histone H3 and histone H4 are followed by a stretch of sequence that also shares high identity percentages with histones. AlphaFold 3D modeling of one such protein, namely, the one expressed in *Notolabrus celidotus* (RefSeq code XP_034546197.1), shows that its first two histone folds pack against each other to form a DHF domain, while the third histone fold along its sequence does not extensively interact with the latter domain (see Figure 3a). In the *N. celidotus* protein, the third histone fold along the protein sequence corresponds to a region of homology to histone H4; most of the triplets included in Table S3 have analogous features, even though occurrences of triplets with the last histone fold being an H3-like region are listed as well. Also, in the case of HF triplets, the residues involved in dimerization (Table S1) and DNA binding (Table S2) are conserved across all HF domains reported in Table S3. This observation is relevant because it suggests that the unpaired HF domain may also retain the potential to form a functional heterodimer with a complementary HF domain. It is also important to notice that by design, our BLAST sequence search could identify triplets including regions of homology to histones H3 and H4 only. However, proteins around 400 residues long that figured among the entries retrieved by our BLAST search were considered for AlphaFold analysis, which evidenced the existence of triplets featuring a DHF flanked by a single histone fold that was tightly related to neither histone H3 nor H4. This is the case, for example, of the protein VDP36995.1 expressed in the roundworm *Heligmosomoides polygyrus*, in which the single histone fold flanking the DHF domain corresponds to a region of homology to histone H2A (see Figure 3c,d for the AlphaFold model of such protein and for the analysis of its conserved domain by the NCBI CD-Search tool^17^).

**Figure 3:**
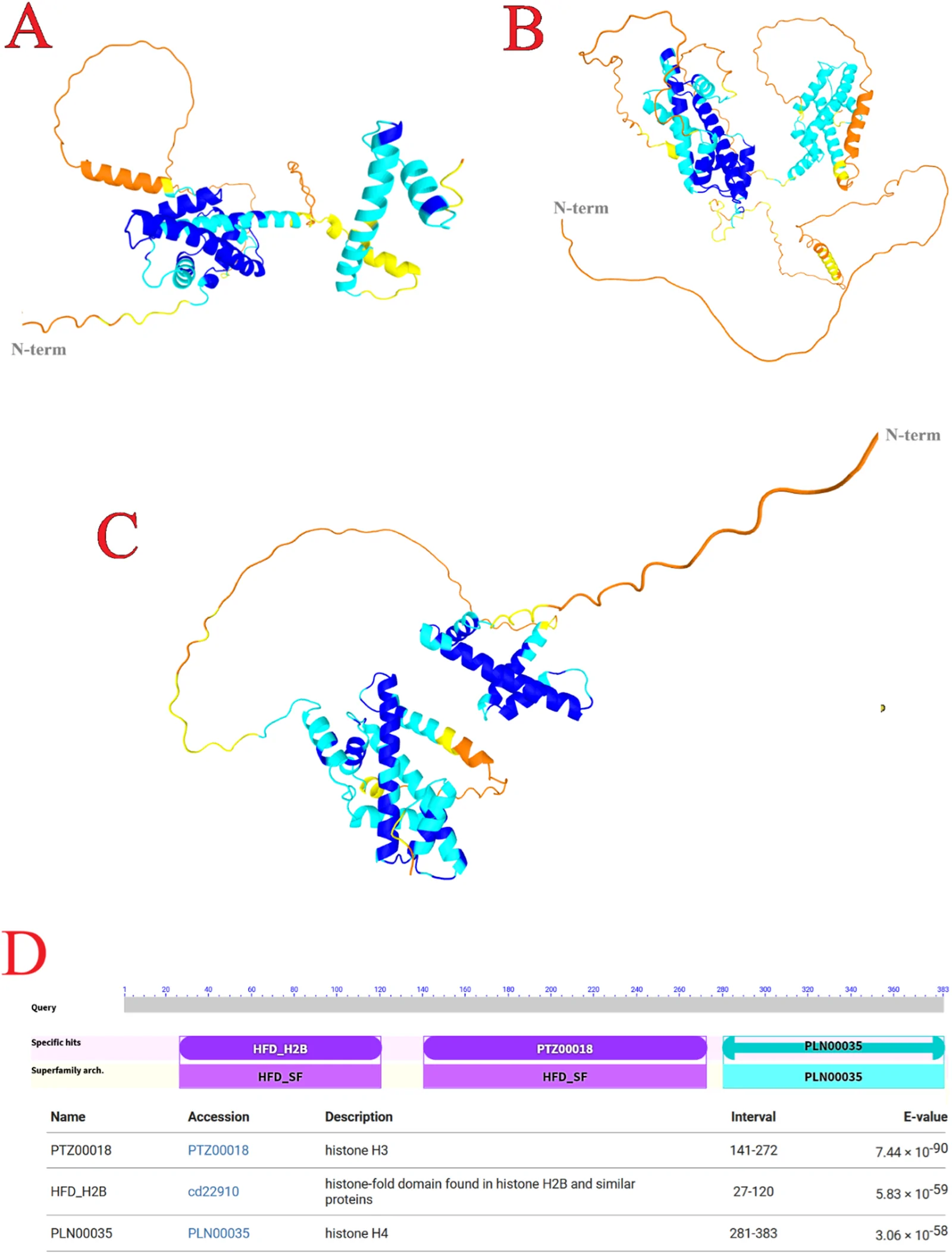
AlphaFold models for (a) protein XP_034546197.1 from *N. celidotus*, (b) protein EI555_001631 from *M. monoceros* that shows a couple of DHF domains, (c) protein VDP36995.1 from *H. polygyrus*; in all cases, the color code is in relation to model quality, and is the same as in Figure 2. (d) NCBI Conserved Domain Database (CDD) record for protein VDP36995.1,^20^ with specification of locations of conserved domain footprints; name, accession number, and description of the inferred structural motifs are reported, together with the sequence interval that corresponds to each motif and the *E*-value associated with each assignment.

Among multiplets, other forms are also represented; in particular, BLAST sequence searches followed by AlphaFold analysis highlighted the existence of a protein featuring a couple of DHFs, i.e., *Monodon monoceros* protein EI555_001631 (Figure 3b). Analysis of other long protein sequences among BLAST entries also evidenced the presence of proteins featuring multiplets of regions of homology to core histones, flanked by a region of homology with linker histone H1. A protein of such kind is *Ostrea edulis* protein XP_056017360.1, the conserved domains of which are shown in the Supporting Information (Figure S1).

Finally, we also carried out a BLAST sequence search by employing a ‘chimeric’ query sequence obtained by joining together the full sequences of histones H4 and H3, in such relative order (query H4H3). Also in this case, occurrences of proteins were found with a DHF flanked by a single histone fold (Table S4), with the latter being either H3- or H4-like, localized both before or after the H4H3 sequence query. HF triplets of Table S4 show a remarkable conservation of residues involved in dimerization and DNA binding, with the only exception of *Mirounga leonina* XP_034841793.1, where the second H4 domain shows a markedly lower conservation of residues involved in dimerization (60% sequence identity). This feature is due to a deletion in the region corresponding to the first two helices of the H4 domain, resulting only in a partial histone folding, as predicted by AlphaFold (not shown).

Another interesting case is represented by the *Anisakis simplex* protein VDK57329.1, whose AlphaFold structural model is shown in Figure 4. In practice, this class of proteins consists of triplets in which the DHF domain contains regions homologous to histones H4 and H3, arranged in a swapped configuration compared to that observed in the previously described protein from *N. celidotus* (Figure 3a); the additional unpaired HF domain is homologous to H2A. In addition to triplets, higher-order multiplets were also found, such as protein CAL5913199 found in *Lampetra fluviatilis* (see Supporting Information, Figure S2).

**Figure 4:**
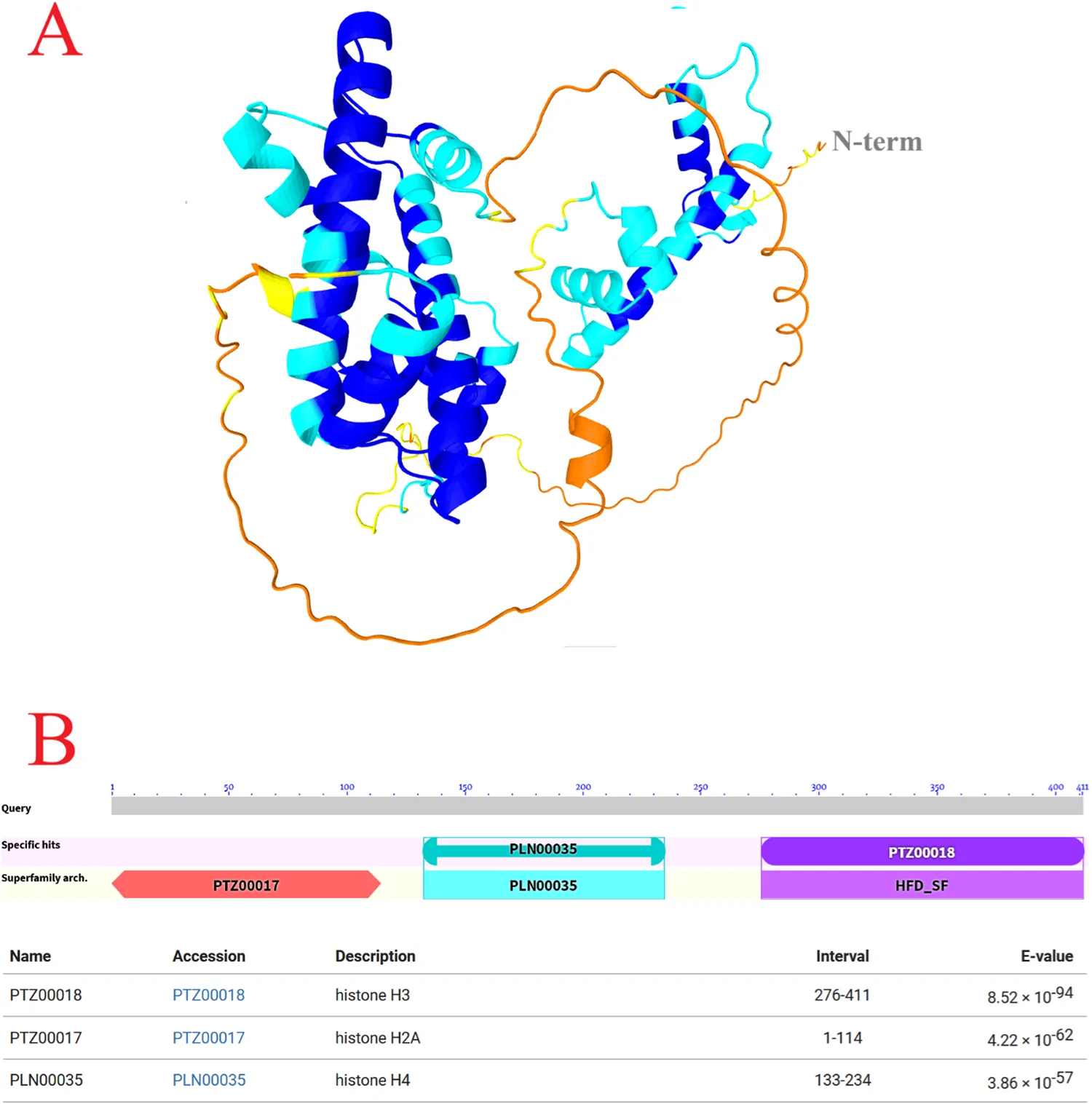
(a) AlphaFold model of the multiplet of histone folds from *Anisakis simplex* (NCBI RefSeq protein code VDK57329.1); the color code is related to model quality, and is the same as in Figure 2. (b) NCBI Conserved Domain Database (CDD) record for protein VDK57329.1,^20^ with specification of locations of conserved domain footprints; name, accession number, and description of the inferred structural motifs are reported, together with the sequence interval that corresponds to each motif and the *E*-value associated with each assignment.

To explore the evolutionary origins of these multiplets and determine whether they share a common ancestor or emerged independently, we performed a phylogenetic analysis of sequences featuring at least 75% pairwise identity to the *Pongo pygmaeus* H3–H4 doublet (Figure 5; see also SI, first section, for methodological details). The considered sequences, which include previously commented doublets and other cited multiplets, show a remarkable taxonomic diversity, encompassing species across major eukaryotic groups, both vertebrates (Mammalia, Actinopterygii, Aves, Amphibia, Reptilia, Petromyzontiformes) and invertebrates (including Arthropoda, Nematoda, Mollusca, and Xenacoelomorpha), and even Viridiplantae (*Chlorella vulgaris*). Notably, the phylogenetic tree shows that the H3/H4 doublets embedded within these sequences lack a clear taxonomic structure. Instead of clustering into distinct, lineage-specific clades, sequences with similar domain compositions are scattered across the tree; the same is true for sequences from the same or related species, which also have different domain architectures. Underscoring the broad distribution and structural plasticity of these motifs, some of the identified sequences reach remarkable sizes, such as a 1283-amino acid multiplet in *Lampetra fluviatilis*, an 891-amino acid protein in *Grammomys surdaster* (H4–H3–H1/H5–H3–H4), and an 851-amino acid protein in *Coregonus albula* (DUF–H3–H3–H3–H4). Despite the fact that the overall phylogenetic signal is low (bootstrap at nodes are not high), the mixed taxonomic topology and large variance in sequence length for closely related sequences support the hypothesis that such complex domain arrangements did not arise from a single ancestral evolutionary event but rather evolved independently many times.

**Figure 5:**
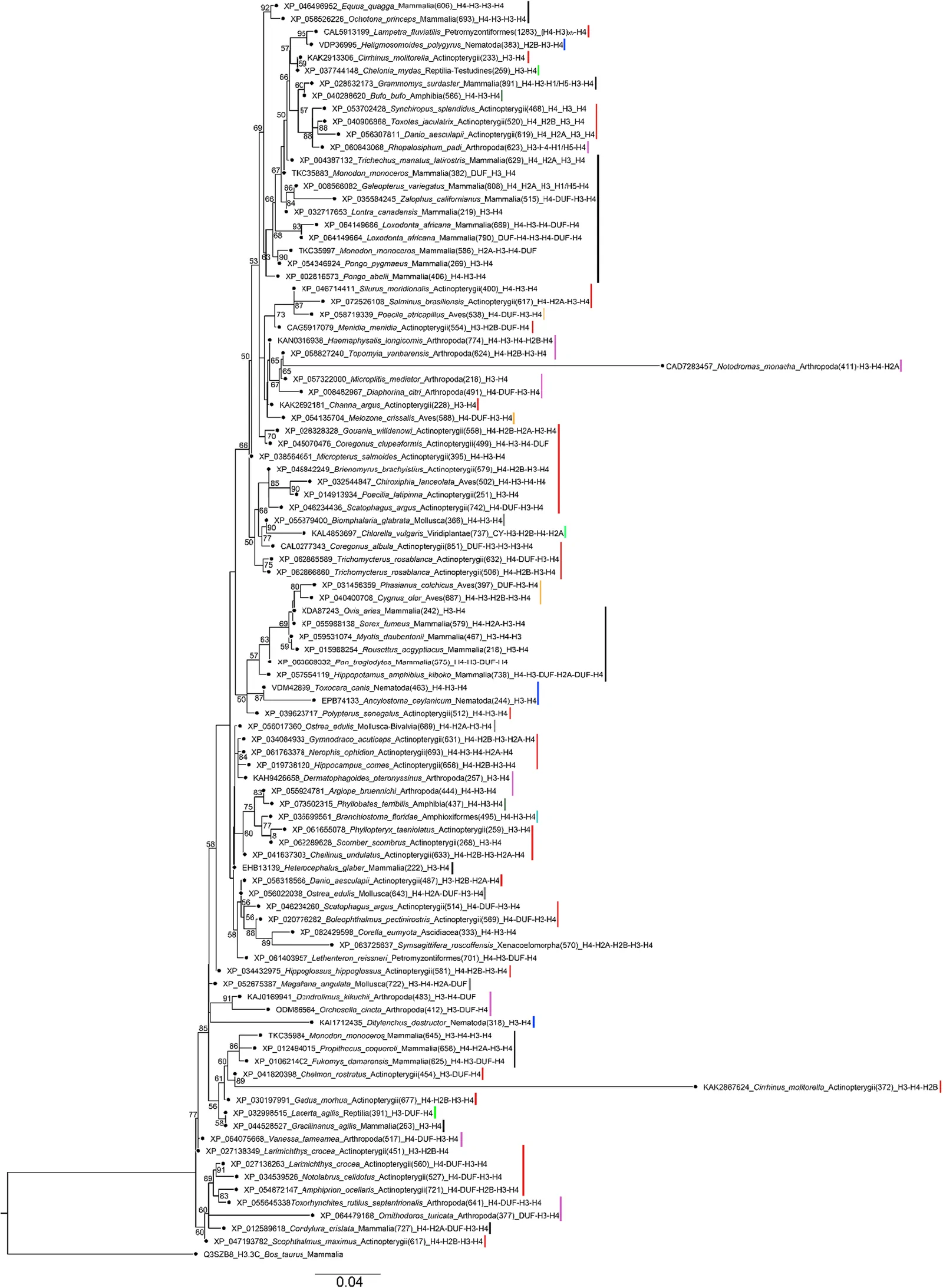
Phylogenetic tree of doublet to multiplet histone folds with at least 75% pairwise identity to *P. pygmaeus* H3–H4 doublet (NCBI protein ID: XP_054346924). A minimum 80% coverage was used to rule out any single-domain canonical nucleosome protein. Sequences were made nonredundant prior to analysis by clustering at 85% min global sequence identity. Outgroup used for rooting the tree is made by *Bos taurus* H3.3C, the closest characterized histone-like protein with <75% identity to *P. pygmaeus* H3–H4 doublet and with a different function with respect to canonical H3.^21^ The NCBI protein IDs, species’ name, and taxonomy are reported as header. Major taxonomy groups are also highlighted by vertical bars of different colors. The sequence length is reported in parentheses. The domain composition of each sequence is also annotated with 80% min identity and coverage to corresponding domains. UniProt IDs of references used for annotation: H3 = P68431; H4 = P62805; H2A = P62805; H2B = O60814; H1/H5 = Q02539; CY = P01040; DUF = one or more domain(s) of unknown function. The bar at the bottom is the scale of the fraction of expected number of substitutions per alignment position. Bootstrap values (0–100) are shown at each internal node, if >50.

To summarize, in this study, we show that single-domain proteins featuring the double histone fold and sharing very high sequence identity with histones H3 and H4 are rather common in eukaryotes. Moreover, and very interestingly, we found that a variety of multiplets also exist, with diverse and unique arrangements of histone folds along their respective protein sequences. Multiplets often display striking features, as detailed in the following. In many cases, at least one of the histone folds within these assemblies shares nearly complete sequence identity with that of canonical nucleosomal histones. Moreover, residues involved in dimerization and DNA binding are highly conserved, thus suggesting a direct role in chromatin structural organization. It is also noticeable that in some cases the histone multiplets identified in the present study encompass a region of homology to the linker histone H1; this fact further corroborates the hypothesis that multiplets can be involved in chromatin organization.

Although all functional hypotheses will need experimental validation in future studies, the above observations have an intriguing nature, as they provide possible novel degrees of freedom for the histone code. For example, the newly identified double histone fold proteins as well as the multiplets may undergo covalent modifications in the region of the linker between histone folds, which effectively replaces the N- and C-termini within conventional histones that are well-known targets of modifications functional to epigenetic modulation.^18^ More generally speaking, our results open broader questions related to whether the novel DHF and multiplets can be implicated in roles different from chromatin structural organization (e.g., gene transcription^14,19^ ).

Despite the significant functional signals at the domain level, the heterogeneous domain composition seems to have been highly explorative and originated from many different evolutionary events. Such a picture, to the best of our knowledge, is novel in the case of eukaryotes; it is curious that it somehow resembles what has been recently found in some giant viruses, which organize their DNA around protein structures that tightly resemble the nucleosome core, including histone doublets, triplets, or quartets in place of canonical single histones intermolecularly packed against each other. This suggests that the cause of this putatively functional architectural diversity may stem from duplications, deletions, and insertions promoted by transposable elements in eukaryotic genomes, with the contributions of ex- or present prophages.

Finally, our study corroborates the evidence that the presence of histone pseudodimers and multiplets is a shared feature of very diverse kinds of eukaryotic organisms;^14,19^ deepening insights into the function of the linkage between histone folds that give place to the double histone folds and multiplets is expected to provide fresh perspectives in key areas, from enhancing our fundamental knowledge of chromatin structure and dynamics, up to epigenetics and related areas.

## Supplementary Material



## Notes

Detailed methodologies and relevant protein sequence alignments (Tables S1–S4) (Figures S1 and S2) (PDF)
