# Supplementary material for: Novel Eukaryotic Double Histone Fold Motifs Sharing High Sequence Identity with Histones H3 and H4 in Single-Domain Proteins and in Proteins Featuring Remarkable Histone Fold Multiplets
Source: ACS Omega. 2026 Aug 10;11(33):49105–12. doi: 10.1021/acsomega.6c01090 (PMC13520046; doi:10.1021/acsomega.6c01090)
Supplement: Supplementary file 1 [file ao6c01090_si_001.pdf]

Supporting Information for

Novel eukaryotic double histone fold motifs sharing high sequence identity with histones H3 and H4 in single-domain proteins and in proteins featuring remarkable histone fold multiplets

Anna Ranaudo [1,$], Toshiko Miyake [2,$], Haidi Shehi[3,$] , Ugo Cosentino [1], Marco Orlando [4,], Marco Nardini[3,*], Claudio Greco [1,*]*

1 Department of Earth and Environmental Sciences, University of Milano-Bicocca, Piazza della Scienza 1, 20126, Milan (Italy)

2 Department of Pharmaceutical and Pharmacological Sciences, University of Padova, via Marzolo, 5, 35131 Padova (Italy)

3 Department of Biosciences, University of Milano, via Celoria 26, 20133 Milan (Italy)

4 Laboratory for Environmental and Life Sciences, University of Nova Gorica (Slovenia)

Detailed methodological procedures, with references

Protein sequence searches were performed using BLASTP against the UniProtKB reference proteomes and Swiss-Prot databases.[1,2] Searches were conducted with the following parameters: E-value threshold of 10, Auto BLOSUM62 substitution matrix, gapped alignments enabled, and no low-complexity filtering applied.

The BLASTP queries consisted of chimeric histone sequences generated by concatenating full-length human histone proteins, selected and arranged in reciprocal orientations as detailed in the present document (see below).

Three-dimensional models of the proteins presented in this study were obtained from AlphaFold predictions.[3] Specifically, structures were retrieved from the AlphaFold Protein Structure Database[4] using the BLASTP web interface for each sequence identified in the protein similarity searches.

Interaction distances at the interface of the histone H3–H4 dimer (PDB code 3AV2)[5] were calculated using a Python script implemented in PyMOL[6], with a distance cutoff of 4.5 Å. Polar interactions between H3–DNA and H4–DNA were identified using the "polar contacts" tool in PyMOL, considering a maximum distance cutoff of 3.6 Å.

To investigate the molecular evolution of sequences containing the H3-H4 dimer, a BLASTP search was conducted setting the minimum % identity to 75 and the minimum coverage to query 90 with respect to *Pongo pygmaeus* H3-H4 doublet (NCBI protein ID: XP_054346924). In this way, only sequences with at least an H3 and an H4 domain were collected. Then these sequences were down sampled to remove redundancy prior to analysis, by clustering at 85% min global sequence identity. Output used for rooting the tree is made by *Bos taurus* H3.3C, the closest characterized histone-like protein with < 75% identity to *Pongo pygmaeus* H3-H4 doublet and with a different function with respect to canonical H3.[7] These sequences were aligned using MAFFT v.7.525 with the "--genafpair" option,[8] incorporating structural information via the DASH option.[9] Gap positions shared by more than 90% of sequences were trimmed as non-informative. A rooted maximum likelihood (ML) tree was inferred with IQ-TREE multicore version 2.2.2.6,[10] using the best fit model according to both AIC and BIC criteria after a ModelFinder run.[11] Branch support was assessed using 1,000 ultrafast bootstrap replicates[12] and transfer bootstrap expectation.[13]

1. Altschul, S. F.; Madden, T. L.; Schäffer, A. A.; Zhang, J.; Zhang, Z.; Miller, W.; Lipman, D. J. Gapped BLAST and PSI-BLAST: A New Generation of Protein Database Search Programs. *Nucleic Acids Res.* **1997**, *25* (17), 3389–3402. https://doi.org/10.1093/nar/25.17.3389.
2. O'Donovan, C.; Apweiler, R. A Guide to UniProt for Protein Scientists. *Methods Mol. Biol. Clifton NJ* **2011**, *694*, 25–35. https://doi.org/10.1007/978-1-60761-977-2_2.
3. Jumper, J.; Evans, R.; Pritzel, A.; Green, T.; Figurnov, M.; Ronneberger, O.; Tunyasuvunakool, K.; Bates, R.; Žídek, A.; Potapenko, A. *et al*. Highly Accurate Protein Structure Prediction with AlphaFold. *Nature* **2021**, *596* (7873), 583–589. https://doi.org/10.1038/s41586-021-03819-2.
4. Varadi, M.; Bertoni, D.; Magana, P.; Paramval, U.; Pidruchna, I.; Radhakrishnan, M.; Tsenkov, M.; Nair, S.; Mirdita, M.; Yeo, J.; Kovalevskiy, O.; Tunyasuvunakool, K.; Laydon, A.; Žídek, A.; Tomlinson, H.; Hariharan, D.; Abrahamson, J.; Green, T.; Jumper,

J.; Birney, E.; Steinegger, M.; Hassabis, D.; Velankar, S. AlphaFold Protein Structure Database in 2024: Providing Structure Coverage for over 214 Million Protein Sequences. *Nucleic Acids Res.* **2024**, *52* (D1), D368–D375. https://doi.org/10.1093/nar/gkad1011.

5. Tachiwana, H.; Osakabe, A.; Shiga, T.; Miya, Y.; Kimura, H.; Kagawa, W.; Kurumizaka, H. Structures of Human Nucleosomes Containing Major Histone H3 Variants. *Acta Crystallogr D Biol Crystallogr* **2011**, *67* (6), 578–583. https://doi.org/10.1107/S0907444911014818.
6. DeLano, W.L. The PyMOL Molecular Graphics System **2002**, DeLano Scientific, San Carlos, CA, USA. http://www.pymol.org.
7. Wong, L. H.; Ren, H.; Williams, E. J.; McGhie, J. D. R.; Ahn, S.; Sim, M. L. J.; Tam, A.; Earle, E.; Anderson, M. A.; Mann, J. R.; Choo, K. H. A. Histone H3.3 Incorporation Provides a Unique and Functionally Essential Telomeric Chromatin in Embryonic Stem Cells. *Genome Res.* **2009**, *19* (3), 404–414. https://doi.org/10.1101/gr.084947.108
8. Wei, Y.; Zou, Q.; Tang, F.; Yu, L. WMSA: A Novel Method for Multiple Sequence Alignment of DNA Sequences. *Bioinformatics* **2022**, *38* (22), 5019–5025. https://doi.org/10.1093/bioinformatics/btac658
9. Kozewicki, J.; Li, S.; Amada, K. M.; Standley, D. M.; Katoh, K. MAFFT-DASH: Integrated Protein Sequence and Structural Alignment. *Nucleic Acids Res*. **2019**, 47, W5–W10. https://doi.org/10.1093/nar/gkz342
10. Minh, B. Q.; Schmidt, H. A.; Chernomor, O.; Schrempf, D.; Woodhams, M. D.; von Haeseler, A.; Lanfear, R. IQ-TREE 2: New Models and Efficient Methods for Phylogenetic Inference in the Genomic Era. *Mol. Biol. Evol.* **2020**, *37* (5), 1530–1534. https://doi.org/10.1093/molbev/msaa015
11. Kalyaanamoorthy, S.; Minh, B. Q.; Wong, T. K. F.; von Haeseler, A.; Jermiin, L. S. ModelFinder: Fast Model Selection for Accurate Phylogenetic Estimates. *Nat. Methods* **2017**, *14*, 587–589. https://doi.org/10.1038/nmeth.4285
12. Minh, B. Q.; Nguyen, M. A. T.; von Haeseler, A. Ultrafast Approximation for Phylogenetic Bootstrap. *Mol. Biol. Evol.* **2013**, *30* (5), 1188–1195. https://doi.org/10.1093/molbev/mst024
13. Lemoine, F.; Domelevo Entfellner, J.-B.; Wilkinson, E.; Correia, D.; Dávila Felipe, M.; De Oliveira, T.; Gascuel, O. Renewing Felsenstein's Phylogenetic Bootstrap in the Era of Big Data. *Nature* **2018**, *556*, 452–456. https://doi.org/10.1038/s41586-018-0043-0

Table S1: Non-bonded interactions at the H3–H4 dimerization interface (distance ≤ 4.5 Å), identified through analysis of the crystal structure of the human nucleosome core particle (PDB ID: 3AV2; H3 chains A and E, H4 chains B and F)[5].

H3 (chain A)	H4 (chain B)	Distance (Å)	H3 (chain E)	H4 (chain F)	Distance (Å)
E51	R40	3.2	E51	R40<br>R36	3.6<br>2.7
Q56	R40<br>R41	3.7<br>3.8	Q56	R40	3.2
S58	R41	3.0	S58	R41	3.2
E60	R41	3.6	E60	R41	3.1
L62	R37<br>L38	3.1<br>4.1	L62	R37<br>L38	3.0<br>4.1
I63	A34<br>L38	4.3<br>4.1	I63	A34<br>L38<br>I30	4.1<br>4.0<br>3.8

P67	G29	4.4	P67	G29	4.1
F68	L63	4.2	F68	L63	4.0
L71	L63<br>I27<br>I30<br>L59	3.7<br>3.6<br>3.3<br>3.7	L71	L63<br>I27<br>I30<br>L59	3.7<br>3.5<br>4.1<br>4.4
V72	I67	3.6	V72	I67	3.9
E74	N26	2.6	E74	N26	2.7
I75	I67<br>L63	4.1<br>4.5	I75	I67<br>L63	4.3<br>3.8
A76	I67	3.6	A76	I67	3.7
F79	I67<br>V70	3.9<br>4.3	F79	I67	4.2
			K80	E79	3.1
L83	V71<br>V82<br>T74	3.6<br>4.4<br>4.0	L83	V71<br>V82	3.8<br>4.1
F85	V82	3.6	F85	V82	3.9
A88			A88	A84<br>F101	3.9<br>4.3
A89	A84<br>V87	4.5<br>4.0	A89	A84<br>V87<br>V82	3.9<br>4.2<br>4.2
A92	L98<br>V87	3.9<br>4.3	A92	L98<br>V87	3.9<br>3.8
L93	V87<br>I67<br>L63<br>V66	3.6<br>4.1<br>4.5<br>3.5	L93	V87<br>I67<br><br>V66	3.5<br>4.4<br><br>3.7
A96	F62<br>L91	4.2<br>3.6	A96	F62<br>L91	4.4<br>3.7
Y100	F62<br>V58	3.6<br>3.8	Y100	F62	3.5
L101	L38<br>L59<br>T55	3.8<br>4.8<br>4.1	L101	L38<br>L59<br>V58<br>T55	3.2<br>4.1<br>4.4<br>4.0
V102	L38	4.1	V102	L38	3.7
L104	V58	3.6	L104	V58	3.7
F105	V44<br>A39<br>L38<br>I51<br>T55<br>I35	4.2<br>3.5<br>3.7<br>4.2<br>3.3<br>3.9	F105	V44<br>A39<br>L38<br>I51<br>T55<br>I35	4.2<br>4.1<br>4.3<br>4.1<br>3.9<br>3.8
N109	G43	3.0	N109	G43	2.9
T119	R46	2.8	T119	R46	2.8
I120	I51<br>I47<br>V44	3.6<br>3.8<br>3.5	I120	I51<br>I47<br>V44	4.2<br>3.8<br>3.8
P122	L50	3.3	P122	I51<br>L50	3.5<br>3.7
I125	I51<br>V58	3.9<br>4.0	I125	I51<br>V58	3.7<br>4.2
Q126	E54	2.8	Q126	E54	3.3

Table S2. Polar interactions, including salt bridges and hydrogen bonds (distance ≤ 3.5 Å), between histones H3 and H4 and DNA, identified from analysis of the crystal structure of the human nucleosome core particle (PDB ID: 3AV2; chains A and E for H3, and chains B and F for H4)[5]. Interactions mediated by water molecules are indicated by (H_2O) in parentheses.

**H3 (chain A)**	**Distance (Å)**	**H3 (chain E)**	**Distance (Å)**
R41	2.6 and 3.2	R41	2.8 and 3.2,
Y42	2.5 and 2.8	Y42	2.6, 3,5 and 3.6
R43	3.0 and 2.9	R43	2.8, 2.9 and 3.4
G45	3.0 and 3.1	G45	3.0 and 3.4
		T46	2.7
V47	2.9 and 2.9	V47	3.0 and 3.1
		R50	3.6
R54		R54	3.5
K57		K57	3.5
R64	3.6		
K65	2.7	K65	2.7
L66	2.8	L66	3.0
R70	2.9 and 2.7	R70	2.6
R73	3.4 and 3.3	R73	3.0 and 3.1
R84	3.3	R84	3.4
F85	2.4	F85	3.0
Q86		Q86	3.0
S87	2.9 (H_2O)		
V118	2.8	V118	2.8
T119	2.9 and 2.8	T119	2.7 and 2.9

**H4 (chain B)**	**Distance (Å)**	**H4 (chain F)**	**Distance (Å)**
		R20	3.3
R36	2.7		
R37	2.7 and 3.3	R37	3.0 and 3.1
R46	3.1 and 2.8	R46	3.4 and 3.0
		I47	2.7
S48	3.2		
		G49	3.3
K78	2.9		
K80	3.1	K80	2.6
T81	3.3 and 2.8	T81	2.9, and 3.5

Table S3: Proteins containing triplets of histone fold (HF) domains, identified using a chimeric query sequence generated by joining human histones H3 (113 amino acids) and H4 (103 amino acids) (H3H4 query). The domain organization along each sequence is shown, with domains predicted by AlphaFold to most likely form dimers indicated in parentheses. The percentage sequence identity relative to the isolated H3 and H4 domains is reported, along with the identity of residues involved in dimerization (see Table S1) and DNA binding (see Table S2).

Protein sequence ID[a] (amino acid length)	Organism	HF domain organization (AlphaFold)[b]	HF domain % identity	% identity of dimerization residues	% identity of DNA-binding residues
XP_064010917.1 (327)	*Pogoniulus pusillus*	(H4-H3)-H4	(99-79)-100	(100-82)-100	(100-90)-100
XP_022382829.1 (328)	*Enhydra lutris kenyoni*	H4-(H3-H4)	93-(80-100)	94-(82-100)	100-(90-100)
XP_059119301.1 (331)	*Peromyscus eremicus*	(H4-H3)-H4	(93-78)-100	(94-82)-100	(100-90)-100
XP_055943683.1 (344)	*Argiope bruennichi*	H4-(H3-H4)	96-(96-100)	97-(97-100)	100-(100-100)
XP_023977603.2 (355)	*Physeter macrocephalus*	H4-(H3-H4)	93-(96-100)	94-(97-100)	100-(100-100)
XP_027535400.1 (364)	*Neopelma chrysocephalum*	(H4-H3)-H4	(100-79)-100	(100-82)-100	(100-90)-100
XP_071151798.1 (365)	*Mytilus edulis*	H4-(H3-H4)	100-(97-100)	100-(97-100)	100-(100-100)
XP_058861958.1 (369)	*Acipenser ruthenus*	H4-(H3-H4)	92-(96-100)	94-(97-100)	100-(100-100)
XP_034546197.1[c] (373)	*Notolabrus celidotus*	(H4-H3)-H4	(99-80)-100	(100-82)-100	(100-90)-100
XP_028335332.1 (383)	*Physeter macrocephalus*	(H3-H4)-H3	(96-99)-96	(97-100)-97	(100-100)-100
CAN0156380.1 (404)	*Lampetra fluviatilis*	(H3-H4)-H3	(96-98)-96	(97-100)-97	(100-100)-100)
XP_014303395.1 (436)	*Myotis lucifugus*	(H3-H4)-H3	(96-100)-96	(97-100)-97	(100-100)-100
VDP36995.1[d] (383)	*Heligmosomoides polygyrus*	H2B-(H3-H4)	(94-99)	(97-97)	(100-100)

[a]NCBI RefSeq collection protein code. [b]Domains along the sequence that are involved in pseudo-dimerization are indicated in parentheses, based on the most probable AlphaFold prediction. [c]For this protein, the AlphaFold model is reported in Figure 3a. [d]For this protein, the conserved domain footprints scheme is reported in Figure 3d.

Table S4. Proteins showing triplets of histone folds (HF), retrieved using a chimeric sequence obtained by joining human histones H4 (103 amino acids) and H3 (113 amino acids) sequences (H4H3 query). The domain organization along each sequence is shown, with domains predicted by AlphaFold to most likely form dimers indicated in parentheses. The percentage sequence identity relative to the isolated H3 and H4 domains is reported, along with the identity of residues involved in dimerization (see Table S1) and DNA binding (see Table S2). Protein sequences already identified with the H3H4 query (see Table S3), namely XP_064010917.1, XP_055943683.1, XP_023977603.2, XP_071151798.1, XP_058861958.1, XP_028335332.1, CAN0156380.1, XP_014303395.1, are not shown again in this table.

Protein sequence ID[a] (amino acid length)	Organism	HF domain organization (AlphaFold)[b]	HF domain % identity	% identity of dimerization residues	% identity of DNA-binding residues
XP_034841793.1 (310)	*Mirounga leonina*	(H4-H3)-H4	(100-78)-72	(100-82)-60	(100-90)-80
XP_055645334.1 (321)	*Toxorhynchites rutilus septentrionalis*	(H4-H3)-H4	(98-78)-99	(100-82)-100	(100-90)-100
XP_064169827.1 (323)	*Anguilla rostrata*	(H4-H3)-H4	(100-82)-100	(100-82)-100	(100-90)-100
XP_063836701.1 (326)	*Ostrinia nubilalis*	H4-(H3-H4)	96-(82-100)	97-(82-100)	100-(90-100)
XP_023818559.1 (329)	*Oryzias latipes*	(H4)-H4-(H3)	(100)-71-(83)	(100)-91-(82)	(100)- 90-(100)
XP_064367322.1 (329)	*Dromaius novaehollandiae*	H4-(H3-H4)	93-(83-100)	94-(82-100)	100-(90-100)
XP_059672939.1 (332)	*Gavia stellata*	(H4-H3)-H4	(100-83)-100	(100-82)-100	(100-90)-100
XP_063785865.1 (344)	*Pseudophryne corroboree*	(H4-H3)-H4	(99-79)-100	(100-79)-100	(100-90)-100
TEA21837.1 (346)	*Sousa chinensis*	H4-(H3-H4)	100-(82-100)	100-(82-100)	100-(90-100)
XP_042638117.1 (358)	*Orycteropus afer afer*	H3-(H4-H3)	96-(66-97)	97-(89-97)	100-(90-100)
XP_055879400.1 (366)	*Biomphalaria glabrata*	(H4-H3)-H4	(100-83)-100	(100-82)-100	(100-90)-100
KAG8140557.1 (369)	*Naja naja*	(H4)-H4-(H3)	(100)-93-(87)	(100)- 97-(91)	(100)-100-(100)
XP_037558005.2 (371)	*Dermacentor silvarum*	H4-(H3-H4)	96-(79-100)	97-(82-100)	100-(90-100)
XP_063442209.1 (374)	*Mytilus trossulus*	(H3-H4)-H3	(96-91)-97	(97-94)-97	(100-100)-100
XP_043539660.1 (384)	*Chiloscyllium plagiosum*	H3-(H4-H3)	95-(82-97)	97-(91-97)	100-(100-100)
XP_046714411.1 (400)	*Silurus meridionalis*	H4-(H3-H4)	(99-96)-100	(100-97)-100	(100-100)-100
XP_002816573.3 (406)	*Pongo abelii*	(H4-H3)-H4	(98-78)-100	(100-82)-100	(100-90)-100
VDK57329.1[c] (411)	*Anisakis simplex*	H2A-(H4-H3)	(99-97)	(100-97)	(100-100)
XP_035205101.1 (412)	*Stegodyphus dumicola*	(H3-H4)-H3	(96-98)-97	(97-100)- 97	(100-100)-100
XP_055262377.1 (418)	*Moschus berezovskii*	H3-(H4-H3)	96-(90-97)	97-(94-97)	100-(100-100)
XP_061053392.1 (419)	*Eubalaena glacialis*	(H3-H4)-H3	(96-95)-96	(97-97)-97	(100-100)-100
XP_058861937.1 (422)	*Acipenser ruthenus*	H4-(H3-H4)	95-(96-100)	97-(97-100)	100-(100-100)
XP_064908964.1 (423)	*Columba livia*	H4-(H3-H4)	93-(97-100)	94-(97-100)	100-(100-100)
XP_036987727.2 (426)	*Artibeus jamaicensis*	H3-(H4-H3)	96-(85-96)	97-(91-97)	100-(100-100)
XP_032126308.1 (428)	*Sapajus apella*	(H3-H4)-H3	(96-98)-96	(97-100)- 97	(100-100)-100
XP_031749188.1 (430)	*Xenopus tropicalis*	H4-(H3-H4)	94-(97-100)	97-(97-100)	100-(100-100)
XP_041260510.1 (431)	*Onychostruthus taczanowskii*	(H4-H3)-H4	(100-98)-100	(100-97)-100	(100-100)-100
XP_056410747.1 (445)	*Hyla sarda*	H4-(H3-H4)	(92-93)-100	(94-97)-100	(100-100)-100

[a]NCBI RefSeq collection protein code. [b]Domains along the sequence that are involved in pseudo-dimerization are indicated in parentheses, based on the most probable AlphaFold prediction. [c]For this protein, the AlphaFold model is reported in Figure 4.

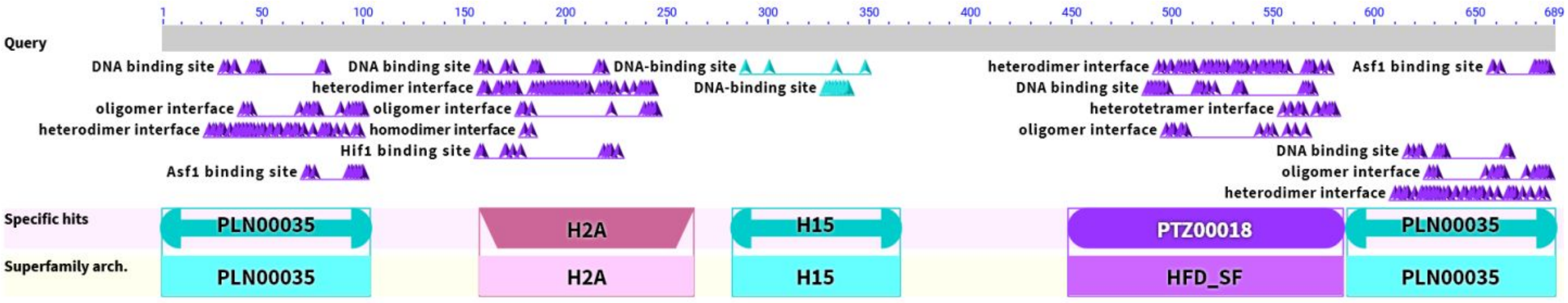

Name	Accession	Description	Interval	E-value
+ PTZ00018	PTZ00018	histone H3	449-584	6.75×10^{-89}
+ H2A	smart00414	Histone 2A	158-263	2.02×10^{-64}
+ PLN00035	PLN00035	histone H4	587-689	1.54×10^{-55}
+ PLN00035	PLN00035	histone H4	1-103	3.73×10^{-55}
+ H15	cd00073	linker histone 1 and histone 5 domains	283-365	6.94×10^{-31}

Figure S1. NCBI Conserved Domain Database (CDD) record for protein XP_056017360.1 expressed in *Ostrea edulis*, with specification of locations of conserved protein domain footprints; name, accession number, and description of the inferred structural motifs are reported, together with the sequence interval that corresponds to each motif and the E-value associated to each assignment

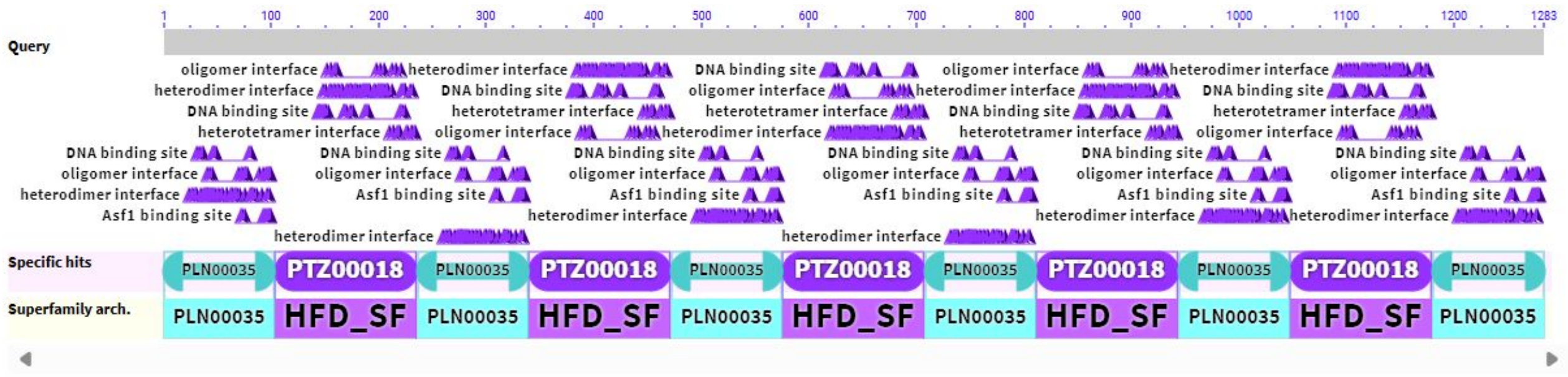

Name	Accession	Description	Interval	E-value
PTZ00018	PTZ00018	histone H3	340-470	7.69×10^{-79}
PTZ00018	PTZ00018	histone H3	576-706	7.69×10^{-79}
PTZ00018	PTZ00018	histone H3	812-942	7.69×10^{-79}
PTZ00018	PTZ00018	histone H3	1048-1178	7.69×10^{-79}
PTZ00018	PTZ00018	histone H3	104-234	7.69×10^{-79}
PLN00035	PLN00035	histone H4	1181-1283	3.82×10^{-52}
PLN00035	PLN00035	histone H4	945-1046	1.11×10^{-51}
PLN00035	PLN00035	histone H4	709-810	1.11×10^{-51}
PLN00035	PLN00035	histone H4	237-338	1.11×10^{-51}
PLN00035	PLN00035	histone H4	1-102	1.11×10^{-51}
PLN00035	PLN00035	histone H4	473-574	1.11×10^{-51}

Figure S2. NCBI Conserved Domain Database (CDD) record for protein CAL5913199 found in *Lampetra fluviatilis*, with specification of locations of conserved protein domain footprints; name, accession number, and description of the inferred structural motifs are reported, together with the sequence interval that corresponds to each motif and the E-value associated to each assignment

H3H4 query sequence and obtained alignments through BLAST searches

<u>Query sequence</u>:

MARTKQTARKSTGGKAPRKQLATKAARKSAPSTGGVKKPHRYRPGTVALREIRRYQKSTE
LLIRKLPFQRLVREIAQDFKTDLRFQSAAIGALQEASEAYLVGLFEDTNLCAIHAKRVTIMP
KDIQLARRIRGERAMSGRGKGGKGLGKGGAKRHRKVLRDNIQGITKPAIRRLARRGGVKR
ISGLIYEETRGVLKVFLENVIRDAVTYTEHAKRKTVTAMDVVYALKRQGRTLYGFGG

<u>Obtained alignments between the query and the BLAST-retrieved sequences</u>:

```
>uncharacterized protein [Mytilus edulis]
Sequence ID: XP_071151798.1 Length: 365
Range 1: 127 to 365
```

```
Score:469 bits(1206), Expect:2e-164,
Method:Compositional matrix adjust.,
Identities:235/239(98%), Positives:238/239(99%), Gaps:0/239(0%)

Query  1    MARTKQTARKSTGGKAPRKQLATKAARKSAPSTGGVKKPHRYRPGTVALREIRRYQKSTE  60
            MARTKQTARKSTGGKAPRKQLATKAARKSAP+TGGVKKPHRYRPGTVALREIRRYQKSTE
Sbjct  127  MARTKQTARKSTGGKAPRKQLATKAARKSAPATGGVKKPHRYRPGTVALREIRRYQKSTE  186

Query  61   LLIRKLPFQRLVREIAQDFKTDLRFQSAAIGALQEASEAYLVGLFEDTNLCAIHAKRVTI  120
            LLIRKLPFQRLVREIAQDFKTDLRFQS+A+ ALQEASEAYLVGLFEDTNLCAIHAKRVTI
Sbjct  187  LLIRKLPFQRLVREIAQDFKTDLRFQSSAVMALQEASEAYLVGLFEDTNLCAIHAKRVTI  246

Query  121  MPKDIQLARRIRGERAMSGRGKGGKGLGKGGAKRHRKVLRDNIQGITKPAIRRLARRGGV  180
            MPKDIQLARRIRGERAMSGRGKGGKGLGKGGAKRHRKVLRDNIQGITKPAIRRLARRGGV
Sbjct  247  MPKDIQLARRIRGERAMSGRGKGGKGLGKGGAKRHRKVLRDNIQGITKPAIRRLARRGGV  306

Query  181  KRISGLIYEETRGVLKVFLENVIRDAVTYTEHAKRKTVTAMDVVYALKRQGRTLYGFGG  239
            KRISGLIYEETRGVLKVFLENVIRDAVTYTEHAKRKTVTAMDVVYALKRQGRTLYGFGG
Sbjct  307  KRISGLIYEETRGVLKVFLENVIRDAVTYTEHAKRKTVTAMDVVYALKRQGRTLYGFGG  365



Range 2: 1 to 103

Score:206 bits(523), Expect:5e-61,
Method:Compositional matrix adjust.,
Identities:103/103(100%), Positives:103/103(100%), Gaps:0/103(0%)

Query  137  MSGRGKGGKGLGKGGAKRHRKVLRDNIQGITKPAIRRLARRGGVKRISGLIYEETRGVLK  196
            MSGRGKGGKGLGKGGAKRHRKVLRDNIQGITKPAIRRLARRGGVKRISGLIYEETRGVLK
Sbjct  1    MSGRGKGGKGLGKGGAKRHRKVLRDNIQGITKPAIRRLARRGGVKRISGLIYEETRGVLK  60

Query  197  VFLENVIRDAVTYTEHAKRKTVTAMDVVYALKRQGRTLYGFGG  239
            VFLENVIRDAVTYTEHAKRKTVTAMDVVYALKRQGRTLYGFGG
Sbjct  61   VFLENVIRDAVTYTEHAKRKTVTAMDVVYALKRQGRTLYGFGG  103



>hypothetical protein R6Z07_017237 [Ovis aries]
Sequence ID: XDA87243.1 Length: 242
Range 1: 1 to 242

Score:463 bits(1191), Expect:4e-164,
Method:Compositional matrix adjust.,
Identities:234/242(97%), Positives:237/242(97%), Gaps:3/242(1%)

Query  1    MARTKQTARKSTGGKAPRKQLATKAARKSAPSTGGVKKPHRYRPGTVALREIRRYQKSTE  60
            MARTKQTARKSTGGKAPRKQLATKAARKSAP+TGGVKKPHRYRPGTVALREIRRYQKSTE
Sbjct  1    MARTKQTARKSTGGKAPRKQLATKAARKSAPATGGVKKPHRYRPGTVALREIRRYQKSTE  60

Query  61   LLIRKLPFQRLVREIAQDFKTDLRFQSAAIGALQEASEAYLVGLFEDTNLCAIHAKRVTI  120
            LLIRKLPFQRLVREIAQDFKTDLRFQS+A+ ALQEA EAYLVGLFEDTNLCAIHAKRVTI
Sbjct  61   LLIRKLPFQRLVREIAQDFKTDLRFQSSAVMALQEACEAYLVGLFEDTNLCAIHAKRVTI  120

Query  121  MPKDIQLARRIRGERA---MSGRGKGGKGLGKGGAKRHRKVLRDNIQGITKPAIRRLARR  177
            MPKDIQLARRIRGERA   MSGRGKGGKGLGKGGAKRHRKVLRDNIQGITKPAIRRLARR
Sbjct  121  MPKDIQLARRIRGERASGSMSGRGKGGKGLGKGGAKRHRKVLRDNIQGITKPAIRRLARR  180

Query  178  GGVKRISGLIYEETRGVLKVFLENVIRDAVTYTEHAKRKTVTAMDVVYALKRQGRTLYGF  237
            GGVKRISGLIYEETRGVLKVFLENVIRDAVTYTEHAKRKTVTAMDVVYALKRQGRTLYGF
Sbjct  181  GGVKRISGLIYEETRGVLKVFLENVIRDAVTYTEHAKRKTVTAMDVVYALKRQGRTLYGF  240
```

```
Query  238  GG  239
            GG
Sbjct  241  GG  242
```

>uncharacterized protein LOC113264214 [Ursus arctos]
Sequence ID: XP_057160765.1 Length: 471
Range 1: 1 to 239

Score:468 bits(1204), Expect:1e-162,
Method:Compositional matrix adjust.,
Identities:233/239(97%), Positives:236/239(98%), Gaps:0/239(0%)

```
Query  1    MARTKQTARKSTGGKAPRKQLATKAARKSAPSTGGVKKPHRYRPGTVALREIRRYQKSTE  60
            MARTKQTARKSTGGKAPRKQLATKAARKSAP+TGGVKKPHRYRPGTVALREIRRYQKSTE
Sbjct  1    MARTKQTARKSTGGKAPRKQLATKAARKSAPATGGVKKPHRYRPGTVALREIRRYQKSTE  60

Query  61   LLIRKLPFQRLVREIAQDFKTDLRFQSAAIGALQEASEAYLVGLFEDTNLCAIHAKRVTI  120
            LLIRKLPFQRLVREIAQDFKTDLRFQS+A+ ALQEA EAYLVGLFEDTNLCAIHAKRVTI
Sbjct  61   LLIRKLPFQRLVREIAQDFKTDLRFQSSAVMALQEACEAYLVGLFEDTNLCAIHAKRVTI  120

Query  121  MPKDIQLARRIRGERAMSGRGKGGKGLGKGGAKRHRKVLRDNIQGITKPAIRRLARRGGV  180
            MPKDIQLARRIRGERAMSGRGKGGKGLGKGGAKRHRKVLRDNIQGITKPAIRRLARRGGV
Sbjct  121  MPKDIQLARRIRGERAMSGRGKGGKGLGKGGAKRHRKVLRDNIQGITKPAIRRLARRGGV  180

Query  181  KRISGLIYEETRGVLKVFLENVIRDAVTYTEHAKRKTVTAMDVVYALKRQGRTLYGFGG  239
            KRISGLIYEETRGVLKVFLENVIRDAVTYTEHAKRKTVTAMDVVYALKRQGRTLYGFG
Sbjct  181  KRISGLIYEETRGVLKVFLENVIRDAVTYTEHAKRKTVTAMDVVYALKRQGRTLYGFGA  239
```

Range 2: 336 to 471

Score:265 bits(678), Expect:5e-83,
Method:Compositional matrix adjust.,
Identities:131/136(96%), Positives:134/136(98%), Gaps:0/136(0%)

```
Query  1    MARTKQTARKSTGGKAPRKQLATKAARKSAPSTGGVKKPHRYRPGTVALREIRRYQKSTE  60
            MARTKQTARKSTGGKAPRKQLATKAARKSAP+TGGVKKPHRYRPGTVALREIRRYQKSTE
Sbjct  336  MARTKQTARKSTGGKAPRKQLATKAARKSAPATGGVKKPHRYRPGTVALREIRRYQKSTE  395

Query  61   LLIRKLPFQRLVREIAQDFKTDLRFQSAAIGALQEASEAYLVGLFEDTNLCAIHAKRVTI  120
            LLIRKLPFQRLVREIAQDFKTDLRFQS+A+ ALQEA EAYLVGLFEDTNLCAIHAKRVTI
Sbjct  396  LLIRKLPFQRLVREIAQDFKTDLRFQSSAVMALQEACEAYLVGLFEDTNLCAIHAKRVTI  455

Query  121  MPKDIQLARRIRGERA  136
            MPKDIQLARRIRGERA
Sbjct  456  MPKDIQLARRIRGERA  471
```

>uncharacterized protein LOC102419892 [Myotis lucifugus]
Sequence ID: XP_014303395.1 Length: 436
Range 1: 36 to 275

Score:465 bits(1196), Expect:7e-162,
Method:Compositional matrix adjust.,
Identities:234/240(98%), Positives:237/240(98%), Gaps:1/240(0%)

```
Query  1    MARTKQTARKSTGGKAPRKQLATKAARKSAPSTGGVKKPHRYRPGTVALREIRRYQKSTE  60
            MARTKQTARKSTGGKAPRKQLATKAARKSAP+TGGVKKPHRYRPGTVALREIRRYQKSTE
Sbjct  36   MARTKQTARKSTGGKAPRKQLATKAARKSAPATGGVKKPHRYRPGTVALREIRRYQKSTE  95

Query  61   LLIRKLPFQRLVREIAQDFKTDLRFQSAAIGALQEASEAYLVGLFEDTNLCAIHAKRVTI  120
            LLIRKLPFQRLVREIAQDFKTDLRFQS+A+ ALQEA EAYLVGLFEDTNLCAIHAKRVTI
Sbjct  96   LLIRKLPFQRLVREIAQDFKTDLRFQSSAVMALQEACEAYLVGLFEDTNLCAIHAKRVTI  155

Query  121  MPKDIQLARRIRGERA-MSGRGKGGKGLGKGGAKRHRKVLRDNIQGITKPAIRRLARRGG  179
            MPKDIQLARRIRGERA MSGRGKGGKGLGKGGAKRHRKVLRDNIQGITKPAIRRLARRGG
Sbjct  156  MPKDIQLARRIRGERASMSGRGKGGKGLGKGGAKRHRKVLRDNIQGITKPAIRRLARRGG  215

Query  180  VKRISGLIYEETRGVLKVFLENVIRDAVTYTEHAKRKTVTAMDVVYALKRQGRTLYGFGG  239
            VKRISGLIYEETRGVLKVFLENVIRDAVTYTEHAKRKTVTAMDVVYALKRQGRTLYGFGG
Sbjct  216  VKRISGLIYEETRGVLKVFLENVIRDAVTYTEHAKRKTVTAMDVVYALKRQGRTLYGFGG  275
```

Range 2: 301 to 436

Score:265 bits(678), Expect:2e-83,
Method:Compositional matrix adjust.,
Identities:131/136(96%), Positives:134/136(98%), Gaps:0/136(0%)

```
Query  1    MARTKQTARKSTGGKAPRKQLATKAARKSAPSTGGVKKPHRYRPGTVALREIRRYQKSTE  60
            MARTKQTARKSTGGKAPRKQLATKAARKSAP+TGGVKKPHRYRPGTVALREIRRYQKSTE
Sbjct  301  MARTKQTARKSTGGKAPRKQLATKAARKSAPATGGVKKPHRYRPGTVALREIRRYQKSTE  360

Query  61   LLIRKLPFQRLVREIAQDFKTDLRFQSAAIGALQEASEAYLVGLFEDTNLCAIHAKRVTI  120
            LLIRKLPFQRLVREIAQDFKTDLRFQS+A+ ALQEA EAYLVGLFEDTNLCAIHAKRVTI
Sbjct  361  LLIRKLPFQRLVREIAQDFKTDLRFQSSAVMALQEACEAYLVGLFEDTNLCAIHAKRVTI  420

Query  121  MPKDIQLARRIRGERA  136
            MPKDIQLARRIRGERA
Sbjct  421  MPKDIQLARRIRGERA  436
```

>LOW QUALITY PROTEIN: uncharacterized protein LOC129974918 [Argiope bruennichi]
Sequence ID: XP_055943683.1 Length: 344
Range 1: 107 to 344

Score:461 bits(1186), Expect:1e-161,
Method:Compositional matrix adjust.,
Identities:233/239(97%), Positives:236/239(98%), Gaps:1/239(0%)

```
Query  1    MARTKQTARKSTGGKAPRKQLATKAARKSAPSTGGVKKPHRYRPGTVALREIRRYQKSTE  60
            MARTKQTARKSTGGKAPRKQLATKAARKSAP+TGGVKKPHRYRPGTVALREIRRYQKSTE
Sbjct  107  MARTKQTARKSTGGKAPRKQLATKAARKSAPATGGVKKPHRYRPGTVALREIRRYQKSTE  166

Query  61   LLIRKLPFQRLVREIAQDFKTDLRFQSAAIGALQEASEAYLVGLFEDTNLCAIHAKRVTI  120
            LLIRKLPFQRLVREIAQDFKTDLRFQS+A+ ALQEASEAYLVGLFEDTNLCAIHAKRVTI
Sbjct  167  LLIRKLPFQRLVREIAQDFKTDLRFQSSAVMALQEASEAYLVGLFEDTNLCAIHAKRVTI  226

Query  121  MPKDIQLARRIRGERAMSGRGKGGKGLGKGGAKRHRKVLRDNIQGITKPAIRRLARRGGV  180
            MPKDIQLARRIRGE  MSGRGKGGKGLGKGGAKRHRKVLRDNIQGITKPAIRRLARRGGV
Sbjct  227  MPKDIQLARRIRGE-LMSGRGKGGKGLGKGGAKRHRKVLRDNIQGITKPAIRRLARRGGV  285

Query  181  KRISGLIYEETRGVLKVFLENVIRDAVTYTEHAKRKTVTAMDVVYALKRQGRTLYGFGG  239
            KRISGLIYEETRGVLKVFLENVIRDAVTYTEHAKRKTVTAMDVVYALKRQGRTLYGFGG
```

```
Sbjct  286  KRISGLIYEETRGVLKVFLENVIRDAVTYTEHAKRKTVTAMDVVYALKRQGRTLYGFGG  344
```

Range 2: 1 to 99

Score:197 bits(500), Expect:9e-58,
Method:Compositional matrix adjust.,
Identities:99/99(100%), Positives:99/99(100%), Gaps:0/99(0%)

```
Query  137  MSGRGKGGKGLGKGGAKRHRKVLRDNIQGITKPAIRRLARRGGVKRISGLIYEETRGVLK  196
            MSGRGKGGKGLGKGGAKRHRKVLRDNIQGITKPAIRRLARRGGVKRISGLIYEETRGVLK
Sbjct  1    MSGRGKGGKGLGKGGAKRHRKVLRDNIQGITKPAIRRLARRGGVKRISGLIYEETRGVLK  60

Query  197  VFLENVIRDAVTYTEHAKRKTVTAMDVVYALKRQGRTLY  235
            VFLENVIRDAVTYTEHAKRKTVTAMDVVYALKRQGRTLY
Sbjct  61   VFLENVIRDAVTYTEHAKRKTVTAMDVVYALKRQGRTLY  99
```

>uncharacterized protein [Physeter macrocephalus]
Sequence ID: XP_023977603.2 Length: 355
Range 1: 111 to 355

Score:461 bits(1185), Expect:2e-161,
Method:Compositional matrix adjust.,
Identities:234/245(96%), Positives:237/245(96%), Gaps:6/245(2%)

```
Query  1    MARTKQTARKSTGGKAPRKQLATKAARKSAPSTGGVKKPHRYRPGTVALREIRRYQKSTE  60
            MARTKQTARKSTGGKAPRKQLATKAARKSAP+TGGVKKPHRYRPGTVALREIRRYQKSTE
Sbjct  111  MARTKQTARKSTGGKAPRKQLATKAARKSAPATGGVKKPHRYRPGTVALREIRRYQKSTE  170

Query  61   LLIRKLPFQRLVREIAQDFKTDLRFQSAAIGALQEASEAYLVGLFEDTNLCAIHAKRVTI  120
            LLIRKLPFQRLVREIAQDFKTDLRFQS+A+ ALQEA EAYLVGLFEDTNLCAIHAKRVTI
Sbjct  171  LLIRKLPFQRLVREIAQDFKTDLRFQSSAVMALQEACEAYLVGLFEDTNLCAIHAKRVTI  230

Query  121  MPKDIQLARRIRGER------AMSGRGKGGKGLGKGGAKRHRKVLRDNIQGITKPAIRRL  174
            MPKDIQLARRIRGER      AMSGRGKGGKGLGKGGAKRHRKVLRDNIQGITKPAIRRL
Sbjct  231  MPKDIQLARRIRGERGITATVAMSGRGKGGKGLGKGGAKRHRKVLRDNIQGITKPAIRRL  290

Query  175  ARRGGVKRISGLIYEETRGVLKVFLENVIRDAVTYTEHAKRKTVTAMDVVYALKRQGRTL  234
            ARRGGVKRISGLIYEETRGVLKVFLENVIRDAVTYTEHAKRKTVTAMDVVYALKRQGRTL
Sbjct  291  ARRGGVKRISGLIYEETRGVLKVFLENVIRDAVTYTEHAKRKTVTAMDVVYALKRQGRTL  350

Query  235  YGFGG  239
            YGFGG
Sbjct  351  YGFGG  355
```

Range 2: 1 to 96

Score:190 bits(482), Expect:5e-55,
Method:Compositional matrix adjust.,
Identities:96/96(100%), Positives:96/96(100%), Gaps:0/96(0%)

```
Query  137  MSGRGKGGKGLGKGGAKRHRKVLRDNIQGITKPAIRRLARRGGVKRISGLIYEETRGVLK  196
            MSGRGKGGKGLGKGGAKRHRKVLRDNIQGITKPAIRRLARRGGVKRISGLIYEETRGVLK
Sbjct  1    MSGRGKGGKGLGKGGAKRHRKVLRDNIQGITKPAIRRLARRGGVKRISGLIYEETRGVLK  60
```

```
Query  197  VFLENVIRDAVTYTEHAKRKTVTAMDVVYALKRQGR  232
            VFLENVIRDAVTYTEHAKRKTVTAMDVVYALKRQGR
Sbjct  61   VFLENVIRDAVTYTEHAKRKTVTAMDVVYALKRQGR  96
```

>uncharacterized protein [Physeter macrocephalus]
Sequence ID: XP_028335332.1 Length: 383
Range 1: 1 to 246

Score:460 bits(1184), Expect:8e-161,
Method:Compositional matrix adjust.,
Identities:233/246(95%), Positives:236/246(95%), Gaps:8/246(3%)

```
Query  1    MARTKQTARKSTGGKAPRKQLATKAARKSAPSTGGVKKPHRYRPGTVALREIRRYQKSTE  60
            MARTKQTARKSTGGKAPRKQLATKAARKSAP+TGGVKKPHRYRPGTVALREIRRYQKSTE
Sbjct  1    MARTKQTARKSTGGKAPRKQLATKAARKSAPATGGVKKPHRYRPGTVALREIRRYQKSTE  60

Query  61   LLIRKLPFQRLVREIAQDFKTDLRFQSAAIGALQEASEAYLVGLFEDTNLCAIHAKRVTI  120
            LLIRKLPFQRLVREIAQDFKTDLRFQS+A+ ALQEA EAYLVGLFEDTNLCAIHAKRVTI
Sbjct  61   LLIRKLPFQRLVREIAQDFKTDLRFQSSAVMALQEACEAYLVGLFEDTNLCAIHAKRVTI  120

Query  121  MPKDIQLARRIRGERA--------MSGRGKGGKGLGKGGAKRHRKVLRDNIQGITKPAIR  172
            MPKDIQLARRIRGERA        MSGRGKGGKGLGKGGAKRHRKVLRDNIQGITKPAIR
Sbjct  121  MPKDIQLARRIRGERATVPDPVVVMSGRGKGGKGLGKGGAKRHRKVLRDNIQGITKPAIR  180

Query  173  RLARRGGVKRISGLIYEETRGVLKVFLENVIRDAVTYTEHAKRKTVTAMDVVYALKRQGR  232
            RLARRGGVKRISGLIYEETRGVLKVFLENVIRDAVTYTEHAKRKTVTAMDVVYALKRQGR
Sbjct  181  RLARRGGVKRISGLIYEETRGVLKVFLENVIRDAVTYTEHAKRKTVTAMDVVYALKRQGR  240

Query  233  TLYGFG  238
            TLYGFG
Sbjct  241  TLYGFG  246
```

Range 2: 248 to 383

Score:265 bits(678), Expect:4e-84,
Method:Compositional matrix adjust.,
Identities:131/136(96%), Positives:134/136(98%), Gaps:0/136(0%)

```
Query  1    MARTKQTARKSTGGKAPRKQLATKAARKSAPSTGGVKKPHRYRPGTVALREIRRYQKSTE  60
            MARTKQTARKSTGGKAPRKQLATKAARKSAP+TGGVKKPHRYRPGTVALREIRRYQKSTE
Sbjct  248  MARTKQTARKSTGGKAPRKQLATKAARKSAPATGGVKKPHRYRPGTVALREIRRYQKSTE  307

Query  61   LLIRKLPFQRLVREIAQDFKTDLRFQSAAIGALQEASEAYLVGLFEDTNLCAIHAKRVTI  120
            LLIRKLPFQRLVREIAQDFKTDLRFQS+A+ ALQEA EAYLVGLFEDTNLCAIHAKRVTI
Sbjct  308  LLIRKLPFQRLVREIAQDFKTDLRFQSSAVMALQEACEAYLVGLFEDTNLCAIHAKRVTI  367

Query  121  MPKDIQLARRIRGERA  136
            MPKDIQLARRIRGERA
Sbjct  368  MPKDIQLARRIRGERA  383
```

>uncharacterized protein LOC108899806 [Lates calcarifer]
Sequence ID: XP_050933830.1 Length: 682
Range 1: 1 to 240

```
Score:471 bits(1212), Expect:8e-161,
Method:Compositional matrix adjust.,
Identities:234/240(98%), Positives:237/240(98%), Gaps:2/240(0%)

Query  1    MARTKQTARKSTGGKAPRKQLATKAARKSAPSTGGVKKPHRYRPGTVALREIRRYQKSTE  60
            MARTKQTARKSTGGKAPRKQLATKAARKSAP+TGGVKKPHRYRPGTVALREIRRYQKSTE
Sbjct  1    MARTKQTARKSTGGKAPRKQLATKAARKSAPATGGVKKPHRYRPGTVALREIRRYQKSTE  60

Query  61   LLIRKLPFQRLVREIAQDFKTDLRFQSAAIGALQEASEAYLVGLFEDTNLCAIHAKRVTI  120
            LLIRKLPFQRLVREIAQDFKTDLRFQS+A+ ALQEASEAYLVGLFEDTNLCAIHAKRVTI
Sbjct  61   LLIRKLPFQRLVREIAQDFKTDLRFQSSAVMALQEASEAYLVGLFEDTNLCAIHAKRVTI  120

Query  121  MPKDIQLARRIRGERA--MSGRGKGGKGLGKGGAKRHRKVLRDNIQGITKPAIRRLARRG  178
            MPKDIQLARRIRGERA  MSGRGKGGKGLGKGGAKRHRKVLRDNIQGITKPAIRRLARRG
Sbjct  121  MPKDIQLARRIRGERALNMSGRGKGGKGLGKGGAKRHRKVLRDNIQGITKPAIRRLARRG  180

Query  179  GVKRISGLIYEETRGVLKVFLENVIRDAVTYTEHAKRKTVTAMDVVYALKRQGRTLYGFG  238
            GVKRISGLIYEETRGVLKVFLENVIRDAVTYTEHAKRKTVTAMDVVYALKRQGRTLYGFG
Sbjct  181  GVKRISGLIYEETRGVLKVFLENVIRDAVTYTEHAKRKTVTAMDVVYALKRQGRTLYGFG  240



Range 2: 547 to 682

Score:270 bits(690), Expect:1e-82,
Method:Compositional matrix adjust.,
Identities:132/136(97%), Positives:135/136(99%), Gaps:0/136(0%)

Query  1    MARTKQTARKSTGGKAPRKQLATKAARKSAPSTGGVKKPHRYRPGTVALREIRRYQKSTE  60
            MARTKQTARKSTGGKAPRKQLATKAARKSAP+TGGVKKPHRYRPGTVALREIRRYQKSTE
Sbjct  547  MARTKQTARKSTGGKAPRKQLATKAARKSAPATGGVKKPHRYRPGTVALREIRRYQKSTE  606

Query  61   LLIRKLPFQRLVREIAQDFKTDLRFQSAAIGALQEASEAYLVGLFEDTNLCAIHAKRVTI  120
            LLIRKLPFQRLVREIAQDFKTDLRFQS+A+ ALQEASEAYLVGLFEDTNLCAIHAKRVTI
Sbjct  607  LLIRKLPFQRLVREIAQDFKTDLRFQSSAVMALQEASEAYLVGLFEDTNLCAIHAKRVTI  666

Query  121  MPKDIQLARRIRGERA  136
            MPKDIQLARRIRGERA
Sbjct  667  MPKDIQLARRIRGERA  682



>histone H3 [Chelonia mydas]
Sequence ID: XP_037744148.1 Length: 259
Range 1: 1 to 259

Score:455 bits(1170), Expect:1e-160,
Method:Compositional matrix adjust.,
Identities:234/259(90%), Positives:237/259(91%), Gaps:20/259(7%)

Query  1    MARTKQTARKSTGGKAPRKQLATKAARKSAPSTGGVKKPHRYRPGTVALREIRRYQKSTE  60
            MARTKQTARKSTGGKAPRKQLATKAARKSAP+TGGVKKPHRYRPGTVALREIRRYQKSTE
Sbjct  1    MARTKQTARKSTGGKAPRKQLATKAARKSAPATGGVKKPHRYRPGTVALREIRRYQKSTE  60

Query  61   LLIRKLPFQRLVREIAQDFKTDLRFQSAAIGALQEASEAYLVGLFEDTNLCAIHAKRVTI  120
            LLIRKLPFQRLVREIAQDFKTDLRFQS+A+ ALQEASEAYLVGLFEDTNLCAIHAKRVTI
Sbjct  61   LLIRKLPFQRLVREIAQDFKTDLRFQSSAVMALQEASEAYLVGLFEDTNLCAIHAKRVTI  120

Query  121  MPKDIQLARRIRGER--------------------AMSGRGKGGKGLGKGGAKRHRKVLR  160
            MPKDIQLARRIRGER                     MSGRGKGGKGLGKGGAKRHRKVLR
Sbjct  121  MPKDIQLARRIRGERLSPWPAPEDTFLFVLPRLRVEMSGRGKGGKGLGKGGAKRHRKVLR  180
```

```
Query  161  DNIQGITKPAIRRLARRGGVKRISGLIYEETRGVLKVFLENVIRDAVTYTEHAKRKTVTA  220
            DNIQGITKPAIRRLARRGGVKRISGLIYEETRGVLKVFLENVIRDAVTYTEHAKRKTVTA
Sbjct  181  DNIQGITKPAIRRLARRGGVKRISGLIYEETRGVLKVFLENVIRDAVTYTEHAKRKTVTA  240

Query  221  MDVVYALKRQGRTLYGFGG  239
            MDVVYALKRQGRTLYGFGG
Sbjct  241  MDVVYALKRQGRTLYGFGG  259
```

>histone H3.1, partial [Galemys pyrenaicus]
Sequence ID: KAG8507280.1 Length: 524
Range 1: 281 to 524

Score:465 bits(1196), Expect:1e-160,
Method:Compositional matrix adjust.,
Identities:234/244(96%), Positives:237/244(97%), Gaps:5/244(2%)

```
Query  1    MARTKQTARKSTGGKAPRKQLATKAARKSAPSTGGVKKPHRYRPGTVALREIRRYQKSTE  60
            MARTKQTARKSTGGKAPRKQLATKAARKSAP+TGGVKKPHRYRPGTVALREIRRYQKSTE
Sbjct  281  MARTKQTARKSTGGKAPRKQLATKAARKSAPATGGVKKPHRYRPGTVALREIRRYQKSTE  340

Query  61   LLIRKLPFQRLVREIAQDFKTDLRFQSAAIGALQEASEAYLVGLFEDTNLCAIHAKRVTI  120
            LLIRKLPFQRLVREIAQDFKTDLRFQS+A+ ALQEA EAYLVGLFEDTNLCAIHAKRVTI
Sbjct  341  LLIRKLPFQRLVREIAQDFKTDLRFQSSAVMALQEACEAYLVGLFEDTNLCAIHAKRVTI  400

Query  121  MPKDIQLARRIRGERA-----MSGRGKGGKGLGKGGAKRHRKVLRDNIQGITKPAIRRLA  175
            MPKDIQLARRIRGERA     MSGRGKGGKGLGKGGAKRHRKVLRDNIQGITKPAIRRLA
Sbjct  401  MPKDIQLARRIRGERASITVVMSGRGKGGKGLGKGGAKRHRKVLRDNIQGITKPAIRRLA  460

Query  176  RRGGVKRISGLIYEETRGVLKVFLENVIRDAVTYTEHAKRKTVTAMDVVYALKRQGRTLY  235
            RRGGVKRISGLIYEETRGVLKVFLENVIRDAVTYTEHAKRKTVTAMDVVYALKRQGRTLY
Sbjct  461  RRGGVKRISGLIYEETRGVLKVFLENVIRDAVTYTEHAKRKTVTAMDVVYALKRQGRTLY  520

Query  236  GFGG  239
            GFGG
Sbjct  521  GFGG  524
```

>uncharacterized protein LOC114907512 [Monodon monoceros]
Sequence ID: XP_029099086.1 Length: 461
Range 1: 1 to 241

Score:461 bits(1187), Expect:4e-160,
Method:Compositional matrix adjust.,
Identities:233/241(97%), Positives:236/241(97%), Gaps:3/241(1%)

```
Query  1    MARTKQTARKSTGGKAPRKQLATKAARKSAPSTGGVKKPHRYRPGTVALREIRRYQKSTE  60
            MARTKQTARKSTGGKAPRKQLATKAARKSAP+TGGVKKPHRYRPGTVALREIRRYQKSTE
Sbjct  1    MARTKQTARKSTGGKAPRKQLATKAARKSAPATGGVKKPHRYRPGTVALREIRRYQKSTE  60

Query  61   LLIRKLPFQRLVREIAQDFKTDLRFQSAAIGALQEASEAYLVGLFEDTNLCAIHAKRVTI  120
            LLIRKLPFQRLVREIAQDFKTDLRFQS+A+ ALQEA EAYLVGLFEDTNLCAIHAKRVTI
Sbjct  61   LLIRKLPFQRLVREIAQDFKTDLRFQSSAVMALQEACEAYLVGLFEDTNLCAIHAKRVTI  120

Query  121  MPKDIQLARRIRGERA---MSGRGKGGKGLGKGGAKRHRKVLRDNIQGITKPAIRRLARR  177
            MPKDIQLARRIRGERA   MSGRGKGGKGLGKGGAKRHRKVLRDNIQGITKPAIRRLARR
Sbjct  121  MPKDIQLARRIRGERASLVMSGRGKGGKGLGKGGAKRHRKVLRDNIQGITKPAIRRLARR  180
```

```
Query  178  GGVKRISGLIYEETRGVLKVFLENVIRDAVTYTEHAKRKTVTAMDVVYALKRQGRTLYGF  237
            GGVKRISGLIYEETRGVLKVFLENVIRDAVTYTEHAKRKTVTAMDVVYALKRQGRTLYGF
Sbjct  181  GGVKRISGLIYEETRGVLKVFLENVIRDAVTYTEHAKRKTVTAMDVVYALKRQGRTLYGF  240

Query  238  G  238
            G
Sbjct  241  G  241
```

Range 2: 326 to 461

Score:265 bits(678), Expect:4e-83,
Method:Compositional matrix adjust.,
Identities:131/136(96%), Positives:134/136(98%), Gaps:0/136(0%)

```
Query  1    MARTKQTARKSTGGKAPRKQLATKAARKSAPSTGGVKKPHRYRPGTVALREIRRYQKSTE  60
            MARTKQTARKSTGGKAPRKQLATKAARKSAP+TGGVKKPHRYRPGTVALREIRRYQKSTE
Sbjct  326  MARTKQTARKSTGGKAPRKQLATKAARKSAPATGGVKKPHRYRPGTVALREIRRYQKSTE  385

Query  61   LLIRKLPFQRLVREIAQDFKTDLRFQSAAIGALQEASEAYLVGLFEDTNLCAIHAKRVTI  120
            LLIRKLPFQRLVREIAQDFKTDLRFQS+A+ ALQEA EAYLVGLFEDTNLCAIHAKRVTI
Sbjct  386  LLIRKLPFQRLVREIAQDFKTDLRFQSSAVMALQEACEAYLVGLFEDTNLCAIHAKRVTI  445

Query  121  MPKDIQLARRIRGERA  136
            MPKDIQLARRIRGERA
Sbjct  446  MPKDIQLARRIRGERA  461
```

>uncharacterized protein LOC131703110 [Acipenser ruthenus]
Sequence ID: XP_058861958.1 Length: 369
Range 1: 114 to 369

Score:457 bits(1177), Expect:4e-160,
Method:Compositional matrix adjust.,
Identities:234/256(91%), Positives:238/256(92%), Gaps:17/256(6%)

```
Query  1    MARTKQTARKSTGGKAPRKQLATKAARKSAPSTGGVKKPHRYRPGTVALREIRRYQKSTE  60
            MARTKQTARKSTGGKAPRKQLATKAARKSAP+TGGVKKPHRYRPGTVALREIRRYQKSTE
Sbjct  114  MARTKQTARKSTGGKAPRKQLATKAARKSAPATGGVKKPHRYRPGTVALREIRRYQKSTE  173

Query  61   LLIRKLPFQRLVREIAQDFKTDLRFQSAAIGALQEASEAYLVGLFEDTNLCAIHAKRVTI  120
            LLIRKLPFQRLVREIAQDFKTDLRFQS+A+ ALQEASEAYLVGLFEDTNLCAIHAKRVTI
Sbjct  174  LLIRKLPFQRLVREIAQDFKTDLRFQSSAVMALQEASEAYLVGLFEDTNLCAIHAKRVTI  233

Query  121  MPKDIQLARRIRGER-----------------AMSGRGKGGKGLGKGGAKRHRKVLRDNI  163
            MPKDIQLARRIRGER                 +MSGRGKGGKGLGKGGAKRHRKVLRDNI
Sbjct  234  MPKDIQLARRIRGERSLSPRFKYCIIFIIRIYSMSGRGKGGKGLGKGGAKRHRKVLRDNI  293

Query  164  QGITKPAIRRLARRGGVKRISGLIYEETRGVLKVFLENVIRDAVTYTEHAKRKTVTAMDV  223
            QGITKPAIRRLARRGGVKRISGLIYEETRGVLKVFLENVIRDAVTYTEHAKRKTVTAMDV
Sbjct  294  QGITKPAIRRLARRGGVKRISGLIYEETRGVLKVFLENVIRDAVTYTEHAKRKTVTAMDV  353

Query  224  VYALKRQGRTLYGFGG  239
            VYALKRQGRTLYGFGG
Sbjct  354  VYALKRQGRTLYGFGG  369
```

```
Range 2: 1 to 94

Score:187 bits(474), Expect:1e-53,
Method:Compositional matrix adjust.,
Identities:94/94(100%), Positives:94/94(100%), Gaps:0/94(0%)

Query  137  MSGRGKGGKGLGKGGAKRHRKVLRDNIQGITKPAIRRLARRGGVKRISGLIYEETRGVLK  196
            MSGRGKGGKGLGKGGAKRHRKVLRDNIQGITKPAIRRLARRGGVKRISGLIYEETRGVLK
Sbjct  1    MSGRGKGGKGLGKGGAKRHRKVLRDNIQGITKPAIRRLARRGGVKRISGLIYEETRGVLK  60

Query  197  VFLENVIRDAVTYTEHAKRKTVTAMDVVYALKRQ  230
            VFLENVIRDAVTYTEHAKRKTVTAMDVVYALKRQ
Sbjct  61   VFLENVIRDAVTYTEHAKRKTVTAMDVVYALKRQ  94



>unnamed protein product [Heligmosomoides polygyrus]
Sequence ID: VDP36995.1 Length: 383
Range 1: 141 to 383

Score:457 bits(1176), Expect:1e-159,
Method:Compositional matrix adjust.,
Identities:231/243(95%), Positives:234/243(96%), Gaps:4/243(1%)

Query  1    MARTKQTARKSTGGKAPRKQLATKAARKSAPSTGGVKKPHRYRPGTVALREIRRYQKSTE  60
            MARTKQTARKSTGGKAPRKQLATKAARKSAP+TGGVKKPHRYRPGTVALREIRRYQKSTE
Sbjct  141  MARTKQTARKSTGGKAPRKQLATKAARKSAPATGGVKKPHRYRPGTVALREIRRYQKSTE  200

Query  61   LLIRKLPFQRLVREIAQDFKTDLRFQSAAIGALQEASEAYLVGLFEDTNLCAIHAKRVTI  120
            LLIRKLPFQRLVREIAQDFKTDLRFQS+A+ ALQEASEAYLVGLFEDTNLCAIHAKRVTI
Sbjct  201  LLIRKLPFQRLVREIAQDFKTDLRFQSSAVMALQEASEAYLVGLFEDTNLCAIHAKRVTI  260

Query  121  MPKDIQLARRIRGER----AMSGRGKGGKGLGKGGAKRHRKVLRDNIQGITKPAIRRLAR  176
            MPKDIQLARRIR       AMSGRGKGGKGLGKGGAKRHRKVLRDNIQGITKPAIRRLAR
Sbjct  261  MPKDIQLARRIRVLLNRIVAMSGRGKGGKGLGKGGAKRHRKVLRDNIQGITKPAIRRLAR  320

Query  177  RGGVKRISGLIYEETRGVLKVFLENVIRDAVTYTEHAKRKTVTAMDVVYALKRQGRTLYG  236
            RGGVKRISGLIYEETRGVLKVFLENVIRDAVTY EHAKRKTVTAMDVVYALKRQGRTLYG
Sbjct  321  RGGVKRISGLIYEETRGVLKVFLENVIRDAVTYCEHAKRKTVTAMDVVYALKRQGRTLYG  380

Query  237  FGG  239
            FGG
Sbjct  381  FGG  383



>uncharacterized protein LOC129974911 [Argiope bruennichi]
Sequence ID: XP_055943672.1 Length: 463
Range 1: 226 to 463

Score:460 bits(1184), Expect:1e-159,
Method:Compositional matrix adjust.,
Identities:233/239(97%), Positives:236/239(98%), Gaps:1/239(0%)

Query  1    MARTKQTARKSTGGKAPRKQLATKAARKSAPSTGGVKKPHRYRPGTVALREIRRYQKSTE  60
            MARTKQTARKSTGGKAPRKQLATKAARKSAP+TGGVKKPHRYRPGTVALREIRRYQKSTE
Sbjct  226  MARTKQTARKSTGGKAPRKQLATKAARKSAPATGGVKKPHRYRPGTVALREIRRYQKSTE  285

Query  61   LLIRKLPFQRLVREIAQDFKTDLRFQSAAIGALQEASEAYLVGLFEDTNLCAIHAKRVTI  120
            LLIRKLPFQRLVREIAQDFKTDLRFQS+A+ ALQEASEAYLVGLFEDTNLCAIHAKRVTI
Sbjct  286  LLIRKLPFQRLVREIAQDFKTDLRFQSSAVMALQEASEAYLVGLFEDTNLCAIHAKRVTI  345
```

```
Query  121  MPKDIQLARRIRGERAMSGRGKGGKGLGKGGAKRHRKVLRDNIQGITKPAIRRLARRGGV  180
            MPKDIQLARRIRGE  MSGRGKGGKGLGKGGAKRHRKVLRDNIQGITKPAIRRLARRGGV
Sbjct  346  MPKDIQLARRIRGE-LMSGRGKGGKGLGKGGAKRHRKVLRDNIQGITKPAIRRLARRGGV  404

Query  181  KRISGLIYEETRGVLKVFLENVIRDAVTYTEHAKRKTVTAMDVVYALKRQGRTLYGFGG  239
            KRISGLIYEETRGVLKVFLENVIRDAVTYTEHAKRKTVTAMDVVYALKRQGRTLYGFGG
Sbjct  405  KRISGLIYEETRGVLKVFLENVIRDAVTYTEHAKRKTVTAMDVVYALKRQGRTLYGFGG  463
```

Range 2: 1 to 103

Score:206 bits(525), Expect:3e-60,
Method:Compositional matrix adjust.,
Identities:103/103(100%), Positives:103/103(100%), Gaps:0/103(0%)

```
Query  137  MSGRGKGGKGLGKGGAKRHRKVLRDNIQGITKPAIRRLARRGGVKRISGLIYEETRGVLK  196
            MSGRGKGGKGLGKGGAKRHRKVLRDNIQGITKPAIRRLARRGGVKRISGLIYEETRGVLK
Sbjct  1    MSGRGKGGKGLGKGGAKRHRKVLRDNIQGITKPAIRRLARRGGVKRISGLIYEETRGVLK  60

Query  197  VFLENVIRDAVTYTEHAKRKTVTAMDVVYALKRQGRTLYGFGG  239
            VFLENVIRDAVTYTEHAKRKTVTAMDVVYALKRQGRTLYGFGG
Sbjct  61   VFLENVIRDAVTYTEHAKRKTVTAMDVVYALKRQGRTLYGFGG  103
```

>uncharacterized protein LOC134684449 [Mytilus trossulus]
Sequence ID: XP_063399802.1 Length: 478
Range 1: 239 to 478

Score:461 bits(1186), Expect:1e-159,
Method:Compositional matrix adjust.,
Identities:233/240(97%), Positives:236/240(98%), Gaps:1/240(0%)

```
Query  1    MARTKQTARKSTGGKAPRKQLATKAARKSAPSTGGVKKPHRYRPGTVALREIRRYQKSTE  60
            MARTKQTARKSTGGKAPRKQLATKAARKSAP+TGGVKKPHRYRPGTVALREIRRYQKSTE
Sbjct  239  MARTKQTARKSTGGKAPRKQLATKAARKSAPATGGVKKPHRYRPGTVALREIRRYQKSTE  298

Query  61   LLIRKLPFQRLVREIAQDFKTDLRFQSAAIGALQEASEAYLVGLFEDTNLCAIHAKRVTI  120
            LLIRKLPFQRLVREIAQDFKTDLRFQS+A+ ALQEASEAYLVGLFEDTNLCAIHAKRVTI
Sbjct  299  LLIRKLPFQRLVREIAQDFKTDLRFQSSAVMALQEASEAYLVGLFEDTNLCAIHAKRVTI  358

Query  121  MPKDIQLARRIRGE-RAMSGRGKGGKGLGKGGAKRHRKVLRDNIQGITKPAIRRLARRGG  179
            MPKDIQLARRIRGE   MSGRGKGGKGLGKGGAKRHRKVLRDNIQGITKPAIRRLARRGG
Sbjct  359  MPKDIQLARRIRGEPNNMSGRGKGGKGLGKGGAKRHRKVLRDNIQGITKPAIRRLARRGG  418

Query  180  VKRISGLIYEETRGVLKVFLENVIRDAVTYTEHAKRKTVTAMDVVYALKRQGRTLYGFGG  239
            VKRISGLIYEETRGVLKVFLENVIRDAVTYTEHAKRKTVTAMDVVYALKRQGRTLYGFGG
Sbjct  419  VKRISGLIYEETRGVLKVFLENVIRDAVTYTEHAKRKTVTAMDVVYALKRQGRTLYGFGG  478
```

Range 2: 1 to 103

Score:206 bits(524), Expect:6e-60,
Method:Compositional matrix adjust.,
Identities:103/103(100%), Positives:103/103(100%), Gaps:0/103(0%)

```
Query  137  MSGRGKGGKGLGKGGAKRHRKVLRDNIQGITKPAIRRLARRGGVKRISGLIYEETRGVLK  196
```

```
              MSGRGKGGKGLGKGGAKRHRKVLRDNIQGITKPAIRRLARRGGVKRISGLIYEETRGVLK
Sbjct  1      MSGRGKGGKGLGKGGAKRHRKVLRDNIQGITKPAIRRLARRGGVKRISGLIYEETRGVLK  60

Query  197    VFLENVIRDAVTYTEHAKRKTVTAMDVVYALKRQGRTLYGFGG  239
              VFLENVIRDAVTYTEHAKRKTVTAMDVVYALKRQGRTLYGFGG
Sbjct  61     VFLENVIRDAVTYTEHAKRKTVTAMDVVYALKRQGRTLYGFGG  103
```

>uncharacterized protein LOC130053805 [Ostrea edulis]
Sequence ID: XP_056017360.1 Length: 689
Range 1: 449 to 689

Score:468 bits(1205), Expect:1e-159,
Method:Compositional matrix adjust.,
Identities:234/241(97%), Positives:238/241(98%), Gaps:2/241(0%)

```
Query  1      MARTKQTARKSTGGKAPRKQLATKAARKSAPSTGGVKKPHRYRPGTVALREIRRYQKSTE  60
              MARTKQTARKSTGGKAPRKQLATKAARKSAP+TGGVKKPHRYRPGTVALREIRRYQKSTE
Sbjct  449    MARTKQTARKSTGGKAPRKQLATKAARKSAPATGGVKKPHRYRPGTVALREIRRYQKSTE  508

Query  61     LLIRKLPFQRLVREIAQDFKTDLRFQSAAIGALQEASEAYLVGLFEDTNLCAIHAKRVTI  120
              LLIRKLPFQRLVREIAQDFKTDLRFQS+A+ ALQEASEAYLVGLFEDTNLCAIHAKRVTI
Sbjct  509    LLIRKLPFQRLVREIAQDFKTDLRFQSSAVMALQEASEAYLVGLFEDTNLCAIHAKRVTI  568

Query  121    MPKDIQLARRIRGERA--MSGRGKGGKGLGKGGAKRHRKVLRDNIQGITKPAIRRLARRG  178
              MPKDIQLARRIRGER+  MSGRGKGGKGLGKGGAKRHRKVLRDNIQGITKPAIRRLARRG
Sbjct  569    MPKDIQLARRIRGERSAIMSGRGKGGKGLGKGGAKRHRKVLRDNIQGITKPAIRRLARRG  628

Query  179    GVKRISGLIYEETRGVLKVFLENVIRDAVTYTEHAKRKTVTAMDVVYALKRQGRTLYGFG  238
              GVKRISGLIYEETRGVLKVFLENVIRDAVTYTEHAKRKTVTAMDVVYALKRQGRTLYGFG
Sbjct  629    GVKRISGLIYEETRGVLKVFLENVIRDAVTYTEHAKRKTVTAMDVVYALKRQGRTLYGFG  688

Query  239    G  239
              G
Sbjct  689    G  689
```

Range 2: 1 to 102

Score:207 bits(527), Expect:1e-58,
Method:Compositional matrix adjust.,
Identities:102/102(100%), Positives:102/102(100%), Gaps:0/102(0%)

```
Query  137    MSGRGKGGKGLGKGGAKRHRKVLRDNIQGITKPAIRRLARRGGVKRISGLIYEETRGVLK  196
              MSGRGKGGKGLGKGGAKRHRKVLRDNIQGITKPAIRRLARRGGVKRISGLIYEETRGVLK
Sbjct  1      MSGRGKGGKGLGKGGAKRHRKVLRDNIQGITKPAIRRLARRGGVKRISGLIYEETRGVLK  60

Query  197    VFLENVIRDAVTYTEHAKRKTVTAMDVVYALKRQGRTLYGFG  238
              VFLENVIRDAVTYTEHAKRKTVTAMDVVYALKRQGRTLYGFG
Sbjct  61     VFLENVIRDAVTYTEHAKRKTVTAMDVVYALKRQGRTLYGFG  102
```

>uncharacterized protein LOC129956849 [Argiope bruennichi]
Sequence ID: XP_055924781.1 Length: 444
Range 1: 192 to 444

Score:458 bits(1179), Expect:3e-159,

```
Method:Compositional matrix adjust.,
Identities:234/253(92%), Positives:237/253(93%), Gaps:14/253(5%)

Query  1    MARTKQTARKSTGGKAPRKQLATKAARKSAPSTGGVKKPHRYRPGTVALREIRRYQKSTE  60
            MARTKQTARKSTGGKAPRKQLATKAARKSAP+TGGVKKPHRYRPGTVALREIRRYQKSTE
Sbjct  192  MARTKQTARKSTGGKAPRKQLATKAARKSAPATGGVKKPHRYRPGTVALREIRRYQKSTE  251

Query  61   LLIRKLPFQRLVREIAQDFKTDLRFQSAAIGALQEASEAYLVGLFEDTNLCAIHAKRVTI  120
            LLIRKLPFQRLVREIAQDFKTDLRFQS+A+ ALQEASEAYLVGLFEDTNLCAIHAKRVTI
Sbjct  252  LLIRKLPFQRLVREIAQDFKTDLRFQSSAVMALQEASEAYLVGLFEDTNLCAIHAKRVTI  311

Query  121  MPKDIQLARRIRGERA--------------MSGRGKGGKGLGKGGAKRHRKVLRDNIQGI  166
            MPKDIQLARRIRGER               MSGRGKGGKGLGKGGAKRHRKVLRDNIQGI
Sbjct  312  MPKDIQLARRIRGERCSPIQDMHKLVLDAIMSGRGKGGKGLGKGGAKRHRKVLRDNIQGI  371

Query  167  TKPAIRRLARRGGVKRISGLIYEETRGVLKVFLENVIRDAVTYTEHAKRKTVTAMDVVYA  226
            TKPAIRRLARRGGVKRISGLIYEETRGVLKVFLENVIRDAVTYTEHAKRKTVTAMDVVYA
Sbjct  372  TKPAIRRLARRGGVKRISGLIYEETRGVLKVFLENVIRDAVTYTEHAKRKTVTAMDVVYA  431

Query  227  LKRQGRTLYGFGG  239
            LKRQGRTLYGFGG
Sbjct  432  LKRQGRTLYGFGG  444


Range 2: 1 to 102

Score:204 bits(519), Expect:2e-59,
Method:Compositional matrix adjust.,
Identities:102/102(100%), Positives:102/102(100%), Gaps:0/102(0%)

Query  137  MSGRGKGGKGLGKGGAKRHRKVLRDNIQGITKPAIRRLARRGGVKRISGLIYEETRGVLK  196
            MSGRGKGGKGLGKGGAKRHRKVLRDNIQGITKPAIRRLARRGGVKRISGLIYEETRGVLK
Sbjct  1    MSGRGKGGKGLGKGGAKRHRKVLRDNIQGITKPAIRRLARRGGVKRISGLIYEETRGVLK  60

Query  197  VFLENVIRDAVTYTEHAKRKTVTAMDVVYALKRQGRTLYGFG  238
            VFLENVIRDAVTYTEHAKRKTVTAMDVVYALKRQGRTLYGFG
Sbjct  61   VFLENVIRDAVTYTEHAKRKTVTAMDVVYALKRQGRTLYGFG  102


>histone H3.1-like [Pongo pygmaeus]
Sequence ID: XP_054346924.1 Length: 269
Range 1: 1 to 269

Score:451 bits(1161), Expect:4e-159,
Method:Compositional matrix adjust.,
Identities:234/269(87%), Positives:237/269(88%), Gaps:30/269(11%)

Query  1    MARTKQTARKSTGGKAPRKQLATKAARKSAPSTGGVKKPHRYRPGTVALREIRRYQKSTE  60
            MARTKQTARKSTGGKAPRKQLATKAARKSAP+TGGVKKPHRYRPGTVALREIRRYQKSTE
Sbjct  1    MARTKQTARKSTGGKAPRKQLATKAARKSAPATGGVKKPHRYRPGTVALREIRRYQKSTE  60

Query  61   LLIRKLPFQRLVREIAQDFKTDLRFQSAAIGALQEASEAYLVGLFEDTNLCAIHAKRVTI  120
            LLIRKLPFQRLVREIAQDFKTDLRFQS+A+ ALQEA EAYLVGLFEDTNLCAIHAKRVTI
Sbjct  61   LLIRKLPFQRLVREIAQDFKTDLRFQSSAVMALQEACEAYLVGLFEDTNLCAIHAKRVTI  120

Query  121  MPKDIQLARRIRGER------------------------------AMSGRGKGGKGLGKG  150
            MPKDIQLARRIRGER                              AMSGRGKGGKGLGKG
Sbjct  121  MPKDIQLARRIRGERAFGGWGLSTGRKFIGMGILYVNKRNKGVGVAMSGRGKGGKGLGKG  180
```

```
Query  151  GAKRHRKVLRDNIQGITKPAIRRLARRGGVKRISGLIYEETRGVLKVFLENVIRDAVTYT  210
            GAKRHRKVLRDNIQGITKPAIRRLARRGGVKRISGLIYEETRGVLKVFLENVIRDAVTYT
Sbjct  181  GAKRHRKVLRDNIQGITKPAIRRLARRGGVKRISGLIYEETRGVLKVFLENVIRDAVTYT  240

Query  211  EHAKRKTVTAMDVVYALKRQGRTLYGFGG  239
            EHAKRKTVTAMDVVYALKRQGRTLYGFGG
Sbjct  241  EHAKRKTVTAMDVVYALKRQGRTLYGFGG  269
```

>uncharacterized protein LOC114233576 [Eptesicus fuscus]
Sequence ID: XP_054568212.1 Length: 553
Range 1: 310 to 553

Score:462 bits(1188), Expect:5e-159,
Method:Compositional matrix adjust.,
Identities:234/244(96%), Positives:237/244(97%), Gaps:5/244(2%)

```
Query  1    MARTKQTARKSTGGKAPRKQLATKAARKSAPSTGGVKKPHRYRPGTVALREIRRYQKSTE  60
            MARTKQTARKSTGGKAPRKQLATKAARKSAP+TGGVKKPHRYRPGTVALREIRRYQKSTE
Sbjct  310  MARTKQTARKSTGGKAPRKQLATKAARKSAPATGGVKKPHRYRPGTVALREIRRYQKSTE  369

Query  61   LLIRKLPFQRLVREIAQDFKTDLRFQSAAIGALQEASEAYLVGLFEDTNLCAIHAKRVTI  120
            LLIRKLPFQRLVREIAQDFKTDLRFQS+A+ ALQEA EAYLVGLFEDTNLCAIHAKRVTI
Sbjct  370  LLIRKLPFQRLVREIAQDFKTDLRFQSSAVMALQEACEAYLVGLFEDTNLCAIHAKRVTI  429

Query  121  MPKDIQLARRIRGERA-----MSGRGKGGKGLGKGGAKRHRKVLRDNIQGITKPAIRRLA  175
            MPKDIQLARRIRGERA     MSGRGKGGKGLGKGGAKRHRKVLRDNIQGITKPAIRRLA
Sbjct  430  MPKDIQLARRIRGERAVRSATMSGRGKGGKGLGKGGAKRHRKVLRDNIQGITKPAIRRLA  489

Query  176  RRGGVKRISGLIYEETRGVLKVFLENVIRDAVTYTEHAKRKTVTAMDVVYALKRQGRTLY  235
            RRGGVKRISGLIYEETRGVLKVFLENVIRDAVTYTEHAKRKTVTAMDVVYALKRQGRTLY
Sbjct  490  RRGGVKRISGLIYEETRGVLKVFLENVIRDAVTYTEHAKRKTVTAMDVVYALKRQGRTLY  549

Query  236  GFGG  239
            GFGG
Sbjct  550  GFGG  553
```

Range 2: 113 to 247

Score:263 bits(673), Expect:2e-81,
Method:Compositional matrix adjust.,
Identities:130/135(96%), Positives:133/135(98%), Gaps:0/135(0%)

```
Query  1    MARTKQTARKSTGGKAPRKQLATKAARKSAPSTGGVKKPHRYRPGTVALREIRRYQKSTE  60
            MARTKQTARKSTGGKAPRKQLATKAARKSAP+TGGVKKPHRYRPGTVALREIRRYQKSTE
Sbjct  113  MARTKQTARKSTGGKAPRKQLATKAARKSAPATGGVKKPHRYRPGTVALREIRRYQKSTE  172

Query  61   LLIRKLPFQRLVREIAQDFKTDLRFQSAAIGALQEASEAYLVGLFEDTNLCAIHAKRVTI  120
            LLIRKLPFQRLVREIAQDFKTDLRFQS+A+ ALQEA EAYLVGLFEDTNLCAIHAKRVTI
Sbjct  173  LLIRKLPFQRLVREIAQDFKTDLRFQSSAVMALQEACEAYLVGLFEDTNLCAIHAKRVTI  232

Query  121  MPKDIQLARRIRGER  135
            MPKDIQLARRIRGER
Sbjct  233  MPKDIQLARRIRGER  247
```

```
Range 3: 1 to 96

Score:191 bits(486), Expect:1e-53,
Method:Compositional matrix adjust.,
Identities:96/96(100%), Positives:96/96(100%), Gaps:0/96(0%)

Query  137  MSGRGKGGKGLGKGGAKRHRKVLRDNIQGITKPAIRRLARRGGVKRISGLIYEETRGVLK  196
            MSGRGKGGKGLGKGGAKRHRKVLRDNIQGITKPAIRRLARRGGVKRISGLIYEETRGVLK
Sbjct  1    MSGRGKGGKGLGKGGAKRHRKVLRDNIQGITKPAIRRLARRGGVKRISGLIYEETRGVLK  60

Query  197  VFLENVIRDAVTYTEHAKRKTVTAMDVVYALKRQGR  232
            VFLENVIRDAVTYTEHAKRKTVTAMDVVYALKRQGR
Sbjct  61   VFLENVIRDAVTYTEHAKRKTVTAMDVVYALKRQGR  96
```

```
>uncharacterized protein LOC134722516 [Mytilus trossulus]
Sequence ID: XP_063442207.1 Length: 501
Range 1: 1 to 235

Score:459 bits(1181), Expect:1e-158,
Method:Compositional matrix adjust.,
Identities:231/239(97%), Positives:234/239(97%), Gaps:4/239(1%)

Query  1    MARTKQTARKSTGGKAPRKQLATKAARKSAPSTGGVKKPHRYRPGTVALREIRRYQKSTE  60
            MARTKQTARKSTGGKAPRKQLATKAARKSAP+TGGVKKPHRYRPGTVALREIRRYQKSTE
Sbjct  1    MARTKQTARKSTGGKAPRKQLATKAARKSAPATGGVKKPHRYRPGTVALREIRRYQKSTE  60

Query  61   LLIRKLPFQRLVREIAQDFKTDLRFQSAAIGALQEASEAYLVGLFEDTNLCAIHAKRVTI  120
            LLIRKLPFQRLVREIAQDFKTDLRFQS+A+ ALQEASEAYLVGLFEDTNLCAIHAKRVTI
Sbjct  61   LLIRKLPFQRLVREIAQDFKTDLRFQSSAVMALQEASEAYLVGLFEDTNLCAIHAKRVTI  120

Query  121  MPKDIQLARRIRGERAMSGRGKGGKGLGKGGAKRHRKVLRDNIQGITKPAIRRLARRGGV  180
            MPKDIQLARRIRGER    RGKGGKGLGKGGAKRHRKVLRDNIQGITKPAIRRLARRGGV
Sbjct  121  MPKDIQLARRIRGER----RGKGGKGLGKGGAKRHRKVLRDNIQGITKPAIRRLARRGGV  176

Query  181  KRISGLIYEETRGVLKVFLENVIRDAVTYTEHAKRKTVTAMDVVYALKRQGRTLYGFGG  239
            KRISGLIYEETRGVLKVFLENVIRDAVTYTEHAKRKTVTAMDVVYALKRQGRTLYGFGG
Sbjct  177  KRISGLIYEETRGVLKVFLENVIRDAVTYTEHAKRKTVTAMDVVYALKRQGRTLYGFGG  235
```

```
Range 2: 366 to 501

Score:268 bits(685), Expect:9e-84,
Method:Compositional matrix adjust.,
Identities:132/136(97%), Positives:135/136(99%), Gaps:0/136(0%)

Query  1    MARTKQTARKSTGGKAPRKQLATKAARKSAPSTGGVKKPHRYRPGTVALREIRRYQKSTE  60
            MARTKQTARKSTGGKAPRKQLATKAARKSAP+TGGVKKPHRYRPGTVALREIRRYQKSTE
Sbjct  366  MARTKQTARKSTGGKAPRKQLATKAARKSAPATGGVKKPHRYRPGTVALREIRRYQKSTE  425

Query  61   LLIRKLPFQRLVREIAQDFKTDLRFQSAAIGALQEASEAYLVGLFEDTNLCAIHAKRVTI  120
            LLIRKLPFQRLVREIAQDFKTDLRFQS+A+ ALQEASEAYLVGLFEDTNLCAIHAKRVTI
Sbjct  426  LLIRKLPFQRLVREIAQDFKTDLRFQSSAVMALQEASEAYLVGLFEDTNLCAIHAKRVTI  485

Query  121  MPKDIQLARRIRGERA  136
            MPKDIQLARRIRGERA
Sbjct  486  MPKDIQLARRIRGERA  501
```

```
>uncharacterized protein zgc:163040 [Brienomyrus brachyistius]
Sequence ID: XP_048842249.1 Length: 579
Range 1: 328 to 579

Score:462 bits(1188), Expect:1e-158,
Method:Compositional matrix adjust.,
Identities:234/252(93%), Positives:237/252(94%), Gaps:13/252(5%)

Query  1    MARTKQTARKSTGGKAPRKQLATKAARKSAPSTGGVKKPHRYRPGTVALREIRRYQKSTE  60
            MARTKQTARKSTGGKAPRKQLATKAARKSAP+TGGVKKPHRYRPGTVALREIRRYQKSTE
Sbjct  328  MARTKQTARKSTGGKAPRKQLATKAARKSAPATGGVKKPHRYRPGTVALREIRRYQKSTE  387

Query  61   LLIRKLPFQRLVREIAQDFKTDLRFQSAAIGALQEASEAYLVGLFEDTNLCAIHAKRVTI  120
            LLIRKLPFQRLVREIAQDFKTDLRFQS+A+ ALQEASEAYLVGLFEDTNLCAIHAKRVTI
Sbjct  388  LLIRKLPFQRLVREIAQDFKTDLRFQSSAVMALQEASEAYLVGLFEDTNLCAIHAKRVTI  447

Query  121  MPKDIQLARRIRGER-------------AMSGRGKGGKGLGKGGAKRHRKVLRDNIQGIT  167
            MPKDIQLARRIRGER              MSGRGKGGKGLGKGGAKRHRKVLRDNIQGIT
Sbjct  448  MPKDIQLARRIRGERERDLSIFVSNSEVVMSGRGKGGKGLGKGGAKRHRKVLRDNIQGIT  507

Query  168  KPAIRRLARRGGVKRISGLIYEETRGVLKVFLENVIRDAVTYTEHAKRKTVTAMDVVYAL  227
            KPAIRRLARRGGVKRISGLIYEETRGVLKVFLENVIRDAVTYTEHAKRKTVTAMDVVYAL
Sbjct  508  KPAIRRLARRGGVKRISGLIYEETRGVLKVFLENVIRDAVTYTEHAKRKTVTAMDVVYAL  567

Query  228  KRQGRTLYGFGG  239
            KRQGRTLYGFGG
Sbjct  568  KRQGRTLYGFGG  579



Range 2: 1 to 102

Score:206 bits(523), Expect:7e-59,
Method:Compositional matrix adjust.,
Identities:102/102(100%), Positives:102/102(100%), Gaps:0/102(0%)

Query  137  MSGRGKGGKGLGKGGAKRHRKVLRDNIQGITKPAIRRLARRGGVKRISGLIYEETRGVLK  196
            MSGRGKGGKGLGKGGAKRHRKVLRDNIQGITKPAIRRLARRGGVKRISGLIYEETRGVLK
Sbjct  1    MSGRGKGGKGLGKGGAKRHRKVLRDNIQGITKPAIRRLARRGGVKRISGLIYEETRGVLK  60

Query  197  VFLENVIRDAVTYTEHAKRKTVTAMDVVYALKRQGRTLYGFG  238
            VFLENVIRDAVTYTEHAKRKTVTAMDVVYALKRQGRTLYGFG
Sbjct  61   VFLENVIRDAVTYTEHAKRKTVTAMDVVYALKRQGRTLYGFG  102



>hypothetical protein Btru_026001 [Bulinus truncatus]
Sequence ID: KAH9492501.1 Length: 382
Range 1: 131 to 382

Score:454 bits(1168), Expect:2e-158,
Method:Compositional matrix adjust.,
Identities:232/252(92%), Positives:235/252(93%), Gaps:13/252(5%)

Query  1    MARTKQTARKSTGGKAPRKQLATKAARKSAPSTGGVKKPHRYRPGTVALREIRRYQKSTE  60
            MARTKQTARKSTGGKAPRKQLATKAARKSAP+TGGVKKPHRYRPGTVALREIRRYQKSTE
Sbjct  131  MARTKQTARKSTGGKAPRKQLATKAARKSAPATGGVKKPHRYRPGTVALREIRRYQKSTE  190

Query  61   LLIRKLPFQRLVREIAQDFKTDLRFQSAAIGALQEASEAYLVGLFEDTNLCAIHAKRVTI  120
```

```
           LLIRKLPFQRLVREIAQDFKTDLRFQS+A+ ALQEASEAYLVGLFEDTNLCAIHAKRVTI
Sbjct  191  LLIRKLPFQRLVREIAQDFKTDLRFQSSAVMALQEASEAYLVGLFEDTNLCAIHAKRVTI  250

Query  121  MPKDIQLARRIRG-------------ERAMSGRGKGGKGLGKGGAKRHRKVLRDNIQGIT  167
            MPKDIQLARRIRG                MSGRGKGGKGLGKGGAKRHRKVLRDNIQGIT
Sbjct  251  MPKDIQLARRIRGGFGRQYSFFQQKFAATMSGRGKGGKGLGKGGAKRHRKVLRDNIQGIT  310

Query  168  KPAIRRLARRGGVKRISGLIYEETRGVLKVFLENVIRDAVTYTEHAKRKTVTAMDVVYAL  227
            KPAIRRLARRGGVKRISGLIYEETRGVLKVFLENVIRDAVTYTEHAKRKTVTAMDVVYAL
Sbjct  311  KPAIRRLARRGGVKRISGLIYEETRGVLKVFLENVIRDAVTYTEHAKRKTVTAMDVVYAL  370

Query  228  KRQGRTLYGFGG  239
            KRQGRTLYGFGG
Sbjct  371  KRQGRTLYGFGG  382
```

>LOW QUALITY PROTEIN: uncharacterized protein LOC116544226 [Sapajus apella]
Sequence ID: XP_032126308.1 Length: 428
Range 1: 1 to 255

Score:456 bits(1172), Expect:2e-158,
Method:Compositional matrix adjust.,
Identities:232/255(91%), Positives:235/255(92%), Gaps:18/255(7%)

```
Query  1    MARTKQTARKSTGGKAPRKQLATKAARKSAPSTGGVKKPHRYRPGTVALREIRRYQKSTE  60
            MARTKQTARKSTGGKAPRKQLATKAARKSAP+TGGVKKPHRYRPGTVALREIRRYQKSTE
Sbjct  1    MARTKQTARKSTGGKAPRKQLATKAARKSAPATGGVKKPHRYRPGTVALREIRRYQKSTE  60

Query  61   LLIRKLPFQRLVREIAQDFKTDLRFQSAAIGALQEASEAYLVGLFEDTNLCAIHAKRVTI  120
            LLIRKLPFQRLVREIAQDFKTDLRFQS+A+ ALQEA EAYLVGLFEDTNLCAIHAKRVTI
Sbjct  61   LLIRKLPFQRLVREIAQDFKTDLRFQSSAVMALQEACEAYLVGLFEDTNLCAIHAKRVTI  120

Query  121  MPKDIQLARRIRGERA------------------MSGRGKGGKGLGKGGAKRHRKVLRDN  162
            MPKDIQLARRIRGERA                  MSGRGKGGKGLGKGGAKRHRKVLRDN
Sbjct  121  MPKDIQLARRIRGERAXSPARAWGRFLAFSLERSMSGRGKGGKGLGKGGAKRHRKVLRDN  180

Query  163  IQGITKPAIRRLARRGGVKRISGLIYEETRGVLKVFLENVIRDAVTYTEHAKRKTVTAMD  222
            IQGITKPAIRRLARRGGVKRISGLIYEETRGVLKVFLENVIRDAVTYTEHAKRKTVTAMD
Sbjct  181  IQGITKPAIRRLARRGGVKRISGLIYEETRGVLKVFLENVIRDAVTYTEHAKRKTVTAMD  240

Query  223  VVYALKRQGRTLYGF  237
            VVYALKRQGRTLYGF
Sbjct  241  VVYALKRQGRTLYGF  255
```

Range 2: 293 to 428

Score:266 bits(680), Expect:7e-84,
Method:Compositional matrix adjust.,
Identities:131/136(96%), Positives:134/136(98%), Gaps:0/136(0%)

```
Query  1    MARTKQTARKSTGGKAPRKQLATKAARKSAPSTGGVKKPHRYRPGTVALREIRRYQKSTE  60
            MARTKQTARKSTGGKAPRKQLATKAARKSAP+TGGVKKPHRYRPGTVALREIRRYQKSTE
Sbjct  293  MARTKQTARKSTGGKAPRKQLATKAARKSAPATGGVKKPHRYRPGTVALREIRRYQKSTE  352

Query  61   LLIRKLPFQRLVREIAQDFKTDLRFQSAAIGALQEASEAYLVGLFEDTNLCAIHAKRVTI  120
            LLIRKLPFQRLVREIAQDFKTDLRFQS+A+ ALQEA EAYLVGLFEDTNLCAIHAKRVTI
Sbjct  353  LLIRKLPFQRLVREIAQDFKTDLRFQSSAVMALQEACEAYLVGLFEDTNLCAIHAKRVTI  412
```

```
Query  121  MPKDIQLARRIRGERA  136
            MPKDIQLARRIRGERA
Sbjct  413  MPKDIQLARRIRGERA  428
```

>uncharacterized protein LOC132221136 [Myotis daubentonii]
Sequence ID: XP_059531074.1 Length: 467
Range 1: 36 to 297

Score:456 bits(1173), Expect:6e-158,
Method:Compositional matrix adjust.,
Identities:234/262(89%), Positives:237/262(90%), Gaps:23/262(8%)

```
Query  1    MARTKQTARKSTGGKAPRKQLATKAARKSAPSTGGVKKPHRYRPGTVALREIRRYQKSTE  60
            MARTKQTARKSTGGKAPRKQLATKAARKSAP+TGGVKKPHRYRPGTVALREIRRYQKSTE
Sbjct  36   MARTKQTARKSTGGKAPRKQLATKAARKSAPATGGVKKPHRYRPGTVALREIRRYQKSTE  95

Query  61   LLIRKLPFQRLVREIAQDFKTDLRFQSAAIGALQEASEAYLVGLFEDTNLCAIHAKRVTI  120
            LLIRKLPFQRLVREIAQDFKTDLRFQS+A+ ALQEA EAYLVGLFEDTNLCAIHAKRVTI
Sbjct  96   LLIRKLPFQRLVREIAQDFKTDLRFQSSAVMALQEACEAYLVGLFEDTNLCAIHAKRVTI  155

Query  121  MPKDIQLARRIRGERA-----------------------MSGRGKGGKGLGKGGAKRHRK  157
            MPKDIQLARRIRGERA                       MSGRGKGGKGLGKGGAKRHRK
Sbjct  156  MPKDIQLARRIRGERACPQQKYISTAREGAGVAFFPTASMSGRGKGGKGLGKGGAKRHRK  215

Query  158  VLRDNIQGITKPAIRRLARRGGVKRISGLIYEETRGVLKVFLENVIRDAVTYTEHAKRKT  217
            VLRDNIQGITKPAIRRLARRGGVKRISGLIYEETRGVLKVFLENVIRDAVTYTEHAKRKT
Sbjct  216  VLRDNIQGITKPAIRRLARRGGVKRISGLIYEETRGVLKVFLENVIRDAVTYTEHAKRKT  275

Query  218  VTAMDVVYALKRQGRTLYGFGG  239
            VTAMDVVYALKRQGRTLYGFGG
Sbjct  276  VTAMDVVYALKRQGRTLYGFGG  297
```

Range 2: 332 to 467

Score:265 bits(677), Expect:8e-83,
Method:Compositional matrix adjust.,
Identities:131/136(96%), Positives:134/136(98%), Gaps:0/136(0%)

```
Query  1    MARTKQTARKSTGGKAPRKQLATKAARKSAPSTGGVKKPHRYRPGTVALREIRRYQKSTE  60
            MARTKQTARKSTGGKAPRKQLATKAARKSAP+TGGVKKPHRYRPGTVALREIRRYQKSTE
Sbjct  332  MARTKQTARKSTGGKAPRKQLATKAARKSAPATGGVKKPHRYRPGTVALREIRRYQKSTE  391

Query  61   LLIRKLPFQRLVREIAQDFKTDLRFQSAAIGALQEASEAYLVGLFEDTNLCAIHAKRVTI  120
            LLIRKLPFQRLVREIAQDFKTDLRFQS+A+ ALQEA EAYLVGLFEDTNLCAIHAKRVTI
Sbjct  392  LLIRKLPFQRLVREIAQDFKTDLRFQSSAVMALQEACEAYLVGLFEDTNLCAIHAKRVTI  451

Query  121  MPKDIQLARRIRGERA  136
            MPKDIQLARRIRGERA
Sbjct  452  MPKDIQLARRIRGERA  467
```

>uncharacterized protein LOC118180062, partial [Stegodyphus dumicola]
Sequence ID: XP_035205101.1 Length: 412
Range 1: 1 to 258

```
Score:454 bits(1167), Expect:7e-158,
Method:Compositional matrix adjust.,
Identities:232/258(90%), Positives:235/258(91%), Gaps:21/258(8%)

Query  1    MARTKQTARKSTGGKAPRKQLATKAARKSAPSTGGVKKPHRYRPGTVALREIRRYQKSTE  60
            MARTKQTARKSTGGKAPRKQLATKAARKSAP+TGGVKKPHRYRPGTVALREIRRYQKSTE
Sbjct  1    MARTKQTARKSTGGKAPRKQLATKAARKSAPATGGVKKPHRYRPGTVALREIRRYQKSTE  60

Query  61   LLIRKLPFQRLVREIAQDFKTDLRFQSAAIGALQEASEAYLVGLFEDTNLCAIHAKRVTI  120
            LLIRKLPFQRLVREIAQDFKTDLRFQS+A+ ALQEASEAYLVGLFEDTNLCAIHAKRVTI
Sbjct  61   LLIRKLPFQRLVREIAQDFKTDLRFQSSAVMALQEASEAYLVGLFEDTNLCAIHAKRVTI  120

Query  121  MPKDIQLARRIRGERA---------------------MSGRGKGGKGLGKGGAKRHRKVL  159
            MPKDIQLARRIRGER                      MSGRGKGGKGLGKGGAKRHRKVL
Sbjct  121  MPKDIQLARRIRGERETCTLLELKKCKLVYCAENKQKMSGRGKGGKGLGKGGAKRHRKVL  180

Query  160  RDNIQGITKPAIRRLARRGGVKRISGLIYEETRGVLKVFLENVIRDAVTYTEHAKRKTVT  219
            RDNIQGITKPAIRRLARRGGVKRISGLIYEETRGVLKVFLENVIRDAVTYTEHAKRKTVT
Sbjct  181  RDNIQGITKPAIRRLARRGGVKRISGLIYEETRGVLKVFLENVIRDAVTYTEHAKRKTVT  240

Query  220  AMDVVYALKRQGRTLYGF  237
            AMDVVYALKRQGRTLYGF
Sbjct  241  AMDVVYALKRQGRTLYGF  258



Range 2: 275 to 409

Score:266 bits(680), Expect:5e-84,
Method:Compositional matrix adjust.,
Identities:131/135(97%), Positives:134/135(99%), Gaps:0/135(0%)

Query  1    MARTKQTARKSTGGKAPRKQLATKAARKSAPSTGGVKKPHRYRPGTVALREIRRYQKSTE  60
            MARTKQTARKSTGGKAPRKQLATKAARKSAP+TGGVKKPHRYRPGTVALREIRRYQKSTE
Sbjct  275  MARTKQTARKSTGGKAPRKQLATKAARKSAPATGGVKKPHRYRPGTVALREIRRYQKSTE  334

Query  61   LLIRKLPFQRLVREIAQDFKTDLRFQSAAIGALQEASEAYLVGLFEDTNLCAIHAKRVTI  120
            LLIRKLPFQRLVREIAQDFKTDLRFQS+A+ ALQEASEAYLVGLFEDTNLCAIHAKRVTI
Sbjct  335  LLIRKLPFQRLVREIAQDFKTDLRFQSSAVMALQEASEAYLVGLFEDTNLCAIHAKRVTI  394

Query  121  MPKDIQLARRIRGER  135
            MPKDIQLARRIRGER
Sbjct  395  MPKDIQLARRIRGER  409



>uncharacterized protein LOC124227097 [Equus quagga]
Sequence ID: XP_046496952.1 Length: 606
Range 1: 363 to 606

Score:460 bits(1184), Expect:1e-157,
Method:Compositional matrix adjust.,
Identities:233/244(95%), Positives:236/244(96%), Gaps:5/244(2%)

Query  1    MARTKQTARKSTGGKAPRKQLATKAARKSAPSTGGVKKPHRYRPGTVALREIRRYQKSTE  60
            MARTKQTARKSTGGKAPRKQLATKAARKSAP+TGGVKKPHRYRPGTVALREIRRYQKSTE
Sbjct  363  MARTKQTARKSTGGKAPRKQLATKAARKSAPATGGVKKPHRYRPGTVALREIRRYQKSTE  422

Query  61   LLIRKLPFQRLVREIAQDFKTDLRFQSAAIGALQEASEAYLVGLFEDTNLCAIHAKRVTI  120
            LLIRKLPFQRLVREIAQDFKTDLRFQS+A+ ALQEA EAYLVGLFEDTNLCAIHAKRVTI
Sbjct  423  LLIRKLPFQRLVREIAQDFKTDLRFQSSAVMALQEACEAYLVGLFEDTNLCAIHAKRVTI  482
```

```
Query  121   MPKDIQLARRIRGER-----AMSGRGKGGKGLGKGGAKRHRKVLRDNIQGITKPAIRRLA  175
             MPKDIQLARRIRGER      MSGRGKGGKGLGKGGAKRHRKVLRDNIQGITKPAIRRLA
Sbjct  483   MPKDIQLARRIRGERQSLVLTMSGRGKGGKGLGKGGAKRHRKVLRDNIQGITKPAIRRLA  542

Query  176   RRGGVKRISGLIYEETRGVLKVFLENVIRDAVTYTEHAKRKTVTAMDVVYALKRQGRTLY  235
             RRGGVKRISGLIYEETRGVLKVFLENVIRDAVTYTEHAKRKTVTAMDVVYALKRQGRTLY
Sbjct  543   RRGGVKRISGLIYEETRGVLKVFLENVIRDAVTYTEHAKRKTVTAMDVVYALKRQGRTLY  602

Query  236   GFGG  239
             GFGG
Sbjct  603   GFGG  606
```

Range 2: 112 to 258

Score:268 bits(684), Expect:2e-82,
Method:Compositional matrix adjust.,
Identities:133/147(90%), Positives:136/147(92%), Gaps:0/147(0%)

```
Query  1     MARTKQTARKSTGGKAPRKQLATKAARKSAPSTGGVKKPHRYRPGTVALREIRRYQKSTE  60
             MARTKQTARKSTGGKAPRKQLATKAARKSAP+TGGVKKPHRYRPGTVALREIRRYQKSTE
Sbjct  112   MARTKQTARKSTGGKAPRKQLATKAARKSAPATGGVKKPHRYRPGTVALREIRRYQKSTE  171

Query  61    LLIRKLPFQRLVREIAQDFKTDLRFQSAAIGALQEASEAYLVGLFEDTNLCAIHAKRVTI  120
             LLIRKLPFQRLVREIAQDFKTDLRFQS+A+ ALQEA EAYLVGLFEDTNLCAIHAKRVTI
Sbjct  172   LLIRKLPFQRLVREIAQDFKTDLRFQSSAVMALQEACEAYLVGLFEDTNLCAIHAKRVTI  231

Query  121   MPKDIQLARRIRGERAMSGRGKGGKGL  147
             MPKDIQLARRIRGER   G  K    L
Sbjct  232   MPKDIQLARRIRGERECHGVAKSSSSL  258
```

Range 3: 1 to 96

Score:191 bits(485), Expect:4e-53,
Method:Compositional matrix adjust.,
Identities:96/96(100%), Positives:96/96(100%), Gaps:0/96(0%)

```
Query  137   MSGRGKGGKGLGKGGAKRHRKVLRDNIQGITKPAIRRLARRGGVKRISGLIYEETRGVLK  196
             MSGRGKGGKGLGKGGAKRHRKVLRDNIQGITKPAIRRLARRGGVKRISGLIYEETRGVLK
Sbjct  1     MSGRGKGGKGLGKGGAKRHRKVLRDNIQGITKPAIRRLARRGGVKRISGLIYEETRGVLK  60

Query  197   VFLENVIRDAVTYTEHAKRKTVTAMDVVYALKRQGR  232
             VFLENVIRDAVTYTEHAKRKTVTAMDVVYALKRQGR
Sbjct  61    VFLENVIRDAVTYTEHAKRKTVTAMDVVYALKRQGR  96
```

>uncharacterized protein LOC123392674 [Mustela putorius furo]
Sequence ID: XP_044938242.1 Length: 470
Range 1: 1 to 262

Score:454 bits(1169), Expect:3e-157,
Method:Compositional matrix adjust.,
Identities:234/262(89%), Positives:237/262(90%), Gaps:23/262(8%)

```
Query  1     MARTKQTARKSTGGKAPRKQLATKAARKSAPSTGGVKKPHRYRPGTVALREIRRYQKSTE  60
```

```
              MARTKQTARKSTGGKAPRKQLATKAARKSAP+TGGVKKPHRYRPGTVALREIRRYQKSTE
Sbjct  1      MARTKQTARKSTGGKAPRKQLATKAARKSAPATGGVKKPHRYRPGTVALREIRRYQKSTE  60

Query  61     LLIRKLPFQRLVREIAQDFKTDLRFQSAAIGALQEASEAYLVGLFEDTNLCAIHAKRVTI  120
              LLIRKLPFQRLVREIAQDFKTDLRFQS+A+ ALQEA EAYLVGLFEDTNLCAIHAKRVTI
Sbjct  61     LLIRKLPFQRLVREIAQDFKTDLRFQSSAVMALQEACEAYLVGLFEDTNLCAIHAKRVTI  120

Query  121    MPKDIQLARRIRGERA-----------------------MSGRGKGGKGLGKGGAKRHRK  157
              MPKDIQLARRIRGERA                       MSGRGKGGKGLGKGGAKRHRK
Sbjct  121    MPKDIQLARRIRGERACPTNDYIGRHPERRAVVPDPFVVMSGRGKGGKGLGKGGAKRHRK  180

Query  158    VLRDNIQGITKPAIRRLARRGGVKRISGLIYEETRGVLKVFLENVIRDAVTYTEHAKRKT  217
              VLRDNIQGITKPAIRRLARRGGVKRISGLIYEETRGVLKVFLENVIRDAVTYTEHAKRKT
Sbjct  181    VLRDNIQGITKPAIRRLARRGGVKRISGLIYEETRGVLKVFLENVIRDAVTYTEHAKRKT  240

Query  218    VTAMDVVYALKRQGRTLYGFGG  239
              VTAMDVVYALKRQGRTLYGFGG
Sbjct  241    VTAMDVVYALKRQGRTLYGFGG  262
```

Range 2: 335 to 470

Score:264 bits(675), Expect:1e-82,
Method:Compositional matrix adjust.,
Identities:131/136(96%), Positives:134/136(98%), Gaps:0/136(0%)

```
Query  1      MARTKQTARKSTGGKAPRKQLATKAARKSAPSTGGVKKPHRYRPGTVALREIRRYQKSTE  60
              MARTKQTARKSTGGKAPRKQLATKAARKSAP+TGGVKKPHRYRPGTVALREIRRYQKSTE
Sbjct  335    MARTKQTARKSTGGKAPRKQLATKAARKSAPATGGVKKPHRYRPGTVALREIRRYQKSTE  394

Query  61     LLIRKLPFQRLVREIAQDFKTDLRFQSAAIGALQEASEAYLVGLFEDTNLCAIHAKRVTI  120
              LLIRKLPFQRLVREIAQDFKTDLRFQS+A+ ALQEA EAYLVGLFEDTNLCAIHAKRVTI
Sbjct  395    LLIRKLPFQRLVREIAQDFKTDLRFQSSAVMALQEACEAYLVGLFEDTNLCAIHAKRVTI  454

Query  121    MPKDIQLARRIRGERA  136
              MPKDIQLARRIRGERA
Sbjct  455    MPKDIQLARRIRGERA  470
```

>hypothetical protein Q8A67_001705 [Cirrhinus molitorella]
Sequence ID: KAK2913306.1 Length: 233
Range 1: 1 to 233

Score:445 bits(1145), Expect:3e-157,
Method:Compositional matrix adjust.,
Identities:226/239(95%), Positives:229/239(95%), Gaps:6/239(2%)

```
Query  1      MARTKQTARKSTGGKAPRKQLATKAARKSAPSTGGVKKPHRYRPGTVALREIRRYQKSTE  60
              MARTKQTARKSTGGKAPRKQLATKAARKSAP+TGGVKKPHRYRPGTVALREIRRYQKSTE
Sbjct  1      MARTKQTARKSTGGKAPRKQLATKAARKSAPATGGVKKPHRYRPGTVALREIRRYQKSTE  60

Query  61     LLIRKLPFQRLVREIAQDFKTDLRFQSAAIGALQEASEAYLVGLFEDTNLCAIHAKRVTI  120
              LLIRKLPFQRLVREIAQDFKTDLRFQS+A+ ALQEASEAYLVGLFEDTNLCAIHAKRVTI
Sbjct  61     LLIRKLPFQRLVREIAQDFKTDLRFQSSAVMALQEASEAYLVGLFEDTNLCAIHAKRVTI  120

Query  121    MPKDIQLARRIRGERAMSGRGKGGKGLGKGGAKRHRKVLRDNIQGITKPAIRRLARRGGV  180
              MPKDIQ A        MSGRGKGGKGLGKGGAKRHRKVLRDNIQGITKPAIRRLARRGGV
Sbjct  121    MPKDIQAAVE------MSGRGKGGKGLGKGGAKRHRKVLRDNIQGITKPAIRRLARRGGV  174
```

```
Query  181  KRISGLIYEETRGVLKVFLENVIRDAVTYTEHAKRKTVTAMDVVYALKRQGRTLYGFGG  239
            KRISGLIYEETRGVLKVFLENVIRDAVTYTEHAKRKTVTAMDVVYALKRQGRTLYGFGG
Sbjct  175  KRISGLIYEETRGVLKVFLENVIRDAVTYTEHAKRKTVTAMDVVYALKRQGRTLYGFGG  233
```

>uncharacterized protein LOC118673331 [Myotis myotis]
Sequence ID: XP_036199852.1 Length: 454
Range 1: 1 to 256

Score:453 bits(1165), Expect:7e-157,
Method:Compositional matrix adjust.,
Identities:232/256(91%), Positives:235/256(91%), Gaps:18/256(7%)

```
Query  1    MARTKQTARKSTGGKAPRKQLATKAARKSAPSTGGVKKPHRYRPGTVALREIRRYQKSTE  60
            MARTKQTARKSTGGKAPRKQLATKAARKSAP+TGGVKKPHRYRPGTVALREIRRYQKSTE
Sbjct  1    MARTKQTARKSTGGKAPRKQLATKAARKSAPATGGVKKPHRYRPGTVALREIRRYQKSTE  60

Query  61   LLIRKLPFQRLVREIAQDFKTDLRFQSAAIGALQEASEAYLVGLFEDTNLCAIHAKRVTI  120
            LLIRKLPFQRLVREIAQDFKTDLRFQS+A+ ALQEA EAYLVGLFEDTNLCAIHAKRVTI
Sbjct  61   LLIRKLPFQRLVREIAQDFKTDLRFQSSAVMALQEACEAYLVGLFEDTNLCAIHAKRVTI  120

Query  121  MPKDIQLARRIRGER------------------AMSGRGKGGKGLGKGGAKRHRKVLRDN  162
            MPKDIQLARRIRGER                   MSGRGKGGKGLGKGGAKRHRKVLRDN
Sbjct  121  MPKDIQLARRIRGERKYISTAREDAGIAFFPTAGMSGRGKGGKGLGKGGAKRHRKVLRDN  180

Query  163  IQGITKPAIRRLARRGGVKRISGLIYEETRGVLKVFLENVIRDAVTYTEHAKRKTVTAMD  222
            IQGITKPAIRRLARRGGVKRISGLIYEETRGVLKVFLENVIRDAVTYTEHAKRKTVTAMD
Sbjct  181  IQGITKPAIRRLARRGGVKRISGLIYEETRGVLKVFLENVIRDAVTYTEHAKRKTVTAMD  240

Query  223  VVYALKRQGRTLYGFG  238
            VVYALKRQGRTLYGFG
Sbjct  241  VVYALKRQGRTLYGFG  256
```

Range 2: 319 to 454

Score:264 bits(675), Expect:8e-83,
Method:Compositional matrix adjust.,
Identities:131/136(96%), Positives:134/136(98%), Gaps:0/136(0%)

```
Query  1    MARTKQTARKSTGGKAPRKQLATKAARKSAPSTGGVKKPHRYRPGTVALREIRRYQKSTE  60
            MARTKQTARKSTGGKAPRKQLATKAARKSAP+TGGVKKPHRYRPGTVALREIRRYQKSTE
Sbjct  319  MARTKQTARKSTGGKAPRKQLATKAARKSAPATGGVKKPHRYRPGTVALREIRRYQKSTE  378

Query  61   LLIRKLPFQRLVREIAQDFKTDLRFQSAAIGALQEASEAYLVGLFEDTNLCAIHAKRVTI  120
            LLIRKLPFQRLVREIAQDFKTDLRFQS+A+ ALQEA EAYLVGLFEDTNLCAIHAKRVTI
Sbjct  379  LLIRKLPFQRLVREIAQDFKTDLRFQSSAVMALQEACEAYLVGLFEDTNLCAIHAKRVTI  438

Query  121  MPKDIQLARRIRGERA  136
            MPKDIQLARRIRGERA
Sbjct  439  MPKDIQLARRIRGERA  454
```

>uncharacterized protein LOC133095661 [Eubalaena glacialis]
Sequence ID: XP_061053392.1 Length: 419
Range 1: 1 to 233

```
Score:451 bits(1160), Expect:1e-156,
Method:Compositional matrix adjust.,
Identities:228/234(97%), Positives:231/234(98%), Gaps:1/234(0%)

Query  1    MARTKQTARKSTGGKAPRKQLATKAARKSAPSTGGVKKPHRYRPGTVALREIRRYQKSTE  60
            MARTKQTARKSTGGKAPRKQLATKAARKSAP+TGGVKKPHRYRPGTVALREIRRYQKSTE
Sbjct  1    MARTKQTARKSTGGKAPRKQLATKAARKSAPATGGVKKPHRYRPGTVALREIRRYQKSTE  60

Query  61   LLIRKLPFQRLVREIAQDFKTDLRFQSAAIGALQEASEAYLVGLFEDTNLCAIHAKRVTI  120
            LLIRKLPFQRLVREIAQDFKTDLRFQS+A+ ALQEA EAYLVGLFEDTNLCAIHAKRVTI
Sbjct  61   LLIRKLPFQRLVREIAQDFKTDLRFQSSAVMALQEACEAYLVGLFEDTNLCAIHAKRVTI  120

Query  121  MPKDIQLARRIRGERAMSGRGKGGKGLGKGGAKRHRKVLRDNIQGITKPAIRRLARRGGV  180
            MPKDIQLARRIRGER MSGRGKGGKGLGKGGAKRHRKVLRDNIQGITKPAIRRLARRGGV
Sbjct  121  MPKDIQLARRIRGER-MSGRGKGGKGLGKGGAKRHRKVLRDNIQGITKPAIRRLARRGGV  179

Query  181  KRISGLIYEETRGVLKVFLENVIRDAVTYTEHAKRKTVTAMDVVYALKRQGRTL  234
            KRISGLIYEETRGVLKVFLENVIRDAVTYTEHAKRKTVTAMDVVYALKRQGRTL
Sbjct  180  KRISGLIYEETRGVLKVFLENVIRDAVTYTEHAKRKTVTAMDVVYALKRQGRTL  233



Range 2: 284 to 419

Score:266 bits(679), Expect:9e-84,
Method:Compositional matrix adjust.,
Identities:131/136(96%), Positives:134/136(98%), Gaps:0/136(0%)

Query  1    MARTKQTARKSTGGKAPRKQLATKAARKSAPSTGGVKKPHRYRPGTVALREIRRYQKSTE  60
            MARTKQTARKSTGGKAPRKQLATKAARKSAP+TGGVKKPHRYRPGTVALREIRRYQKSTE
Sbjct  284  MARTKQTARKSTGGKAPRKQLATKAARKSAPATGGVKKPHRYRPGTVALREIRRYQKSTE  343

Query  61   LLIRKLPFQRLVREIAQDFKTDLRFQSAAIGALQEASEAYLVGLFEDTNLCAIHAKRVTI  120
            LLIRKLPFQRLVREIAQDFKTDLRFQS+A+ ALQEA EAYLVGLFEDTNLCAIHAKRVTI
Sbjct  344  LLIRKLPFQRLVREIAQDFKTDLRFQSSAVMALQEACEAYLVGLFEDTNLCAIHAKRVTI  403

Query  121  MPKDIQLARRIRGERA  136
            MPKDIQLARRIRGERA
Sbjct  404  MPKDIQLARRIRGERA  419



>hypothetical protein EI555_001644, partial [Monodon monoceros]
Sequence ID: TKC35997.1 Length: 586
Range 1: 219 to 469

Score:455 bits(1171), Expect:6e-156,
Method:Compositional matrix adjust.,
Identities:232/251(92%), Positives:235/251(93%), Gaps:12/251(4%)

Query  1    MARTKQTARKSTGGKAPRKQLATKAARKSAPSTGGVKKPHRYRPGTVALREIRRYQKSTE  60
            MARTKQTARKSTGGKAPRKQLATKAARKSAP+TGGVKKPHRYRPGTVALREIRRYQKSTE
Sbjct  219  MARTKQTARKSTGGKAPRKQLATKAARKSAPATGGVKKPHRYRPGTVALREIRRYQKSTE  278

Query  61   LLIRKLPFQRLVREIAQDFKTDLRFQSAAIGALQEASEAYLVGLFEDTNLCAIHAKRVTI  120
            LLIRKLPFQRLVREIAQDFKTDLRFQS+A+ ALQEA EAYLVGLFEDTNLCAIHAKRVTI
Sbjct  279  LLIRKLPFQRLVREIAQDFKTDLRFQSSAVMALQEACEAYLVGLFEDTNLCAIHAKRVTI  338

Query  121  MPKDIQLARRIRGER------------AMSGRGKGGKGLGKGGAKRHRKVLRDNIQGITK  168
            MPKDI LARRIRGER            AMSGRGKGGKGLGKGGAKRHRKVLRDNIQGITK
Sbjct  339  MPKDIHLARRIRGERASLRCGCDRCTFAMSGRGKGGKGLGKGGAKRHRKVLRDNIQGITK  398
```

```
Query  169  PAIRRLARRGGVKRISGLIYEETRGVLKVFLENVIRDAVTYTEHAKRKTVTAMDVVYALK  228
            PAIRRLARRGGVKRISGLIYEETRGVLKVFLENVIRDAVTYTEHAKRKTVTAMDVVYALK
Sbjct  399  PAIRRLARRGGVKRISGLIYEETRGVLKVFLENVIRDAVTYTEHAKRKTVTAMDVVYALK  458

Query  229  RQGRTLYGFGG  239
            RQGRTLYGF G
Sbjct  459  RQGRTLYGFAG  469
```

>uncharacterized protein LOC120606096 [Pteropus medius]
Sequence ID: XP_039723142.1 Length: 427
Range 1: 1 to 234

Score:449 bits(1156), Expect:6e-156,
Method:Compositional matrix adjust.,
Identities:227/239(95%), Positives:230/239(96%), Gaps:5/239(2%)

```
Query  1    MARTKQTARKSTGGKAPRKQLATKAARKSAPSTGGVKKPHRYRPGTVALREIRRYQKSTE  60
            MARTKQTARKSTGGKAPRKQLATKAARKSAP+TGGVKKPHRYRPGTVALREIRRYQKSTE
Sbjct  1    MARTKQTARKSTGGKAPRKQLATKAARKSAPATGGVKKPHRYRPGTVALREIRRYQKSTE  60

Query  61   LLIRKLPFQRLVREIAQDFKTDLRFQSAAIGALQEASEAYLVGLFEDTNLCAIHAKRVTI  120
            LLIRKLPFQRLVREIAQDFKTDLRFQS+A+ ALQEA EAYLVGLFEDTNLCAIHAKRVTI
Sbjct  61   LLIRKLPFQRLVREIAQDFKTDLRFQSSAVMALQEACEAYLVGLFEDTNLCAIHAKRVTI  120

Query  121  MPKDIQLARRIRGERAMSGRGKGGKGLGKGGAKRHRKVLRDNIQGITKPAIRRLARRGGV  180
            MPKDIQLARRIRGERA      GGKGLGKGGAKRHRKVLRDNIQGITKPAIRRLARRGGV
Sbjct  121  MPKDIQLARRIRGERA-----GGGKGLGKGGAKRHRKVLRDNIQGITKPAIRRLARRGGV  175

Query  181  KRISGLIYEETRGVLKVFLENVIRDAVTYTEHAKRKTVTAMDVVYALKRQGRTLYGFGG  239
            KRISGLIYEETRGVLKVFLENVIRDAVTYTEHAKRKTVTAMDVVYALKRQGRTLYGFG
Sbjct  176  KRISGLIYEETRGVLKVFLENVIRDAVTYTEHAKRKTVTAMDVVYALKRQGRTLYGFGA  234
```

Range 2: 292 to 427

Score:265 bits(678), Expect:1e-83,
Method:Compositional matrix adjust.,
Identities:131/136(96%), Positives:134/136(98%), Gaps:0/136(0%)

```
Query  1    MARTKQTARKSTGGKAPRKQLATKAARKSAPSTGGVKKPHRYRPGTVALREIRRYQKSTE  60
            MARTKQTARKSTGGKAPRKQLATKAARKSAP+TGGVKKPHRYRPGTVALREIRRYQKSTE
Sbjct  292  MARTKQTARKSTGGKAPRKQLATKAARKSAPATGGVKKPHRYRPGTVALREIRRYQKSTE  351

Query  61   LLIRKLPFQRLVREIAQDFKTDLRFQSAAIGALQEASEAYLVGLFEDTNLCAIHAKRVTI  120
            LLIRKLPFQRLVREIAQDFKTDLRFQS+A+ ALQEA EAYLVGLFEDTNLCAIHAKRVTI
Sbjct  352  LLIRKLPFQRLVREIAQDFKTDLRFQSSAVMALQEACEAYLVGLFEDTNLCAIHAKRVTI  411

Query  121  MPKDIQLARRIRGERA  136
            MPKDIQLARRIRGERA
Sbjct  412  MPKDIQLARRIRGERA  427
```

>uncharacterized protein LOC122785248 [Solea senegalensis]
Sequence ID: XP_043906908.1 Length: 535
Range 1: 1 to 244

```
Score:453 bits(1165), Expect:9e-156,
Method:Compositional matrix adjust.,
Identities:228/244(93%), Positives:233/244(95%), Gaps:10/244(4%)

Query  1    MARTKQTARKSTGGKAPRKQLATKAARKSAPSTGGVKKPHRYRPGTVALREIRRYQKSTE  60
            MARTKQTARKSTGGKAPRKQLATKAARKSAP+TGGVKKPHRYRPGTVALREIRRYQKSTE
Sbjct  1    MARTKQTARKSTGGKAPRKQLATKAARKSAPATGGVKKPHRYRPGTVALREIRRYQKSTE  60

Query  61   LLIRKLPFQRLVREIAQDFKTDLRFQSAAIGALQEASEAYLVGLFEDTNLCAIHAKRVTI  120
            LLIRKLPFQRLVREIAQDFKTDLRFQS+A+ ALQE+SEAYLVGLFEDTNLCAIHAKRVTI
Sbjct  61   LLIRKLPFQRLVREIAQDFKTDLRFQSSAVMALQESSEAYLVGLFEDTNLCAIHAKRVTI  120

Query  121  MPKDIQLARRIRGERA----------MSGRGKGGKGLGKGGAKRHRKVLRDNIQGITKPA  170
            MPKDIQLARRIRGER+          MSGRGKGGKGLGKGGAKRHRKVLRDNIQGITKPA
Sbjct  121  MPKDIQLARRIRGERSPYLCILQSDKMSGRGKGGKGLGKGGAKRHRKVLRDNIQGITKPA  180

Query  171  IRRLARRGGVKRISGLIYEETRGVLKVFLENVIRDAVTYTEHAKRKTVTAMDVVYALKRQ  230
            IRRLARRGGVKRISGLIYEETRGVLKVFLENVIRDAVTYTEHAKRKTVTAMDVVYALKRQ
Sbjct  181  IRRLARRGGVKRISGLIYEETRGVLKVFLENVIRDAVTYTEHAKRKTVTAMDVVYALKRQ  240

Query  231  GRTL  234
            GRTL
Sbjct  241  GRTL  244



Range 2: 400 to 535

Score:268 bits(686), Expect:2e-83,
Method:Compositional matrix adjust.,
Identities:131/136(96%), Positives:135/136(99%), Gaps:0/136(0%)

Query  1    MARTKQTARKSTGGKAPRKQLATKAARKSAPSTGGVKKPHRYRPGTVALREIRRYQKSTE  60
            MARTKQTARKSTGGKAPRKQLATKAARKSAP+TGGVKKPHRYRPGTVALREIRRYQKSTE
Sbjct  400  MARTKQTARKSTGGKAPRKQLATKAARKSAPATGGVKKPHRYRPGTVALREIRRYQKSTE  459

Query  61   LLIRKLPFQRLVREIAQDFKTDLRFQSAAIGALQEASEAYLVGLFEDTNLCAIHAKRVTI  120
            LLIRKLPFQRLVREIAQDFKTDLRFQS+A+ ALQE+SEAYLVGLFEDTNLCAIHAKRVTI
Sbjct  460  LLIRKLPFQRLVREIAQDFKTDLRFQSSAVMALQESSEAYLVGLFEDTNLCAIHAKRVTI  519

Query  121  MPKDIQLARRIRGERA  136
            MPKDIQLARRIRGERA
Sbjct  520  MPKDIQLARRIRGERA  535



>histone H3.2 [Dermatophagoides pteronyssinus]
Sequence ID: KAH9426658.1 Length: 257
Range 1: 1 to 230

Score:442 bits(1137), Expect:1e-155,
Method:Compositional matrix adjust.,
Identities:225/238(95%), Positives:229/238(96%), Gaps:8/238(3%)

Query  1    MARTKQTARKSTGGKAPRKQLATKAARKSAPSTGGVKKPHRYRPGTVALREIRRYQKSTE  60
            MARTKQTARKSTGGKAPRKQLATKAARKSAP+TGGVKKPHRYRPGTVALREIRRYQKSTE
Sbjct  1    MARTKQTARKSTGGKAPRKQLATKAARKSAPATGGVKKPHRYRPGTVALREIRRYQKSTE  60

Query  61   LLIRKLPFQRLVREIAQDFKTDLRFQSAAIGALQEASEAYLVGLFEDTNLCAIHAKRVTI  120
            LLIRKLPFQRLVREIAQDFKTDLRFQS+A+ ALQE+SEAYLVGLFEDTNLCAIHAKRVTI
```

```
Sbjct  61   LLIRKLPFQRLVREIAQDFKTDLRFQSSAVMALQESSEAYLVGLFEDTNLCAIHAKRVTI  120

Query  121  MPKDIQLARRIRGERAMSGRGKGGKGLGKGGAKRHRKVLRDNIQGITKPAIRRLARRGGV  180
            MPKDIQLARRIRGER        GKGLGKGGAKRHRKVLRDNIQGITKPAIRRLARRGGV
Sbjct  121  MPKDIQLARRIRGER--------GKGLGKGGAKRHRKVLRDNIQGITKPAIRRLARRGGV  172

Query  181  KRISGLIYEETRGVLKVFLENVIRDAVTYTEHAKRKTVTAMDVVYALKRQGRTLYGFG  238
            KRISGLIYEETRGVLKVFLENVIRDAVTYTEHAKRKTVTAMDVVYALKRQGRTLYGFG
Sbjct  173  KRISGLIYEETRGVLKVFLENVIRDAVTYTEHAKRKTVTAMDVVYALKRQGRTLYGFG  230
```

>uncharacterized protein LOC114489542 [Phyllostomus discolor]
Sequence ID: XP_035871632.1 Length: 434
Range 1: 1 to 260

Score:449 bits(1154), Expect:1e-155,
Method:Compositional matrix adjust.,
Identities:231/260(89%), Positives:234/260(90%), Gaps:22/260(8%)

```
Query  1    MARTKQTARKSTGGKAPRKQLATKAARKSAPSTGGVKKPHRYRPGTVALREIRRYQKSTE  60
            MARTKQTARKSTGGKAPRKQLATKAARKSAP+TGGVKKPHRYRPGTVALREIRRYQKSTE
Sbjct  1    MARTKQTARKSTGGKAPRKQLATKAARKSAPATGGVKKPHRYRPGTVALREIRRYQKSTE  60

Query  61   LLIRKLPFQRLVREIAQDFKTDLRFQSAAIGALQEASEAYLVGLFEDTNLCAIHAKRVTI  120
            LLIRKLPFQRLVREIAQDFKTDLRFQS+A+ ALQEA EAYLVGLFEDTNLCAIHAKRVTI
Sbjct  61   LLIRKLPFQRLVREIAQDFKTDLRFQSSAVMALQEACEAYLVGLFEDTNLCAIHAKRVTI  120

Query  121  MPKDIQLARRIRGERA----------------------MSGRGKGGKGLGKGGAKRHRKV  158
            MPKDIQLARRIRGERA                      MSGRGKGGKGLGKGGAKRHRKV
Sbjct  121  MPKDIQLARRIRGERASAEEVYKALRASLQRLVVPSTNMSGRGKGGKGLGKGGAKRHRKV  180

Query  159  LRDNIQGITKPAIRRLARRGGVKRISGLIYEETRGVLKVFLENVIRDAVTYTEHAKRKTV  218
            LRDNIQGITKPAIRRLARRGGVKRISGLIYEETRGVLKVFLENVIRDAVTYTEHAKRKTV
Sbjct  181  LRDNIQGITKPAIRRLARRGGVKRISGLIYEETRGVLKVFLENVIRDAVTYTEHAKRKTV  240

Query  219  TAMDVVYALKRQGRTLYGFG  238
            TAMDVVYALKRQGRTLY  G
Sbjct  241  TAMDVVYALKRQGRTLYEIG  260
```

Range 2: 299 to 434

Score:265 bits(678), Expect:2e-83,
Method:Compositional matrix adjust.,
Identities:131/136(96%), Positives:134/136(98%), Gaps:0/136(0%)

```
Query  1    MARTKQTARKSTGGKAPRKQLATKAARKSAPSTGGVKKPHRYRPGTVALREIRRYQKSTE  60
            MARTKQTARKSTGGKAPRKQLATKAARKSAP+TGGVKKPHRYRPGTVALREIRRYQKSTE
Sbjct  299  MARTKQTARKSTGGKAPRKQLATKAARKSAPATGGVKKPHRYRPGTVALREIRRYQKSTE  358

Query  61   LLIRKLPFQRLVREIAQDFKTDLRFQSAAIGALQEASEAYLVGLFEDTNLCAIHAKRVTI  120
            LLIRKLPFQRLVREIAQDFKTDLRFQS+A+ ALQEA EAYLVGLFEDTNLCAIHAKRVTI
Sbjct  359  LLIRKLPFQRLVREIAQDFKTDLRFQSSAVMALQEACEAYLVGLFEDTNLCAIHAKRVTI  418

Query  121  MPKDIQLARRIRGERA  136
            MPKDIQLARRIRGERA
Sbjct  419  MPKDIQLARRIRGERA  434
```

```
>unnamed protein product [Heligmosomoides polygyrus]
Sequence ID: VDO79376.1 Length: 311
Range 1: 83 to 311

Score:444 bits(1141), Expect:2e-155,
Method:Compositional matrix adjust.,
Identities:224/239(94%), Positives:227/239(94%), Gaps:10/239(4%)

Query  1    MARTKQTARKSTGGKAPRKQLATKAARKSAPSTGGVKKPHRYRPGTVALREIRRYQKSTE  60
            MARTKQTARKSTGGKAPRKQLATKAARKSAP+TGGVKKPHRYRPGTVALREIRRYQKSTE
Sbjct  83   MARTKQTARKSTGGKAPRKQLATKAARKSAPATGGVKKPHRYRPGTVALREIRRYQKSTE  142

Query  61   LLIRKLPFQRLVREIAQDFKTDLRFQSAAIGALQEASEAYLVGLFEDTNLCAIHAKRVTI  120
            LLIRKLPFQRLVREIAQDFKTDLRFQS+A+ ALQEASEAYLVGLFEDTNLCAIHAKRVTI
Sbjct  143  LLIRKLPFQRLVREIAQDFKTDLRFQSSAVMALQEASEAYLVGLFEDTNLCAIHAKRVTI  202

Query  121  MPKDIQLARRIRGERAMSGRGKGGKGLGKGGAKRHRKVLRDNIQGITKPAIRRLARRGGV  180
            MPKDIQLARRIR          GGKGLGKGGAKRHRKVLRDNIQGITKPAIRRLARRGGV
Sbjct  203  MPKDIQLARRIR----------GGKGLGKGGAKRHRKVLRDNIQGITKPAIRRLARRGGV  252

Query  181  KRISGLIYEETRGVLKVFLENVIRDAVTYTEHAKRKTVTAMDVVYALKRQGRTLYGFGG  239
            KRISGLIYEETRGVLKVFLENVIRDAVTY EHAKRKTVTAMDVVYALKRQGRTLYGFGG
Sbjct  253  KRISGLIYEETRGVLKVFLENVIRDAVTYCEHAKRKTVTAMDVVYALKRQGRTLYGFGG  311
```

```
>uncharacterized protein LOC123826658 [Phyllostomus hastatus]
Sequence ID: XP_045706646.1 Length: 463
Range 1: 90 to 321

Score:449 bits(1154), Expect:4e-155,
Method:Compositional matrix adjust.,
Identities:227/239(95%), Positives:230/239(96%), Gaps:7/239(2%)

Query  1    MARTKQTARKSTGGKAPRKQLATKAARKSAPSTGGVKKPHRYRPGTVALREIRRYQKSTE  60
            MARTKQTARKSTGGKAPRKQLATKAARKSAP+TGGVKKPHRYRPGTVALREIRRYQKSTE
Sbjct  90   MARTKQTARKSTGGKAPRKQLATKAARKSAPATGGVKKPHRYRPGTVALREIRRYQKSTE  149

Query  61   LLIRKLPFQRLVREIAQDFKTDLRFQSAAIGALQEASEAYLVGLFEDTNLCAIHAKRVTI  120
            LLIRKLPFQRLVREIAQDFKTDLRFQS+A+ ALQEA EAYLVGLFEDTNLCAIHAKRVTI
Sbjct  150  LLIRKLPFQRLVREIAQDFKTDLRFQSSAVMALQEACEAYLVGLFEDTNLCAIHAKRVTI  209

Query  121  MPKDIQLARRIRGERAMSGRGKGGKGLGKGGAKRHRKVLRDNIQGITKPAIRRLARRGGV  180
            MPKDIQLARRIRGER       GGKGLGKGGAKRHRKVLRDNIQGITKPAIRRLARRGGV
Sbjct  210  MPKDIQLARRIRGER-------GGKGLGKGGAKRHRKVLRDNIQGITKPAIRRLARRGGV  262

Query  181  KRISGLIYEETRGVLKVFLENVIRDAVTYTEHAKRKTVTAMDVVYALKRQGRTLYGFGG  239
            KRISGLIYEETRGVLKVFLENVIRDAVTYTEHAKRKTVTAMDVVYALKRQGRTLYGFGG
Sbjct  263  KRISGLIYEETRGVLKVFLENVIRDAVTYTEHAKRKTVTAMDVVYALKRQGRTLYGFGG  321
```

```
Range 2: 328 to 463

Score:265 bits(677), Expect:5e-83,
Method:Compositional matrix adjust.,
Identities:131/136(96%), Positives:134/136(98%), Gaps:0/136(0%)

Query  1    MARTKQTARKSTGGKAPRKQLATKAARKSAPSTGGVKKPHRYRPGTVALREIRRYQKSTE  60
```

```
                 MARTKQTARKSTGGKAPRKQLATKAARKSAP+TGGVKKPHRYRPGTVALREIRRYQKSTE
Sbjct  328   MARTKQTARKSTGGKAPRKQLATKAARKSAPATGGVKKPHRYRPGTVALREIRRYQKSTE  387

Query  61    LLIRKLPFQRLVREIAQDFKTDLRFQSAAIGALQEASEAYLVGLFEDTNLCAIHAKRVTI  120
             LLIRKLPFQRLVREIAQDFKTDLRFQS+A+ ALQEA EAYLVGLFEDTNLCAIHAKRVTI
Sbjct  388   LLIRKLPFQRLVREIAQDFKTDLRFQSSAVMALQEACEAYLVGLFEDTNLCAIHAKRVTI  447

Query  121   MPKDIQLARRIRGERA  136
             MPKDIQLARRIRGERA
Sbjct  448   MPKDIQLARRIRGERA  463
```

>uncharacterized protein LOC134722518 [Mytilus trossulus]
Sequence ID: XP_063442209.1 Length: 374
Range 1: 1 to 230

Score:445 bits(1145), Expect:5e-155,
Method:Compositional matrix adjust.,
Identities:225/234(96%), Positives:228/234(97%), Gaps:4/234(1%)

```
Query  1     MARTKQTARKSTGGKAPRKQLATKAARKSAPSTGGVKKPHRYRPGTVALREIRRYQKSTE  60
             MARTKQTARKSTGGKAPRKQLATKAARKSAP+TGGVKKPHRYRPGTVALREIRRYQKSTE
Sbjct  1     MARTKQTARKSTGGKAPRKQLATKAARKSAPATGGVKKPHRYRPGTVALREIRRYQKSTE  60

Query  61    LLIRKLPFQRLVREIAQDFKTDLRFQSAAIGALQEASEAYLVGLFEDTNLCAIHAKRVTI  120
             LLIRKLPFQRLVREIAQDFKTDLRFQS+A+ ALQEASEAYLVGLFEDTNLCAIHAKRVTI
Sbjct  61    LLIRKLPFQRLVREIAQDFKTDLRFQSSAVMALQEASEAYLVGLFEDTNLCAIHAKRVTI  120

Query  121   MPKDIQLARRIRGERAMSGRGKGGKGLGKGGAKRHRKVLRDNIQGITKPAIRRLARRGGV  180
             MPKDIQLARRIRGER    RGKGGKGLGKGGAKRHRKVLRDNIQGITKPAIRRLARRGGV
Sbjct  121   MPKDIQLARRIRGER----RGKGGKGLGKGGAKRHRKVLRDNIQGITKPAIRRLARRGGV  176

Query  181   KRISGLIYEETRGVLKVFLENVIRDAVTYTEHAKRKTVTAMDVVYALKRQGRTL  234
             KRISGLIYEETRGVLKVFLENVIRDAVTYTEHAKRKTVTAMDVVYALKRQGRT
Sbjct  177   KRISGLIYEETRGVLKVFLENVIRDAVTYTEHAKRKTVTAMDVVYALKRQGRTF  230
```

Range 2: 239 to 374

Score:268 bits(684), Expect:3e-85,
Method:Compositional matrix adjust.,
Identities:132/136(97%), Positives:135/136(99%), Gaps:0/136(0%)

```
Query  1     MARTKQTARKSTGGKAPRKQLATKAARKSAPSTGGVKKPHRYRPGTVALREIRRYQKSTE  60
             MARTKQTARKSTGGKAPRKQLATKAARKSAP+TGGVKKPHRYRPGTVALREIRRYQKSTE
Sbjct  239   MARTKQTARKSTGGKAPRKQLATKAARKSAPATGGVKKPHRYRPGTVALREIRRYQKSTE  298

Query  61    LLIRKLPFQRLVREIAQDFKTDLRFQSAAIGALQEASEAYLVGLFEDTNLCAIHAKRVTI  120
             LLIRKLPFQRLVREIAQDFKTDLRFQS+A+ ALQEASEAYLVGLFEDTNLCAIHAKRVTI
Sbjct  299   LLIRKLPFQRLVREIAQDFKTDLRFQSSAVMALQEASEAYLVGLFEDTNLCAIHAKRVTI  358

Query  121   MPKDIQLARRIRGERA  136
             MPKDIQLARRIRGERA
Sbjct  359   MPKDIQLARRIRGERA  374
```

>LOW QUALITY PROTEIN: histone H3.v1-like [Ursus americanus]

```
Sequence ID: XP_045656319.1 Length: 563
Range 1: 305 to 563

Score:452 bits(1163), Expect:5e-155,
Method:Compositional matrix adjust.,
Identities:232/259(90%), Positives:235/259(90%), Gaps:20/259(7%)

Query  1    MARTKQTARKSTGGKAPRKQLATKAARKSAPSTGGVKKPHRYRPGTVALREIRRYQKSTE  60
            MARTKQTARKSTGGKAPRKQLATKAARKSAP+TGGVKKPHRYRPGTVALREIRRYQKSTE
Sbjct  305  MARTKQTARKSTGGKAPRKQLATKAARKSAPATGGVKKPHRYRPGTVALREIRRYQKSTE  364

Query  61   LLIRKLPFQRLVREIAQDFKTDLRFQSAAIGALQEASEAYLVGLFEDTNLCAIHAKRVTI  120
            LLIRKLPFQRLVREIAQDFKTDLRFQS+A+ ALQEA EAYLVGLFEDTNLCAIHAKRVTI
Sbjct  365  LLIRKLPFQRLVREIAQDFKTDLRFQSSAVMALQEACEAYLVGLFEDTNLCAIHAKRVTI  424

Query  121  MPKDIQLARRIRGER--------------------AMSGRGKGGKGLGKGGAKRHRKVLR  160
            MPKDIQLARRIRGER                    A  GRGKGGKGLGKGGAKRHRKVLR
Sbjct  425  MPKDIQLARRIRGERAACCQPDRSSPGPVSEGGLTAYCGRGKGGKGLGKGGAKRHRKVLR  484

Query  161  DNIQGITKPAIRRLARRGGVKRISGLIYEETRGVLKVFLENVIRDAVTYTEHAKRKTVTA  220
            DNIQGITKPAIRRLARRGGVKRISGLIYEETRGVLKVFLENVIRDAVTYTEHAKRKTVTA
Sbjct  485  DNIQGITKPAIRRLARRGGVKRISGLIYEETRGVLKVFLENVIRDAVTYTEHAKRKTVTA  544

Query  221  MDVVYALKRQGRTLYGFGG  239
            MDVVYALKRQGRTLYGFGG
Sbjct  545  MDVVYALKRQGRTLYGFGG  563



Range 2: 118 to 253

Score:265 bits(677), Expect:9e-82,
Method:Compositional matrix adjust.,
Identities:131/136(96%), Positives:134/136(98%), Gaps:0/136(0%)

Query  1    MARTKQTARKSTGGKAPRKQLATKAARKSAPSTGGVKKPHRYRPGTVALREIRRYQKSTE  60
            MARTKQTARKSTGGKAPRKQLATKAARKSAP+TGGVKKPHRYRPGTVALREIRRYQKSTE
Sbjct  118  MARTKQTARKSTGGKAPRKQLATKAARKSAPATGGVKKPHRYRPGTVALREIRRYQKSTE  177

Query  61   LLIRKLPFQRLVREIAQDFKTDLRFQSAAIGALQEASEAYLVGLFEDTNLCAIHAKRVTI  120
            LLIRKLPFQRLVREIAQDFKTDLRFQS+A+ ALQEA EAYLVGLFEDTNLCAIHAKRVTI
Sbjct  178  LLIRKLPFQRLVREIAQDFKTDLRFQSSAVMALQEACEAYLVGLFEDTNLCAIHAKRVTI  237

Query  121  MPKDIQLARRIRGERA  136
            MPKDIQLARRIRGERA
Sbjct  238  MPKDIQLARRIRGERA  253



Range 3: 1 to 103

Score:207 bits(528), Expect:1e-59,
Method:Compositional matrix adjust.,
Identities:103/103(100%), Positives:103/103(100%), Gaps:0/103(0%)

Query  137  MSGRGKGGKGLGKGGAKRHRKVLRDNIQGITKPAIRRLARRGGVKRISGLIYEETRGVLK  196
            MSGRGKGGKGLGKGGAKRHRKVLRDNIQGITKPAIRRLARRGGVKRISGLIYEETRGVLK
Sbjct  1    MSGRGKGGKGLGKGGAKRHRKVLRDNIQGITKPAIRRLARRGGVKRISGLIYEETRGVLK  60

Query  197  VFLENVIRDAVTYTEHAKRKTVTAMDVVYALKRQGRTLYGFGG  239
            VFLENVIRDAVTYTEHAKRKTVTAMDVVYALKRQGRTLYGFGG
```

```
Sbjct  61   VFLENVIRDAVTYTEHAKRKTVTAMDVVYALKRQGRTLYGFGG  103
```

>hypothetical protein Q8A73_017846 [Channa argus]
Sequence ID: KAK2892181.1 Length: 228
Range 1: 1 to 227

Score:439 bits(1130), Expect:6e-155,
Method:Compositional matrix adjust.,
Identities:223/238(94%), Positives:226/238(94%), Gaps:11/238(4%)

```
Query  1    MARTKQTARKSTGGKAPRKQLATKAARKSAPSTGGVKKPHRYRPGTVALREIRRYQKSTE  60
            MARTKQTARKSTGGKAPRKQLATKAARKSAP+TGGVKKPHRYRPGTVALREIRRYQKSTE
Sbjct  1    MARTKQTARKSTGGKAPRKQLATKAARKSAPATGGVKKPHRYRPGTVALREIRRYQKSTE  60

Query  61   LLIRKLPFQRLVREIAQDFKTDLRFQSAAIGALQEASEAYLVGLFEDTNLCAIHAKRVTI  120
            LLIRKLPFQRLVREIAQDFKTDLRFQS+A+ ALQEASEAYLVGLFEDTNLCAIHAKRVTI
Sbjct  61   LLIRKLPFQRLVREIAQDFKTDLRFQSSAVMALQEASEAYLVGLFEDTNLCAIHAKRVTI  120

Query  121  MPKDIQLARRIRGERAMSGRGKGGKGLGKGGAKRHRKVLRDNIQGITKPAIRRLARRGGV  180
            MPKDIQLARR           KGGKGLGKGGAKRHRKVLRDNIQGITKPAIRRLARRGGV
Sbjct  121  MPKDIQLARR-----------KGGKGLGKGGAKRHRKVLRDNIQGITKPAIRRLARRGGV  169

Query  181  KRISGLIYEETRGVLKVFLENVIRDAVTYTEHAKRKTVTAMDVVYALKRQGRTLYGFG  238
            KRISGLIYEETRGVLKVFLENVIRDAVTYTEHAKRKTVTAMDVVYALKRQGRTLYGFG
Sbjct  170  KRISGLIYEETRGVLKVFLENVIRDAVTYTEHAKRKTVTAMDVVYALKRQGRTLYGFG  227
```

>uncharacterized protein LOC129974916 [Argiope bruennichi]
Sequence ID: XP_055943681.1 Length: 433
Range 1: 1 to 231

Score:447 bits(1150), Expect:6e-155,
Method:Compositional matrix adjust.,
Identities:226/239(95%), Positives:229/239(95%), Gaps:8/239(3%)

```
Query  1    MARTKQTARKSTGGKAPRKQLATKAARKSAPSTGGVKKPHRYRPGTVALREIRRYQKSTE  60
            MARTKQTARKSTGGKAPRKQLATKAARKSAP+TGGVKKPHRYRPGTVALREIRRYQKSTE
Sbjct  1    MARTKQTARKSTGGKAPRKQLATKAARKSAPATGGVKKPHRYRPGTVALREIRRYQKSTE  60

Query  61   LLIRKLPFQRLVREIAQDFKTDLRFQSAAIGALQEASEAYLVGLFEDTNLCAIHAKRVTI  120
            LLIRKLPFQRLVREIAQDFKTDLRFQS+A+ ALQEASEAYLVGLFEDTNLCAIHAKRVTI
Sbjct  61   LLIRKLPFQRLVREIAQDFKTDLRFQSSAVMALQEASEAYLVGLFEDTNLCAIHAKRVTI  120

Query  121  MPKDIQLARRIRGERAMSGRGKGGKGLGKGGAKRHRKVLRDNIQGITKPAIRRLARRGGV  180
            MPKDIQLARRIRGER        GKGLGKGGAKRHRKVLRDNIQGITKPAIRRLARRGGV
Sbjct  121  MPKDIQLARRIRGER--------GKGLGKGGAKRHRKVLRDNIQGITKPAIRRLARRGGV  172

Query  181  KRISGLIYEETRGVLKVFLENVIRDAVTYTEHAKRKTVTAMDVVYALKRQGRTLYGFGG  239
            KRISGLIYEETRGVLKVFLENVIRDAVTYTEHAKRKTVTAMDVVYALKRQGRTLYGFG
Sbjct  173  KRISGLIYEETRGVLKVFLENVIRDAVTYTEHAKRKTVTAMDVVYALKRQGRTLYGFGA  231
```

Range 2: 298 to 433

Score:267 bits(683), Expect:3e-84,
Method:Compositional matrix adjust.,

Identities:132/136(97%), Positives:135/136(99%), Gaps:0/136(0%)

```
Query  1    MARTKQTARKSTGGKAPRKQLATKAARKSAPSTGGVKKPHRYRPGTVALREIRRYQKSTE  60
            MARTKQTARKSTGGKAPRKQLATKAARKSAP+TGGVKKPHRYRPGTVALREIRRYQKSTE
Sbjct  298  MARTKQTARKSTGGKAPRKQLATKAARKSAPATGGVKKPHRYRPGTVALREIRRYQKSTE  357

Query  61   LLIRKLPFQRLVREIAQDFKTDLRFQSAAIGALQEASEAYLVGLFEDTNLCAIHAKRVTI  120
            LLIRKLPFQRLVREIAQDFKTDLRFQS+A+ ALQEASEAYLVGLFEDTNLCAIHAKRVTI
Sbjct  358  LLIRKLPFQRLVREIAQDFKTDLRFQSSAVMALQEASEAYLVGLFEDTNLCAIHAKRVTI  417

Query  121  MPKDIQLARRIRGERA  136
            MPKDIQLARRIRGERA
Sbjct  418  MPKDIQLARRIRGERA  433
```

>uncharacterized protein [Salminus brasiliensis]
Sequence ID: XP_072526111.1 Length: 574
Range 1: 339 to 574

Score:451 bits(1161), Expect:2e-154,
Method:Compositional matrix adjust.,
Identities:228/239(95%), Positives:232/239(97%), Gaps:3/239(1%)

```
Query  1    MARTKQTARKSTGGKAPRKQLATKAARKSAPSTGGVKKPHRYRPGTVALREIRRYQKSTE  60
            MARTKQTARKSTGGKAPRKQLATKAARKSAP+TGGVKKPHRYRPGTVALREIRRYQKSTE
Sbjct  339  MARTKQTARKSTGGKAPRKQLATKAARKSAPATGGVKKPHRYRPGTVALREIRRYQKSTE  398

Query  61   LLIRKLPFQRLVREIAQDFKTDLRFQSAAIGALQEASEAYLVGLFEDTNLCAIHAKRVTI  120
            LLIRKLPFQRLVREIAQDFKTDLRFQS+A+ ALQEASEAYLVGLFEDTNLCAIHAKRVTI
Sbjct  399  LLIRKLPFQRLVREIAQDFKTDLRFQSSAVMALQEASEAYLVGLFEDTNLCAIHAKRVTI  458

Query  121  MPKDIQLARRIRGERAMSGRGKGGKGLGKGGAKRHRKVLRDNIQGITKPAIRRLARRGGV  180
            MPKDIQLAR+      MSGRGKGGKGLGKGGAKRHRKVLRDNIQGITKPAIRRLARRGGV
Sbjct  459  MPKDIQLARQ---AARMSGRGKGGKGLGKGGAKRHRKVLRDNIQGITKPAIRRLARRGGV  515

Query  181  KRISGLIYEETRGVLKVFLENVIRDAVTYTEHAKRKTVTAMDVVYALKRQGRTLYGFGG  239
            KRISGLIYEETRGVLKVFLENVIRDAVTYTEHAKRKTVTAMDVVYALKRQGRTLYGFGG
Sbjct  516  KRISGLIYEETRGVLKVFLENVIRDAVTYTEHAKRKTVTAMDVVYALKRQGRTLYGFGG  574
```

Range 2: 1 to 94

Score:188 bits(477), Expect:3e-52,
Method:Compositional matrix adjust.,
Identities:94/94(100%), Positives:94/94(100%), Gaps:0/94(0%)

```
Query  137  MSGRGKGGKGLGKGGAKRHRKVLRDNIQGITKPAIRRLARRGGVKRISGLIYEETRGVLK  196
            MSGRGKGGKGLGKGGAKRHRKVLRDNIQGITKPAIRRLARRGGVKRISGLIYEETRGVLK
Sbjct  1    MSGRGKGGKGLGKGGAKRHRKVLRDNIQGITKPAIRRLARRGGVKRISGLIYEETRGVLK  60

Query  197  VFLENVIRDAVTYTEHAKRKTVTAMDVVYALKRQ  230
            VFLENVIRDAVTYTEHAKRKTVTAMDVVYALKRQ
Sbjct  61   VFLENVIRDAVTYTEHAKRKTVTAMDVVYALKRQ  94
```

>uncharacterized protein LOC134252954 [Saccostrea cucullata]
Sequence ID: XP_062591470.1 Length: 540

```
Range 1: 1 to 233

Score:448 bits(1153), Expect:6e-154,
Method:Compositional matrix adjust.,
Identities:227/238(95%), Positives:231/238(97%), Gaps:5/238(2%)

Query  1    MARTKQTARKSTGGKAPRKQLATKAARKSAPSTGGVKKPHRYRPGTVALREIRRYQKSTE  60
            MARTKQTARKSTGGKAPRKQLATKAARKSAP+TGGVKKPHRYRPGTVALREIRRYQKSTE
Sbjct  1    MARTKQTARKSTGGKAPRKQLATKAARKSAPATGGVKKPHRYRPGTVALREIRRYQKSTE  60

Query  61   LLIRKLPFQRLVREIAQDFKTDLRFQSAAIGALQEASEAYLVGLFEDTNLCAIHAKRVTI  120
            LLIRKLPFQRLVREIAQDFKTDLRFQS+A+ ALQEASEAYLVGLFEDTNLCAIHAKRVTI
Sbjct  61   LLIRKLPFQRLVREIAQDFKTDLRFQSSAVMALQEASEAYLVGLFEDTNLCAIHAKRVTI  120

Query  121  MPKDIQLARRIRGERAMSGRGKGGKGLGKGGAKRHRKVLRDNIQGITKPAIRRLARRGGV  180
            MPKDIQLARRIRGERA     + GKGLGKGGAKRHRKVLRDNIQGITKPAIRRLARRGGV
Sbjct  121  MPKDIQLARRIRGERA-----ERGKGLGKGGAKRHRKVLRDNIQGITKPAIRRLARRGGV  175

Query  181  KRISGLIYEETRGVLKVFLENVIRDAVTYTEHAKRKTVTAMDVVYALKRQGRTLYGFG  238
            KRISGLIYEETRGVLKVFLENVIRDAVTYTEHAKRKTVTAMDVVYALKRQGRTLYGFG
Sbjct  176  KRISGLIYEETRGVLKVFLENVIRDAVTYTEHAKRKTVTAMDVVYALKRQGRTLYGFG  233



Range 2: 405 to 540

Score:268 bits(684), Expect:4e-83,
Method:Compositional matrix adjust.,
Identities:132/136(97%), Positives:135/136(99%), Gaps:0/136(0%)

Query  1    MARTKQTARKSTGGKAPRKQLATKAARKSAPSTGGVKKPHRYRPGTVALREIRRYQKSTE  60
            MARTKQTARKSTGGKAPRKQLATKAARKSAP+TGGVKKPHRYRPGTVALREIRRYQKSTE
Sbjct  405  MARTKQTARKSTGGKAPRKQLATKAARKSAPATGGVKKPHRYRPGTVALREIRRYQKSTE  464

Query  61   LLIRKLPFQRLVREIAQDFKTDLRFQSAAIGALQEASEAYLVGLFEDTNLCAIHAKRVTI  120
            LLIRKLPFQRLVREIAQDFKTDLRFQS+A+ ALQEASEAYLVGLFEDTNLCAIHAKRVTI
Sbjct  465  LLIRKLPFQRLVREIAQDFKTDLRFQSSAVMALQEASEAYLVGLFEDTNLCAIHAKRVTI  524

Query  121  MPKDIQLARRIRGERA  136
            MPKDIQLARRIRGERA
Sbjct  525  MPKDIQLARRIRGERA  540



>uncharacterized protein LOC122219292 [Panthera leo]
Sequence ID: XP_042793404.1 Length: 493
Range 1: 1 to 265

Score:447 bits(1149), Expect:6e-154,
Method:Compositional matrix adjust.,
Identities:229/265(86%), Positives:234/265(88%), Gaps:27/265(10%)

Query  1    MARTKQTARKSTGGKAPRKQLATKAARKSAPSTGGVKKPHRYRPGTVALREIRRYQKSTE  60
            MARTKQTARKSTGGKAPRKQLATKAARKSAP+TGGVKKPHRYRPGTVALREIRRYQKSTE
Sbjct  1    MARTKQTARKSTGGKAPRKQLATKAARKSAPATGGVKKPHRYRPGTVALREIRRYQKSTE  60

Query  61   LLIRKLPFQRLVREIAQDFKTDLRFQSAAIGALQEASEAYLVGLFEDTNLCAIHAKRVTI  120
            LLIRKLPFQRLVREIAQDFKTDLRFQS+A+ ALQEA EAYLVGLFEDTNLCAIHAKRVTI
Sbjct  61   LLIRKLPFQRLVREIAQDFKTDLRFQSSAVMALQEACEAYLVGLFEDTNLCAIHAKRVTI  120

Query  121  MPKDIQLARRIRGERAMSGRG---------------------------KGGKGLGKGGAK  153
```

```
          MPKDIQLARRIRGERA++  G                           KGGKGLGKGGAK
Sbjct  121  MPKDIQLARRIRGERALANPGDMSKETETVNVSGQSTGNAIKSNYTPFKGGKGLGKGGAK  180

Query  154  RHRKVLRDNIQGITKPAIRRLARRGGVKRISGLIYEETRGVLKVFLENVIRDAVTYTEHA  213
            RHRKVLRDNIQGITKPAIRRLARRGGVKRISGLIYEETRGVLKVFLENVIRDAVTYTEHA
Sbjct  181  RHRKVLRDNIQGITKPAIRRLARRGGVKRISGLIYEETRGVLKVFLENVIRDAVTYTEHA  240

Query  214  KRKTVTAMDVVYALKRQGRTLYGFG  238
            KRKTVTAMDVVYALKRQGRTLYGFG
Sbjct  241  KRKTVTAMDVVYALKRQGRTLYGFG  265
```

Range 2: 358 to 493

Score:266 bits(680), Expect:4e-83,
Method:Compositional matrix adjust.,
Identities:131/136(96%), Positives:134/136(98%), Gaps:0/136(0%)

```
Query  1    MARTKQTARKSTGGKAPRKQLATKAARKSAPSTGGVKKPHRYRPGTVALREIRRYQKSTE  60
            MARTKQTARKSTGGKAPRKQLATKAARKSAP+TGGVKKPHRYRPGTVALREIRRYQKSTE
Sbjct  358  MARTKQTARKSTGGKAPRKQLATKAARKSAPATGGVKKPHRYRPGTVALREIRRYQKSTE  417

Query  61   LLIRKLPFQRLVREIAQDFKTDLRFQSAAIGALQEASEAYLVGLFEDTNLCAIHAKRVTI  120
            LLIRKLPFQRLVREIAQDFKTDLRFQS+A+ ALQEA EAYLVGLFEDTNLCAIHAKRVTI
Sbjct  418  LLIRKLPFQRLVREIAQDFKTDLRFQSSAVMALQEACEAYLVGLFEDTNLCAIHAKRVTI  477

Query  121  MPKDIQLARRIRGERA  136
            MPKDIQLARRIRGERA
Sbjct  478  MPKDIQLARRIRGERA  493
```

>hypothetical protein EI555_001631, partial [Monodon monoceros]
Sequence ID: TKC35984.1 Length: 645
Range 1: 132 to 395

Score:451 bits(1160), Expect:1e-153,
Method:Compositional matrix adjust.,
Identities:233/264(88%), Positives:236/264(89%), Gaps:26/264(9%)

```
Query  1    MARTKQTARKSTGGKAPRKQLATKAARKSAPSTGGVKKPHRYRPGTVALREIRRYQKSTE  60
            MARTKQTARKSTGGKAPRKQLATKAARKSAP+TGGVKKPHRYRPGTVALREIRRYQKSTE
Sbjct  132  MARTKQTARKSTGGKAPRKQLATKAARKSAPATGGVKKPHRYRPGTVALREIRRYQKSTE  191

Query  61   LLIRKLPFQRLVREIAQDFKTDLRFQSAAIGALQEASEAYLVGLFEDTNLCAIHAKRVTI  120
            LLIRKLPFQRLVREIAQDFKTDLRFQS+A+ ALQEA EAYLVGLFEDTNLCAIHAKRVTI
Sbjct  192  LLIRKLPFQRLVREIAQDFKTDLRFQSSAVMALQEACEAYLVGLFEDTNLCAIHAKRVTI  251

Query  121  MPKDIQLARRIRGERA--------------------------MSGRGKGGKGLGKGGAKR  154
            MPKDIQLARRIRGERA                          MSGRGKGGKGLGKGGAKR
Sbjct  252  MPKDIQLARRIRGERALFLEPPTLSIRKNCCNAFPYFTSLGMMSGRGKGGKGLGKGGAKR  311

Query  155  HRKVLRDNIQGITKPAIRRLARRGGVKRISGLIYEETRGVLKVFLENVIRDAVTYTEHAK  214
            HRKVLRDNIQGITKPAIRRLARRGGVKRISGLIYEETRGVLKVFLENVIRDAVTYTEHAK
Sbjct  312  HRKVLRDNIQGITKPAIRRLARRGGVKRISGLIYEETRGVLKVFLENVIRDAVTYTEHAK  371

Query  215  RKTVTAMDVVYALKRQGRTLYGFG  238
            RKTVTAMDVVYALKRQGRTLYGFG
Sbjct  372  RKTVTAMDVVYALKRQGRTLYGFG  395
```

```
Range 2: 397 to 645

Score:392 bits(1008), Expect:2e-130,
Method:Compositional matrix adjust.,
Identities:218/249(88%), Positives:226/249(90%), Gaps:10/249(4%)

Query  1    MARTKQTARKSTGGKAPRKQLATKAARKSAPSTGGVKKPHRYRPGTVALREIRRYQKSTE  60
            MARTKQTARKSTGGKAPRKQLATKAARKSAP+TGGVKKPHRYRPGTVALREIRRYQKSTE
Sbjct  397  MARTKQTARKSTGGKAPRKQLATKAARKSAPATGGVKKPHRYRPGTVALREIRRYQKSTE  456

Query  61   LLIRKLPFQRLVREIAQDFKTDLRFQSAAIGALQEASEAYLVGLFEDTNLCAIHAKRVT-  119
            LLIRKLPFQRLVREIAQDFKTDLRFQS+A+ ALQEA EAYLVGLFEDTNLCAIHAKR +
Sbjct  457  LLIRKLPFQRLVREIAQDFKTDLRFQSSAVMALQEACEAYLVGLFEDTNLCAIHAKRFSQ  516

Query  120  ---------IMPKDIQLARRIRGERAMSGRGKGGKGLGKGGAKRHRKVLRDNIQGITKPA  170
                     +    ++L+RR      MSGRGKGGKGLGKGGAKRHRKVLRDNIQGITKPA
Sbjct  517  RSAQSLHKGLQVASVRLSRRAWFSCVMSGRGKGGKGLGKGGAKRHRKVLRDNIQGITKPA  576

Query  171  IRRLARRGGVKRISGLIYEETRGVLKVFLENVIRDAVTYTEHAKRKTVTAMDVVYALKRQ  230
            IRRLARRGGVKRISGLIYEETRGVLKVFLENVIRDAVTYTEHAKRKTVTAMDVVYALKRQ
Sbjct  577  IRRLARRGGVKRISGLIYEETRGVLKVFLENVIRDAVTYTEHAKRKTVTAMDVVYALKRQ  636

Query  231  GRTLYGFGG  239
            GRTLYGFGG
Sbjct  637  GRTLYGFGG  645



>uncharacterized protein LOC122782630 [Solea senegalensis]
Sequence ID: XP_043902998.1 Length: 672
Range 1: 1 to 244

Score:451 bits(1161), Expect:3e-153,
Method:Compositional matrix adjust.,
Identities:228/244(93%), Positives:233/244(95%), Gaps:10/244(4%)

Query  1    MARTKQTARKSTGGKAPRKQLATKAARKSAPSTGGVKKPHRYRPGTVALREIRRYQKSTE  60
            MARTKQTARKSTGGKAPRKQLATKAARKSAP+TGGVKKPHRYRPGTVALREIRRYQKSTE
Sbjct  1    MARTKQTARKSTGGKAPRKQLATKAARKSAPATGGVKKPHRYRPGTVALREIRRYQKSTE  60

Query  61   LLIRKLPFQRLVREIAQDFKTDLRFQSAAIGALQEASEAYLVGLFEDTNLCAIHAKRVTI  120
            LLIRKLPFQRLVREIAQDFKTDLRFQS+A+ ALQE+SEAYLVGLFEDTNLCAIHAKRVTI
Sbjct  61   LLIRKLPFQRLVREIAQDFKTDLRFQSSAVMALQESSEAYLVGLFEDTNLCAIHAKRVTI  120

Query  121  MPKDIQLARRIRGERA----------MSGRGKGGKGLGKGGAKRHRKVLRDNIQGITKPA  170
            MPKDIQLARRIRGER+          MSGRGKGGKGLGKGGAKRHRKVLRDNIQGITKPA
Sbjct  121  MPKDIQLARRIRGERSPYLCILQSDKMSGRGKGGKGLGKGGAKRHRKVLRDNIQGITKPA  180

Query  171  IRRLARRGGVKRISGLIYEETRGVLKVFLENVIRDAVTYTEHAKRKTVTAMDVVYALKRQ  230
            IRRLARRGGVKRISGLIYEETRGVLKVFLENVIRDAVTYTEHAKRKTVTAMDVVYALKRQ
Sbjct  181  IRRLARRGGVKRISGLIYEETRGVLKVFLENVIRDAVTYTEHAKRKTVTAMDVVYALKRQ  240

Query  231  GRTL  234
            GRTL
Sbjct  241  GRTL  244
```

```
Range 2: 537 to 672

Score:268 bits(684), Expect:1e-81,
Method:Compositional matrix adjust.,
Identities:131/136(96%), Positives:135/136(99%), Gaps:0/136(0%)

Query  1    MARTKQTARKSTGGKAPRKQLATKAARKSAPSTGGVKKPHRYRPGTVALREIRRYQKSTE  60
            MARTKQTARKSTGGKAPRKQLATKAARKSAP+TGGVKKPHRYRPGTVALREIRRYQKSTE
Sbjct  537  MARTKQTARKSTGGKAPRKQLATKAARKSAPATGGVKKPHRYRPGTVALREIRRYQKSTE  596

Query  61   LLIRKLPFQRLVREIAQDFKTDLRFQSAAIGALQEASEAYLVGLFEDTNLCAIHAKRVTI  120
            LLIRKLPFQRLVREIAQDFKTDLRFQS+A+ ALQE+SEAYLVGLFEDTNLCAIHAKRVTI
Sbjct  597  LLIRKLPFQRLVREIAQDFKTDLRFQSSAVMALQESSEAYLVGLFEDTNLCAIHAKRVTI  656

Query  121  MPKDIQLARRIRGERA  136
            MPKDIQLARRIRGERA
Sbjct  657  MPKDIQLARRIRGERA  672


>uncharacterized protein LOC134378130 [Cynocephalus volans]
Sequence ID: XP_062953180.1 Length: 503
Range 1: 90 to 360

Score:445 bits(1145), Expect:3e-153,
Method:Compositional matrix adjust.,
Identities:230/271(85%), Positives:235/271(86%), Gaps:33/271(12%)

Query  1    MARTKQTARKSTGGKAPRKQLATKAARKSAPSTGGVKKPHRYRPGTVALREIRRYQKSTE  60
            MARTKQTARKSTGGKAPRKQLATKAARKSAP+TGGVKKPHRYRPGTVALREIRRYQKSTE
Sbjct  90   MARTKQTARKSTGGKAPRKQLATKAARKSAPATGGVKKPHRYRPGTVALREIRRYQKSTE  149

Query  61   LLIRKLPFQRLVREIAQDFKTDLRFQSAAIGALQEASEAYLVGLFEDTNLCAIHAKRVTI  120
            LLIRKLPFQRLVREIAQDFKTDLRFQS+A+ ALQEA EAYLVGLFEDTNLCAIHAKRVTI
Sbjct  150  LLIRKLPFQRLVREIAQDFKTDLRFQSSAVMALQEACEAYLVGLFEDTNLCAIHAKRVTI  209

Query  121  MPKDIQLARRIRGERA---------------------------------MSGRGKGGKGL  147
            MPKDIQLARRIRG+ +                                 MSGRGKGGKGL
Sbjct  210  MPKDIQLARRIRGDSSEKRREQEGALSGIFTRSRTPGNVELATFLGAADMSGRGKGGKGL  269

Query  148  GKGGAKRHRKVLRDNIQGITKPAIRRLARRGGVKRISGLIYEETRGVLKVFLENVIRDAV  207
            GKGGAKRHRKVLRDNIQGITKPAIRRLARRGGVKRISGLIYEETRGVLKVFLENVIRDAV
Sbjct  270  GKGGAKRHRKVLRDNIQGITKPAIRRLARRGGVKRISGLIYEETRGVLKVFLENVIRDAV  329

Query  208  TYTEHAKRKTVTAMDVVYALKRQGRTLYGFG  238
            TYTEHAKRKTVTAMDVVYALKRQGRTLYGFG
Sbjct  330  TYTEHAKRKTVTAMDVVYALKRQGRTLYGFG  360


Range 2: 368 to 503

Score:265 bits(678), Expect:1e-82,
Method:Compositional matrix adjust.,
Identities:131/136(96%), Positives:134/136(98%), Gaps:0/136(0%)

Query  1    MARTKQTARKSTGGKAPRKQLATKAARKSAPSTGGVKKPHRYRPGTVALREIRRYQKSTE  60
            MARTKQTARKSTGGKAPRKQLATKAARKSAP+TGGVKKPHRYRPGTVALREIRRYQKSTE
Sbjct  368  MARTKQTARKSTGGKAPRKQLATKAARKSAPATGGVKKPHRYRPGTVALREIRRYQKSTE  427

Query  61   LLIRKLPFQRLVREIAQDFKTDLRFQSAAIGALQEASEAYLVGLFEDTNLCAIHAKRVTI  120
```

```
          LLIRKLPFQRLVREIAQDFKTDLRFQS+A+ ALQEA EAYLVGLFEDTNLCAIHAKRVTI
Sbjct  428  LLIRKLPFQRLVREIAQDFKTDLRFQSSAVMALQEACEAYLVGLFEDTNLCAIHAKRVTI  487

Query  121  MPKDIQLARRIRGERA  136
            MPKDIQLARRIRGERA
Sbjct  488  MPKDIQLARRIRGERA  503
```

>LOW QUALITY PROTEIN: uncharacterized protein LOC131481407 [Ochotona princeps]
Sequence ID: XP_058526226.1 Length: 693
Range 1: 426 to 693

Score:452 bits(1162), Expect:4e-153,
Method:Compositional matrix adjust.,
Identities:233/268(87%), Positives:236/268(88%), Gaps:29/268(10%)

```
Query  1    MARTKQTARKSTGGKAPRKQLATKAARKSAPSTGGVKKPHRYRPGTVALREIRRYQKSTE  60
            MARTKQTARKSTGGKAPRKQLATKAARKSAP+TGGVKKPHRYRPGTVALREIRRYQKSTE
Sbjct  426  MARTKQTARKSTGGKAPRKQLATKAARKSAPATGGVKKPHRYRPGTVALREIRRYQKSTE  485

Query  61   LLIRKLPFQRLVREIAQDFKTDLRFQSAAIGALQEASEAYLVGLFEDTNLCAIHAKRVTI  120
            LLIRKLPFQRLVREIAQDFKTDLRFQS+A+ ALQEA EAYLVGLFEDTNLCAIHAKRVTI
Sbjct  486  LLIRKLPFQRLVREIAQDFKTDLRFQSSAVMALQEACEAYLVGLFEDTNLCAIHAKRVTI  545

Query  121  MPKDIQLARRIRGERA-----------------------------MSGRGKGGKGLGKGG  151
            MPKDIQLARRIRGER                              MSGRGKGGKGLGKGG
Sbjct  546  MPKDIQLARRIRGERDISLRSAELLYKQTHVGLTGSFLLCFLFLRMSGRGKGGKGLGKGG  605

Query  152  AKRHRKVLRDNIQGITKPAIRRLARRGGVKRISGLIYEETRGVLKVFLENVIRDAVTYTE  211
            AKRHRKVLRDNIQGITKPAIRRLARRGGVKRISGLIYEETRGVLKVFLENVIRDAVTYTE
Sbjct  606  AKRHRKVLRDNIQGITKPAIRRLARRGGVKRISGLIYEETRGVLKVFLENVIRDAVTYTE  665

Query  212  HAKRKTVTAMDVVYALKRQGRTLYGFGG  239
            HAKRKTVTAMDVVYALKRQGRTLYGFGG
Sbjct  666  HAKRKTVTAMDVVYALKRQGRTLYGFGG  693
```

Range 2: 114 to 248

Score:265 bits(676), Expect:2e-80,
Method:Compositional matrix adjust.,
Identities:130/135(96%), Positives:133/135(98%), Gaps:0/135(0%)

```
Query  1    MARTKQTARKSTGGKAPRKQLATKAARKSAPSTGGVKKPHRYRPGTVALREIRRYQKSTE  60
            MARTKQTARKSTGGKAPRKQLATKAARKSAP+TGGVKKPHRYRPGTVALREIRRYQKSTE
Sbjct  114  MARTKQTARKSTGGKAPRKQLATKAARKSAPATGGVKKPHRYRPGTVALREIRRYQKSTE  173

Query  61   LLIRKLPFQRLVREIAQDFKTDLRFQSAAIGALQEASEAYLVGLFEDTNLCAIHAKRVTI  120
            LLIRKLPFQRLVREIAQDFKTDLRFQS+A+ ALQEA EAYLVGLFEDTNLCAIHAKRVTI
Sbjct  174  LLIRKLPFQRLVREIAQDFKTDLRFQSSAVMALQEACEAYLVGLFEDTNLCAIHAKRVTI  233

Query  121  MPKDIQLARRIRGER  135
            MPKDIQLARRIRGER
Sbjct  234  MPKDIQLARRIRGER  248
```

Range 3: 1 to 97

```
Score:193 bits(491), Expect:2e-53,
Method:Compositional matrix adjust.,
Identities:97/97(100%), Positives:97/97(100%), Gaps:0/97(0%)

Query  137  MSGRGKGGKGLGKGGAKRHRKVLRDNIQGITKPAIRRLARRGGVKRISGLIYEETRGVLK  196
            MSGRGKGGKGLGKGGAKRHRKVLRDNIQGITKPAIRRLARRGGVKRISGLIYEETRGVLK
Sbjct  1    MSGRGKGGKGLGKGGAKRHRKVLRDNIQGITKPAIRRLARRGGVKRISGLIYEETRGVLK  60

Query  197  VFLENVIRDAVTYTEHAKRKTVTAMDVVYALKRQGRT  233
            VFLENVIRDAVTYTEHAKRKTVTAMDVVYALKRQGRT
Sbjct  61   VFLENVIRDAVTYTEHAKRKTVTAMDVVYALKRQGRT  97
```

```
>uncharacterized protein LOC132564900 [Ylistrum balloti]
Sequence ID: XP_060085497.1 Length: 544
Range 1: 1 to 228

Score:441 bits(1133), Expect:1e-150,
Method:Compositional matrix adjust.,
Identities:224/234(96%), Positives:227/234(97%), Gaps:6/234(2%)

Query  1    MARTKQTARKSTGGKAPRKQLATKAARKSAPSTGGVKKPHRYRPGTVALREIRRYQKSTE  60
            MARTKQTARKSTGGKAPRKQLATKAARKSAP+TGGVKKPHRYRPGTVALREIRRYQKSTE
Sbjct  1    MARTKQTARKSTGGKAPRKQLATKAARKSAPATGGVKKPHRYRPGTVALREIRRYQKSTE  60

Query  61   LLIRKLPFQRLVREIAQDFKTDLRFQSAAIGALQEASEAYLVGLFEDTNLCAIHAKRVTI  120
            LLIRKLPFQRLVREIAQDFKTDLRFQS+A+ ALQEASEAYLVGLFEDTNLCAIHAKRVTI
Sbjct  61   LLIRKLPFQRLVREIAQDFKTDLRFQSSAVMALQEASEAYLVGLFEDTNLCAIHAKRVTI  120

Query  121  MPKDIQLARRIRGERAMSGRGKGGKGLGKGGAKRHRKVLRDNIQGITKPAIRRLARRGGV  180
            MPKDIQLARRIRGER      KGGKGLGKGGAKRHRKVLRDNIQGITKPAIRRLARRGGV
Sbjct  121  MPKDIQLARRIRGER------KGGKGLGKGGAKRHRKVLRDNIQGITKPAIRRLARRGGV  174

Query  181  KRISGLIYEETRGVLKVFLENVIRDAVTYTEHAKRKTVTAMDVVYALKRQGRTL  234
            KRISGLIYEETRGVLKVFLENVIRDAVTYTEHAKRKTVTAMDVVYALKRQGRTL
Sbjct  175  KRISGLIYEETRGVLKVFLENVIRDAVTYTEHAKRKTVTAMDVVYALKRQGRTL  228
```

```
Range 2: 409 to 544

Score:267 bits(683), Expect:7e-83,
Method:Compositional matrix adjust.,
Identities:132/136(97%), Positives:135/136(99%), Gaps:0/136(0%)

Query  1    MARTKQTARKSTGGKAPRKQLATKAARKSAPSTGGVKKPHRYRPGTVALREIRRYQKSTE  60
            MARTKQTARKSTGGKAPRKQLATKAARKSAP+TGGVKKPHRYRPGTVALREIRRYQKSTE
Sbjct  409  MARTKQTARKSTGGKAPRKQLATKAARKSAPATGGVKKPHRYRPGTVALREIRRYQKSTE  468

Query  61   LLIRKLPFQRLVREIAQDFKTDLRFQSAAIGALQEASEAYLVGLFEDTNLCAIHAKRVTI  120
            LLIRKLPFQRLVREIAQDFKTDLRFQS+A+ ALQEASEAYLVGLFEDTNLCAIHAKRVTI
Sbjct  469  LLIRKLPFQRLVREIAQDFKTDLRFQSSAVMALQEASEAYLVGLFEDTNLCAIHAKRVTI  528

Query  121  MPKDIQLARRIRGERA  136
            MPKDIQLARRIRGERA
Sbjct  529  MPKDIQLARRIRGERA  544
```

```
>Histone H3 [Heterocephalus glaber]
Sequence ID: EHB13139.1 Length: 222
Range 1: 1 to 222

Score:428 bits(1100), Expect:2e-150,
Method:Compositional matrix adjust.,
Identities:218/239(91%), Positives:221/239(92%), Gaps:17/239(7%)

Query  1    MARTKQTARKSTGGKAPRKQLATKAARKSAPSTGGVKKPHRYRPGTVALREIRRYQKSTE  60
            MARTKQTARKSTGGKAPRKQLATKAARKSAP+TGGVKKPHRYRPGTVALREIRRYQKSTE
Sbjct  1    MARTKQTARKSTGGKAPRKQLATKAARKSAPATGGVKKPHRYRPGTVALREIRRYQKSTE  60

Query  61   LLIRKLPFQRLVREIAQDFKTDLRFQSAAIGALQEASEAYLVGLFEDTNLCAIHAKRVTI  120
            LLIRKLPFQRLVREIAQDFKTDLRFQS+A+ ALQEASEAYLVGLFEDTNLCAIHAKRVTI
Sbjct  61   LLIRKLPFQRLVREIAQDFKTDLRFQSSAVMALQEASEAYLVGLFEDTNLCAIHAKRVTI  120

Query  121  MPKDIQLARRIRGERAMSGRGKGGKGLGKGGAKRHRKVLRDNIQGITKPAIRRLARRGGV  180
            MPKDIQLARRIR                 GGAKRHRKVLRDNIQGITKPAIRRLARRGGV
Sbjct  121  MPKDIQLARRIR-----------------GGAKRHRKVLRDNIQGITKPAIRRLARRGGV  163

Query  181  KRISGLIYEETRGVLKVFLENVIRDAVTYTEHAKRKTVTAMDVVYALKRQGRTLYGFGG  239
            KRISGLIYEETRGVLKVFLENVIRDAVTYTEHAKRKTVTAMDVVYALKRQGRTLYGFGG
Sbjct  164  KRISGLIYEETRGVLKVFLENVIRDAVTYTEHAKRKTVTAMDVVYALKRQGRTLYGFGG  222


>PREDICTED: uncharacterized protein LOC105526832 [Colobus angolensis palliatus]
Sequence ID: XP_011818312.1 Length: 892
Range 1: 1 to 232

Score:451 bits(1160), Expect:2e-150,
Method:Compositional matrix adjust.,
Identities:227/239(95%), Positives:230/239(96%), Gaps:7/239(2%)

Query  1    MARTKQTARKSTGGKAPRKQLATKAARKSAPSTGGVKKPHRYRPGTVALREIRRYQKSTE  60
            MARTKQTARKSTGGKAPRKQLATKAARKSAP+TGGVKKPHRYRPGTVALREIRRYQKSTE
Sbjct  1    MARTKQTARKSTGGKAPRKQLATKAARKSAPATGGVKKPHRYRPGTVALREIRRYQKSTE  60

Query  61   LLIRKLPFQRLVREIAQDFKTDLRFQSAAIGALQEASEAYLVGLFEDTNLCAIHAKRVTI  120
            LLIRKLPFQRLVREIAQDFKTDLRFQS+A+ ALQEA EAYLVGLFEDTNLCAIHAKRVTI
Sbjct  61   LLIRKLPFQRLVREIAQDFKTDLRFQSSAVMALQEACEAYLVGLFEDTNLCAIHAKRVTI  120

Query  121  MPKDIQLARRIRGERAMSGRGKGGKGLGKGGAKRHRKVLRDNIQGITKPAIRRLARRGGV  180
            MPKDIQLARRIRGER       GGKGLGKGGAKRHRKVLRDNIQGITKPAIRRLARRGGV
Sbjct  121  MPKDIQLARRIRGER-------GGKGLGKGGAKRHRKVLRDNIQGITKPAIRRLARRGGV  173

Query  181  KRISGLIYEETRGVLKVFLENVIRDAVTYTEHAKRKTVTAMDVVYALKRQGRTLYGFGG  239
            KRISGLIYEETRGVLKVFLENVIRDAVTYTEHAKRKTVTAMDVVYALKRQGRTLYGFGG
Sbjct  174  KRISGLIYEETRGVLKVFLENVIRDAVTYTEHAKRKTVTAMDVVYALKRQGRTLYGFGG  232


Range 2: 757 to 892

Score:266 bits(680), Expect:1e-79,
Method:Compositional matrix adjust.,
Identities:131/136(96%), Positives:134/136(98%), Gaps:0/136(0%)

Query  1    MARTKQTARKSTGGKAPRKQLATKAARKSAPSTGGVKKPHRYRPGTVALREIRRYQKSTE  60
            MARTKQTARKSTGGKAPRKQLATKAARKSAP+TGGVKKPHRYRPGTVALREIRRYQKSTE
```

```
Sbjct  757  MARTKQTARKSTGGKAPRKQLATKAARKSAPATGGVKKPHRYRPGTVALREIRRYQKSTE  816

Query  61   LLIRKLPFQRLVREIAQDFKTDLRFQSAAIGALQEASEAYLVGLFEDTNLCAIHAKRVTI  120
            LLIRKLPFQRLVREIAQDFKTDLRFQS+A+ ALQEA EAYLVGLFEDTNLCAIHAKRVTI
Sbjct  817  LLIRKLPFQRLVREIAQDFKTDLRFQSSAVMALQEACEAYLVGLFEDTNLCAIHAKRVTI  876

Query  121  MPKDIQLARRIRGERA  136
            MPKDIQLARRIRGERA
Sbjct  877  MPKDIQLARRIRGERA  892
```

>uncharacterized protein LOC129543310 [Moschus berezovskii]
Sequence ID: XP_055262377.1 Length: 418
Range 1: 1 to 252

Score:434 bits(1117), Expect:4e-150,
Method:Compositional matrix adjust.,
Identities:224/252(89%), Positives:227/252(90%), Gaps:23/252(9%)

```
Query  1    MARTKQTARKSTGGKAPRKQLATKAARKSAPSTGGVKKPHRYRPGTVALREIRRYQKSTE  60
            MARTKQTARKSTGGKAPRKQLATKAARKSAP+TGGVKKPHRYRPGTVALREIRRYQKSTE
Sbjct  1    MARTKQTARKSTGGKAPRKQLATKAARKSAPATGGVKKPHRYRPGTVALREIRRYQKSTE  60

Query  61   LLIRKLPFQRLVREIAQDFKTDLRFQSAAIGALQEASEAYLVGLFEDTNLCAIHAKRVTI  120
            LLIRKLPFQRLVREIAQDFKTDLRFQS+A+ ALQEA EAYLVGLFEDTNLCAIHAKRVTI
Sbjct  61   LLIRKLPFQRLVREIAQDFKTDLRFQSSAVMALQEACEAYLVGLFEDTNLCAIHAKRVTI  120

Query  121  MPKDIQLARRIRGERA-----------------------MSGRGKGGKGLGKGGAKRHRK  157
            MPKDIQLARRIRGERA                       MSGRGKGGKGLGKGGAKRHRK
Sbjct  121  MPKDIQLARRIRGERAYKASNLWPLAEGRLRRFPGLVLGMSGRGKGGKGLGKGGAKRHRK  180

Query  158  VLRDNIQGITKPAIRRLARRGGVKRISGLIYEETRGVLKVFLENVIRDAVTYTEHAKRKT  217
            VLRDNIQGITKPAIRRLARRGGVKRISGLIYEETRGVLKVFLENVIRDAVTYTEHAKRKT
Sbjct  181  VLRDNIQGITKPAIRRLARRGGVKRISGLIYEETRGVLKVFLENVIRDAVTYTEHAKRKT  240

Query  218  VTAMDVVYALKR  229
            VTAMDVVYALKR
Sbjct  241  VTAMDVVYALKR  252
```

Range 2: 283 to 418

Score:265 bits(678), Expect:1e-83,
Method:Compositional matrix adjust.,
Identities:131/136(96%), Positives:134/136(98%), Gaps:0/136(0%)

```
Query  1    MARTKQTARKSTGGKAPRKQLATKAARKSAPSTGGVKKPHRYRPGTVALREIRRYQKSTE  60
            MARTKQTARKSTGGKAPRKQLATKAARKSAP+TGGVKKPHRYRPGTVALREIRRYQKSTE
Sbjct  283  MARTKQTARKSTGGKAPRKQLATKAARKSAPATGGVKKPHRYRPGTVALREIRRYQKSTE  342

Query  61   LLIRKLPFQRLVREIAQDFKTDLRFQSAAIGALQEASEAYLVGLFEDTNLCAIHAKRVTI  120
            LLIRKLPFQRLVREIAQDFKTDLRFQS+A+ ALQEA EAYLVGLFEDTNLCAIHAKRVTI
Sbjct  343  LLIRKLPFQRLVREIAQDFKTDLRFQSSAVMALQEACEAYLVGLFEDTNLCAIHAKRVTI  402

Query  121  MPKDIQLARRIRGERA  136
            MPKDIQLARRIRGERA
Sbjct  403  MPKDIQLARRIRGERA  418
```

```
>histone H3.v1-like [Scomber scombrus]
Sequence ID: XP_062289628.1 Length: 268
Range 1: 44 to 268

Score:423 bits(1087), Expect:8e-148,
Method:Compositional matrix adjust.,
Identities:216/240(90%), Positives:222/240(92%), Gaps:16/240(6%)

Query  1    MARTKQTARKSTGGKAPRKQLATKAARKSAPSTGGVKKPHRYRPGTVALREIRRYQKSTE  60
            MARTKQTARKSTGGKAPRKQLATKAARKSAP+TGGVKKPHRYRPGTVALREIRRYQKSTE
Sbjct  44   MARTKQTARKSTGGKAPRKQLATKAARKSAPATGGVKKPHRYRPGTVALREIRRYQKSTE  103

Query  61   LLIRKLPFQRLVREIAQDFKTDLRFQSAAIGALQEASEAYLVGLFEDTNLCAIHAKR-VT  119
            LLIRKLPFQRLVREIAQDFKTDLRFQS+A+ ALQEASEAYLVGLFEDTNLCAIHAKR ++
Sbjct  104  LLIRKLPFQRLVREIAQDFKTDLRFQSSAVMALQEASEAYLVGLFEDTNLCAIHAKRKIS  163

Query  120  IMPKDIQLARRIRGERAMSGRGKGGKGLGKGGAKRHRKVLRDNIQGITKPAIRRLARRGG  179
             +               MSGRGKGGKGLGKGGAKRHRKVLRDNIQGITKPAIRRLARRGG
Sbjct  164  FI---------------MSGRGKGGKGLGKGGAKRHRKVLRDNIQGITKPAIRRLARRGG  208

Query  180  VKRISGLIYEETRGVLKVFLENVIRDAVTYTEHAKRKTVTAMDVVYALKRQGRTLYGFGG  239
            VKRISGLIYEETRGVLKVFLENVIRDAVTYTEHAKRKTVTAMDVVYALKRQGRTLYGFGG
Sbjct  209  VKRISGLIYEETRGVLKVFLENVIRDAVTYTEHAKRKTVTAMDVVYALKRQGRTLYGFGG  268


>uncharacterized protein LOC133489876 [Phyllopteryx taeniolatus]
Sequence ID: XP_061655078.1 Length: 259
Range 1: 38 to 259

Score:422 bits(1084), Expect:1e-147,
Method:Compositional matrix adjust.,
Identities:216/239(90%), Positives:219/239(91%), Gaps:17/239(7%)

Query  1    MARTKQTARKSTGGKAPRKQLATKAARKSAPSTGGVKKPHRYRPGTVALREIRRYQKSTE  60
            MARTKQTARKSTGGKAPRKQLATKAARKSAP+TGGVKKPHRYRPGTVALREIRRYQKSTE
Sbjct  38   MARTKQTARKSTGGKAPRKQLATKAARKSAPATGGVKKPHRYRPGTVALREIRRYQKSTE  97

Query  61   LLIRKLPFQRLVREIAQDFKTDLRFQSAAIGALQEASEAYLVGLFEDTNLCAIHAKRVTI  120
            LLIRKLPFQRLVREIAQDFKTDLRFQ +A+ ALQEASEAYLVGLFEDTNLCAIHAKR T
Sbjct  98   LLIRKLPFQRLVREIAQDFKTDLRFQGSAVMALQEASEAYLVGLFEDTNLCAIHAKRST-  156

Query  121  MPKDIQLARRIRGERAMSGRGKGGKGLGKGGAKRHRKVLRDNIQGITKPAIRRLARRGGV  180
                            MSGRGKGGKGLGKGGAKRHRKVLRDNIQGITKPAIRRLARRGGV
Sbjct  157  ----------------MSGRGKGGKGLGKGGAKRHRKVLRDNIQGITKPAIRRLARRGGV  200

Query  181  KRISGLIYEETRGVLKVFLENVIRDAVTYTEHAKRKTVTAMDVVYALKRQGRTLYGFGG  239
            KRISGLIYEETRGVLKVFLENVIRDAVTYTEHAKRKTVTAMDVVYALKRQGRTLYGFGG
Sbjct  201  KRISGLIYEETRGVLKVFLENVIRDAVTYTEHAKRKTVTAMDVVYALKRQGRTLYGFGG  259


>uncharacterized protein LOC106064839 [Biomphalaria glabrata]
Sequence ID: XP_055879400.1 Length: 366
Range 1: 142 to 366

Score:425 bits(1093), Expect:2e-147,
Method:Compositional matrix adjust.,
Identities:217/239(91%), Positives:221/239(92%), Gaps:14/239(5%)
```

```
Query  1    MARTKQTARKSTGGKAPRKQLATKAARKSAPSTGGVKKPHRYRPGTVALREIRRYQKSTE  60
            MARTKQTARKSTGGKAPRKQLATKAARKSAP+TGGVKKPHRYRPGTVALREIRRYQKSTE
Sbjct  142  MARTKQTARKSTGGKAPRKQLATKAARKSAPATGGVKKPHRYRPGTVALREIRRYQKSTE  201

Query  61   LLIRKLPFQRLVREIAQDFKTDLRFQSAAIGALQEASEAYLVGLFEDTNLCAIHAKRVTI  120
            LLIRKLPFQRLVREIAQDFKTDLRFQS+A+ ALQEASEAYLVGLFEDTNLCAIHAKR+
Sbjct  202  LLIRKLPFQRLVREIAQDFKTDLRFQSSAVMALQEASEAYLVGLFEDTNLCAIHAKRLN-  260

Query  121  MPKDIQLARRIRGERAMSGRGKGGKGLGKGGAKRHRKVLRDNIQGITKPAIRRLARRGGV  180
                         E  MSGRGKGGKGLGKGGAKRHRKVLRDNIQGITKPAIRRLARRGGV
Sbjct  261  -------------ESTMSGRGKGGKGLGKGGAKRHRKVLRDNIQGITKPAIRRLARRGGV  307

Query  181  KRISGLIYEETRGVLKVFLENVIRDAVTYTEHAKRKTVTAMDVVYALKRQGRTLYGFGG  239
            KRISGLIYEETRGVLKVFLENVIRDAVTYTEHAKRKTVTAMDVVYALKRQGRTLYGFGG
Sbjct  308  KRISGLIYEETRGVLKVFLENVIRDAVTYTEHAKRKTVTAMDVVYALKRQGRTLYGFGG  366
```

Range 2: 1 to 103

Score:206 bits(524), Expect:4e-61,
Method:Compositional matrix adjust.,
Identities:103/103(100%), Positives:103/103(100%), Gaps:0/103(0%)

```
Query  137  MSGRGKGGKGLGKGGAKRHRKVLRDNIQGITKPAIRRLARRGGVKRISGLIYEETRGVLK  196
            MSGRGKGGKGLGKGGAKRHRKVLRDNIQGITKPAIRRLARRGGVKRISGLIYEETRGVLK
Sbjct  1    MSGRGKGGKGLGKGGAKRHRKVLRDNIQGITKPAIRRLARRGGVKRISGLIYEETRGVLK  60

Query  197  VFLENVIRDAVTYTEHAKRKTVTAMDVVYALKRQGRTLYGFGG  239
            VFLENVIRDAVTYTEHAKRKTVTAMDVVYALKRQGRTLYGFGG
Sbjct  61   VFLENVIRDAVTYTEHAKRKTVTAMDVVYALKRQGRTLYGFGG  103
```

>histone H3 [Anabarilius grahami]
Sequence ID: ROL44179.1 Length: 777
Range 1: 1 to 225

Score:439 bits(1128), Expect:5e-147,
Method:Compositional matrix adjust.,
Identities:221/234(94%), Positives:224/234(95%), Gaps:9/234(3%)

```
Query  1    MARTKQTARKSTGGKAPRKQLATKAARKSAPSTGGVKKPHRYRPGTVALREIRRYQKSTE  60
            MARTKQTARKSTGGKAPRKQLATKAARKSAP+TGGVKKPHRYRPGTVALREIRRYQKSTE
Sbjct  1    MARTKQTARKSTGGKAPRKQLATKAARKSAPATGGVKKPHRYRPGTVALREIRRYQKSTE  60

Query  61   LLIRKLPFQRLVREIAQDFKTDLRFQSAAIGALQEASEAYLVGLFEDTNLCAIHAKRVTI  120
            LLIRKLPFQRLVREIAQDFKTDLRFQS+A+ ALQEASEAYLVGLFEDTNLCAIHAKRVTI
Sbjct  61   LLIRKLPFQRLVREIAQDFKTDLRFQSSAVMALQEASEAYLVGLFEDTNLCAIHAKRVTI  120

Query  121  MPKDIQLARRIRGERAMSGRGKGGKGLGKGGAKRHRKVLRDNIQGITKPAIRRLARRGGV  180
            MPKDIQLARR         RGKGGKGLGKGGAKRHRKVLRDNIQGITKPAIRRLARRGGV
Sbjct  121  MPKDIQLARR---------RGKGGKGLGKGGAKRHRKVLRDNIQGITKPAIRRLARRGGV  171

Query  181  KRISGLIYEETRGVLKVFLENVIRDAVTYTEHAKRKTVTAMDVVYALKRQGRTL  234
            KRISGLIYEETRGVLKVFLENVIRDAVTYTEHAKRKTVTAMDVVYALKRQGRTL
Sbjct  172  KRISGLIYEETRGVLKVFLENVIRDAVTYTEHAKRKTVTAMDVVYALKRQGRTL  225
```

```
>histone H3-like centromeric protein cid [Gracilinanus agilis]
Sequence ID: XP_044528527.1 Length: 263
Range 1: 46 to 263

Score:420 bits(1079), Expect:1e-146,
Method:Compositional matrix adjust.,
Identities:214/239(90%), Positives:217/239(90%), Gaps:21/239(8%)

Query  1    MARTKQTARKSTGGKAPRKQLATKAARKSAPSTGGVKKPHRYRPGTVALREIRRYQKSTE  60
            MARTKQTARKSTGGKAPRKQLATKAARKSAP+TGGVKKPHRYRPGTVALREIRRYQKSTE
Sbjct  46   MARTKQTARKSTGGKAPRKQLATKAARKSAPATGGVKKPHRYRPGTVALREIRRYQKSTE  105

Query  61   LLIRKLPFQRLVREIAQDFKTDLRFQSAAIGALQEASEAYLVGLFEDTNLCAIHAKRVTI  120
            LLIRKLPFQRLVREIAQDFKTDLRFQS+A+ ALQEASEAYLVGLFEDTNLCAIH
Sbjct  106  LLIRKLPFQRLVREIAQDFKTDLRFQSSAVMALQEASEAYLVGLFEDTNLCAIH------  159

Query  121  MPKDIQLARRIRGERAMSGRGKGGKGLGKGGAKRHRKVLRDNIQGITKPAIRRLARRGGV  180
                           AMSGRGKGGKGLGKGGAKRHRKVLRDNIQGITKPAIRRLARRGGV
Sbjct  160  ---------------AMSGRGKGGKGLGKGGAKRHRKVLRDNIQGITKPAIRRLARRGGV  204

Query  181  KRISGLIYEETRGVLKVFLENVIRDAVTYTEHAKRKTVTAMDVVYALKRQGRTLYGFGG  239
            KRISGLIYEETRGVLKVFLENVIRDAVTYTEHAKRKTVTAMDVVYALKRQGRTLYGFGG
Sbjct  205  KRISGLIYEETRGVLKVFLENVIRDAVTYTEHAKRKTVTAMDVVYALKRQGRTLYGFGG  263



>uncharacterized protein LOC112983655 [Dromaius novaehollandiae]
Sequence ID: XP_064367322.1 Length: 329
Range 1: 105 to 329

Score:422 bits(1084), Expect:2e-146,
Method:Compositional matrix adjust.,
Identities:216/239(90%), Positives:220/239(92%), Gaps:14/239(5%)

Query  1    MARTKQTARKSTGGKAPRKQLATKAARKSAPSTGGVKKPHRYRPGTVALREIRRYQKSTE  60
            MARTKQTARKSTGGKAPRKQLATKAARKSAP+TGGVKKPHRYRPGTVALREIRRYQKSTE
Sbjct  105  MARTKQTARKSTGGKAPRKQLATKAARKSAPATGGVKKPHRYRPGTVALREIRRYQKSTE  164

Query  61   LLIRKLPFQRLVREIAQDFKTDLRFQSAAIGALQEASEAYLVGLFEDTNLCAIHAKRVTI  120
            LLIRKLPFQRLVREIAQDFKTDLRFQS+A+ ALQEASEAYLVGLFEDTNLCAIHAKR
Sbjct  165  LLIRKLPFQRLVREIAQDFKTDLRFQSSAVMALQEASEAYLVGLFEDTNLCAIHAKRCLG  224

Query  121  MPKDIQLARRIRGERAMSGRGKGGKGLGKGGAKRHRKVLRDNIQGITKPAIRRLARRGGV  180
            +               MSGRGKGGKGLGKGGAKRHRKVLRDNIQGITKPAIRRLARRGGV
Sbjct  225  V--------------VMSGRGKGGKGLGKGGAKRHRKVLRDNIQGITKPAIRRLARRGGV  270

Query  181  KRISGLIYEETRGVLKVFLENVIRDAVTYTEHAKRKTVTAMDVVYALKRQGRTLYGFGG  239
            KRISGLIYEETRGVLKVFLENVIRDAVTYTEHAKRKTVTAMDVVYALKRQGRTLYGFGG
Sbjct  271  KRISGLIYEETRGVLKVFLENVIRDAVTYTEHAKRKTVTAMDVVYALKRQGRTLYGFGG  329



Range 2: 1 to 96

Score:191 bits(484), Expect:2e-55,
Method:Compositional matrix adjust.,
Identities:96/96(100%), Positives:96/96(100%), Gaps:0/96(0%)

Query  137  MSGRGKGGKGLGKGGAKRHRKVLRDNIQGITKPAIRRLARRGGVKRISGLIYEETRGVLK  196
            MSGRGKGGKGLGKGGAKRHRKVLRDNIQGITKPAIRRLARRGGVKRISGLIYEETRGVLK
```

```
Sbjct  1    MSGRGKGGKGLGKGGAKRHRKVLRDNIQGITKPAIRRLARRGGVKRISGLIYEETRGVLK  60

Query  197  VFLENVIRDAVTYTEHAKRKTVTAMDVVYALKRQGR  232
            VFLENVIRDAVTYTEHAKRKTVTAMDVVYALKRQGR
Sbjct  61   VFLENVIRDAVTYTEHAKRKTVTAMDVVYALKRQGR  96
```

>hypothetical protein DBR06_SOUSAS30410014 [Sousa chinensis]
Sequence ID: TEA24525.1 Length: 298
Range 1: 1 to 220

Score:421 bits(1081), Expect:2e-146,
Method:Compositional matrix adjust.,
Identities:215/239(90%), Positives:218/239(91%), Gaps:19/239(7%)

```
Query  1    MARTKQTARKSTGGKAPRKQLATKAARKSAPSTGGVKKPHRYRPGTVALREIRRYQKSTE  60
            MARTKQTARKSTGGKAPRKQLATKAARKSAP+TGGVKKPHRYRPGTVALREIRRYQKSTE
Sbjct  1    MARTKQTARKSTGGKAPRKQLATKAARKSAPATGGVKKPHRYRPGTVALREIRRYQKSTE  60

Query  61   LLIRKLPFQRLVREIAQDFKTDLRFQSAAIGALQEASEAYLVGLFEDTNLCAIHAKRVTI  120
            LLIRKLPFQRLVREIAQDFKTDLRFQS+A+ ALQEA EAYLVGLFEDTNLCAIHAKR
Sbjct  61   LLIRKLPFQRLVREIAQDFKTDLRFQSSAVMALQEACEAYLVGLFEDTNLCAIHAKR---  117

Query  121  MPKDIQLARRIRGERAMSGRGKGGKGLGKGGAKRHRKVLRDNIQGITKPAIRRLARRGGV  180
                             MSGRGKGGKGLGKGGAKRHRKVLRDNIQGITKPAIRRLARRGGV
Sbjct  118  -----------------MSGRGKGGKGLGKGGAKRHRKVLRDNIQGITKPAIRRLARRGGV  161

Query  181  KRISGLIYEETRGVLKVFLENVIRDAVTYTEHAKRKTVTAMDVVYALKRQGRTLYGFGG  239
            KRISGLIYEETRGVLKVFLENVIRDAVTYTEHAKRKTVTAMDVVYALKRQGRTLYGFGG
Sbjct  162  KRISGLIYEETRGVLKVFLENVIRDAVTYTEHAKRKTVTAMDVVYALKRQGRTLYGFGG  220
```

Range 2: 220 to 298

Score:131 bits(329), Expect:7e-33,
Method:Compositional matrix adjust.,
Identities:74/124(60%), Positives:77/124(62%), Gaps:45/124(36%)

```
Query  13   GGKAPRKQLATKAARKSAPSTGGVKKPHRYRPGTVALREIRRYQKSTELLIRKLPFQRLV  72
            GGKAPRKQLATKAARKSAP+TGG
Sbjct  220  GGKAPRKQLATKAARKSAPATGG-------------------------------------  242

Query  73   REIAQDFKTDLRFQSAAIGALQEASEAYLVGLFEDTNLCAIHAKRVTIMPKDIQLARRIR  132
                    TDLRFQS+A+ ALQEA EAYLVGLFEDTNLCAIHAKRVTIMPKDIQLARRIR
Sbjct  243  --------TDLRFQSSAVMALQEACEAYLVGLFEDTNLCAIHAKRVTIMPKDIQLARRIR  294

Query  133  GERA  136
            GERA
Sbjct  295  GERA  298
```

>uncharacterized protein zgc:163040 [Thalassophryne amazonica]
Sequence ID: XP_034044589.1 Length: 362
Range 1: 140 to 362

Score:423 bits(1087), Expect:2e-146,
Method:Compositional matrix adjust.,

```
Identities:217/239(91%), Positives:220/239(92%), Gaps:16/239(6%)

Query  1    MARTKQTARKSTGGKAPRKQLATKAARKSAPSTGGVKKPHRYRPGTVALREIRRYQKSTE  60
            MARTKQTARKSTGGKAPRKQLATKAARKSAP+TGGVKKPHRYRPGTVALREIRRYQKSTE
Sbjct  140  MARTKQTARKSTGGKAPRKQLATKAARKSAPATGGVKKPHRYRPGTVALREIRRYQKSTE  199

Query  61   LLIRKLPFQRLVREIAQDFKTDLRFQSAAIGALQEASEAYLVGLFEDTNLCAIHAKRVTI  120
            LLIRKLPFQRLVREIAQDFKTDLRFQS+A+ ALQEASEAYLVGLFEDTNLCAIHAKR
Sbjct  200  LLIRKLPFQRLVREIAQDFKTDLRFQSSAVMALQEASEAYLVGLFEDTNLCAIHAKR---  256

Query  121  MPKDIQLARRIRGERAMSGRGKGGKGLGKGGAKRHRKVLRDNIQGITKPAIRRLARRGGV  180
                          R MSGRGKGGKGLGKGGAKRHRKVLRDNIQGITKPAIRRLARRGGV
Sbjct  257  -------------RRNMSGRGKGGKGLGKGGAKRHRKVLRDNIQGITKPAIRRLARRGGV  303

Query  181  KRISGLIYEETRGVLKVFLENVIRDAVTYTEHAKRKTVTAMDVVYALKRQGRTLYGFGG  239
            KRISGLIYEETRGVLKVFLENVIRDAVTYTEHAKRKTVTAMDVVYALKRQGRTLYGFGG
Sbjct  304  KRISGLIYEETRGVLKVFLENVIRDAVTYTEHAKRKTVTAMDVVYALKRQGRTLYGFGG  362



Range 2: 1 to 102

Score:204 bits(519), Expect:2e-60,
Method:Compositional matrix adjust.,
Identities:102/102(100%), Positives:102/102(100%), Gaps:0/102(0%)

Query  137  MSGRGKGGKGLGKGGAKRHRKVLRDNIQGITKPAIRRLARRGGVKRISGLIYEETRGVLK  196
            MSGRGKGGKGLGKGGAKRHRKVLRDNIQGITKPAIRRLARRGGVKRISGLIYEETRGVLK
Sbjct  1    MSGRGKGGKGLGKGGAKRHRKVLRDNIQGITKPAIRRLARRGGVKRISGLIYEETRGVLK  60

Query  197  VFLENVIRDAVTYTEHAKRKTVTAMDVVYALKRQGRTLYGFG  238
            VFLENVIRDAVTYTEHAKRKTVTAMDVVYALKRQGRTLYGFG
Sbjct  61   VFLENVIRDAVTYTEHAKRKTVTAMDVVYALKRQGRTLYGFG  102



>uncharacterized protein LOC109252670 [Panthera pardus]
Sequence ID: XP_053761298.1 Length: 475
Range 1: 1 to 239

Score:427 bits(1097), Expect:3e-146,
Method:Compositional matrix adjust.,
Identities:226/239(95%), Positives:231/239(96%), Gaps:1/239(0%)

Query  1    MARTKQTARKSTGGKAPRKQLATKAARKSAPSTGGVKKPHRYRPGTVALREIRRYQKSTE  60
            MARTKQTARKSTGGKAPRKQLATKAARKSAP+TGGVKKPHRYRPGTVALREIRRYQKSTE
Sbjct  1    MARTKQTARKSTGGKAPRKQLATKAARKSAPATGGVKKPHRYRPGTVALREIRRYQKSTE  60

Query  61   LLIRKLPFQRLVREIAQDFKTDLRFQSAAIGALQEASEAYLVGLFEDTNLCAIHAKRVTI  120
            LLIRKLPFQRLVREIAQDFKTDLRFQS+A+ ALQEA EAYLVGLFEDTNLCAIHAKRVTI
Sbjct  61   LLIRKLPFQRLVREIAQDFKTDLRFQSSAVMALQEACEAYLVGLFEDTNLCAIHAKRVTI  120

Query  121  MPKDIQLARRIRGERA-MSGRGKGGKGLGKGGAKRHRKVLRDNIQGITKPAIRRLARRGG  179
            MPKDIQLARRIRGERA +    + GKGLGKGGAKRHRKVLRDNIQGITKPAIRRLARRGG
Sbjct  121  MPKDIQLARRIRGERADLMPDDECGKGLGKGGAKRHRKVLRDNIQGITKPAIRRLARRGG  180

Query  180  VKRISGLIYEETRGVLKVFLENVIRDAVTYTEHAKRKTVTAMDVVYALKRQGRTLYGFG  238
            VKRISGLIYEETRGVLKVFLENVIRDAVTYTEHAKRKTVTAMDVVYALKRQGRTLYGFG
Sbjct  181  VKRISGLIYEETRGVLKVFLENVIRDAVTYTEHAKRKTVTAMDVVYALKRQGRTLYGFG  239
```

```
Range 2: 340 to 475

Score:266 bits(680), Expect:3e-83,
Method:Compositional matrix adjust.,
Identities:131/136(96%), Positives:134/136(98%), Gaps:0/136(0%)

Query  1    MARTKQTARKSTGGKAPRKQLATKAARKSAPSTGGVKKPHRYRPGTVALREIRRYQKSTE  60
            MARTKQTARKSTGGKAPRKQLATKAARKSAP+TGGVKKPHRYRPGTVALREIRRYQKSTE
Sbjct  340  MARTKQTARKSTGGKAPRKQLATKAARKSAPATGGVKKPHRYRPGTVALREIRRYQKSTE  399

Query  61   LLIRKLPFQRLVREIAQDFKTDLRFQSAAIGALQEASEAYLVGLFEDTNLCAIHAKRVTI  120
            LLIRKLPFQRLVREIAQDFKTDLRFQS+A+ ALQEA EAYLVGLFEDTNLCAIHAKRVTI
Sbjct  400  LLIRKLPFQRLVREIAQDFKTDLRFQSSAVMALQEACEAYLVGLFEDTNLCAIHAKRVTI  459

Query  121  MPKDIQLARRIRGERA  136
            MPKDIQLARRIRGERA
Sbjct  460  MPKDIQLARRIRGERA  475



>hypothetical protein DBR06_SOUSAS20010018 [Sousa chinensis]
Sequence ID: TEA21837.1 Length: 346
Range 1: 122 to 346

Score:422 bits(1084), Expect:3e-146,
Method:Compositional matrix adjust.,
Identities:215/239(90%), Positives:221/239(92%), Gaps:14/239(5%)

Query  1    MARTKQTARKSTGGKAPRKQLATKAARKSAPSTGGVKKPHRYRPGTVALREIRRYQKSTE  60
            MARTKQTARKSTGGKAPRKQLATKAARKSAP+TGGVKKPHRYRPGTVALREIRRYQKSTE
Sbjct  122  MARTKQTARKSTGGKAPRKQLATKAARKSAPATGGVKKPHRYRPGTVALREIRRYQKSTE  181

Query  61   LLIRKLPFQRLVREIAQDFKTDLRFQSAAIGALQEASEAYLVGLFEDTNLCAIHAKRVTI  120
            LLIRKLPFQRLVREIAQDFKTDLRFQS+A+ ALQEA EAYLVGLFEDTNLCAIHAKR ++
Sbjct  182  LLIRKLPFQRLVREIAQDFKTDLRFQSSAVMALQEACEAYLVGLFEDTNLCAIHAKRKSL  241

Query  121  MPKDIQLARRIRGERAMSGRGKGGKGLGKGGAKRHRKVLRDNIQGITKPAIRRLARRGGV  180
            +                MSGRGKGGKGLGKGGAKRHRKVLRDNIQGITKPAIRRLARRGGV
Sbjct  242  L--------------VMSGRGKGGKGLGKGGAKRHRKVLRDNIQGITKPAIRRLARRGGV  287

Query  181  KRISGLIYEETRGVLKVFLENVIRDAVTYTEHAKRKTVTAMDVVYALKRQGRTLYGFGG  239
            KRISGLIYEETRGVLKVFLENVIRDAVTYTEHAKRKTVTAMDVVYALKRQGRTLYGFGG
Sbjct  288  KRISGLIYEETRGVLKVFLENVIRDAVTYTEHAKRKTVTAMDVVYALKRQGRTLYGFGG  346



Range 2: 1 to 103

Score:205 bits(522), Expect:4e-61,
Method:Compositional matrix adjust.,
Identities:103/103(100%), Positives:103/103(100%), Gaps:0/103(0%)

Query  137  MSGRGKGGKGLGKGGAKRHRKVLRDNIQGITKPAIRRLARRGGVKRISGLIYEETRGVLK  196
            MSGRGKGGKGLGKGGAKRHRKVLRDNIQGITKPAIRRLARRGGVKRISGLIYEETRGVLK
Sbjct  1    MSGRGKGGKGLGKGGAKRHRKVLRDNIQGITKPAIRRLARRGGVKRISGLIYEETRGVLK  60

Query  197  VFLENVIRDAVTYTEHAKRKTVTAMDVVYALKRQGRTLYGFGG  239
            VFLENVIRDAVTYTEHAKRKTVTAMDVVYALKRQGRTLYGFGG
Sbjct  61   VFLENVIRDAVTYTEHAKRKTVTAMDVVYALKRQGRTLYGFGG  103
```

```
>LOW QUALITY PROTEIN: uncharacterized protein LOC132316938 [Gavia stellata]
Sequence ID: XP_059672939.1 Length: 332
Range 1: 107 to 332

Score:421 bits(1082), Expect:3e-146,
Method:Compositional matrix adjust.,
Identities:216/239(90%), Positives:219/239(91%), Gaps:13/239(5%)

Query  1    MARTKQTARKSTGGKAPRKQLATKAARKSAPSTGGVKKPHRYRPGTVALREIRRYQKSTE  60
            MARTKQTARKSTGGKAPRKQLATKAARKSAP+TGGVKKPHRYRPGTVALREIRRYQKSTE
Sbjct  107  MARTKQTARKSTGGKAPRKQLATKAARKSAPATGGVKKPHRYRPGTVALREIRRYQKSTE  166

Query  61   LLIRKLPFQRLVREIAQDFKTDLRFQSAAIGALQEASEAYLVGLFEDTNLCAIHAKRVTI  120
            LLIRKLPFQRLVREIAQDFKTDLRFQS+A+ ALQEASEAYLVGLFEDTNLCAIHAKR
Sbjct  167  LLIRKLPFQRLVREIAQDFKTDLRFQSSAVMALQEASEAYLVGLFEDTNLCAIHAKRAA-  225

Query  121  MPKDIQLARRIRGERAMSGRGKGGKGLGKGGAKRHRKVLRDNIQGITKPAIRRLARRGGV  180
                             MSGRGKGGKGLGKGGAKRHRKVLRDNIQGITKPAIRRLARRGGV
Sbjct  226  ------------DSGTMSGRGKGGKGLGKGGAKRHRKVLRDNIQGITKPAIRRLARRGGV  273

Query  181  KRISGLIYEETRGVLKVFLENVIRDAVTYTEHAKRKTVTAMDVVYALKRQGRTLYGFGG  239
            KRISGLIYEETRGVLKVFLENVIRDAVTYTEHAKRKTVTAMDVVYALKRQGRTLYGFGG
Sbjct  274  KRISGLIYEETRGVLKVFLENVIRDAVTYTEHAKRKTVTAMDVVYALKRQGRTLYGFGG  332


Range 2: 1 to 103

Score:206 bits(524), Expect:2e-61,
Method:Compositional matrix adjust.,
Identities:103/103(100%), Positives:103/103(100%), Gaps:0/103(0%)

Query  137  MSGRGKGGKGLGKGGAKRHRKVLRDNIQGITKPAIRRLARRGGVKRISGLIYEETRGVLK  196
            MSGRGKGGKGLGKGGAKRHRKVLRDNIQGITKPAIRRLARRGGVKRISGLIYEETRGVLK
Sbjct  1    MSGRGKGGKGLGKGGAKRHRKVLRDNIQGITKPAIRRLARRGGVKRISGLIYEETRGVLK  60

Query  197  VFLENVIRDAVTYTEHAKRKTVTAMDVVYALKRQGRTLYGFGG  239
            VFLENVIRDAVTYTEHAKRKTVTAMDVVYALKRQGRTLYGFGG
Sbjct  61   VFLENVIRDAVTYTEHAKRKTVTAMDVVYALKRQGRTLYGFGG  103


>uncharacterized protein LOC135085844 [Ostrinia nubilalis]
Sequence ID: XP_063836701.1 Length: 326
Range 1: 107 to 326

Score:420 bits(1079), Expect:8e-146,
Method:Compositional matrix adjust.,
Identities:215/239(90%), Positives:219/239(91%), Gaps:19/239(7%)

Query  1    MARTKQTARKSTGGKAPRKQLATKAARKSAPSTGGVKKPHRYRPGTVALREIRRYQKSTE  60
            MARTKQTARKSTGGKAPRKQLATKAARKSAP+TGGVKKPHRYRPGTVALREIRRYQKSTE
Sbjct  107  MARTKQTARKSTGGKAPRKQLATKAARKSAPATGGVKKPHRYRPGTVALREIRRYQKSTE  166

Query  61   LLIRKLPFQRLVREIAQDFKTDLRFQSAAIGALQEASEAYLVGLFEDTNLCAIHAKRVTI  120
            LLIRKLPFQRLVREIAQDFKTDLRFQS+A+ ALQEASEAYLVGLFEDTNLCAIHAKR
Sbjct  167  LLIRKLPFQRLVREIAQDFKTDLRFQSSAVMALQEASEAYLVGLFEDTNLCAIHAKR---  223
```

```
Query  121  MPKDIQLARRIRGERAMSGRGKGGKGLGKGGAKRHRKVLRDNIQGITKPAIRRLARRGGV  180
                            M+GRGKGGKGLGKGGAKRHRKVLRDNIQGITKPAIRRLARRGGV
Sbjct  224  ----------------MTGRGKGGKGLGKGGAKRHRKVLRDNIQGITKPAIRRLARRGGV  267

Query  181  KRISGLIYEETRGVLKVFLENVIRDAVTYTEHAKRKTVTAMDVVYALKRQGRTLYGFGG  239
            KRISGLIYEETRGVLKVFLENVIRDAVTYTEHAKRKTVTAMDVVYALKRQGRTLYGFGG
Sbjct  268  KRISGLIYEETRGVLKVFLENVIRDAVTYTEHAKRKTVTAMDVVYALKRQGRTLYGFGG  326
```

Range 2: 1 to 99

Score:196 bits(497), Expect:2e-57,
Method:Compositional matrix adjust.,
Identities:98/99(99%), Positives:99/99(100%), Gaps:0/99(0%)

```
Query  137  MSGRGKGGKGLGKGGAKRHRKVLRDNIQGITKPAIRRLARRGGVKRISGLIYEETRGVLK  196
            M+GRGKGGKGLGKGGAKRHRKVLRDNIQGITKPAIRRLARRGGVKRISGLIYEETRGVLK
Sbjct  1    MTGRGKGGKGLGKGGAKRHRKVLRDNIQGITKPAIRRLARRGGVKRISGLIYEETRGVLK  60

Query  197  VFLENVIRDAVTYTEHAKRKTVTAMDVVYALKRQGRTLY  235
            VFLENVIRDAVTYTEHAKRKTVTAMDVVYALKRQGRTLY
Sbjct  61   VFLENVIRDAVTYTEHAKRKTVTAMDVVYALKRQGRTLY  99
```

>uncharacterized protein LOC129038393 [Pongo pygmaeus]
Sequence ID: XP_054347323.1 Length: 331
Range 1: 112 to 331

Score:419 bits(1078), Expect:1e-145,
Method:Compositional matrix adjust.,
Identities:215/239(90%), Positives:218/239(91%), Gaps:19/239(7%)

```
Query  1    MARTKQTARKSTGGKAPRKQLATKAARKSAPSTGGVKKPHRYRPGTVALREIRRYQKSTE  60
            MARTKQTARKSTGGKAPRKQLATKAARKSAP+TGGVKKPHRYRPGTVALREIRRYQKSTE
Sbjct  112  MARTKQTARKSTGGKAPRKQLATKAARKSAPATGGVKKPHRYRPGTVALREIRRYQKSTE  171

Query  61   LLIRKLPFQRLVREIAQDFKTDLRFQSAAIGALQEASEAYLVGLFEDTNLCAIHAKRVTI  120
            LLIRKLPFQRLVREIAQDFKTDLRFQS+A+ ALQEA EAYLVGLFEDTNLCAIHAKR
Sbjct  172  LLIRKLPFQRLVREIAQDFKTDLRFQSSAVMALQEACEAYLVGLFEDTNLCAIHAKR---  228

Query  121  MPKDIQLARRIRGERAMSGRGKGGKGLGKGGAKRHRKVLRDNIQGITKPAIRRLARRGGV  180
                            MSGRGKGGKGLGKGGAKRHRKVLRDNIQGITKPAIRRLARRGGV
Sbjct  229  ----------------MSGRGKGGKGLGKGGAKRHRKVLRDNIQGITKPAIRRLARRGGV  272

Query  181  KRISGLIYEETRGVLKVFLENVIRDAVTYTEHAKRKTVTAMDVVYALKRQGRTLYGFGG  239
            KRISGLIYEETRGVLKVFLENVIRDAVTYTEHAKRKTVTAMDVVYALKRQGRTLYGFGG
Sbjct  273  KRISGLIYEETRGVLKVFLENVIRDAVTYTEHAKRKTVTAMDVVYALKRQGRTLYGFGG  331
```

Range 2: 1 to 98

Score:134 bits(337), Expect:1e-33,
Method:Compositional matrix adjust.,
Identities:87/98(89%), Positives:87/98(88%), Gaps:0/98(0%)

```
Query  137  MSGRGKGGKGLGKGGAKRHRKVLRDNIQGITKPAIRRLARRGGVKRISGLIYEETRGVLK  196
```

```
            MSGRGKGGKGLGKGGAK HRKVL DNIQGITKP IRRLAR GGVKRISGLIYEETR V K
Sbjct  1    MSGRGKGGKGLGKGGAKCHRKVLSDNIQGITKPTIRRLARHGGVKRISGLIYEETRWVFK  60

Query  197  VFLENVIRDAVTYTEHAKRKTVTAMDVVYALKRQGRTL  234
            VFLENVI DAVT TEHAKRKTVTAM VV  LKRQGRTL
Sbjct  61   VFLENVIWDAVTNTEHAKRKTVTAMAVVCVLKRQGRTL  98




>PREDICTED: histone H3.1-like isoform X1 [Lipotes vexillifer]
Sequence ID: XP_007471299.1 Length: 332
Range 1: 111 to 332

Score:419 bits(1077), Expect:2e-145,
Method:Compositional matrix adjust.,
Identities:215/239(90%), Positives:218/239(91%), Gaps:17/239(7%)

Query  1    MARTKQTARKSTGGKAPRKQLATKAARKSAPSTGGVKKPHRYRPGTVALREIRRYQKSTE  60
            MARTKQTARKSTGGKAPRKQLATKAARKSAP+TGGVKKPHRYRPGTVALREIRRYQKSTE
Sbjct  111  MARTKQTARKSTGGKAPRKQLATKAARKSAPATGGVKKPHRYRPGTVALREIRRYQKSTE  170

Query  61   LLIRKLPFQRLVREIAQDFKTDLRFQSAAIGALQEASEAYLVGLFEDTNLCAIHAKRVTI  120
            LLIRKLPFQRLVREIAQDFKTDLRFQS+A+ ALQEA EAYLVGLFEDTNLCAIHAKR
Sbjct  171  LLIRKLPFQRLVREIAQDFKTDLRFQSSAVMALQEACEAYLVGLFEDTNLCAIHAKRAI-  229

Query  121  MPKDIQLARRIRGERAMSGRGKGGKGLGKGGAKRHRKVLRDNIQGITKPAIRRLARRGGV  180
                            MSGRGKGGKGLGKGGAKRHRKVLRDNIQGITKPAIRRLARRGGV
Sbjct  230  ----------------MSGRGKGGKGLGKGGAKRHRKVLRDNIQGITKPAIRRLARRGGV  273

Query  181  KRISGLIYEETRGVLKVFLENVIRDAVTYTEHAKRKTVTAMDVVYALKRQGRTLYGFGG  239
            KRISGLIYEETRGVLKVFLENVIRDAVTYTEHAKRKTVTAMDVVYALKRQGRTLYGFGG
Sbjct  274  KRISGLIYEETRGVLKVFLENVIRDAVTYTEHAKRKTVTAMDVVYALKRQGRTLYGFGG  332



Range 2: 1 to 96

Score:188 bits(478), Expect:1e-54,
Method:Compositional matrix adjust.,
Identities:95/96(99%), Positives:96/96(100%), Gaps:0/96(0%)

Query  137  MSGRGKGGKGLGKGGAKRHRKVLRDNIQGITKPAIRRLARRGGVKRISGLIYEETRGVLK  196
            MSGRGKGGKGLGKGGAKRHRKVLRDNIQGITKPAIRRLARRGGVKRISGLIYEETRGVLK
Sbjct  1    MSGRGKGGKGLGKGGAKRHRKVLRDNIQGITKPAIRRLARRGGVKRISGLIYEETRGVLK  60

Query  197  VFLENVIRDAVTYTEHAKRKTVTAMDVVYALKRQGR  232
            VFLENVIRDAVTYTEHAKRKTVTAMDVVYALKRQG+
Sbjct  61   VFLENVIRDAVTYTEHAKRKTVTAMDVVYALKRQGQ  96




>uncharacterized protein LOC122502212 [Leptopilina heterotoma]
Sequence ID: XP_043468085.1 Length: 271
Range 1: 54 to 271

Score:417 bits(1071), Expect:2e-145,
Method:Compositional matrix adjust.,
Identities:213/239(89%), Positives:217/239(90%), Gaps:21/239(8%)

Query  1    MARTKQTARKSTGGKAPRKQLATKAARKSAPSTGGVKKPHRYRPGTVALREIRRYQKSTE  60
```

```
            MARTKQTARKSTGGKAPRKQLATKAARKSAP+TGGVKKPHRYRPGTVALREIRRYQKSTE
Sbjct  54   MARTKQTARKSTGGKAPRKQLATKAARKSAPATGGVKKPHRYRPGTVALREIRRYQKSTE  113

Query  61   LLIRKLPFQRLVREIAQDFKTDLRFQSAAIGALQEASEAYLVGLFEDTNLCAIHAKRVTI  120
            LLIRKLPFQRLVREIAQDFKTDLRFQS+A+ ALQEASEAYLVGLFEDTNLCAIH
Sbjct  114  LLIRKLPFQRLVREIAQDFKTDLRFQSSAVMALQEASEAYLVGLFEDTNLCAIH------  167

Query  121  MPKDIQLARRIRGERAMSGRGKGGKGLGKGGAKRHRKVLRDNIQGITKPAIRRLARRGGV  180
                           AM+GRGKGGKGLGKGGAKRHRKVLRDNIQGITKPAIRRLARRGGV
Sbjct  168  ---------------AMTGRGKGGKGLGKGGAKRHRKVLRDNIQGITKPAIRRLARRGGV  212

Query  181  KRISGLIYEETRGVLKVFLENVIRDAVTYTEHAKRKTVTAMDVVYALKRQGRTLYGFGG  239
            KRISGLIYEETRGVLKVFLENVIRDAVTYTEHAKRKTVTAMDVVYALKRQGRTLYGFGG
Sbjct  213  KRISGLIYEETRGVLKVFLENVIRDAVTYTEHAKRKTVTAMDVVYALKRQGRTLYGFGG  271
```

>uncharacterized protein LOC119895545 [Micropterus salmoides]
Sequence ID: XP_038564651.1 Length: 395
Range 1: 176 to 395

Score:421 bits(1083), Expect:3e-145,
Method:Compositional matrix adjust.,
Identities:216/239(90%), Positives:219/239(91%), Gaps:19/239(7%)

```
Query  1    MARTKQTARKSTGGKAPRKQLATKAARKSAPSTGGVKKPHRYRPGTVALREIRRYQKSTE  60
            MARTKQTARKSTGGKAPRKQLATKAARKSAP+TGGVKKPHRYRPGTVALREIRRYQKSTE
Sbjct  176  MARTKQTARKSTGGKAPRKQLATKAARKSAPATGGVKKPHRYRPGTVALREIRRYQKSTE  235

Query  61   LLIRKLPFQRLVREIAQDFKTDLRFQSAAIGALQEASEAYLVGLFEDTNLCAIHAKRVTI  120
            LLIRKLPFQRLVREIAQDFKTDLRFQS+A+ ALQEASEAYLVGLFEDTNLCAIHAKR
Sbjct  236  LLIRKLPFQRLVREIAQDFKTDLRFQSSAVMALQEASEAYLVGLFEDTNLCAIHAKR---  292

Query  121  MPKDIQLARRIRGERAMSGRGKGGKGLGKGGAKRHRKVLRDNIQGITKPAIRRLARRGGV  180
                            MSGRGKGGKGLGKGGAKRHRKVLRDNIQGITKPAIRRLARRGGV
Sbjct  293  ----------------MSGRGKGGKGLGKGGAKRHRKVLRDNIQGITKPAIRRLARRGGV  336

Query  181  KRISGLIYEETRGVLKVFLENVIRDAVTYTEHAKRKTVTAMDVVYALKRQGRTLYGFGG  239
            KRISGLIYEETRGVLKVFLENVIRDAVTYTEHAKRKTVTAMDVVYALKRQGRTLYGFGG
Sbjct  337  KRISGLIYEETRGVLKVFLENVIRDAVTYTEHAKRKTVTAMDVVYALKRQGRTLYGFGG  395
```

Range 2: 1 to 103

Score:206 bits(524), Expect:9e-61,
Method:Compositional matrix adjust.,
Identities:103/103(100%), Positives:103/103(100%), Gaps:0/103(0%)

```
Query  137  MSGRGKGGKGLGKGGAKRHRKVLRDNIQGITKPAIRRLARRGGVKRISGLIYEETRGVLK  196
            MSGRGKGGKGLGKGGAKRHRKVLRDNIQGITKPAIRRLARRGGVKRISGLIYEETRGVLK
Sbjct  1    MSGRGKGGKGLGKGGAKRHRKVLRDNIQGITKPAIRRLARRGGVKRISGLIYEETRGVLK  60

Query  197  VFLENVIRDAVTYTEHAKRKTVTAMDVVYALKRQGRTLYGFGG  239
            VFLENVIRDAVTYTEHAKRKTVTAMDVVYALKRQGRTLYGFGG
Sbjct  61   VFLENVIRDAVTYTEHAKRKTVTAMDVVYALKRQGRTLYGFGG  103
```

>uncharacterized protein LOC128157070 [Magallana angulata]

```
Sequence ID: XP_052675387.1 Length: 722
Range 1: 147 to 365

Score:432 bits(1112), Expect:3e-145,
Method:Compositional matrix adjust.,
Identities:215/232(93%), Positives:218/232(93%), Gaps:13/232(5%)

Query  1    MARTKQTARKSTGGKAPRKQLATKAARKSAPSTGGVKKPHRYRPGTVALREIRRYQKSTE  60
            MARTKQTARKSTGGKAPRKQLATKAARKSAP+TGGVKKPHRYRPGTVALREIRRYQKSTE
Sbjct  147  MARTKQTARKSTGGKAPRKQLATKAARKSAPATGGVKKPHRYRPGTVALREIRRYQKSTE  206

Query  61   LLIRKLPFQRLVREIAQDFKTDLRFQSAAIGALQEASEAYLVGLFEDTNLCAIHAKRVTI  120
            LLIRKLPFQRLVREIAQDFKTDLRFQS+A+ ALQEASEAYLVGLFEDTNLCAIHAKRVTI
Sbjct  207  LLIRKLPFQRLVREIAQDFKTDLRFQSSAVMALQEASEAYLVGLFEDTNLCAIHAKRVTI  266

Query  121  MPKDIQLARRIRGERAMSGRGKGGKGLGKGGAKRHRKVLRDNIQGITKPAIRRLARRGGV  180
            MPKDIQLARR             GKGLGKGGAKRHRKVLRDNIQGITKPAIRRLARRGGV
Sbjct  267  MPKDIQLARR-------------GKGLGKGGAKRHRKVLRDNIQGITKPAIRRLARRGGV  313

Query  181  KRISGLIYEETRGVLKVFLENVIRDAVTYTEHAKRKTVTAMDVVYALKRQGR  232
            KRISGLIYEETRGVLKVFLENVIRDAVTYTEHAKRKTVTAMDVVYALKRQGR
Sbjct  314  KRISGLIYEETRGVLKVFLENVIRDAVTYTEHAKRKTVTAMDVVYALKRQGR  365



>histone H3-like [Microplitis mediator]
Sequence ID: XP_057322000.1 Length: 218
Range 1: 1 to 218

Score:414 bits(1063), Expect:5e-145,
Method:Compositional matrix adjust.,
Identities:212/239(89%), Positives:216/239(90%), Gaps:21/239(8%)

Query  1    MARTKQTARKSTGGKAPRKQLATKAARKSAPSTGGVKKPHRYRPGTVALREIRRYQKSTE  60
            MARTKQTARKSTGGKAPRKQLATKAARKSAP+TGGVKKPHRYRPGTVALREIRRYQKSTE
Sbjct  1    MARTKQTARKSTGGKAPRKQLATKAARKSAPATGGVKKPHRYRPGTVALREIRRYQKSTE  60

Query  61   LLIRKLPFQRLVREIAQDFKTDLRFQSAAIGALQEASEAYLVGLFEDTNLCAIHAKRVTI  120
            LLIRKLPFQRLVREIAQDFKTDLRFQS+ + ALQEASEAYLVGLFEDTNLCAIH
Sbjct  61   LLIRKLPFQRLVREIAQDFKTDLRFQSSGVMALQEASEAYLVGLFEDTNLCAIH------  114

Query  121  MPKDIQLARRIRGERAMSGRGKGGKGLGKGGAKRHRKVLRDNIQGITKPAIRRLARRGGV  180
                           AM+GRGKGGKGLGKGGAKRHRKVLRDNIQGITKPAIRRLARRGGV
Sbjct  115  ---------------AMTGRGKGGKGLGKGGAKRHRKVLRDNIQGITKPAIRRLARRGGV  159

Query  181  KRISGLIYEETRGVLKVFLENVIRDAVTYTEHAKRKTVTAMDVVYALKRQGRTLYGFGG  239
            KRISGLIYEETRGVLKVFLENVIRDAVTYTEHAKRKTVTAMDVVYALKRQGRTLYGFGG
Sbjct  160  KRISGLIYEETRGVLKVFLENVIRDAVTYTEHAKRKTVTAMDVVYALKRQGRTLYGFGG  218



>histone H3.1-like [Lontra canadensis]
Sequence ID: XP_032717653.1 Length: 219
Range 1: 1 to 219

Score:413 bits(1062), Expect:8e-145,
Method:Compositional matrix adjust.,
Identities:212/239(89%), Positives:215/239(89%), Gaps:20/239(8%)

Query  1    MARTKQTARKSTGGKAPRKQLATKAARKSAPSTGGVKKPHRYRPGTVALREIRRYQKSTE  60
            MARTKQTARKSTGGKAPRKQLATKAARKSAP+TGGVKKPHRYRPGTVALREIRRYQKSTE
```

```
Sbjct  1    MARTKQTARKSTGGKAPRKQLATKAARKSAPATGGVKKPHRYRPGTVALREIRRYQKSTE  60

Query  61   LLIRKLPFQRLVREIAQDFKTDLRFQSAAIGALQEASEAYLVGLFEDTNLCAIHAKRVTI  120
            LLIRKLPFQRLVREIAQDFKTDLRFQS+A+ ALQEA EAYLVGLFEDTNLCAIH
Sbjct  61   LLIRKLPFQRLVREIAQDFKTDLRFQSSAVMALQEACEAYLVGLFEDTNLCAIHVD----  116

Query  121  MPKDIQLARRIRGERAMSGRGKGGKGLGKGGAKRHRKVLRDNIQGITKPAIRRLARRGGV  180
                            MSGRGKGGKGLGKGGAKRHRKVLRDNIQGITKPAIRRLARRGGV
Sbjct  117  ----------------MSGRGKGGKGLGKGGAKRHRKVLRDNIQGITKPAIRRLARRGGV  160

Query  181  KRISGLIYEETRGVLKVFLENVIRDAVTYTEHAKRKTVTAMDVVYALKRQGRTLYGFGG  239
            KRISGLIYEETRGVLKVFLENVIRDAVTYTEHAKRKTVTAMDVVYALKRQGRTLYGFGG
Sbjct  161  KRISGLIYEETRGVLKVFLENVIRDAVTYTEHAKRKTVTAMDVVYALKRQGRTLYGFGG  219
```

>uncharacterized protein LOC132474238 [Gadus macrocephalus]
Sequence ID: XP_059930793.1 Length: 613
Range 1: 392 to 613

Score:428 bits(1100), Expect:9e-145,
Method:Compositional matrix adjust.,
Identities:216/239(90%), Positives:219/239(91%), Gaps:17/239(7%)

```
Query  1    MARTKQTARKSTGGKAPRKQLATKAARKSAPSTGGVKKPHRYRPGTVALREIRRYQKSTE  60
            MARTKQTARKSTGGKAPRKQLATKAARKSAP+TGGVKKPHRYRPGTVALREIRRYQKSTE
Sbjct  392  MARTKQTARKSTGGKAPRKQLATKAARKSAPATGGVKKPHRYRPGTVALREIRRYQKSTE  451

Query  61   LLIRKLPFQRLVREIAQDFKTDLRFQSAAIGALQEASEAYLVGLFEDTNLCAIHAKRVTI  120
            LLIRKLPFQRLVREIAQDFKTDLRFQS+A+ ALQEASEAYLVGLFEDTNLCAIHAKR
Sbjct  452  LLIRKLPFQRLVREIAQDFKTDLRFQSSAVMALQEASEAYLVGLFEDTNLCAIHAKRSI-  510

Query  121  MPKDIQLARRIRGERAMSGRGKGGKGLGKGGAKRHRKVLRDNIQGITKPAIRRLARRGGV  180
                            MSGRGKGGKGLGKGGAKRHRKVLRDNIQGITKPAIRRLARRGGV
Sbjct  511  ----------------MSGRGKGGKGLGKGGAKRHRKVLRDNIQGITKPAIRRLARRGGV  554

Query  181  KRISGLIYEETRGVLKVFLENVIRDAVTYTEHAKRKTVTAMDVVYALKRQGRTLYGFGG  239
            KRISGLIYEETRGVLKVFLENVIRDAVTYTEHAKRKTVTAMDVVYALKRQGRTLYGFGG
Sbjct  555  KRISGLIYEETRGVLKVFLENVIRDAVTYTEHAKRKTVTAMDVVYALKRQGRTLYGFGG  613
```

Range 2: 1 to 102

Score:208 bits(530), Expect:1e-59,
Method:Compositional matrix adjust.,
Identities:102/102(100%), Positives:102/102(100%), Gaps:0/102(0%)

```
Query  137  MSGRGKGGKGLGKGGAKRHRKVLRDNIQGITKPAIRRLARRGGVKRISGLIYEETRGVLK  196
            MSGRGKGGKGLGKGGAKRHRKVLRDNIQGITKPAIRRLARRGGVKRISGLIYEETRGVLK
Sbjct  1    MSGRGKGGKGLGKGGAKRHRKVLRDNIQGITKPAIRRLARRGGVKRISGLIYEETRGVLK  60

Query  197  VFLENVIRDAVTYTEHAKRKTVTAMDVVYALKRQGRTLYGFG  238
            VFLENVIRDAVTYTEHAKRKTVTAMDVVYALKRQGRTLYGFG
Sbjct  61   VFLENVIRDAVTYTEHAKRKTVTAMDVVYALKRQGRTLYGFG  102
```

>uncharacterized protein [Phyllobates terribilis]
Sequence ID: XP_073502315.1 Length: 437

Range 1: 218 to 437

Score:421 bits(1083), Expect:1e-144,
Method:Compositional matrix adjust.,
Identities:216/239(90%), Positives:219/239(91%), Gaps:19/239(7%)

```
Query  1    MARTKQTARKSTGGKAPRKQLATKAARKSAPSTGGVKKPHRYRPGTVALREIRRYQKSTE  60
            MARTKQTARKSTGGKAPRKQLATKAARKSAP+TGGVKKPHRYRPGTVALREIRRYQKSTE
Sbjct  218  MARTKQTARKSTGGKAPRKQLATKAARKSAPATGGVKKPHRYRPGTVALREIRRYQKSTE  277

Query  61   LLIRKLPFQRLVREIAQDFKTDLRFQSAAIGALQEASEAYLVGLFEDTNLCAIHAKRVTI  120
            LLIRKLPFQRLVREIAQDFKTDLRFQS+A+ ALQEASEAYLVGLFEDTNLCAIHAKR
Sbjct  278  LLIRKLPFQRLVREIAQDFKTDLRFQSSAVMALQEASEAYLVGLFEDTNLCAIHAKR---  334

Query  121  MPKDIQLARRIRGERAMSGRGKGGKGLGKGGAKRHRKVLRDNIQGITKPAIRRLARRGGV  180
                             MSGRGKGGKGLGKGGAKRHRKVLRDNIQGITKPAIRRLARRGGV
Sbjct  335  ----------------MSGRGKGGKGLGKGGAKRHRKVLRDNIQGITKPAIRRLARRGGV  378

Query  181  KRISGLIYEETRGVLKVFLENVIRDAVTYTEHAKRKTVTAMDVVYALKRQGRTLYGFGG  239
            KRISGLIYEETRGVLKVFLENVIRDAVTYTEHAKRKTVTAMDVVYALKRQGRTLYGFGG
Sbjct  379  KRISGLIYEETRGVLKVFLENVIRDAVTYTEHAKRKTVTAMDVVYALKRQGRTLYGFGG  437
```

Range 2: 1 to 102

Score:204 bits(519), Expect:1e-59,
Method:Compositional matrix adjust.,
Identities:102/102(100%), Positives:102/102(100%), Gaps:0/102(0%)

```
Query  137  MSGRGKGGKGLGKGGAKRHRKVLRDNIQGITKPAIRRLARRGGVKRISGLIYEETRGVLK  196
            MSGRGKGGKGLGKGGAKRHRKVLRDNIQGITKPAIRRLARRGGVKRISGLIYEETRGVLK
Sbjct  1    MSGRGKGGKGLGKGGAKRHRKVLRDNIQGITKPAIRRLARRGGVKRISGLIYEETRGVLK  60

Query  197  VFLENVIRDAVTYTEHAKRKTVTAMDVVYALKRQGRTLYGFG  238
            VFLENVIRDAVTYTEHAKRKTVTAMDVVYALKRQGRTLYGFG
Sbjct  61   VFLENVIRDAVTYTEHAKRKTVTAMDVVYALKRQGRTLYGFG  102
```

>uncharacterized protein LOC122544485 [Chiloscyllium plagiosum]
Sequence ID: XP_043539660.1 Length: 384
Range 1: 1 to 217

Score:419 bits(1077), Expect:1e-144,
Method:Compositional matrix adjust.,
Identities:211/223(95%), Positives:215/223(96%), Gaps:6/223(2%)

```
Query  1    MARTKQTARKSTGGKAPRKQLATKAARKSAPSTGGVKKPHRYRPGTVALREIRRYQKSTE  60
            MARTKQTA KSTGGKAPRKQLATKAARKSAP+TGGVKKPHRYRPGTVALREIRRYQKSTE
Sbjct  1    MARTKQTAHKSTGGKAPRKQLATKAARKSAPATGGVKKPHRYRPGTVALREIRRYQKSTE  60

Query  61   LLIRKLPFQRLVREIAQDFKTDLRFQSAAIGALQEASEAYLVGLFEDTNLCAIHAKRVTI  120
            LLIRKLPFQRLVREIAQDFKTDLRFQS+A+ ALQEASEAY+VGLFEDTNLCAIHAKRVTI
Sbjct  61   LLIRKLPFQRLVREIAQDFKTDLRFQSSAVMALQEASEAYMVGLFEDTNLCAIHAKRVTI  120

Query  121  MPKDIQLARRIRGERAMSGRGKGGKGLGKGGAKRHRKVLRDNIQGITKPAIRRLARRGGV  180
            MPKDIQLARRIRGE      GKGGKGLGKGGAKRHRKVLRDNIQGITKPAIRRLARRGGV
Sbjct  121  MPKDIQLARRIRGE------GKGGKGLGKGGAKRHRKVLRDNIQGITKPAIRRLARRGGV  174

Query  181  KRISGLIYEETRGVLKVFLENVIRDAVTYTEHAKRKTVTAMDV  223
```

```
            KRISGLIYEETRGVLKVFLENVIRDAVTYTEHAKRKTVTAMDV
Sbjct  175  KRISGLIYEETRGVLKVFLENVIRDAVTYTEHAKRKTVTAMDV  217
```

Range 2: 249 to 384

Score:268 bits(685), Expect:4e-85,
Method:Compositional matrix adjust.,
Identities:132/136(97%), Positives:135/136(99%), Gaps:0/136(0%)

```
Query  1    MARTKQTARKSTGGKAPRKQLATKAARKSAPSTGGVKKPHRYRPGTVALREIRRYQKSTE  60
            MARTKQTARKSTGGKAPRKQLATKAARKSAP+TGGVKKPHRYRPGTVALREIRRYQKSTE
Sbjct  249  MARTKQTARKSTGGKAPRKQLATKAARKSAPATGGVKKPHRYRPGTVALREIRRYQKSTE  308

Query  61   LLIRKLPFQRLVREIAQDFKTDLRFQSAAIGALQEASEAYLVGLFEDTNLCAIHAKRVTI  120
            LLIRKLPFQRLVREIAQDFKTDLRFQS+A+ ALQEASEAYLVGLFEDTNLCAIHAKRVTI
Sbjct  309  LLIRKLPFQRLVREIAQDFKTDLRFQSSAVMALQEASEAYLVGLFEDTNLCAIHAKRVTI  368

Query  121  MPKDIQLARRIRGERA  136
            MPKDIQLARRIRGERA
Sbjct  369  MPKDIQLARRIRGERA  384
```

>uncharacterized protein LOC135242606 [Anguilla rostrata]
Sequence ID: XP_064169827.1 Length: 323
Range 1: 106 to 323

Score:416 bits(1070), Expect:2e-144,
Method:Compositional matrix adjust.,
Identities:214/239(90%), Positives:217/239(90%), Gaps:21/239(8%)

```
Query  1    MARTKQTARKSTGGKAPRKQLATKAARKSAPSTGGVKKPHRYRPGTVALREIRRYQKSTE  60
            MARTKQTARKSTGGKAPRKQLATKAARKSAP+TGGVKKPHRYRPGTVALREIRRYQKSTE
Sbjct  106  MARTKQTARKSTGGKAPRKQLATKAARKSAPATGGVKKPHRYRPGTVALREIRRYQKSTE  165

Query  61   LLIRKLPFQRLVREIAQDFKTDLRFQSAAIGALQEASEAYLVGLFEDTNLCAIHAKRVTI  120
            LLIRKLPFQRLVREIAQDFKTDLRFQS+A+ ALQEASEAYLVGLFEDTNLCAIH
Sbjct  166  LLIRKLPFQRLVREIAQDFKTDLRFQSSAVMALQEASEAYLVGLFEDTNLCAIH------  219

Query  121  MPKDIQLARRIRGERAMSGRGKGGKGLGKGGAKRHRKVLRDNIQGITKPAIRRLARRGGV  180
                           AMSGRGKGGKGLGKGGAKRHRKVLRDNIQGITKPAIRRLARRGGV
Sbjct  220  ---------------AMSGRGKGGKGLGKGGAKRHRKVLRDNIQGITKPAIRRLARRGGV  264

Query  181  KRISGLIYEETRGVLKVFLENVIRDAVTYTEHAKRKTVTAMDVVYALKRQGRTLYGFGG  239
            KRISGLIYEETRGVLKVFLENVIRDAVTYTEHAKRKTVTAMDVVYALKRQGRTLYGFGG
Sbjct  265  KRISGLIYEETRGVLKVFLENVIRDAVTYTEHAKRKTVTAMDVVYALKRQGRTLYGFGG  323
```

Range 2: 1 to 103

Score:205 bits(522), Expect:2e-61,
Method:Compositional matrix adjust.,
Identities:103/103(100%), Positives:103/103(100%), Gaps:0/103(0%)

```
Query  137  MSGRGKGGKGLGKGGAKRHRKVLRDNIQGITKPAIRRLARRGGVKRISGLIYEETRGVLK  196
            MSGRGKGGKGLGKGGAKRHRKVLRDNIQGITKPAIRRLARRGGVKRISGLIYEETRGVLK
Sbjct  1    MSGRGKGGKGLGKGGAKRHRKVLRDNIQGITKPAIRRLARRGGVKRISGLIYEETRGVLK  60
```

```
Query  197   VFLENVIRDAVTYTEHAKRKTVTAMDVVYALKRQGRTLYGFGG  239
             VFLENVIRDAVTYTEHAKRKTVTAMDVVYALKRQGRTLYGFGG
Sbjct  61    VFLENVIRDAVTYTEHAKRKTVTAMDVVYALKRQGRTLYGFGG  103
```

>uncharacterized protein LOC117756542 [Hippoglossus hippoglossus]
Sequence ID: XP_034432975.1 Length: 581
Range 1: 360 to 581

Score:426 bits(1094), Expect:2e-144,
Method:Compositional matrix adjust.,
Identities:217/239(91%), Positives:220/239(92%), Gaps:17/239(7%)

```
Query  1     MARTKQTARKSTGGKAPRKQLATKAARKSAPSTGGVKKPHRYRPGTVALREIRRYQKSTE  60
             MARTKQTARKSTGGKAPRKQLATKAARKSAP+TGGVKKPHRYRPGTVALREIRRYQKSTE
Sbjct  360   MARTKQTARKSTGGKAPRKQLATKAARKSAPATGGVKKPHRYRPGTVALREIRRYQKSTE  419

Query  61    LLIRKLPFQRLVREIAQDFKTDLRFQSAAIGALQEASEAYLVGLFEDTNLCAIHAKRVTI  120
             LLIRKLPFQRLVREIAQDFKTDLRFQS+A+ ALQEASEAYLVGLFEDTNLCAIHAKR
Sbjct  420   LLIRKLPFQRLVREIAQDFKTDLRFQSSAVMALQEASEAYLVGLFEDTNLCAIHAKR---  476

Query  121   MPKDIQLARRIRGERAMSGRGKGGKGLGKGGAKRHRKVLRDNIQGITKPAIRRLARRGGV  180
                            R MSGRGKGGKGLGKGGAKRHRKVLRDNIQGITKPAIRRLARRGGV
Sbjct  477   --------------REMSGRGKGGKGLGKGGAKRHRKVLRDNIQGITKPAIRRLARRGGV  522

Query  181   KRISGLIYEETRGVLKVFLENVIRDAVTYTEHAKRKTVTAMDVVYALKRQGRTLYGFGG  239
             KRISGLIYEETRGVLKVFLENVIRDAVTYTEHAKRKTVTAMDVVYALKRQGRTLYGFGG
Sbjct  523   KRISGLIYEETRGVLKVFLENVIRDAVTYTEHAKRKTVTAMDVVYALKRQGRTLYGFGG  581
```

Range 2: 1 to 103

Score:207 bits(528), Expect:1e-59,
Method:Compositional matrix adjust.,
Identities:103/103(100%), Positives:103/103(100%), Gaps:0/103(0%)

```
Query  137   MSGRGKGGKGLGKGGAKRHRKVLRDNIQGITKPAIRRLARRGGVKRISGLIYEETRGVLK  196
             MSGRGKGGKGLGKGGAKRHRKVLRDNIQGITKPAIRRLARRGGVKRISGLIYEETRGVLK
Sbjct  1     MSGRGKGGKGLGKGGAKRHRKVLRDNIQGITKPAIRRLARRGGVKRISGLIYEETRGVLK  60

Query  197   VFLENVIRDAVTYTEHAKRKTVTAMDVVYALKRQGRTLYGFGG  239
             VFLENVIRDAVTYTEHAKRKTVTAMDVVYALKRQGRTLYGFGG
Sbjct  61    VFLENVIRDAVTYTEHAKRKTVTAMDVVYALKRQGRTLYGFGG  103
```

>neurofilament heavy polypeptide-like [Hippocampus comes]
Sequence ID: XP_019738120.1 Length: 658
Range 1: 439 to 658

Score:428 bits(1100), Expect:3e-144,
Method:Compositional matrix adjust.,
Identities:215/239(90%), Positives:219/239(91%), Gaps:19/239(7%)

```
Query  1     MARTKQTARKSTGGKAPRKQLATKAARKSAPSTGGVKKPHRYRPGTVALREIRRYQKSTE  60
             MARTKQTARKSTGGKAPRKQLATKAARKSAP+TGGVKKPHRYRPGTVALREIRRYQKSTE
Sbjct  439   MARTKQTARKSTGGKAPRKQLATKAARKSAPATGGVKKPHRYRPGTVALREIRRYQKSTE  498
```

```
Query  61   LLIRKLPFQRLVREIAQDFKTDLRFQSAAIGALQEASEAYLVGLFEDTNLCAIHAKRVTI  120
            LLIRKLPFQRLVREIAQDFKTDLRFQS+A+ ALQE+SEAYLVGLFEDTNLCAIHAKR
Sbjct  499  LLIRKLPFQRLVREIAQDFKTDLRFQSSAVMALQESSEAYLVGLFEDTNLCAIHAKR---  555

Query  121  MPKDIQLARRIRGERAMSGRGKGGKGLGKGGAKRHRKVLRDNIQGITKPAIRRLARRGGV  180
                            MSGRGKGGKGLGKGGAKRHRKVLRDNIQGITKPAIRRLARRGGV
Sbjct  556  ----------------MSGRGKGGKGLGKGGAKRHRKVLRDNIQGITKPAIRRLARRGGV  599

Query  181  KRISGLIYEETRGVLKVFLENVIRDAVTYTEHAKRKTVTAMDVVYALKRQGRTLYGFGG  239
            KRISGLIYEETRGVLKVFLENVIRDAVTYTEHAKRKTVTAMDVVYALKRQGRTLYGFGG
Sbjct  600  KRISGLIYEETRGVLKVFLENVIRDAVTYTEHAKRKTVTAMDVVYALKRQGRTLYGFGG  658
```

Range 2: 1 to 103

Score:211 bits(538), Expect:2e-60,
Method:Compositional matrix adjust.,
Identities:103/103(100%), Positives:103/103(100%), Gaps:0/103(0%)

```
Query  137  MSGRGKGGKGLGKGGAKRHRKVLRDNIQGITKPAIRRLARRGGVKRISGLIYEETRGVLK  196
            MSGRGKGGKGLGKGGAKRHRKVLRDNIQGITKPAIRRLARRGGVKRISGLIYEETRGVLK
Sbjct  1    MSGRGKGGKGLGKGGAKRHRKVLRDNIQGITKPAIRRLARRGGVKRISGLIYEETRGVLK  60

Query  197  VFLENVIRDAVTYTEHAKRKTVTAMDVVYALKRQGRTLYGFGG  239
            VFLENVIRDAVTYTEHAKRKTVTAMDVVYALKRQGRTLYGFGG
Sbjct  61   VFLENVIRDAVTYTEHAKRKTVTAMDVVYALKRQGRTLYGFGG  103
```

>histone H3.1, partial [Galemys pyrenaicus]
Sequence ID: KAG8511098.1 Length: 640
Range 1: 250 to 479

Score:427 bits(1098), Expect:3e-144,
Method:Compositional matrix adjust.,
Identities:217/230(94%), Positives:220/230(95%), Gaps:8/230(3%)

```
Query  1    MARTKQTARKSTGGKAPRKQLATKAARKSAPSTGGVKKPHRYRPGTVALREIRRYQKSTE  60
            MARTKQTARKSTGGKAPRKQLATKAARKSAP+TGGVKKPHRYRPGTVALREIRRYQKSTE
Sbjct  250  MARTKQTARKSTGGKAPRKQLATKAARKSAPATGGVKKPHRYRPGTVALREIRRYQKSTE  309

Query  61   LLIRKLPFQRLVREIAQDFKTDLRFQSAAIGALQEASEAYLVGLFEDTNLCAIHAKRVTI  120
            LLIRKLPFQRLVREIAQDFKTDLRFQS+A+ ALQEA EAYLVGLFEDTNLCAIHAKRVTI
Sbjct  310  LLIRKLPFQRLVREIAQDFKTDLRFQSSAVMALQEACEAYLVGLFEDTNLCAIHAKRVTI  369

Query  121  MPKDIQLARRIRGERA--------MSGRGKGGKGLGKGGAKRHRKVLRDNIQGITKPAIR  172
            MPKDIQLARRIRGERA        MSGRGKGGKGLGKGGAKRHRKVLRDNIQGITKPAIR
Sbjct  370  MPKDIQLARRIRGERASDVALYRWMSGRGKGGKGLGKGGAKRHRKVLRDNIQGITKPAIR  429

Query  173  RLARRGGVKRISGLIYEETRGVLKVFLENVIRDAVTYTEHAKRKTVTAMD  222
            RLARRGGVKRISGLIYEETRGVLKVFLENVIRDAVTYTEHAKRKTVTAMD
Sbjct  430  RLARRGGVKRISGLIYEETRGVLKVFLENVIRDAVTYTEHAKRKTVTAMD  479
```

Range 2: 505 to 640

Score:266 bits(679), Expect:3e-81,

Method:Compositional matrix adjust.,
Identities:131/136(96%), Positives:134/136(98%), Gaps:0/136(0%)

```
Query  1    MARTKQTARKSTGGKAPRKQLATKAARKSAPSTGGVKKPHRYRPGTVALREIRRYQKSTE  60
            MARTKQTARKSTGGKAPRKQLATKAARKSAP+TGGVKKPHRYRPGTVALREIRRYQKSTE
Sbjct  505  MARTKQTARKSTGGKAPRKQLATKAARKSAPATGGVKKPHRYRPGTVALREIRRYQKSTE  564

Query  61   LLIRKLPFQRLVREIAQDFKTDLRFQSAAIGALQEASEAYLVGLFEDTNLCAIHAKRVTI  120
            LLIRKLPFQRLVREIAQDFKTDLRFQS+A+ ALQEA EAYLVGLFEDTNLCAIHAKRVTI
Sbjct  565  LLIRKLPFQRLVREIAQDFKTDLRFQSSAVMALQEACEAYLVGLFEDTNLCAIHAKRVTI  624

Query  121  MPKDIQLARRIRGERA  136
            MPKDIQLARRIRGERA
Sbjct  625  MPKDIQLARRIRGERA  640
```

>uncharacterized protein LOC116589447 [Mustela erminea]
Sequence ID: XP_032197276.1 Length: 404
Range 1: 183 to 404

Score:418 bits(1075), Expect:5e-144,
Method:Compositional matrix adjust.,
Identities:215/239(90%), Positives:219/239(91%), Gaps:17/239(7%)

```
Query  1    MARTKQTARKSTGGKAPRKQLATKAARKSAPSTGGVKKPHRYRPGTVALREIRRYQKSTE  60
            MARTKQTARKSTGGKAPRKQLATKAARKSAP+TGGVKKPHRYRPGTVALREIRRYQKSTE
Sbjct  183  MARTKQTARKSTGGKAPRKQLATKAARKSAPATGGVKKPHRYRPGTVALREIRRYQKSTE  242

Query  61   LLIRKLPFQRLVREIAQDFKTDLRFQSAAIGALQEASEAYLVGLFEDTNLCAIHAKRVTI  120
            LLIRKLPFQRLVREIAQDFKTDLRFQS+A+ ALQEA EAYLVGLFEDTNLCAIHAKR+
Sbjct  243  LLIRKLPFQRLVREIAQDFKTDLRFQSSAVMALQEACEAYLVGLFEDTNLCAIHAKRLI-  301

Query  121  MPKDIQLARRIRGERAMSGRGKGGKGLGKGGAKRHRKVLRDNIQGITKPAIRRLARRGGV  180
                            MSGRGKGGKGLGKGGAKRHRKVLRDNIQGITKPAIRRLARRGGV
Sbjct  302  ----------------MSGRGKGGKGLGKGGAKRHRKVLRDNIQGITKPAIRRLARRGGV  345

Query  181  KRISGLIYEETRGVLKVFLENVIRDAVTYTEHAKRKTVTAMDVVYALKRQGRTLYGFGG  239
            KRISGLIYEETRGVLKVFLENVIRDAVTYTEHAKRKTVTAMDVVYALKRQGRTLYGFGG
Sbjct  346  KRISGLIYEETRGVLKVFLENVIRDAVTYTEHAKRKTVTAMDVVYALKRQGRTLYGFGG  404
```

Range 2: 1 to 103

Score:205 bits(522), Expect:2e-60,
Method:Compositional matrix adjust.,
Identities:103/103(100%), Positives:103/103(100%), Gaps:0/103(0%)

```
Query  137  MSGRGKGGKGLGKGGAKRHRKVLRDNIQGITKPAIRRLARRGGVKRISGLIYEETRGVLK  196
            MSGRGKGGKGLGKGGAKRHRKVLRDNIQGITKPAIRRLARRGGVKRISGLIYEETRGVLK
Sbjct  1    MSGRGKGGKGLGKGGAKRHRKVLRDNIQGITKPAIRRLARRGGVKRISGLIYEETRGVLK  60

Query  197  VFLENVIRDAVTYTEHAKRKTVTAMDVVYALKRQGRTLYGFGG  239
            VFLENVIRDAVTYTEHAKRKTVTAMDVVYALKRQGRTLYGFGG
Sbjct  61   VFLENVIRDAVTYTEHAKRKTVTAMDVVYALKRQGRTLYGFGG  103
```

>hypothetical protein SFRURICE_010739, partial [Spodoptera frugiperda]

```
Sequence ID: KAF9810277.1 Length: 437
Range 1: 1 to 209

Score:419 bits(1077), Expect:1e-143,
Method:Compositional matrix adjust.,
Identities:205/219(94%), Positives:208/219(94%), Gaps:10/219(4%)

Query  21   LATKAARKSAPSTGGVKKPHRYRPGTVALREIRRYQKSTELLIRKLPFQRLVREIAQDFK  80
            LATKAARKSAP+TGGVKKPHRYRPGTVALREIRRYQKSTELLIRKLPFQRLVREIAQDFK
Sbjct  1    LATKAARKSAPATGGVKKPHRYRPGTVALREIRRYQKSTELLIRKLPFQRLVREIAQDFK  60

Query  81   TDLRFQSAAIGALQEASEAYLVGLFEDTNLCAIHAKRVTIMPKDIQLARRIRGERAMSGR  140
            TDLRFQS+A+ ALQEASEAYLVGLFEDTNLCAIHAKRVTIMPKDIQLARRIR
Sbjct  61   TDLRFQSSAVMALQEASEAYLVGLFEDTNLCAIHAKRVTIMPKDIQLARRIR--------  112

Query  141  GKGGKGLGKGGAKRHRKVLRDNIQGITKPAIRRLARRGGVKRISGLIYEETRGVLKVFLE  200
              GGKGLGKGGAKRHRKVLRDNIQGITKPAIRRLARRGGVKRISGLIYEETRGVLKVFLE
Sbjct  113  --GGKGLGKGGAKRHRKVLRDNIQGITKPAIRRLARRGGVKRISGLIYEETRGVLKVFLE  170

Query  201  NVIRDAVTYTEHAKRKTVTAMDVVYALKRQGRTLYGFGG  239
            NVIRDAVTYTEHAKRKTVTAMDVVYALKRQGRTLYGFGG
Sbjct  171  NVIRDAVTYTEHAKRKTVTAMDVVYALKRQGRTLYGFGG  209
```

```
>uncharacterized protein LOC130054944 [Ostrea edulis]
Sequence ID: XP_056022038.1 Length: 643
Range 1: 424 to 643

Score:426 bits(1094), Expect:2e-143,
Method:Compositional matrix adjust.,
Identities:216/239(90%), Positives:219/239(91%), Gaps:19/239(7%)

Query  1    MARTKQTARKSTGGKAPRKQLATKAARKSAPSTGGVKKPHRYRPGTVALREIRRYQKSTE  60
            MARTKQTARKSTGGKAPRKQLATKAARKSAP+TGGVKKPHRYRPGTVALREIRRYQKSTE
Sbjct  424  MARTKQTARKSTGGKAPRKQLATKAARKSAPATGGVKKPHRYRPGTVALREIRRYQKSTE  483

Query  61   LLIRKLPFQRLVREIAQDFKTDLRFQSAAIGALQEASEAYLVGLFEDTNLCAIHAKRVTI  120
            LLIRKLPFQRLVREIAQDFKTDLRFQS+A+ ALQEASEAYLVGLFEDTNLCAIHAKR
Sbjct  484  LLIRKLPFQRLVREIAQDFKTDLRFQSSAVMALQEASEAYLVGLFEDTNLCAIHAKR---  540

Query  121  MPKDIQLARRIRGERAMSGRGKGGKGLGKGGAKRHRKVLRDNIQGITKPAIRRLARRGGV  180
                             MSGRGKGGKGLGKGGAKRHRKVLRDNIQGITKPAIRRLARRGGV
Sbjct  541  ----------------MSGRGKGGKGLGKGGAKRHRKVLRDNIQGITKPAIRRLARRGGV  584

Query  181  KRISGLIYEETRGVLKVFLENVIRDAVTYTEHAKRKTVTAMDVVYALKRQGRTLYGFGG  239
            KRISGLIYEETRGVLKVFLENVIRDAVTYTEHAKRKTVTAMDVVYALKRQGRTLYGFGG
Sbjct  585  KRISGLIYEETRGVLKVFLENVIRDAVTYTEHAKRKTVTAMDVVYALKRQGRTLYGFGG  643
```

```
Range 2: 1 to 103

Score:209 bits(532), Expect:9e-60,
Method:Compositional matrix adjust.,
Identities:103/103(100%), Positives:103/103(100%), Gaps:0/103(0%)

Query  137  MSGRGKGGKGLGKGGAKRHRKVLRDNIQGITKPAIRRLARRGGVKRISGLIYEETRGVLK  196
            MSGRGKGGKGLGKGGAKRHRKVLRDNIQGITKPAIRRLARRGGVKRISGLIYEETRGVLK
Sbjct  1    MSGRGKGGKGLGKGGAKRHRKVLRDNIQGITKPAIRRLARRGGVKRISGLIYEETRGVLK  60
```

```
Query  197  VFLENVIRDAVTYTEHAKRKTVTAMDVVYALKRQGRTLYGFGG  239
            VFLENVIRDAVTYTEHAKRKTVTAMDVVYALKRQGRTLYGFGG
Sbjct  61   VFLENVIRDAVTYTEHAKRKTVTAMDVVYALKRQGRTLYGFGG  103
```

>hypothetical protein EI555_005829, partial [Monodon monoceros]
Sequence ID: TKC35883.1 Length: 382
Range 1: 93 to 311

Score:416 bits(1068), Expect:3e-143,
Method:Compositional matrix adjust.,
Identities:214/238(90%), Positives:217/238(91%), Gaps:19/238(7%)

```
Query  1    MARTKQTARKSTGGKAPRKQLATKAARKSAPSTGGVKKPHRYRPGTVALREIRRYQKSTE  60
            MARTKQTARKSTGGKAPRKQLATKAARKSAP+TGGVKKPHRYRPGTVALREIRRYQKSTE
Sbjct  93   MARTKQTARKSTGGKAPRKQLATKAARKSAPATGGVKKPHRYRPGTVALREIRRYQKSTE  152

Query  61   LLIRKLPFQRLVREIAQDFKTDLRFQSAAIGALQEASEAYLVGLFEDTNLCAIHAKRVTI  120
            LLIRKLPFQRLVREIAQDFKTDLRFQS+A+ ALQEA EAYLVGLFEDTNLCAIHAKR
Sbjct  153  LLIRKLPFQRLVREIAQDFKTDLRFQSSAVMALQEACEAYLVGLFEDTNLCAIHAKR---  209

Query  121  MPKDIQLARRIRGERAMSGRGKGGKGLGKGGAKRHRKVLRDNIQGITKPAIRRLARRGGV  180
                             MSGRGKGGKGLGKGGAKRHRKVLRDNIQGITKPAIRRLARRGGV
Sbjct  210  ----------------MSGRGKGGKGLGKGGAKRHRKVLRDNIQGITKPAIRRLARRGGV  253

Query  181  KRISGLIYEETRGVLKVFLENVIRDAVTYTEHAKRKTVTAMDVVYALKRQGRTLYGFG  238
            KRISGLIYEETRGVLKVFLENVIRDAVTYTEHAKRKTVTAMDVVYALKRQGRTLYGFG
Sbjct  254  KRISGLIYEETRGVLKVFLENVIRDAVTYTEHAKRKTVTAMDVVYALKRQGRTLYGFG  311
```

>LOW QUALITY PROTEIN: uncharacterized protein LOC115532367 [Gadus morhua]
Sequence ID: XP_030197991.1 Length: 677
Range 1: 460 to 677

Score:426 bits(1095), Expect:3e-143,
Method:Compositional matrix adjust.,
Identities:214/239(90%), Positives:217/239(90%), Gaps:21/239(8%)

```
Query  1    MARTKQTARKSTGGKAPRKQLATKAARKSAPSTGGVKKPHRYRPGTVALREIRRYQKSTE  60
            MARTKQTARKSTGGKAPRKQLATKAARKSAP+TGGVKKPHRYRPGTVALREIRRYQKSTE
Sbjct  460  MARTKQTARKSTGGKAPRKQLATKAARKSAPATGGVKKPHRYRPGTVALREIRRYQKSTE  519

Query  61   LLIRKLPFQRLVREIAQDFKTDLRFQSAAIGALQEASEAYLVGLFEDTNLCAIHAKRVTI  120
            LLIRKLPFQRLVREIAQDFKTDLRFQS+A+ ALQEASEAYLVGLFEDTNLCAIH
Sbjct  520  LLIRKLPFQRLVREIAQDFKTDLRFQSSAVMALQEASEAYLVGLFEDTNLCAIH------  573

Query  121  MPKDIQLARRIRGERAMSGRGKGGKGLGKGGAKRHRKVLRDNIQGITKPAIRRLARRGGV  180
                            AMSGRGKGGKGLGKGGAKRHRKVLRDNIQGITKPAIRRLARRGGV
Sbjct  574  ---------------AMSGRGKGGKGLGKGGAKRHRKVLRDNIQGITKPAIRRLARRGGV  618

Query  181  KRISGLIYEETRGVLKVFLENVIRDAVTYTEHAKRKTVTAMDVVYALKRQGRTLYGFGG  239
            KRISGLIYEETRGVLKVFLENVIRDAVTYTEHAKRKTVTAMDVVYALKRQGRTLYGFGG
Sbjct  619  KRISGLIYEETRGVLKVFLENVIRDAVTYTEHAKRKTVTAMDVVYALKRQGRTLYGFGG  677
```

Range 2: 1 to 102

```
Score:210 bits(534), Expect:1e-59,
Method:Compositional matrix adjust.,
Identities:102/102(100%), Positives:102/102(100%), Gaps:0/102(0%)

Query  137  MSGRGKGGKGLGKGGAKRHRKVLRDNIQGITKPAIRRLARRGGVKRISGLIYEETRGVLK  196
            MSGRGKGGKGLGKGGAKRHRKVLRDNIQGITKPAIRRLARRGGVKRISGLIYEETRGVLK
Sbjct  1    MSGRGKGGKGLGKGGAKRHRKVLRDNIQGITKPAIRRLARRGGVKRISGLIYEETRGVLK  60

Query  197  VFLENVIRDAVTYTEHAKRKTVTAMDVVYALKRQGRTLYGFG  238
            VFLENVIRDAVTYTEHAKRKTVTAMDVVYALKRQGRTLYGFG
Sbjct  61   VFLENVIRDAVTYTEHAKRKTVTAMDVVYALKRQGRTLYGFG  102



>uncharacterized protein LOC111162609 [Enhydra lutris kenyoni]
Sequence ID: XP_022382829.1 Length: 328
Range 1: 111 to 328

Score:413 bits(1062), Expect:4e-143,
Method:Compositional matrix adjust.,
Identities:212/239(89%), Positives:215/239(89%), Gaps:21/239(8%)

Query  1    MARTKQTARKSTGGKAPRKQLATKAARKSAPSTGGVKKPHRYRPGTVALREIRRYQKSTE  60
            MARTKQTARKSTGGKAPRKQLATKAARKSAP+TGGVKKPHRYRPGTVALREIRRYQKSTE
Sbjct  111  MARTKQTARKSTGGKAPRKQLATKAARKSAPATGGVKKPHRYRPGTVALREIRRYQKSTE  170

Query  61   LLIRKLPFQRLVREIAQDFKTDLRFQSAAIGALQEASEAYLVGLFEDTNLCAIHAKRVTI  120
            LLIRKLPFQRLVREIAQDFKTDLRFQS+A+ ALQEA EAYLVGLFEDTNLCAIH
Sbjct  171  LLIRKLPFQRLVREIAQDFKTDLRFQSSAVMALQEACEAYLVGLFEDTNLCAIH------  224

Query  121  MPKDIQLARRIRGERAMSGRGKGGKGLGKGGAKRHRKVLRDNIQGITKPAIRRLARRGGV  180
                            MSGRGKGGKGLGKGGAKRHRKVLRDNIQGITKPAIRRLARRGGV
Sbjct  225  ---------------IMSGRGKGGKGLGKGGAKRHRKVLRDNIQGITKPAIRRLARRGGV  269

Query  181  KRISGLIYEETRGVLKVFLENVIRDAVTYTEHAKRKTVTAMDVVYALKRQGRTLYGFGG  239
            KRISGLIYEETRGVLKVFLENVIRDAVTYTEHAKRKTVTAMDVVYALKRQGRTLYGFGG
Sbjct  270  KRISGLIYEETRGVLKVFLENVIRDAVTYTEHAKRKTVTAMDVVYALKRQGRTLYGFGG  328



Range 2: 1 to 96

Score:190 bits(483), Expect:2e-55,
Method:Compositional matrix adjust.,
Identities:96/96(100%), Positives:96/96(100%), Gaps:0/96(0%)

Query  137  MSGRGKGGKGLGKGGAKRHRKVLRDNIQGITKPAIRRLARRGGVKRISGLIYEETRGVLK  196
            MSGRGKGGKGLGKGGAKRHRKVLRDNIQGITKPAIRRLARRGGVKRISGLIYEETRGVLK
Sbjct  1    MSGRGKGGKGLGKGGAKRHRKVLRDNIQGITKPAIRRLARRGGVKRISGLIYEETRGVLK  60

Query  197  VFLENVIRDAVTYTEHAKRKTVTAMDVVYALKRQGR  232
            VFLENVIRDAVTYTEHAKRKTVTAMDVVYALKRQGR
Sbjct  61   VFLENVIRDAVTYTEHAKRKTVTAMDVVYALKRQGR  96



>uncharacterized protein LOC100980546 [Pan paniscus]
Sequence ID: XP_054970369.2 Length: 573
Range 1: 349 to 573
```

```
Score:422 bits(1084), Expect:6e-143,
Method:Compositional matrix adjust.,
Identities:215/239(90%), Positives:219/239(91%), Gaps:14/239(5%)

Query  1    MARTKQTARKSTGGKAPRKQLATKAARKSAPSTGGVKKPHRYRPGTVALREIRRYQKSTE  60
            MARTKQTARKSTGGKAPRKQLATKAARKSAP+TGGVKKPHRYRPGTVALREIRRYQKSTE
Sbjct  349  MARTKQTARKSTGGKAPRKQLATKAARKSAPATGGVKKPHRYRPGTVALREIRRYQKSTE  408

Query  61   LLIRKLPFQRLVREIAQDFKTDLRFQSAAIGALQEASEAYLVGLFEDTNLCAIHAKRVTI  120
            LLIRKLPFQRLVREIAQDFKTDLRFQS+A+ ALQEA EAYLVGLFEDTNLCAIHAKR+
Sbjct  409  LLIRKLPFQRLVREIAQDFKTDLRFQSSAVMALQEACEAYLVGLFEDTNLCAIHAKRLL-  467

Query  121  MPKDIQLARRIRGERAMSGRGKGGKGLGKGGAKRHRKVLRDNIQGITKPAIRRLARRGGV  180
                             MSGRGKGGKGLGKGGAKRHRKVLRDNIQGITKPAIRRLARRGGV
Sbjct  468  -------------SSIMSGRGKGGKGLGKGGAKRHRKVLRDNIQGITKPAIRRLARRGGV  514

Query  181  KRISGLIYEETRGVLKVFLENVIRDAVTYTEHAKRKTVTAMDVVYALKRQGRTLYGFGG  239
            KRISGLIYEETRGVLKVFLENVIRDAVTYTEHAKRKTVTAMDVVYALKRQGRTLYGFGG
Sbjct  515  KRISGLIYEETRGVLKVFLENVIRDAVTYTEHAKRKTVTAMDVVYALKRQGRTLYGFGG  573



Range 2: 11 to 138

Score:173 bits(439), Expect:1e-46,
Method:Compositional matrix adjust.,
Identities:108/128(84%), Positives:116/128(90%), Gaps:2/128(1%)

Query  114  HAKRVTIMPKDI-QLARRIR-GERAMSGRGKGGKGLGKGGAKRHRKVLRDNIQGITKPAI  171
            +++ + I+P  + Q++  I  G   MSGRGKGGKGLGKGGAKRHRKVLRDNIQGITKPAI
Sbjct  11   NSRHIRILPSLVFQISAAIFVGVLVMSGRGKGGKGLGKGGAKRHRKVLRDNIQGITKPAI  70

Query  172  RRLARRGGVKRISGLIYEETRGVLKVFLENVIRDAVTYTEHAKRKTVTAMDVVYALKRQG  231
            RRLARRGGVKRISGLIYEETRGVLKVFLENVIRDAVTYTEHAKRKTVTAMDVVYALKRQG
Sbjct  71   RRLARRGGVKRISGLIYEETRGVLKVFLENVIRDAVTYTEHAKRKTVTAMDVVYALKRQG  130

Query  232  RTLYGFGG  239
            RTLYGFGG
Sbjct  131  RTLYGFGG  138



>uncharacterized protein LOC131687205 [Topomyia yanbarensis]
Sequence ID: XP_058827240.1 Length: 624
Range 1: 407 to 624

Score:422 bits(1084), Expect:3e-142,
Method:Compositional matrix adjust.,
Identities:213/239(89%), Positives:217/239(90%), Gaps:21/239(8%)

Query  1    MARTKQTARKSTGGKAPRKQLATKAARKSAPSTGGVKKPHRYRPGTVALREIRRYQKSTE  60
            MARTKQTARKSTGGKAPRKQLATKAARKSAP+TGGVKKPHRYRPGTVALREIRRYQKSTE
Sbjct  407  MARTKQTARKSTGGKAPRKQLATKAARKSAPATGGVKKPHRYRPGTVALREIRRYQKSTE  466

Query  61   LLIRKLPFQRLVREIAQDFKTDLRFQSAAIGALQEASEAYLVGLFEDTNLCAIHAKRVTI  120
            LLIRKLPFQRLVREIAQDFKTDLRFQS+A+ ALQEASEAYLVGLFEDTNLCAIH
Sbjct  467  LLIRKLPFQRLVREIAQDFKTDLRFQSSAVMALQEASEAYLVGLFEDTNLCAIH------  520

Query  121  MPKDIQLARRIRGERAMSGRGKGGKGLGKGGAKRHRKVLRDNIQGITKPAIRRLARRGGV  180
                           AM+GRGKGGKGLGKGGAKRHRKVLRDNIQGITKPAIRRLARRGGV
Sbjct  521  ---------------AMTGRGKGGKGLGKGGAKRHRKVLRDNIQGITKPAIRRLARRGGV  565
```

```
Query  181  KRISGLIYEETRGVLKVFLENVIRDAVTYTEHAKRKTVTAMDVVYALKRQGRTLYGFGG  239
            KRISGLIYEETRGVLKVFLENVIRDAVTYTEHAKRKTVTAMDVVYALKRQGRTLYGFGG
Sbjct  566  KRISGLIYEETRGVLKVFLENVIRDAVTYTEHAKRKTVTAMDVVYALKRQGRTLYGFGG  624
```

Range 2: 1 to 102

Score:207 bits(526), Expect:6e-59,
Method:Compositional matrix adjust.,
Identities:101/102(99%), Positives:102/102(100%), Gaps:0/102(0%)

```
Query  137  MSGRGKGGKGLGKGGAKRHRKVLRDNIQGITKPAIRRLARRGGVKRISGLIYEETRGVLK  196
            M+GRGKGGKGLGKGGAKRHRKVLRDNIQGITKPAIRRLARRGGVKRISGLIYEETRGVLK
Sbjct  1    MTGRGKGGKGLGKGGAKRHRKVLRDNIQGITKPAIRRLARRGGVKRISGLIYEETRGVLK  60

Query  197  VFLENVIRDAVTYTEHAKRKTVTAMDVVYALKRQGRTLYGFG  238
            VFLENVIRDAVTYTEHAKRKTVTAMDVVYALKRQGRTLYGFG
Sbjct  61   VFLENVIRDAVTYTEHAKRKTVTAMDVVYALKRQGRTLYGFG  102
```

>histone H3.1 [Rousettus aegyptiacus]
Sequence ID: XP_015988254.2 Length: 218
Range 1: 1 to 218

Score:407 bits(1045), Expect:3e-142,
Method:Compositional matrix adjust.,
Identities:209/239(87%), Positives:212/239(88%), Gaps:21/239(8%)

```
Query  1    MARTKQTARKSTGGKAPRKQLATKAARKSAPSTGGVKKPHRYRPGTVALREIRRYQKSTE  60
            MARTKQTARKSTGGKAPRKQLATKAARKSAP+TGGVKKPHRYRPGTVALREIRRYQKSTE
Sbjct  1    MARTKQTARKSTGGKAPRKQLATKAARKSAPATGGVKKPHRYRPGTVALREIRRYQKSTE  60

Query  61   LLIRKLPFQRLVREIAQDFKTDLRFQSAAIGALQEASEAYLVGLFEDTNLCAIHAKRVTI  120
            LLIRKLPFQRLVREIAQDFKTDLRFQS+A+ ALQEA EAYLVGLFEDTNLC
Sbjct  61   LLIRKLPFQRLVREIAQDFKTDLRFQSSAVMALQEACEAYLVGLFEDTNLCVTA------  114

Query  121  MPKDIQLARRIRGERAMSGRGKGGKGLGKGGAKRHRKVLRDNIQGITKPAIRRLARRGGV  180
                             MSGRGKGGKGLGKGGAKRHRKVLRDNIQGITKPAIRRLARRGGV
Sbjct  115  ---------------TMSGRGKGGKGLGKGGAKRHRKVLRDNIQGITKPAIRRLARRGGV  159

Query  181  KRISGLIYEETRGVLKVFLENVIRDAVTYTEHAKRKTVTAMDVVYALKRQGRTLYGFGG  239
            KRISGLIYEETRGVLKVFLENVIRDAVTYTEHAKRKTVTAMDVVYALKRQGRTLYGFGG
Sbjct  160  KRISGLIYEETRGVLKVFLENVIRDAVTYTEHAKRKTVTAMDVVYALKRQGRTLYGFGG  218
```

>uncharacterized protein LOC117732052 [Cyclopterus lumpus]
Sequence ID: XP_034390553.1 Length: 605
Range 1: 386 to 605

Score:421 bits(1081), Expect:5e-142,
Method:Compositional matrix adjust.,
Identities:215/239(90%), Positives:219/239(91%), Gaps:19/239(7%)

```
Query  1    MARTKQTARKSTGGKAPRKQLATKAARKSAPSTGGVKKPHRYRPGTVALREIRRYQKSTE  60
            MARTKQTARKSTGGKAPRKQLATKAARKSAP+TGGVKKPHRYRPGTVALREIRRYQKSTE
Sbjct  386  MARTKQTARKSTGGKAPRKQLATKAARKSAPATGGVKKPHRYRPGTVALREIRRYQKSTE  445
```

```
Query  61   LLIRKLPFQRLVREIAQDFKTDLRFQSAAIGALQEASEAYLVGLFEDTNLCAIHAKRVTI  120
            LLIRKLPFQRLVREIAQDFKTDLRFQS+A+ ALQE+SEAYLVGLFEDTNLCAIHAKR
Sbjct  446  LLIRKLPFQRLVREIAQDFKTDLRFQSSAVMALQESSEAYLVGLFEDTNLCAIHAKR---  502

Query  121  MPKDIQLARRIRGERAMSGRGKGGKGLGKGGAKRHRKVLRDNIQGITKPAIRRLARRGGV  180
                             MSGRGKGGKGLGKGGAKRHRKVLRDNIQGITKPAIRRLARRGGV
Sbjct  503  ----------------MSGRGKGGKGLGKGGAKRHRKVLRDNIQGITKPAIRRLARRGGV  546

Query  181  KRISGLIYEETRGVLKVFLENVIRDAVTYTEHAKRKTVTAMDVVYALKRQGRTLYGFGG  239
            KRISGLIYEETRGVLKVFLENVIRDAVTYTEHAKRKTVTAMDVVYALKRQGRTLYGFGG
Sbjct  547  KRISGLIYEETRGVLKVFLENVIRDAVTYTEHAKRKTVTAMDVVYALKRQGRTLYGFGG  605
```

Range 2: 1 to 102

Score:204 bits(519), Expect:4e-58,
Method:Compositional matrix adjust.,
Identities:102/102(100%), Positives:102/102(100%), Gaps:0/102(0%)

```
Query  137  MSGRGKGGKGLGKGGAKRHRKVLRDNIQGITKPAIRRLARRGGVKRISGLIYEETRGVLK  196
            MSGRGKGGKGLGKGGAKRHRKVLRDNIQGITKPAIRRLARRGGVKRISGLIYEETRGVLK
Sbjct  1    MSGRGKGGKGLGKGGAKRHRKVLRDNIQGITKPAIRRLARRGGVKRISGLIYEETRGVLK  60

Query  197  VFLENVIRDAVTYTEHAKRKTVTAMDVVYALKRQGRTLYGFG  238
            VFLENVIRDAVTYTEHAKRKTVTAMDVVYALKRQGRTLYGFG
Sbjct  61   VFLENVIRDAVTYTEHAKRKTVTAMDVVYALKRQGRTLYGFG  102
```

Range 3: 220 to 268

Score:102 bits(254), Expect:7e-21,
Method:Compositional matrix adjust.,
Identities:49/49(100%), Positives:49/49(100%), Gaps:0/49(0%)

```
Query  191  TRGVLKVFLENVIRDAVTYTEHAKRKTVTAMDVVYALKRQGRTLYGFGG  239
            TRGVLKVFLENVIRDAVTYTEHAKRKTVTAMDVVYALKRQGRTLYGFGG
Sbjct  220  TRGVLKVFLENVIRDAVTYTEHAKRKTVTAMDVVYALKRQGRTLYGFGG  268
```

>uncharacterized protein LOC130040149 [Sorex fumeus]
Sequence ID: XP_055988138.1 Length: 579
Range 1: 360 to 579

Score:419 bits(1077), Expect:7e-142,
Method:Compositional matrix adjust.,
Identities:215/239(90%), Positives:218/239(91%), Gaps:19/239(7%)

```
Query  1    MARTKQTARKSTGGKAPRKQLATKAARKSAPSTGGVKKPHRYRPGTVALREIRRYQKSTE  60
            MARTKQTARKSTGGKAPRKQLATKAARKSAP+TGGVKKPHRYRPGTVALREIRRYQKSTE
Sbjct  360  MARTKQTARKSTGGKAPRKQLATKAARKSAPATGGVKKPHRYRPGTVALREIRRYQKSTE  419

Query  61   LLIRKLPFQRLVREIAQDFKTDLRFQSAAIGALQEASEAYLVGLFEDTNLCAIHAKRVTI  120
            LLIRKLPFQRLVREIAQDFKTDLRFQS+A+ ALQEA EAYLVGLFEDTNLCAIHAKR
Sbjct  420  LLIRKLPFQRLVREIAQDFKTDLRFQSSAVMALQEACEAYLVGLFEDTNLCAIHAKR---  476

Query  121  MPKDIQLARRIRGERAMSGRGKGGKGLGKGGAKRHRKVLRDNIQGITKPAIRRLARRGGV  180
```

```
                                 MSGRGKGGKGLGKGGAKRHRKVLRDNIQGITKPAIRRLARRGGV
Sbjct  477  ----------------MSGRGKGGKGLGKGGAKRHRKVLRDNIQGITKPAIRRLARRGGV  520

Query  181  KRISGLIYEETRGVLKVFLENVIRDAVTYTEHAKRKTVTAMDVVYALKRQGRTLYGFGG  239
            KRISGLIYEETRGVLKVFLENVIRDAVTYTEHAKRKTVTAMDVVYALKRQGRTLYGFGG
Sbjct  521  KRISGLIYEETRGVLKVFLENVIRDAVTYTEHAKRKTVTAMDVVYALKRQGRTLYGFGG  579
```

Range 2: 1 to 103

Score:207 bits(526), Expect:3e-59,
Method:Compositional matrix adjust.,
Identities:103/103(100%), Positives:103/103(100%), Gaps:0/103(0%)

```
Query  137  MSGRGKGGKGLGKGGAKRHRKVLRDNIQGITKPAIRRLARRGGVKRISGLIYEETRGVLK  196
            MSGRGKGGKGLGKGGAKRHRKVLRDNIQGITKPAIRRLARRGGVKRISGLIYEETRGVLK
Sbjct  1    MSGRGKGGKGLGKGGAKRHRKVLRDNIQGITKPAIRRLARRGGVKRISGLIYEETRGVLK  60

Query  197  VFLENVIRDAVTYTEHAKRKTVTAMDVVYALKRQGRTLYGFGG  239
            VFLENVIRDAVTYTEHAKRKTVTAMDVVYALKRQGRTLYGFGG
Sbjct  61   VFLENVIRDAVTYTEHAKRKTVTAMDVVYALKRQGRTLYGFGG  103
```

>uncharacterized protein LOC117817566 [Notolabrus celidotus]
Sequence ID: XP_034546197.1 Length: 373
Range 1: 156 to 373

Score:411 bits(1057), Expect:1e-141,
Method:Compositional matrix adjust.,
Identities:212/239(89%), Positives:215/239(89%), Gaps:21/239(8%)

```
Query  1    MARTKQTARKSTGGKAPRKQLATKAARKSAPSTGGVKKPHRYRPGTVALREIRRYQKSTE  60
            MARTKQTARKSTGGKAPRKQLATKAARKSAP+TGGVKKPHRYRPGTVALREIRRYQKSTE
Sbjct  156  MARTKQTARKSTGGKAPRKQLATKAARKSAPATGGVKKPHRYRPGTVALREIRRYQKSTE  215

Query  61   LLIRKLPFQRLVREIAQDFKTDLRFQSAAIGALQEASEAYLVGLFEDTNLCAIHAKRVTI  120
            LLIRKLPFQRLVREIAQDFKTDLRFQS+A+ ALQEASEAYLVGLFEDTNLCAI
Sbjct  216  LLIRKLPFQRLVREIAQDFKTDLRFQSSAVMALQEASEAYLVGLFEDTNLCAII------  269

Query  121  MPKDIQLARRIRGERAMSGRGKGGKGLGKGGAKRHRKVLRDNIQGITKPAIRRLARRGGV  180
                             MSGRGKGGKGLGKGGAKRHRKVLRDNIQGITKPAIRRLARRGGV
Sbjct  270  ---------------IMSGRGKGGKGLGKGGAKRHRKVLRDNIQGITKPAIRRLARRGGV  314

Query  181  KRISGLIYEETRGVLKVFLENVIRDAVTYTEHAKRKTVTAMDVVYALKRQGRTLYGFGG  239
            KRISGLIYEETRGVLKVFLENVIRDAVTYTEHAKRKTVTAMDVVYALKRQGRTLYGFGG
Sbjct  315  KRISGLIYEETRGVLKVFLENVIRDAVTYTEHAKRKTVTAMDVVYALKRQGRTLYGFGG  373
```

Range 2: 1 to 102

Score:204 bits(520), Expect:2e-60,
Method:Compositional matrix adjust.,
Identities:102/102(100%), Positives:102/102(100%), Gaps:0/102(0%)

```
Query  137  MSGRGKGGKGLGKGGAKRHRKVLRDNIQGITKPAIRRLARRGGVKRISGLIYEETRGVLK  196
            MSGRGKGGKGLGKGGAKRHRKVLRDNIQGITKPAIRRLARRGGVKRISGLIYEETRGVLK
Sbjct  1    MSGRGKGGKGLGKGGAKRHRKVLRDNIQGITKPAIRRLARRGGVKRISGLIYEETRGVLK  60
```

```
Query  197   VFLENVIRDAVTYTEHAKRKTVTAMDVVYALKRQGRTLYGFG  238
             VFLENVIRDAVTYTEHAKRKTVTAMDVVYALKRQGRTLYGFG
Sbjct  61    VFLENVIRDAVTYTEHAKRKTVTAMDVVYALKRQGRTLYGFG  102
```

>uncharacterized protein LOC131977843 [Centropristis striata]
Sequence ID: XP_059197270.1 Length: 598
Range 1: 381 to 598

Score:419 bits(1078), Expect:1e-141,
Method:Compositional matrix adjust.,
Identities:213/239(89%), Positives:217/239(90%), Gaps:21/239(8%)

```
Query  1     MARTKQTARKSTGGKAPRKQLATKAARKSAPSTGGVKKPHRYRPGTVALREIRRYQKSTE  60
             MARTKQTARKSTGGKAPRKQLATKAARKSAP+TGGVKKPHRYRPGTVALREIRRYQKSTE
Sbjct  381   MARTKQTARKSTGGKAPRKQLATKAARKSAPATGGVKKPHRYRPGTVALREIRRYQKSTE  440

Query  61    LLIRKLPFQRLVREIAQDFKTDLRFQSAAIGALQEASEAYLVGLFEDTNLCAIHAKRVTI  120
             LLIRKLPFQRLVREIAQDFKTDLRFQS+A+ ALQE+SEAYLVGLFEDTNLCAIH
Sbjct  441   LLIRKLPFQRLVREIAQDFKTDLRFQSSAVMALQESSEAYLVGLFEDTNLCAIH------  494

Query  121   MPKDIQLARRIRGERAMSGRGKGGKGLGKGGAKRHRKVLRDNIQGITKPAIRRLARRGGV  180
                             AMSGRGKGGKGLGKGGAKRHRKVLRDNIQGITKPAIRRLARRGGV
Sbjct  495   ---------------AMSGRGKGGKGLGKGGAKRHRKVLRDNIQGITKPAIRRLARRGGV  539

Query  181   KRISGLIYEETRGVLKVFLENVIRDAVTYTEHAKRKTVTAMDVVYALKRQGRTLYGFGG  239
             KRISGLIYEETRGVLKVFLENVIRDAVTYTEHAKRKTVTAMDVVYALKRQGRTLYGFGG
Sbjct  540   KRISGLIYEETRGVLKVFLENVIRDAVTYTEHAKRKTVTAMDVVYALKRQGRTLYGFGG  598
```

Range 2: 1 to 103

Score:208 bits(530), Expect:1e-59,
Method:Compositional matrix adjust.,
Identities:103/103(100%), Positives:103/103(100%), Gaps:0/103(0%)

```
Query  137   MSGRGKGGKGLGKGGAKRHRKVLRDNIQGITKPAIRRLARRGGVKRISGLIYEETRGVLK  196
             MSGRGKGGKGLGKGGAKRHRKVLRDNIQGITKPAIRRLARRGGVKRISGLIYEETRGVLK
Sbjct  1     MSGRGKGGKGLGKGGAKRHRKVLRDNIQGITKPAIRRLARRGGVKRISGLIYEETRGVLK  60

Query  197   VFLENVIRDAVTYTEHAKRKTVTAMDVVYALKRQGRTLYGFGG  239
             VFLENVIRDAVTYTEHAKRKTVTAMDVVYALKRQGRTLYGFGG
Sbjct  61    VFLENVIRDAVTYTEHAKRKTVTAMDVVYALKRQGRTLYGFGG  103
```

>uncharacterized protein LOC135181613 [Pogoniulus pusillus]
Sequence ID: XP_064010917.1 Length: 327
Range 1: 110 to 327

Score:409 bits(1051), Expect:2e-141,
Method:Compositional matrix adjust.,
Identities:210/239(88%), Positives:214/239(89%), Gaps:21/239(8%)

```
Query  1     MARTKQTARKSTGGKAPRKQLATKAARKSAPSTGGVKKPHRYRPGTVALREIRRYQKSTE  60
             MARTKQTARKSTGGKAPRKQLATKAARKSAP+TGGVKKPHRYRPGTVALREIRRYQKSTE
Sbjct  110   MARTKQTARKSTGGKAPRKQLATKAARKSAPATGGVKKPHRYRPGTVALREIRRYQKSTE  169
```

```
Query  61   LLIRKLPFQRLVREIAQDFKTDLRFQSAAIGALQEASEAYLVGLFEDTNLCAIHAKRVTI  120
            LLIRKLPFQRLVREIAQDFKTDLRFQS+A+ ALQEASEAYLVGLFEDTNLC +
Sbjct  170  LLIRKLPFQRLVREIAQDFKTDLRFQSSAVMALQEASEAYLVGLFEDTNLCIVS------  223

Query  121  MPKDIQLARRIRGERAMSGRGKGGKGLGKGGAKRHRKVLRDNIQGITKPAIRRLARRGGV  180
                             MSGRGKGGKGLGKGGAKRHRKVLRDNIQGITKPAIRRLARRGGV
Sbjct  224  ---------------VMSGRGKGGKGLGKGGAKRHRKVLRDNIQGITKPAIRRLARRGGV  268

Query  181  KRISGLIYEETRGVLKVFLENVIRDAVTYTEHAKRKTVTAMDVVYALKRQGRTLYGFGG  239
            KRISGLIYEETRGVLKVFLENVIRDAVTYTEHAKRKTVTAMDVVYALKRQGRTLYGFGG
Sbjct  269  KRISGLIYEETRGVLKVFLENVIRDAVTYTEHAKRKTVTAMDVVYALKRQGRTLYGFGG  327
```

Range 2: 1 to 102

Score:204 bits(518), Expect:1e-60,
Method:Compositional matrix adjust.,
Identities:102/102(100%), Positives:102/102(100%), Gaps:0/102(0%)

```
Query  137  MSGRGKGGKGLGKGGAKRHRKVLRDNIQGITKPAIRRLARRGGVKRISGLIYEETRGVLK  196
            MSGRGKGGKGLGKGGAKRHRKVLRDNIQGITKPAIRRLARRGGVKRISGLIYEETRGVLK
Sbjct  1    MSGRGKGGKGLGKGGAKRHRKVLRDNIQGITKPAIRRLARRGGVKRISGLIYEETRGVLK  60

Query  197  VFLENVIRDAVTYTEHAKRKTVTAMDVVYALKRQGRTLYGFG  238
            VFLENVIRDAVTYTEHAKRKTVTAMDVVYALKRQGRTLYGFG
Sbjct  61   VFLENVIRDAVTYTEHAKRKTVTAMDVVYALKRQGRTLYGFG  102
```

>uncharacterized protein LOC135194285 [Vanessa tameamea]
Sequence ID: XP_064075668.1 Length: 517
Range 1: 300 to 517

Score:415 bits(1067), Expect:3e-141,
Method:Compositional matrix adjust.,
Identities:212/239(89%), Positives:216/239(90%), Gaps:21/239(8%)

```
Query  1    MARTKQTARKSTGGKAPRKQLATKAARKSAPSTGGVKKPHRYRPGTVALREIRRYQKSTE  60
            MARTKQTARKSTGGKAPRKQLATKAARKSAP+TGGVKKPHRYRPGTVALREIRRYQKSTE
Sbjct  300  MARTKQTARKSTGGKAPRKQLATKAARKSAPATGGVKKPHRYRPGTVALREIRRYQKSTE  359

Query  61   LLIRKLPFQRLVREIAQDFKTDLRFQSAAIGALQEASEAYLVGLFEDTNLCAIHAKRVTI  120
            LLIRKLPFQRLVREIAQDFKTDLRFQS+A+ ALQEASEAYLVGLFEDTNLCAIH
Sbjct  360  LLIRKLPFQRLVREIAQDFKTDLRFQSSAVMALQEASEAYLVGLFEDTNLCAIH------  413

Query  121  MPKDIQLARRIRGERAMSGRGKGGKGLGKGGAKRHRKVLRDNIQGITKPAIRRLARRGGV  180
                             M+GRGKGGKGLGKGGAKRHRKVLRDNIQGITKPAIRRLARRGGV
Sbjct  414  ---------------KMTGRGKGGKGLGKGGAKRHRKVLRDNIQGITKPAIRRLARRGGV  458

Query  181  KRISGLIYEETRGVLKVFLENVIRDAVTYTEHAKRKTVTAMDVVYALKRQGRTLYGFGG  239
            KRISGLIYEETRGVLKVFLENVIRDAVTYTEHAKRKTVTAMDVVYALKRQGRTLYGFGG
Sbjct  459  KRISGLIYEETRGVLKVFLENVIRDAVTYTEHAKRKTVTAMDVVYALKRQGRTLYGFGG  517
```

Range 2: 1 to 102

Score:205 bits(521), Expect:4e-59,

```
Method:Compositional matrix adjust.,
Identities:101/102(99%), Positives:102/102(100%), Gaps:0/102(0%)

Query  137   MSGRGKGGKGLGKGGAKRHRKVLRDNIQGITKPAIRRLARRGGVKRISGLIYEETRGVLK  196
             M+GRGKGGKGLGKGGAKRHRKVLRDNIQGITKPAIRRLARRGGVKRISGLIYEETRGVLK
Sbjct  1     MTGRGKGGKGLGKGGAKRHRKVLRDNIQGITKPAIRRLARRGGVKRISGLIYEETRGVLK  60

Query  197   VFLENVIRDAVTYTEHAKRKTVTAMDVVYALKRQGRTLYGFG  238
             VFLENVIRDAVTYTEHAKRKTVTAMDVVYALKRQGRTLYGFG
Sbjct  61    VFLENVIRDAVTYTEHAKRKTVTAMDVVYALKRQGRTLYGFG  102



>unnamed protein product [Lampetra fluviatilis]
Sequence ID: CAN0156380.1 Length: 404
Range 1: 1 to 268

Score:411 bits(1056), Expect:5e-141,
Method:Compositional matrix adjust.,
Identities:220/269(82%), Positives:223/269(82%), Gaps:31/269(11%)

Query  1     MARTKQTARKSTGGKAPRKQLATKAARKSAPSTGGVKKPHRYRPGTVALREIRRYQKSTE  60
             MARTKQTARKSTGGKAPRKQLATKAARKSAP+TGGVKKPHRYRPGTVALREIRRYQKSTE
Sbjct  1     MARTKQTARKSTGGKAPRKQLATKAARKSAPATGGVKKPHRYRPGTVALREIRRYQKSTE  60

Query  61    LLIRKLPFQRLVREIAQDFKTDLRFQSAAIGALQEASEAYLVGLFEDTNLCAIHAKRVTI  120
             LLIRKLPFQRLVREIAQDFKTDLRFQS+A+ ALQEASEAYLV LFEDTNLCAIHAKRVTI
Sbjct  61    LLIRKLPFQRLVREIAQDFKTDLRFQSSAVMALQEASEAYLVALFEDTNLCAIHAKRVTI  120

Query  121   MPKDIQLARRIRGER-----AMSGRGKGGKGLGKG------------------------  150
             MPKDIQLARRIRGER      M  RG
Sbjct  121   MPKDIQLARRIRGERVIPPCVMLHRGSLTTAYSYSRNVVLFTQVVGMSGRGKGGKGLGKG  180

Query  151   GAKRHRKVLRDNIQGITKPAIRRLARRGGVKRISGLIYEETRGVLKVFLENVIRDAVTYT  210
             GAKRHRKVLRDNIQGITKPAIRRLARRGGVKRISGLIYEETRGVLKVFLENVIRDAVTYT
Sbjct  181   GAKRHRKVLRDNIQGITKPAIRRLARRGGVKRISGLIYEETRGVLKVFLENVIRDAVTYT  240

Query  211   EHAKRKTVTAMDVVYALKRQGRTLYGFGG  239
             EHAKRKTVTAMDVVYAL RQGRTLYGFG
Sbjct  241   EHAKRKTVTAMDVVYAL-RQGRTLYGFGA  268



Range 2: 269 to 404

Score:266 bits(679), Expect:6e-84,
Method:Compositional matrix adjust.,
Identities:131/136(96%), Positives:134/136(98%), Gaps:0/136(0%)

Query  1     MARTKQTARKSTGGKAPRKQLATKAARKSAPSTGGVKKPHRYRPGTVALREIRRYQKSTE  60
             MARTKQTARKSTGGKAPRKQLATKAARKSAP+TGGVKKPHRYRPGTVALREIRRYQKSTE
Sbjct  269   MARTKQTARKSTGGKAPRKQLATKAARKSAPATGGVKKPHRYRPGTVALREIRRYQKSTE  328

Query  61    LLIRKLPFQRLVREIAQDFKTDLRFQSAAIGALQEASEAYLVGLFEDTNLCAIHAKRVTI  120
             LLIRKLPFQRLVREIAQDFKTDLRFQS+A+ ALQEASEAYLV LFEDTNLCAIHAKRVTI
Sbjct  329   LLIRKLPFQRLVREIAQDFKTDLRFQSSAVMALQEASEAYLVALFEDTNLCAIHAKRVTI  388

Query  121   MPKDIQLARRIRGERA  136
             MPKDIQLARRIRGERA
Sbjct  389   MPKDIQLARRIRGERA  404
```

```
>histone H3-like centromeric protein CSE4 [Poecilia latipinna]
Sequence ID: XP_014913934.1 Length: 251
Range 1: 37 to 251

Score:405 bits(1040), Expect:5e-141,
Method:Compositional matrix adjust.,
Identities:209/239(87%), Positives:212/239(88%), Gaps:24/239(10%)

Query  1    MARTKQTARKSTGGKAPRKQLATKAARKSAPSTGGVKKPHRYRPGTVALREIRRYQKSTE  60
            MARTKQTARKSTGGKAPRKQLATKAARKSAP+TGGVKKPHRYRPGTVALREIRRYQKSTE
Sbjct  37   MARTKQTARKSTGGKAPRKQLATKAARKSAPATGGVKKPHRYRPGTVALREIRRYQKSTE  96

Query  61   LLIRKLPFQRLVREIAQDFKTDLRFQSAAIGALQEASEAYLVGLFEDTNLCAIHAKRVTI  120
            LLIRKLPFQRLVREIAQDFKTDLRFQS+A+ ALQEASEAYLVGLFEDTNL
Sbjct  97   LLIRKLPFQRLVREIAQDFKTDLRFQSSAVMALQEASEAYLVGLFEDTNLV---------  147

Query  121  MPKDIQLARRIRGERAMSGRGKGGKGLGKGGAKRHRKVLRDNIQGITKPAIRRLARRGGV  180
                            MSGRGKGGKGLGKGGAKRHRKVLRDNIQGITKPAIRRLARRGGV
Sbjct  148  ---------------IMSGRGKGGKGLGKGGAKRHRKVLRDNIQGITKPAIRRLARRGGV  192

Query  181  KRISGLIYEETRGVLKVFLENVIRDAVTYTEHAKRKTVTAMDVVYALKRQGRTLYGFGG  239
            KRISGLIYEETRGVLKVFLENVIRDAVTYTEHAKRKTVTAMDVVYALKRQGRTLYGFGG
Sbjct  193  KRISGLIYEETRGVLKVFLENVIRDAVTYTEHAKRKTVTAMDVVYALKRQGRTLYGFGG  251
```

```
>LOW QUALITY PROTEIN: uncharacterized protein LOC116762442 [Phocoena sinus]
Sequence ID: XP_032505252.1 Length: 307
Range 1: 90 to 307

Score:407 bits(1046), Expect:6e-141,
Method:Compositional matrix adjust.,
Identities:209/239(87%), Positives:213/239(89%), Gaps:21/239(8%)

Query  1    MARTKQTARKSTGGKAPRKQLATKAARKSAPSTGGVKKPHRYRPGTVALREIRRYQKSTE  60
            MARTKQTARKSTGGKAPRKQLATKAARKSAP+TGGVKKPHRYRPGTVALREIRRYQKSTE
Sbjct  90   MARTKQTARKSTGGKAPRKQLATKAARKSAPATGGVKKPHRYRPGTVALREIRRYQKSTE  149

Query  61   LLIRKLPFQRLVREIAQDFKTDLRFQSAAIGALQEASEAYLVGLFEDTNLCAIHAKRVTI  120
            LLIRKLPFQRLVREIAQDFKTDLRFQS+A+ ALQEA EAYLVGLFEDTNLC +
Sbjct  150  LLIRKLPFQRLVREIAQDFKTDLRFQSSAVMALQEACEAYLVGLFEDTNLCXVL------  203

Query  121  MPKDIQLARRIRGERAMSGRGKGGKGLGKGGAKRHRKVLRDNIQGITKPAIRRLARRGGV  180
                            MSGRGKGGKGLGKGGAKRHRKVLRDNIQGITKPAIRRLARRGGV
Sbjct  204  ---------------VMSGRGKGGKGLGKGGAKRHRKVLRDNIQGITKPAIRRLARRGGV  248

Query  181  KRISGLIYEETRGVLKVFLENVIRDAVTYTEHAKRKTVTAMDVVYALKRQGRTLYGFGG  239
            KRISGLIYEETRGVLKVFLENVIRDAVTYTEHAKRKTVTAMDVVYALKRQGRTLYGFGG
Sbjct  249  KRISGLIYEETRGVLKVFLENVIRDAVTYTEHAKRKTVTAMDVVYALKRQGRTLYGFGG  307
```

```
>uncharacterized protein LOC120982704 [Bufo bufo]
Sequence ID: XP_040268895.1 Length: 509
Range 1: 292 to 509

Score:414 bits(1063), Expect:1e-140,
Method:Compositional matrix adjust.,
```

```
Identities:213/239(89%), Positives:216/239(90%), Gaps:21/239(8%)

Query  1    MARTKQTARKSTGGKAPRKQLATKAARKSAPSTGGVKKPHRYRPGTVALREIRRYQKSTE  60
            MARTKQTARKSTGGKAPRKQLATKAARKSAP+TGGVKKPHRYRPGTVALREIRRYQKSTE
Sbjct  292  MARTKQTARKSTGGKAPRKQLATKAARKSAPATGGVKKPHRYRPGTVALREIRRYQKSTE  351

Query  61   LLIRKLPFQRLVREIAQDFKTDLRFQSAAIGALQEASEAYLVGLFEDTNLCAIHAKRVTI  120
            LLIRKLPFQRLVREIAQDFKTDLRFQS+A+ ALQEASEAYLVGLFEDTNLCAIH
Sbjct  352  LLIRKLPFQRLVREIAQDFKTDLRFQSSAVMALQEASEAYLVGLFEDTNLCAIH------  405

Query  121  MPKDIQLARRIRGERAMSGRGKGGKGLGKGGAKRHRKVLRDNIQGITKPAIRRLARRGGV  180
                             MSGRGKGGKGLGKGGAKRHRKVLRDNIQGITKPAIRRLARRGGV
Sbjct  406  ---------------KMSGRGKGGKGLGKGGAKRHRKVLRDNIQGITKPAIRRLARRGGV  450

Query  181  KRISGLIYEETRGVLKVFLENVIRDAVTYTEHAKRKTVTAMDVVYALKRQGRTLYGFGG  239
            KRISGLIYEETRGVLKVFLENVIRDAVTYTEHAKRKTVTAMDVVYALKRQGRTLYGFGG
Sbjct  451  KRISGLIYEETRGVLKVFLENVIRDAVTYTEHAKRKTVTAMDVVYALKRQGRTLYGFGG  509



Range 2: 1 to 100

Score:199 bits(506), Expect:5e-57,
Method:Compositional matrix adjust.,
Identities:100/100(100%), Positives:100/100(100%), Gaps:0/100(0%)

Query  137  MSGRGKGGKGLGKGGAKRHRKVLRDNIQGITKPAIRRLARRGGVKRISGLIYEETRGVLK  196
            MSGRGKGGKGLGKGGAKRHRKVLRDNIQGITKPAIRRLARRGGVKRISGLIYEETRGVLK
Sbjct  1    MSGRGKGGKGLGKGGAKRHRKVLRDNIQGITKPAIRRLARRGGVKRISGLIYEETRGVLK  60

Query  197  VFLENVIRDAVTYTEHAKRKTVTAMDVVYALKRQGRTLYG  236
            VFLENVIRDAVTYTEHAKRKTVTAMDVVYALKRQGRTLYG
Sbjct  61   VFLENVIRDAVTYTEHAKRKTVTAMDVVYALKRQGRTLYG  100



>histone H3.v1-like [Periophthalmus magnuspinnatus]
Sequence ID: XP_033834558.1 Length: 520
Range 1: 303 to 520

Score:414 bits(1064), Expect:1e-140,
Method:Compositional matrix adjust.,
Identities:211/239(88%), Positives:216/239(90%), Gaps:21/239(8%)

Query  1    MARTKQTARKSTGGKAPRKQLATKAARKSAPSTGGVKKPHRYRPGTVALREIRRYQKSTE  60
            MARTKQTARKSTGGKAPRKQLATKAARKSAP+TGGVKKPHRYRPGTVALREIRRYQKSTE
Sbjct  303  MARTKQTARKSTGGKAPRKQLATKAARKSAPATGGVKKPHRYRPGTVALREIRRYQKSTE  362

Query  61   LLIRKLPFQRLVREIAQDFKTDLRFQSAAIGALQEASEAYLVGLFEDTNLCAIHAKRVTI  120
            LLIRKLPFQRLVREIAQDFKTDLRFQS+A+ ALQEASEAYLVGLFEDTNL A+ +K
Sbjct  363  LLIRKLPFQRLVREIAQDFKTDLRFQSSAVMALQEASEAYLVGLFEDTNL-AVSSK----  417

Query  121  MPKDIQLARRIRGERAMSGRGKGGKGLGKGGAKRHRKVLRDNIQGITKPAIRRLARRGGV  180
                             MSGRGKGGKGLGKGGAKRHRKVLRDNIQGITKPAIRRLARRGGV
Sbjct  418  ----------------MSGRGKGGKGLGKGGAKRHRKVLRDNIQGITKPAIRRLARRGGV  461

Query  181  KRISGLIYEETRGVLKVFLENVIRDAVTYTEHAKRKTVTAMDVVYALKRQGRTLYGFGG  239
            KRISGLIYEETRGVLKVFLENVIRDAVTYTEHAKRKTVTAMDVVYALKRQGRTLYGFGG
Sbjct  462  KRISGLIYEETRGVLKVFLENVIRDAVTYTEHAKRKTVTAMDVVYALKRQGRTLYGFGG  520
```

Range 2: 1 to 103

Score:213 bits(541), Expect:5e-62,
Method:Compositional matrix adjust.,
Identities:103/103(100%), Positives:103/103(100%), Gaps:0/103(0%)

```
Query  137   MSGRGKGGKGLGKGGAKRHRKVLRDNIQGITKPAIRRLARRGGVKRISGLIYEETRGVLK  196
             MSGRGKGGKGLGKGGAKRHRKVLRDNIQGITKPAIRRLARRGGVKRISGLIYEETRGVLK
Sbjct  1     MSGRGKGGKGLGKGGAKRHRKVLRDNIQGITKPAIRRLARRGGVKRISGLIYEETRGVLK  60

Query  197   VFLENVIRDAVTYTEHAKRKTVTAMDVVYALKRQGRTLYGFGG  239
             VFLENVIRDAVTYTEHAKRKTVTAMDVVYALKRQGRTLYGFGG
Sbjct  61    VFLENVIRDAVTYTEHAKRKTVTAMDVVYALKRQGRTLYGFGG  103
```

>uncharacterized protein LOC113965756 [Neopelma chrysocephalum]
Sequence ID: XP_027535400.1 Length: 364
Range 1: 147 to 364

Score:408 bits(1049), Expect:1e-140,
Method:Compositional matrix adjust.,
Identities:211/239(88%), Positives:216/239(90%), Gaps:21/239(8%)

```
Query  1     MARTKQTARKSTGGKAPRKQLATKAARKSAPSTGGVKKPHRYRPGTVALREIRRYQKSTE  60
             MARTKQTARKSTGGKAPRKQLATKAARKSAP+TGGVKKPHRYRPGTVALREIRRYQKSTE
Sbjct  147   MARTKQTARKSTGGKAPRKQLATKAARKSAPATGGVKKPHRYRPGTVALREIRRYQKSTE  206

Query  61    LLIRKLPFQRLVREIAQDFKTDLRFQSAAIGALQEASEAYLVGLFEDTNLCAIHAKRVTI  120
             LLIRKLPFQRLVREIAQDFKTDLRFQS+A+ ALQEASEAYLVGLFEDTNL A++
Sbjct  207   LLIRKLPFQRLVREIAQDFKTDLRFQSSAVMALQEASEAYLVGLFEDTNLAAVN------  260

Query  121   MPKDIQLARRIRGERAMSGRGKGGKGLGKGGAKRHRKVLRDNIQGITKPAIRRLARRGGV  180
                             AMSGRGKGGKGLGKGGAKRHRKVLRDNIQGITKPAIRRLARRGGV
Sbjct  261   ---------------AMSGRGKGGKGLGKGGAKRHRKVLRDNIQGITKPAIRRLARRGGV  305

Query  181   KRISGLIYEETRGVLKVFLENVIRDAVTYTEHAKRKTVTAMDVVYALKRQGRTLYGFGG  239
             KRISGLIYEETRGVLKVFLENVIRDAVTYTEHAKRKTVTAMDVVYALKRQGRTLYGFGG
Sbjct  306   KRISGLIYEETRGVLKVFLENVIRDAVTYTEHAKRKTVTAMDVVYALKRQGRTLYGFGG  364
```

Range 2: 1 to 103

Score:206 bits(523), Expect:5e-61,
Method:Compositional matrix adjust.,
Identities:103/103(100%), Positives:103/103(100%), Gaps:0/103(0%)

```
Query  137   MSGRGKGGKGLGKGGAKRHRKVLRDNIQGITKPAIRRLARRGGVKRISGLIYEETRGVLK  196
             MSGRGKGGKGLGKGGAKRHRKVLRDNIQGITKPAIRRLARRGGVKRISGLIYEETRGVLK
Sbjct  1     MSGRGKGGKGLGKGGAKRHRKVLRDNIQGITKPAIRRLARRGGVKRISGLIYEETRGVLK  60

Query  197   VFLENVIRDAVTYTEHAKRKTVTAMDVVYALKRQGRTLYGFGG  239
             VFLENVIRDAVTYTEHAKRKTVTAMDVVYALKRQGRTLYGFGG
Sbjct  61    VFLENVIRDAVTYTEHAKRKTVTAMDVVYALKRQGRTLYGFGG  103
```

```
>uncharacterized protein LOC131911336 [Peromyscus eremicus]
Sequence ID: XP_059119301.1 Length: 331
Range 1: 114 to 331

Score:407 bits(1045), Expect:2e-140,
Method:Compositional matrix adjust.,
Identities:209/239(87%), Positives:214/239(89%), Gaps:21/239(8%)

Query  1    MARTKQTARKSTGGKAPRKQLATKAARKSAPSTGGVKKPHRYRPGTVALREIRRYQKSTE  60
            MARTKQTARKSTGGKAPRKQLATKAARKSAP+TGGVKKPHRYRPGTVALREIRRYQKSTE
Sbjct  114  MARTKQTARKSTGGKAPRKQLATKAARKSAPATGGVKKPHRYRPGTVALREIRRYQKSTE  173

Query  61   LLIRKLPFQRLVREIAQDFKTDLRFQSAAIGALQEASEAYLVGLFEDTNLCAIHAKRVTI  120
            LLIRKLPFQRLVREIAQDFKTDLRFQS+A+ ALQEA EAYLVGLFEDTNLC +
Sbjct  174  LLIRKLPFQRLVREIAQDFKTDLRFQSSAVMALQEACEAYLVGLFEDTNLCIVV------  227

Query  121  MPKDIQLARRIRGERAMSGRGKGGKGLGKGGAKRHRKVLRDNIQGITKPAIRRLARRGGV  180
                           +MSGRGKGGKGLGKGGAKRHRKVLRDNIQGITKPAIRRLARRGGV
Sbjct  228  ---------------SMSGRGKGGKGLGKGGAKRHRKVLRDNIQGITKPAIRRLARRGGV  272

Query  181  KRISGLIYEETRGVLKVFLENVIRDAVTYTEHAKRKTVTAMDVVYALKRQGRTLYGFGG  239
            KRISGLIYEETRGVLKVFLENVIRDAVTYTEHAKRKTVTAMDVVYALKRQGRTLYGFGG
Sbjct  273  KRISGLIYEETRGVLKVFLENVIRDAVTYTEHAKRKTVTAMDVVYALKRQGRTLYGFGG  331



Range 2: 1 to 96

Score:190 bits(482), Expect:3e-55,
Method:Compositional matrix adjust.,
Identities:96/96(100%), Positives:96/96(100%), Gaps:0/96(0%)

Query  137  MSGRGKGGKGLGKGGAKRHRKVLRDNIQGITKPAIRRLARRGGVKRISGLIYEETRGVLK  196
            MSGRGKGGKGLGKGGAKRHRKVLRDNIQGITKPAIRRLARRGGVKRISGLIYEETRGVLK
Sbjct  1    MSGRGKGGKGLGKGGAKRHRKVLRDNIQGITKPAIRRLARRGGVKRISGLIYEETRGVLK  60

Query  197  VFLENVIRDAVTYTEHAKRKTVTAMDVVYALKRQGR  232
            VFLENVIRDAVTYTEHAKRKTVTAMDVVYALKRQGR
Sbjct  61   VFLENVIRDAVTYTEHAKRKTVTAMDVVYALKRQGR  96
```

H4H3 query sequence and obtained alignments through BLAST searches

<u>Query sequence</u>:

MSGRGKGGKGLGKGGAKRHRKVLRDNIQGITKPAIRRLARRGGVKRISGLIYEETRGVLK
VFLENVIRDAVTYTEHAKRKTVTAMDVVYALKRQGRTLYGFGGMARTKQTARKSTGGK
APRKQLATKAARKSAPSTGGVKKPHRYRPGTVALREIRRYQKSTELLIRKLPFQRLVREIAQ
DFKTDLRFQSAAIGALQEASEAYLVGLFEDTNLCAIHAKRVTIMPKDIQLARRIRGERA

<u>Obtained alignments between the query and the BLAST-retrieved sequences</u>:

>histone H3-like [Pomacea canaliculata]
Sequence ID: XP_025104607.1 Length: 245
Range 1: 1 to 245

Score:463 bits(1192), Expect:3e-164,
Method:Compositional matrix adjust.,
Identities:235/245(96%), Positives:238/245(97%), Gaps:6/245(2%)

```
Query  1    MSGRGKGGKGLGKGGAKRHRKVLRDNIQGITKPAIRRLARRGGVKRISGLIYEETRGVLK  60
            MSGRGKGGKGLGKGGAKRHRKVLRDNIQGITKPAIRRLARRGGVKRISGLIYEETRGVLK
Sbjct  1    MSGRGKGGKGLGKGGAKRHRKVLRDNIQGITKPAIRRLARRGGVKRISGLIYEETRGVLK  60

Query  61   VFLENVIRDAVTYTEHAKRKTVTAMDVVYALKRQGRTLYGFGG------MARTKQTARKS  114
            VFLENVIRDAVTYTEHAKRKTVTAMDVVYALKRQGRTLYGFGG      MARTKQTARKS
Sbjct  61   VFLENVIRDAVTYTEHAKRKTVTAMDVVYALKRQGRTLYGFGGTHKTFAMARTKQTARKS  120

Query  115  TGGKAPRKQLATKAARKSAPSTGGVKKPHRYRPGTVALREIRRYQKSTELLIRKLPFQRL  174
            TGGKAPRKQLATKAARKSAP+TGGVKKPHRYRPGTVALREIRRYQKSTELLIRKLPFQRL
Sbjct  121  TGGKAPRKQLATKAARKSAPATGGVKKPHRYRPGTVALREIRRYQKSTELLIRKLPFQRL  180

Query  175  VREIAQDFKTDLRFQSAAIGALQEASEAYLVGLFEDTNLCAIHAKRVTIMPKDIQLARRI  234
            VREIAQDFKTDLRFQS+A+ ALQEASEAYLVGLFEDTNLCAIHAKRVTIMPKDIQLARRI
Sbjct  181  VREIAQDFKTDLRFQSSAVMALQEASEAYLVGLFEDTNLCAIHAKRVTIMPKDIQLARRI  240

Query  235  RGERA  239
            RGERA
Sbjct  241  RGERA  245
```

>uncharacterized protein [Physeter macrocephalus]
Sequence ID: XP_028335332.1 Length: 383
Range 1: 145 to 383

Score:466 bits(1200), Expect:3e-163,
Method:Compositional matrix adjust.,
Identities:233/239(97%), Positives:236/239(98%), Gaps:0/239(0%)

```
Query  1    MSGRGKGGKGLGKGGAKRHRKVLRDNIQGITKPAIRRLARRGGVKRISGLIYEETRGVLK  60
            MSGRGKGGKGLGKGGAKRHRKVLRDNIQGITKPAIRRLARRGGVKRISGLIYEETRGVLK
Sbjct  145  MSGRGKGGKGLGKGGAKRHRKVLRDNIQGITKPAIRRLARRGGVKRISGLIYEETRGVLK  204

Query  61   VFLENVIRDAVTYTEHAKRKTVTAMDVVYALKRQGRTLYGFGGMARTKQTARKSTGGKAP  120
            VFLENVIRDAVTYTEHAKRKTVTAMDVVYALKRQGRTLYGFG MARTKQTARKSTGGKAP
Sbjct  205  VFLENVIRDAVTYTEHAKRKTVTAMDVVYALKRQGRTLYGFGVMARTKQTARKSTGGKAP  264

Query  121  RKQLATKAARKSAPSTGGVKKPHRYRPGTVALREIRRYQKSTELLIRKLPFQRLVREIAQ  180
            RKQLATKAARKSAP+TGGVKKPHRYRPGTVALREIRRYQKSTELLIRKLPFQRLVREIAQ
Sbjct  265  RKQLATKAARKSAPATGGVKKPHRYRPGTVALREIRRYQKSTELLIRKLPFQRLVREIAQ  324

Query  181  DFKTDLRFQSAAIGALQEASEAYLVGLFEDTNLCAIHAKRVTIMPKDIQLARRIRGERA  239
            DFKTDLRFQS+A+ ALQEA EAYLVGLFEDTNLCAIHAKRVTIMPKDIQLARRIRGERA
Sbjct  325  DFKTDLRFQSSAVMALQEACEAYLVGLFEDTNLCAIHAKRVTIMPKDIQLARRIRGERA  383
```

Range 2: 1 to 136

Score:265 bits(678), Expect:4e-84,
Method:Compositional matrix adjust.,
Identities:131/136(96%), Positives:134/136(98%), Gaps:0/136(0%)

```
Query  104  MARTKQTARKSTGGKAPRKQLATKAARKSAPSTGGVKKPHRYRPGTVALREIRRYQKSTE  163
            MARTKQTARKSTGGKAPRKQLATKAARKSAP+TGGVKKPHRYRPGTVALREIRRYQKSTE
Sbjct  1    MARTKQTARKSTGGKAPRKQLATKAARKSAPATGGVKKPHRYRPGTVALREIRRYQKSTE  60

Query  164  LLIRKLPFQRLVREIAQDFKTDLRFQSAAIGALQEASEAYLVGLFEDTNLCAIHAKRVTI  223
            LLIRKLPFQRLVREIAQDFKTDLRFQS+A+ ALQEA EAYLVGLFEDTNLCAIHAKRVTI
Sbjct  61   LLIRKLPFQRLVREIAQDFKTDLRFQSSAVMALQEACEAYLVGLFEDTNLCAIHAKRVTI  120

Query  224  MPKDIQLARRIRGERA  239
            MPKDIQLARRIRGERA
Sbjct  121  MPKDIQLARRIRGERA  136
```

>unnamed protein product [Lampetra fluviatilis]
Sequence ID: CAN0156380.1 Length: 404
Range 1: 167 to 404

Score:462 bits(1189), Expect:2e-161,
Method:Compositional matrix adjust.,
Identities:232/239(97%), Positives:235/239(98%), Gaps:1/239(0%)

```
Query  1    MSGRGKGGKGLGKGGAKRHRKVLRDNIQGITKPAIRRLARRGGVKRISGLIYEETRGVLK  60
            MSGRGKGGKGLGKGGAKRHRKVLRDNIQGITKPAIRRLARRGGVKRISGLIYEETRGVLK
Sbjct  167  MSGRGKGGKGLGKGGAKRHRKVLRDNIQGITKPAIRRLARRGGVKRISGLIYEETRGVLK  226

Query  61   VFLENVIRDAVTYTEHAKRKTVTAMDVVYALKRQGRTLYGFGGMARTKQTARKSTGGKAP  120
            VFLENVIRDAVTYTEHAKRKTVTAMDVVYAL RQGRTLYGFG MARTKQTARKSTGGKAP
Sbjct  227  VFLENVIRDAVTYTEHAKRKTVTAMDVVYAL-RQGRTLYGFGAMARTKQTARKSTGGKAP  285

Query  121  RKQLATKAARKSAPSTGGVKKPHRYRPGTVALREIRRYQKSTELLIRKLPFQRLVREIAQ  180
            RKQLATKAARKSAP+TGGVKKPHRYRPGTVALREIRRYQKSTELLIRKLPFQRLVREIAQ
Sbjct  286  RKQLATKAARKSAPATGGVKKPHRYRPGTVALREIRRYQKSTELLIRKLPFQRLVREIAQ  345

Query  181  DFKTDLRFQSAAIGALQEASEAYLVGLFEDTNLCAIHAKRVTIMPKDIQLARRIRGERA  239
            DFKTDLRFQS+A+ ALQEASEAYLV LFEDTNLCAIHAKRVTIMPKDIQLARRIRGERA
Sbjct  346  DFKTDLRFQSSAVMALQEASEAYLVALFEDTNLCAIHAKRVTIMPKDIQLARRIRGERA  404
```

Range 2: 1 to 135

Score:264 bits(675), Expect:2e-83,
Method:Compositional matrix adjust.,
Identities:130/135(96%), Positives:133/135(98%), Gaps:0/135(0%)

```
Query  104  MARTKQTARKSTGGKAPRKQLATKAARKSAPSTGGVKKPHRYRPGTVALREIRRYQKSTE  163
            MARTKQTARKSTGGKAPRKQLATKAARKSAP+TGGVKKPHRYRPGTVALREIRRYQKSTE
Sbjct  1    MARTKQTARKSTGGKAPRKQLATKAARKSAPATGGVKKPHRYRPGTVALREIRRYQKSTE  60

Query  164  LLIRKLPFQRLVREIAQDFKTDLRFQSAAIGALQEASEAYLVGLFEDTNLCAIHAKRVTI  223
            LLIRKLPFQRLVREIAQDFKTDLRFQS+A+ ALQEASEAYLV LFEDTNLCAIHAKRVTI
Sbjct  61   LLIRKLPFQRLVREIAQDFKTDLRFQSSAVMALQEASEAYLVALFEDTNLCAIHAKRVTI  120

Query  224  MPKDIQLARRIRGER  238
            MPKDIQLARRIRGER
Sbjct  121  MPKDIQLARRIRGER  135
```

>uncharacterized protein LOC119458563 [Dermacentor silvarum]
Sequence ID: XP_037576330.2 Length: 289
Range 1: 45 to 289

Score:456 bits(1172), Expect:1e-160,
Method:Compositional matrix adjust.,
Identities:231/245(94%), Positives:235/245(95%), Gaps:6/245(2%)

```
Query  1    MSGRGKGGKGLGKGGAKRHRKVLRDNIQGITKPAIRRLARRGGVKRISGLIYEETRGVLK  60
            MSGRGKGGKGLGKGGAKRHRKVLRDNIQGITKPAIRRLARRGGVKRISGLIYEETRGVLK
Sbjct  45   MSGRGKGGKGLGKGGAKRHRKVLRDNIQGITKPAIRRLARRGGVKRISGLIYEETRGVLK  104

Query  61   VFLENVIRDAVTYTEHAKRKTVTAMDVVYALKRQGRTLYGF------GGMARTKQTARKS  114
            VFLENVIRDAVTYTEHAKRKTVTAMDVVYALKRQGRTLY +        MARTKQTARKS
Sbjct  105  VFLENVIRDAVTYTEHAKRKTVTAMDVVYALKRQGRTLYVWTLPSPSSAMARTKQTARKS  164

Query  115  TGGKAPRKQLATKAARKSAPSTGGVKKPHRYRPGTVALREIRRYQKSTELLIRKLPFQRL  174
            TGGKAPRKQLATKAARKSAP+TGGVKKPHRYRPGTVALREIRRYQKSTELLIRKLPFQRL
Sbjct  165  TGGKAPRKQLATKAARKSAPATGGVKKPHRYRPGTVALREIRRYQKSTELLIRKLPFQRL  224

Query  175  VREIAQDFKTDLRFQSAAIGALQEASEAYLVGLFEDTNLCAIHAKRVTIMPKDIQLARRI  234
            VREIAQDFKTDLRFQS+A+ ALQEASEAYLVGLFEDTNLCAIHAKRVTIMPKDIQLARRI
Sbjct  225  VREIAQDFKTDLRFQSSAVMALQEASEAYLVGLFEDTNLCAIHAKRVTIMPKDIQLARRI  284

Query  235  RGERA  239
            RGERA
Sbjct  285  RGERA  289
```

>histone H3-like [Centropristis striata]
Sequence ID: XP_059197081.1 Length: 239
Range 1: 1 to 239

```
Score:454 bits(1167), Expect:2e-160,
Method:Compositional matrix adjust.,
Identities:229/239(96%), Positives:233/239(97%), Gaps:0/239(0%)

Query  1    MSGRGKGGKGLGKGGAKRHRKVLRDNIQGITKPAIRRLARRGGVKRISGLIYEETRGVLK  60
            MSGRGKGGKGLGKGGAKRHRKVLRDNIQGITKPAIRRLARRGGVKRISGLIYEETRGVLK
Sbjct  1    MSGRGKGGKGLGKGGAKRHRKVLRDNIQGITKPAIRRLARRGGVKRISGLIYEETRGVLK  60

Query  61   VFLENVIRDAVTYTEHAKRKTVTAMDVVYALKRQGRTLYGFGGMARTKQTARKSTGGKAP  120
            VFLENVIRDAVTYTEHAKRKTVTAMDVVYALKRQGRTL     MARTKQTARKSTGGKAP
Sbjct  61   VFLENVIRDAVTYTEHAKRKTVTAMDVVYALKRQGRTLKKEVKMARTKQTARKSTGGKAP  120

Query  121  RKQLATKAARKSAPSTGGVKKPHRYRPGTVALREIRRYQKSTELLIRKLPFQRLVREIAQ  180
            RKQLATKAARKSAP+TGGVKKPHRYRPGTVALREIRRYQKSTELLIRKLPFQRLVREIAQ
Sbjct  121  RKQLATKAARKSAPATGGVKKPHRYRPGTVALREIRRYQKSTELLIRKLPFQRLVREIAQ  180

Query  181  DFKTDLRFQSAAIGALQEASEAYLVGLFEDTNLCAIHAKRVTIMPKDIQLARRIRGERA  239
            DFKTDLRFQS+A+ ALQE+SEAYLVGLFEDTNLCAIHAKRVTIMPKDIQLARRIRGERA
Sbjct  181  DFKTDLRFQSSAVMALQESSEAYLVGLFEDTNLCAIHAKRVTIMPKDIQLARRIRGERA  239
```

```
>histone H3-like [Pteropus medius]
Sequence ID: XP_039697405.1 Length: 242
Range 1: 1 to 242

Score:453 bits(1166), Expect:3e-160,
Method:Compositional matrix adjust.,
Identities:232/242(96%), Positives:235/242(97%), Gaps:3/242(1%)

Query  1    MSGRGKGGKGLGKGGAKRHRKVLRDNIQGITKPAIRRLARRGGVKRISGLIYEETRGVLK  60
            MSGRGKGGKGLGKGGAKRHRKVLRDNIQGITKPAIRRLARRGGVKRISGLIYEETRGVLK
Sbjct  1    MSGRGKGGKGLGKGGAKRHRKVLRDNIQGITKPAIRRLARRGGVKRISGLIYEETRGVLK  60

Query  61   VFLENVIRDAVTYTEHAKRKTVTAMDVVYALKRQGRTLY--GF-GGMARTKQTARKSTGG  117
            VFLENVIRDAVTYTEHAKRKTVTAMDVVYALKRQGRTLY   F   MARTKQTARKSTGG
Sbjct  61   VFLENVIRDAVTYTEHAKRKTVTAMDVVYALKRQGRTLYVRSFRFAMARTKQTARKSTGG  120

Query  118  KAPRKQLATKAARKSAPSTGGVKKPHRYRPGTVALREIRRYQKSTELLIRKLPFQRLVRE  177
            KAPRKQLATKAARKSAP+TGGVKKPHRYRPGTVALREIRRYQKSTELLIRKLPFQRLVRE
Sbjct  121  KAPRKQLATKAARKSAPATGGVKKPHRYRPGTVALREIRRYQKSTELLIRKLPFQRLVRE  180

Query  178  IAQDFKTDLRFQSAAIGALQEASEAYLVGLFEDTNLCAIHAKRVTIMPKDIQLARRIRGE  237
            IAQDFKTDLRFQS+A+ ALQEASEAYLVGLFEDTNLCAIHAKRVTIMPKDIQLARRIRGE
Sbjct  181  IAQDFKTDLRFQSSAVMALQEASEAYLVGLFEDTNLCAIHAKRVTIMPKDIQLARRIRGE  240

Query  238  RA  239
            RA
Sbjct  241  RA  242
```

>uncharacterized protein LOC124391707 [Silurus meridionalis]
Sequence ID: XP_046714411.1 Length: 400
Range 1: 1 to 240

Score:459 bits(1182), Expect:3e-160,
Method:Compositional matrix adjust.,
Identities:232/240(97%), Positives:235/240(97%), Gaps:3/240(1%)

```
Query  1    MSGRGKGGKGLGKGGAKRHRKVLRDNIQGITKPAIRRLARRGGVKRISGLIYEETRGVLK  60
            MSGRGKGGKGLGKGGAKRHRKVLRDNIQGITKPAIRRLARRGGVKRISGLIYEETRGVLK
Sbjct  1    MSGRGKGGKGLGKGGAKRHRKVLRDNIQGITKPAIRRLARRGGVKRISGLIYEETRGVLK  60

Query  61   VFLENVIRDAVTYTEHAKRKTVTAMDVVYALKRQGRTLYGFGG---MARTKQTARKSTGG  117
            VFLENVIRDAVTYTEHAKRKTVTAMDVVYALKRQGRTLYGFG    MARTKQTARKSTGG
Sbjct  61   VFLENVIRDAVTYTEHAKRKTVTAMDVVYALKRQGRTLYGFGALNAMARTKQTARKSTGG  120

Query  118  KAPRKQLATKAARKSAPSTGGVKKPHRYRPGTVALREIRRYQKSTELLIRKLPFQRLVRE  177
            KAPRKQLATKAARKSAP+TGGVKKPHRYRPGTVALREIRRYQKSTELLIRKLPFQRLVRE
Sbjct  121  KAPRKQLATKAARKSAPATGGVKKPHRYRPGTVALREIRRYQKSTELLIRKLPFQRLVRE  180

Query  178  IAQDFKTDLRFQSAAIGALQEASEAYLVGLFEDTNLCAIHAKRVTIMPKDIQLARRIRGE  237
            IAQDFKTDLRFQS+A+ ALQEASEAYLVGLFEDTNLCAIHAKRVTIMPKDIQLARRIRGE
Sbjct  181  IAQDFKTDLRFQSSAVMALQEASEAYLVGLFEDTNLCAIHAKRVTIMPKDIQLARRIRGE  240
```

Range 2: 298 to 400

Score:207 bits(526), Expect:4e-61,
Method:Compositional matrix adjust.,
Identities:103/103(100%), Positives:103/103(100%), Gaps:0/103(0%)

```
Query  1    MSGRGKGGKGLGKGGAKRHRKVLRDNIQGITKPAIRRLARRGGVKRISGLIYEETRGVLK  60
            MSGRGKGGKGLGKGGAKRHRKVLRDNIQGITKPAIRRLARRGGVKRISGLIYEETRGVLK
Sbjct  298  MSGRGKGGKGLGKGGAKRHRKVLRDNIQGITKPAIRRLARRGGVKRISGLIYEETRGVLK  357

Query  61   VFLENVIRDAVTYTEHAKRKTVTAMDVVYALKRQGRTLYGFGG  103
            VFLENVIRDAVTYTEHAKRKTVTAMDVVYALKRQGRTLYGFGG
Sbjct  358  VFLENVIRDAVTYTEHAKRKTVTAMDVVYALKRQGRTLYGFGG  400
```

>LOW QUALITY PROTEIN: uncharacterized protein LOC119946961 [Tachyglossus aculeatus]
Sequence ID: XP_038624369.1 Length: 454
Range 1: 207 to 454

Score:461 bits(1185), Expect:5e-160,
Method:Compositional matrix adjust.,
Identities:234/248(94%), Positives:237/248(95%), Gaps:9/248(3%)

```
Query  1    MSGRGKGGKGLGKGGAKRHRKVLRDNIQGITKPAIRRLARRGGVKRISGLIYEETRGVLK  60
```

```
            MSGRGKGGKGLGKGGAKRHRKVLRDNIQGITKPAIRRLARRGGVKRISGLIYEETRGVLK
Sbjct  207  MSGRGKGGKGLGKGGAKRHRKVLRDNIQGITKPAIRRLARRGGVKRISGLIYEETRGVLK  266

Query  61   VFLENVIRDAVTYTEHAKRKTVTAMDVVYALKRQGRTLYGFGG---------MARTKQTA  111
            VFLENVIRDAVTYTEHAKRKTVTAMDVVYALKRQGRTLYGFG          MARTKQTA
Sbjct  267  VFLENVIRDAVTYTEHAKRKTVTAMDVVYALKRQGRTLYGFGVTCYLLALLIMARTKQTA  326

Query  112  RKSTGGKAPRKQLATKAARKSAPSTGGVKKPHRYRPGTVALREIRRYQKSTELLIRKLPF  171
            RKSTGGKAPRKQLATKAARKSAP+TGGVKKPHRYRPGTVALREIRRYQKSTELLIRKLPF
Sbjct  327  RKSTGGKAPRKQLATKAARKSAPATGGVKKPHRYRPGTVALREIRRYQKSTELLIRKLPF  386

Query  172  QRLVREIAQDFKTDLRFQSAAIGALQEASEAYLVGLFEDTNLCAIHAKRVTIMPKDIQLA  231
            QRLVREIAQDFKTDLRFQS+A+ ALQEASEAYLVGLFEDTNLCAIHAKRVTIMPKDIQLA
Sbjct  387  QRLVREIAQDFKTDLRFQSSAVMALQEASEAYLVGLFEDTNLCAIHAKRVTIMPKDIQLA  446

Query  232  RRIRGERA  239
            RRIRGERA
Sbjct  447  RRIRGERA  454
```

Range 2: 1 to 133

Score:260 bits(665), Expect:3e-81,
Method:Compositional matrix adjust.,
Identities:128/133(96%), Positives:131/133(98%), Gaps:0/133(0%)

```
Query  104  MARTKQTARKSTGGKAPRKQLATKAARKSAPSTGGVKKPHRYRPGTVALREIRRYQKSTE  163
            MARTKQTARKSTGGKAPRKQLATKAARKSAP+TGGVKKPHRYRPGTVALREIRRYQKSTE
Sbjct  1    MARTKQTARKSTGGKAPRKQLATKAARKSAPATGGVKKPHRYRPGTVALREIRRYQKSTE  60

Query  164  LLIRKLPFQRLVREIAQDFKTDLRFQSAAIGALQEASEAYLVGLFEDTNLCAIHAKRVTI  223
            LLIRKLPFQRLVREIAQDFKTDLRFQS+A+ ALQEASEAYLVGLFEDTNLCAIHAKRVTI
Sbjct  61   LLIRKLPFQRLVREIAQDFKTDLRFQSSAVMALQEASEAYLVGLFEDTNLCAIHAKRVTI  120

Query  224  MPKDIQLARRIRG  236
            MPKDI LARRIRG
Sbjct  121  MPKDIHLARRIRG  133
```

>uncharacterized protein LOC121335966 [Onychostruthus taczanowskii]
Sequence ID: XP_041260510.1 Length: 431
Range 1: 1 to 240

Score:459 bits(1181), Expect:1e-159,
Method:Compositional matrix adjust.,
Identities:233/240(97%), Positives:236/240(98%), Gaps:3/240(1%)

```
Query  1    MSGRGKGGKGLGKGGAKRHRKVLRDNIQGITKPAIRRLARRGGVKRISGLIYEETRGVLK  60
            MSGRGKGGKGLGKGGAKRHRKVLRDNIQGITKPAIRRLARRGGVKRISGLIYEETRGVLK
Sbjct  1    MSGRGKGGKGLGKGGAKRHRKVLRDNIQGITKPAIRRLARRGGVKRISGLIYEETRGVLK  60
```

```
Query  61   VFLENVIRDAVTYTEHAKRKTVTAMDVVYALKRQGRTLYGFGG---MARTKQTARKSTGG  117
            VFLENVIRDAVTYTEHAKRKTVTAMDVVYALKRQGRTLYGFGG   MARTKQTARKSTGG
Sbjct  61   VFLENVIRDAVTYTEHAKRKTVTAMDVVYALKRQGRTLYGFGGGGAMARTKQTARKSTGG  120

Query  118  KAPRKQLATKAARKSAPSTGGVKKPHRYRPGTVALREIRRYQKSTELLIRKLPFQRLVRE  177
            KAPRKQLATKAARKSAP+TGGVKKPHRYRPGTVALREIRRYQKSTELLIRKLPFQRLVRE
Sbjct  121  KAPRKQLATKAARKSAPATGGVKKPHRYRPGTVALREIRRYQKSTELLIRKLPFQRLVRE  180

Query  178  IAQDFKTDLRFQSAAIGALQEASEAYLVGLFEDTNLCAIHAKRVTIMPKDIQLARRIRGE  237
            IAQDFKTDLRFQS+A+ ALQEASEAYLVGLFEDTNLCAIHAKRVTIMPKDIQLARRIRGE
Sbjct  181  IAQDFKTDLRFQSSAVMALQEASEAYLVGLFEDTNLCAIHAKRVTIMPKDIQLARRIRGE  240
```

Range 2: 329 to 431

Score:205 bits(522), Expect:4e-60,
Method:Compositional matrix adjust.,
Identities:103/103(100%), Positives:103/103(100%), Gaps:0/103(0%)

```
Query  1    MSGRGKGGKGLGKGGAKRHRKVLRDNIQGITKPAIRRLARRGGVKRISGLIYEETRGVLK  60
            MSGRGKGGKGLGKGGAKRHRKVLRDNIQGITKPAIRRLARRGGVKRISGLIYEETRGVLK
Sbjct  329  MSGRGKGGKGLGKGGAKRHRKVLRDNIQGITKPAIRRLARRGGVKRISGLIYEETRGVLK  388

Query  61   VFLENVIRDAVTYTEHAKRKTVTAMDVVYALKRQGRTLYGFGG  103
            VFLENVIRDAVTYTEHAKRKTVTAMDVVYALKRQGRTLYGFGG
Sbjct  389  VFLENVIRDAVTYTEHAKRKTVTAMDVVYALKRQGRTLYGFGG  431
```

>uncharacterized protein [Mytilus edulis]
Sequence ID: XP_071151798.1 Length: 365
Range 1: 1 to 262

Score:456 bits(1172), Expect:2e-159,
Method:Compositional matrix adjust.,
Identities:235/262(90%), Positives:238/262(90%), Gaps:23/262(8%)

```
Query  1    MSGRGKGGKGLGKGGAKRHRKVLRDNIQGITKPAIRRLARRGGVKRISGLIYEETRGVLK  60
            MSGRGKGGKGLGKGGAKRHRKVLRDNIQGITKPAIRRLARRGGVKRISGLIYEETRGVLK
Sbjct  1    MSGRGKGGKGLGKGGAKRHRKVLRDNIQGITKPAIRRLARRGGVKRISGLIYEETRGVLK  60

Query  61   VFLENVIRDAVTYTEHAKRKTVTAMDVVYALKRQGRTLYGFGG-----------------  103
            VFLENVIRDAVTYTEHAKRKTVTAMDVVYALKRQGRTLYGFGG
Sbjct  61   VFLENVIRDAVTYTEHAKRKTVTAMDVVYALKRQGRTLYGFGGHKNTFTEIFKYSFCSKS  120

Query  104  ------MARTKQTARKSTGGKAPRKQLATKAARKSAPSTGGVKKPHRYRPGTVALREIRR  157
                  MARTKQTARKSTGGKAPRKQLATKAARKSAP+TGGVKKPHRYRPGTVALREIRR
Sbjct  121  STELIVMARTKQTARKSTGGKAPRKQLATKAARKSAPATGGVKKPHRYRPGTVALREIRR  180

Query  158  YQKSTELLIRKLPFQRLVREIAQDFKTDLRFQSAAIGALQEASEAYLVGLFEDTNLCAIH  217
```

```
           YQKSTELLIRKLPFQRLVREIAQDFKTDLRFQS+A+ ALQEASEAYLVGLFEDTNLCAIH
Sbjct  181  YQKSTELLIRKLPFQRLVREIAQDFKTDLRFQSSAVMALQEASEAYLVGLFEDTNLCAIH  240

Query  218  AKRVTIMPKDIQLARRIRGERA  239
            AKRVTIMPKDIQLARRIRGERA
Sbjct  241  AKRVTIMPKDIQLARRIRGERA  262
```

Range 2: 263 to 365

Score:206 bits(523), Expect:5e-61,
Method:Compositional matrix adjust.,
Identities:103/103(100%), Positives:103/103(100%), Gaps:0/103(0%)

```
Query  1    MSGRGKGGKGLGKGGAKRHRKVLRDNIQGITKPAIRRLARRGGVKRISGLIYEETRGVLK  60
            MSGRGKGGKGLGKGGAKRHRKVLRDNIQGITKPAIRRLARRGGVKRISGLIYEETRGVLK
Sbjct  263  MSGRGKGGKGLGKGGAKRHRKVLRDNIQGITKPAIRRLARRGGVKRISGLIYEETRGVLK  322

Query  61   VFLENVIRDAVTYTEHAKRKTVTAMDVVYALKRQGRTLYGFGG  103
            VFLENVIRDAVTYTEHAKRKTVTAMDVVYALKRQGRTLYGFGG
Sbjct  323  VFLENVIRDAVTYTEHAKRKTVTAMDVVYALKRQGRTLYGFGG  365
```

>uncharacterized protein LOC118180062, partial [Stegodyphus dumicola]
Sequence ID: XP_035205101.1 Length: 412
Range 1: 158 to 409

Score:457 bits(1175), Expect:5e-159,
Method:Compositional matrix adjust.,
Identities:232/252(92%), Positives:235/252(93%), Gaps:14/252(5%)

```
Query  1    MSGRGKGGKGLGKGGAKRHRKVLRDNIQGITKPAIRRLARRGGVKRISGLIYEETRGVLK  60
            MSGRGKGGKGLGKGGAKRHRKVLRDNIQGITKPAIRRLARRGGVKRISGLIYEETRGVLK
Sbjct  158  MSGRGKGGKGLGKGGAKRHRKVLRDNIQGITKPAIRRLARRGGVKRISGLIYEETRGVLK  217

Query  61   VFLENVIRDAVTYTEHAKRKTVTAMDVVYALKRQGRTLYGFGG--------------MAR  106
            VFLENVIRDAVTYTEHAKRKTVTAMDVVYALKRQGRTLYGF                MAR
Sbjct  218  VFLENVIRDAVTYTEHAKRKTVTAMDVVYALKRQGRTLYGFWWRAHFLVLTKSRPLDMAR  277

Query  107  TKQTARKSTGGKAPRKQLATKAARKSAPSTGGVKKPHRYRPGTVALREIRRYQKSTELLI  166
            TKQTARKSTGGKAPRKQLATKAARKSAP+TGGVKKPHRYRPGTVALREIRRYQKSTELLI
Sbjct  278  TKQTARKSTGGKAPRKQLATKAARKSAPATGGVKKPHRYRPGTVALREIRRYQKSTELLI  337

Query  167  RKLPFQRLVREIAQDFKTDLRFQSAAIGALQEASEAYLVGLFEDTNLCAIHAKRVTIMPK  226
            RKLPFQRLVREIAQDFKTDLRFQS+A+ ALQEASEAYLVGLFEDTNLCAIHAKRVTIMPK
Sbjct  338  RKLPFQRLVREIAQDFKTDLRFQSSAVMALQEASEAYLVGLFEDTNLCAIHAKRVTIMPK  397

Query  227  DIQLARRIRGER  238
            DIQLARRIRGER
Sbjct  398  DIQLARRIRGER  409
```

```
Range 2: 1 to 135

Score:266 bits(680), Expect:5e-84,
Method:Compositional matrix adjust.,
Identities:131/135(97%), Positives:134/135(99%), Gaps:0/135(0%)

Query  104  MARTKQTARKSTGGKAPRKQLATKAARKSAPSTGGVKKPHRYRPGTVALREIRRYQKSTE  163
            MARTKQTARKSTGGKAPRKQLATKAARKSAP+TGGVKKPHRYRPGTVALREIRRYQKSTE
Sbjct  1    MARTKQTARKSTGGKAPRKQLATKAARKSAPATGGVKKPHRYRPGTVALREIRRYQKSTE  60

Query  164  LLIRKLPFQRLVREIAQDFKTDLRFQSAAIGALQEASEAYLVGLFEDTNLCAIHAKRVTI  223
            LLIRKLPFQRLVREIAQDFKTDLRFQS+A+ ALQEASEAYLVGLFEDTNLCAIHAKRVTI
Sbjct  61   LLIRKLPFQRLVREIAQDFKTDLRFQSSAVMALQEASEAYLVGLFEDTNLCAIHAKRVTI  120

Query  224  MPKDIQLARRIRGER  238
            MPKDIQLARRIRGER
Sbjct  121  MPKDIQLARRIRGER  135
```

```
>uncharacterized protein LOC134378130 [Cynocephalus volans]
Sequence ID: XP_062953180.1 Length: 503
Range 1: 259 to 503

Score:460 bits(1183), Expect:6e-159,
Method:Compositional matrix adjust.,
Identities:233/245(95%), Positives:236/245(96%), Gaps:6/245(2%)

Query  1    MSGRGKGGKGLGKGGAKRHRKVLRDNIQGITKPAIRRLARRGGVKRISGLIYEETRGVLK  60
            MSGRGKGGKGLGKGGAKRHRKVLRDNIQGITKPAIRRLARRGGVKRISGLIYEETRGVLK
Sbjct  259  MSGRGKGGKGLGKGGAKRHRKVLRDNIQGITKPAIRRLARRGGVKRISGLIYEETRGVLK  318

Query  61   VFLENVIRDAVTYTEHAKRKTVTAMDVVYALKRQGRTLYGFGG------MARTKQTARKS  114
            VFLENVIRDAVTYTEHAKRKTVTAMDVVYALKRQGRTLYGFG       MARTKQTARKS
Sbjct  319  VFLENVIRDAVTYTEHAKRKTVTAMDVVYALKRQGRTLYGFGDPQVCLTMARTKQTARKS  378

Query  115  TGGKAPRKQLATKAARKSAPSTGGVKKPHRYRPGTVALREIRRYQKSTELLIRKLPFQRL  174
            TGGKAPRKQLATKAARKSAP+TGGVKKPHRYRPGTVALREIRRYQKSTELLIRKLPFQRL
Sbjct  379  TGGKAPRKQLATKAARKSAPATGGVKKPHRYRPGTVALREIRRYQKSTELLIRKLPFQRL  438

Query  175  VREIAQDFKTDLRFQSAAIGALQEASEAYLVGLFEDTNLCAIHAKRVTIMPKDIQLARRI  234
            VREIAQDFKTDLRFQS+A+ ALQEA EAYLVGLFEDTNLCAIHAKRVTIMPKDIQLARRI
Sbjct  439  VREIAQDFKTDLRFQSSAVMALQEACEAYLVGLFEDTNLCAIHAKRVTIMPKDIQLARRI  498

Query  235  RGERA  239
            RGERA
Sbjct  499  RGERA  503
```

Range 2: 1 to 223

Score:262 bits(670), Expect:2e-81,
Method:Compositional matrix adjust.,
Identities:150/240(63%), Positives:168/240(70%), Gaps:20/240(8%)

```
Query  1    MSGRGKGGKGLGKGGAKRHRKVLRDNIQGITKPA--IRRLARRGG-VKRISGLIYEETRG  57
            MSGRGK      +GG  R +   R +  G+  P   + RL R+G   +R+
Sbjct  1    MSGRGK------QGGKARAKAKTRSSRAGLQFPVGRVHRLLRKGNYAERVGAGAPVYLAA  54

Query  58   VLKVFLENVIRDAVTYTEHAKRKTVTAMDVVYALKRQGRTLYGFGGMARTKQTARKSTGG  117
            VL+     ++  A       K+  +    +  A             MARTKQTARKSTGG
Sbjct  55   VLEYLTAEILELAGNAARDNKKTRIIPRHLQLAFV-----------MARTKQTARKSTGG  103

Query  118  KAPRKQLATKAARKSAPSTGGVKKPHRYRPGTVALREIRRYQKSTELLIRKLPFQRLVRE  177
            KAPRKQLATKAARKSAP+TGGVKKPHRYRPGTVALREIRRYQKSTELLIRKLPFQRLVRE
Sbjct  104  KAPRKQLATKAARKSAPATGGVKKPHRYRPGTVALREIRRYQKSTELLIRKLPFQRLVRE  163

Query  178  IAQDFKTDLRFQSAAIGALQEASEAYLVGLFEDTNLCAIHAKRVTIMPKDIQLARRIRGE  237
            IAQDFKTDLRFQS+A+ ALQEA EAYLVGLFEDTNLCAIHAKRVTIMPKDIQLARRIRG+
Sbjct  164  IAQDFKTDLRFQSSAVMALQEACEAYLVGLFEDTNLCAIHAKRVTIMPKDIQLARRIRGD  223
```

>uncharacterized protein LOC116968014 [Amblyraja radiata]
Sequence ID: XP_032870537.1 Length: 609
Range 1: 373 to 609

Score:463 bits(1192), Expect:8e-159,
Method:Compositional matrix adjust.,
Identities:231/237(97%), Positives:234/237(98%), Gaps:2/237(0%)

```
Query  5    GKGGKGLGKGGAKRHRKVLRDNIQGITKPAIRRLARRGGVKRISGLIYEETRGVLKVFLE  64
            GKGGKGLGKGGAKRHRKVLRDNIQGITKPAIRRLARRGGVKRISGLIYEETRGVLKVFLE
Sbjct  373  GKGGKGLGKGGAKRHRKVLRDNIQGITKPAIRRLARRGGVKRISGLIYEETRGVLKVFLE  432

Query  65   NVIRDAVTYTEHAKRKTVTAMDVVYALKRQGRTLYGFGG--MARTKQTARKSTGGKAPRK  122
            NVIRDAVTYTEHAKRKTVTAMDVVYALKRQGRTLYGFGG  MARTKQTARKSTGGKAPRK
Sbjct  433  NVIRDAVTYTEHAKRKTVTAMDVVYALKRQGRTLYGFGGSTMARTKQTARKSTGGKAPRK  492

Query  123  QLATKAARKSAPSTGGVKKPHRYRPGTVALREIRRYQKSTELLIRKLPFQRLVREIAQDF  182
            QLATKAARKSAP+TGGVKKPHRYRPGTVALREIRRYQKSTELLIRKLPFQRLVREIAQDF
Sbjct  493  QLATKAARKSAPATGGVKKPHRYRPGTVALREIRRYQKSTELLIRKLPFQRLVREIAQDF  552

Query  183  KTDLRFQSAAIGALQEASEAYLVGLFEDTNLCAIHAKRVTIMPKDIQLARRIRGERA  239
            KTDLRFQS+A+ ALQEASEAYLVGLFEDTNLCAIHAKRVTIMPKDIQLARRIRGERA
Sbjct  553  KTDLRFQSSAVMALQEASEAYLVGLFEDTNLCAIHAKRVTIMPKDIQLARRIRGERA  609
```

Range 2: 1 to 136

Score:270 bits(689), Expect:4e-83,
Method:Compositional matrix adjust.,
Identities:132/136(97%), Positives:135/136(99%), Gaps:0/136(0%)

```
Query  104  MARTKQTARKSTGGKAPRKQLATKAARKSAPSTGGVKKPHRYRPGTVALREIRRYQKSTE  163
            MARTKQTARKSTGGKAPRKQLATKAARKSAP+TGGVKKPHRYRPGTVALREIRRYQKSTE
Sbjct  1    MARTKQTARKSTGGKAPRKQLATKAARKSAPATGGVKKPHRYRPGTVALREIRRYQKSTE  60

Query  164  LLIRKLPFQRLVREIAQDFKTDLRFQSAAIGALQEASEAYLVGLFEDTNLCAIHAKRVTI  223
            LLIRKLPFQRLVREIAQDFKTDLRFQS+A+ ALQEASEAYLVGLFEDTNLCAIHAKRVTI
Sbjct  61   LLIRKLPFQRLVREIAQDFKTDLRFQSSAVMALQEASEAYLVGLFEDTNLCAIHAKRVTI  120

Query  224  MPKDIQLARRIRGERA  239
            MPKDIQLARRIRGERA
Sbjct  121  MPKDIQLARRIRGERA  136
```

>uncharacterized protein LOC133040791 [Dama dama]
Sequence ID: XP_060976928.1 Length: 299
Range 1: 54 to 299

Score:451 bits(1159), Expect:2e-158,
Method:Compositional matrix adjust.,
Identities:230/246(93%), Positives:234/246(95%), Gaps:7/246(2%)

```
Query  1    MSGRGKGGKGLGKGGAKRHRKVLRDNIQGITKPAIRRLARRGGVKRISGLIYEETRGVLK  60
            MSGRGKGGKGLGKGGAKRHRKVLRDNIQGITKPAIRRLARRGGVKRISGLIYEETRGVLK
Sbjct  54   MSGRGKGGKGLGKGGAKRHRKVLRDNIQGITKPAIRRLARRGGVKRISGLIYEETRGVLK  113

Query  61   VFLENVIRDAVTYTEHAKRKTVTAMDVVYALKRQGRTL-------YGFGGMARTKQTARK  113
            VFLENVIRDAVTYTEHAKRKTVTAMDVVYALKRQGRTL         +  MARTKQTARK
Sbjct  114  VFLENVIRDAVTYTEHAKRKTVTAMDVVYALKRQGRTLPSLMHEFASWFAMARTKQTARK  173

Query  114  STGGKAPRKQLATKAARKSAPSTGGVKKPHRYRPGTVALREIRRYQKSTELLIRKLPFQR  173
            STGGKAPRKQLATKAARKSAP+TGGVKKPHRYRPGTVALREIRRYQKSTELLIRKLPFQR
Sbjct  174  STGGKAPRKQLATKAARKSAPATGGVKKPHRYRPGTVALREIRRYQKSTELLIRKLPFQR  233

Query  174  LVREIAQDFKTDLRFQSAAIGALQEASEAYLVGLFEDTNLCAIHAKRVTIMPKDIQLARR  233
            LVREIAQDFKTDLRFQS+A+ ALQEASEAYLVGLFEDTNLCAIHAKRVTIMPKDIQLARR
Sbjct  234  LVREIAQDFKTDLRFQSSAVMALQEASEAYLVGLFEDTNLCAIHAKRVTIMPKDIQLARR  293

Query  234  IRGERA  239
            IRGERA
Sbjct  294  IRGERA  299
```

>histone H3-like centromeric protein A [Pipistrellus kuhlii]
Sequence ID: XP_036297710.2 Length: 275

```
Range 1: 30 to 275

Score:449 bits(1156), Expect:3e-158,
Method:Compositional matrix adjust.,
Identities:230/246(93%), Positives:234/246(95%), Gaps:7/246(2%)

Query  1    MSGRGKGGKGLGKGGAKRHRKVLRDNIQGITKPAIRRLARRGGVKRISGLIYEETRGVLK  60
            MSGRGKGGKGLGKGGAKRHRKVLRDNIQGITKPAIRRLARRGGVKRISGLIYEETRGVLK
Sbjct  30   MSGRGKGGKGLGKGGAKRHRKVLRDNIQGITKPAIRRLARRGGVKRISGLIYEETRGVLK  89

Query  61   VFLENVIRDAVTYTEHAKRKTVTAMDVVYALKRQGRTL----YGFG---GMARTKQTARK  113
            VFLENVIRDAVTYTEHAKRKTVTAMDVVYALKRQGRTL    +       MARTKQTARK
Sbjct  90   VFLENVIRDAVTYTEHAKRKTVTAMDVVYALKRQGRTLPTSIHWLAPRTAMARTKQTARK  149

Query  114  STGGKAPRKQLATKAARKSAPSTGGVKKPHRYRPGTVALREIRRYQKSTELLIRKLPFQR  173
            STGGKAPRKQLATKAARKSAP+TGGVKKPHRYRPGTVALREIRRYQKSTELLIRKLPFQR
Sbjct  150  STGGKAPRKQLATKAARKSAPATGGVKKPHRYRPGTVALREIRRYQKSTELLIRKLPFQR  209

Query  174  LVREIAQDFKTDLRFQSAAIGALQEASEAYLVGLFEDTNLCAIHAKRVTIMPKDIQLARR  233
            LVREIAQDFKTDLRFQS+A+ ALQEASEAYLVGLFEDTNLCAIHAKRVTIMPKDIQLARR
Sbjct  210  LVREIAQDFKTDLRFQSSAVMALQEASEAYLVGLFEDTNLCAIHAKRVTIMPKDIQLARR  269

Query  234  IRGERA  239
            IRGERA
Sbjct  270  IRGERA  275



>uncharacterized protein LOC102419892 [Myotis lucifugus]
Sequence ID: XP_014303395.1 Length: 436
Range 1: 173 to 436

Score:456 bits(1172), Expect:3e-158,
Method:Compositional matrix adjust.,
Identities:234/264(89%), Positives:237/264(89%), Gaps:25/264(9%)

Query  1    MSGRGKGGKGLGKGGAKRHRKVLRDNIQGITKPAIRRLARRGGVKRISGLIYEETRGVLK  60
            MSGRGKGGKGLGKGGAKRHRKVLRDNIQGITKPAIRRLARRGGVKRISGLIYEETRGVLK
Sbjct  173  MSGRGKGGKGLGKGGAKRHRKVLRDNIQGITKPAIRRLARRGGVKRISGLIYEETRGVLK  232

Query  61   VFLENVIRDAVTYTEHAKRKTVTAMDVVYALKRQGRTLYGFGG-----------------  103
            VFLENVIRDAVTYTEHAKRKTVTAMDVVYALKRQGRTLYGFGG
Sbjct  233  VFLENVIRDAVTYTEHAKRKTVTAMDVVYALKRQGRTLYGFGGAIQSERESRYKYRAAGP  292

Query  104  --------MARTKQTARKSTGGKAPRKQLATKAARKSAPSTGGVKKPHRYRPGTVALREI  155
                    MARTKQTARKSTGGKAPRKQLATKAARKSAP+TGGVKKPHRYRPGTVALREI
Sbjct  293  PSLVRSSVMARTKQTARKSTGGKAPRKQLATKAARKSAPATGGVKKPHRYRPGTVALREI  352

Query  156  RRYQKSTELLIRKLPFQRLVREIAQDFKTDLRFQSAAIGALQEASEAYLVGLFEDTNLCA  215
            RRYQKSTELLIRKLPFQRLVREIAQDFKTDLRFQS+A+ ALQEA EAYLVGLFEDTNLCA
Sbjct  353  RRYQKSTELLIRKLPFQRLVREIAQDFKTDLRFQSSAVMALQEACEAYLVGLFEDTNLCA  412

Query  216  IHAKRVTIMPKDIQLARRIRGERA  239
```

```
            IHAKRVTIMPKDIQLARRIRGERA
Sbjct  413  IHAKRVTIMPKDIQLARRIRGERA  436
```

Range 2: 36 to 171

Score:265 bits(678), Expect:2e-83,
Method:Compositional matrix adjust.,
Identities:131/136(96%), Positives:134/136(98%), Gaps:0/136(0%)

```
Query  104  MARTKQTARKSTGGKAPRKQLATKAARKSAPSTGGVKKPHRYRPGTVALREIRRYQKSTE  163
            MARTKQTARKSTGGKAPRKQLATKAARKSAP+TGGVKKPHRYRPGTVALREIRRYQKSTE
Sbjct  36   MARTKQTARKSTGGKAPRKQLATKAARKSAPATGGVKKPHRYRPGTVALREIRRYQKSTE  95

Query  164  LLIRKLPFQRLVREIAQDFKTDLRFQSAAIGALQEASEAYLVGLFEDTNLCAIHAKRVTI  223
            LLIRKLPFQRLVREIAQDFKTDLRFQS+A+ ALQEA EAYLVGLFEDTNLCAIHAKRVTI
Sbjct  96   LLIRKLPFQRLVREIAQDFKTDLRFQSSAVMALQEACEAYLVGLFEDTNLCAIHAKRVTI  155

Query  224  MPKDIQLARRIRGERA  239
            MPKDIQLARRIRGERA
Sbjct  156  MPKDIQLARRIRGERA  171
```

>uncharacterized protein LOC114628358 [Grammomys surdaster]
Sequence ID: XP_028632173.1 Length: 891
Range 1: 1 to 239

Score:471 bits(1212), Expect:4e-158,
Method:Compositional matrix adjust.,
Identities:234/239(98%), Positives:237/239(99%), Gaps:0/239(0%)

```
Query  1    MSGRGKGGKGLGKGGAKRHRKVLRDNIQGITKPAIRRLARRGGVKRISGLIYEETRGVLK  60
            MSGRGKGGKGLGKGGAKRHRKVLRDNIQGITKPAIRRLARRGGVKRISGLIYEETRGVLK
Sbjct  1    MSGRGKGGKGLGKGGAKRHRKVLRDNIQGITKPAIRRLARRGGVKRISGLIYEETRGVLK  60

Query  61   VFLENVIRDAVTYTEHAKRKTVTAMDVVYALKRQGRTLYGFGGMARTKQTARKSTGGKAP  120
            VFLENVIRDAVTYTEHAKRKTVTAMDVVYALKRQGRTLYGFG MARTKQTARKSTGGKAP
Sbjct  61   VFLENVIRDAVTYTEHAKRKTVTAMDVVYALKRQGRTLYGFGVMARTKQTARKSTGGKAP  120

Query  121  RKQLATKAARKSAPSTGGVKKPHRYRPGTVALREIRRYQKSTELLIRKLPFQRLVREIAQ  180
            RKQLATKAARKSAP+TGGVKKPHRYRPGTVALREIRRYQKSTELLIRKLPFQRLVREIAQ
Sbjct  121  RKQLATKAARKSAPATGGVKKPHRYRPGTVALREIRRYQKSTELLIRKLPFQRLVREIAQ  180

Query  181  DFKTDLRFQSAAIGALQEASEAYLVGLFEDTNLCAIHAKRVTIMPKDIQLARRIRGERA  239
            DFKTDLRFQS+A+ ALQEASEAYLVGLFEDTNLCAIHAKRVTIMPKDIQLARRIRGERA
Sbjct  181  DFKTDLRFQSSAVMALQEASEAYLVGLFEDTNLCAIHAKRVTIMPKDIQLARRIRGERA  239
```

Range 2: 789 to 891

Score:209 bits(533), Expect:1e-58,
Method:Compositional matrix adjust.,
Identities:103/103(100%), Positives:103/103(100%), Gaps:0/103(0%)

```
Query  1    MSGRGKGGKGLGKGGAKRHRKVLRDNIQGITKPAIRRLARRGGVKRISGLIYEETRGVLK  60
            MSGRGKGGKGLGKGGAKRHRKVLRDNIQGITKPAIRRLARRGGVKRISGLIYEETRGVLK
Sbjct  789  MSGRGKGGKGLGKGGAKRHRKVLRDNIQGITKPAIRRLARRGGVKRISGLIYEETRGVLK  848

Query  61   VFLENVIRDAVTYTEHAKRKTVTAMDVVYALKRQGRTLYGFGG  103
            VFLENVIRDAVTYTEHAKRKTVTAMDVVYALKRQGRTLYGFGG
Sbjct  849  VFLENVIRDAVTYTEHAKRKTVTAMDVVYALKRQGRTLYGFGG  891
```

Range 3: 685 to 754

Score:139 bits(351), Expect:1e-33,
Method:Compositional matrix adjust.,
Identities:65/70(93%), Positives:68/70(97%), Gaps:0/70(0%)

```
Query  170  PFQRLVREIAQDFKTDLRFQSAAIGALQEASEAYLVGLFEDTNLCAIHAKRVTIMPKDIQ  229
            P +RLVREIAQDFKTDLRFQS+A+ ALQEASEAYLVGLFEDTNLCAIHAKRVTIMPKDIQ
Sbjct  685  PLRRLVREIAQDFKTDLRFQSSAVMALQEASEAYLVGLFEDTNLCAIHAKRVTIMPKDIQ  744

Query  230  LARRIRGERA  239
            LARRIRGERA
Sbjct  745  LARRIRGERA  754
```

>histone H3, embryonic-like [Patiria miniata]
Sequence ID: XP_038066779.1 Length: 271
Range 1: 1 to 271

Score:449 bits(1154), Expect:6e-158,
Method:Compositional matrix adjust.,
Identities:233/271(86%), Positives:237/271(87%), Gaps:32/271(11%)

```
Query  1    MSGRGKGGKGLGKGGAKRHRKVLRDNIQGITKPAIRRLARRGGVKRISGLIYEETRGVLK  60
            MSGRGKGGKGLGKGGAKRHRKVLRDNIQGITKPAIRRLARRGGVKRISGLIYEETRGVLK
Sbjct  1    MSGRGKGGKGLGKGGAKRHRKVLRDNIQGITKPAIRRLARRGGVKRISGLIYEETRGVLK  60

Query  61   VFLENVIRDAVTYTEHAKRKTVTAMDVVYALKRQGRTLYGFGG-----------------  103
            VFLENVIRDAVTY EHAKRKTVTAMDVVYALKRQGRTLYGFGG
Sbjct  61   VFLENVIRDAVTYCEHAKRKTVTAMDVVYALKRQGRTLYGFGGCSIDQSEGPCKGYILRG  120

Query  104  ---------------MARTKQTARKSTGGKAPRKQLATKAARKSAPSTGGVKKPHRYRPG  148
                           MARTKQTARKSTGGKAPRKQLATKAARKSAP+TGGVKKPHRYRPG
Sbjct  121  GFFKFHILFYSKTSKMARTKQTARKSTGGKAPRKQLATKAARKSAPATGGVKKPHRYRPG  180
```

```
Query  149   TVALREIRRYQKSTELLIRKLPFQRLVREIAQDFKTDLRFQSAAIGALQEASEAYLVGLF  208
             TVALREIRRYQKSTELLIRKLPFQRLVREIAQDFKT+LRFQS+A+ ALQEASEAYLVGLF
Sbjct  181   TVALREIRRYQKSTELLIRKLPFQRLVREIAQDFKTELRFQSSAVMALQEASEAYLVGLF  240

Query  209   EDTNLCAIHAKRVTIMPKDIQLARRIRGERA  239
             EDTNLCAIHAKRVTIMPKDIQLARRIRGERA
Sbjct  241   EDTNLCAIHAKRVTIMPKDIQLARRIRGERA  271
```

>uncharacterized protein LOC123609392 [Leopardus geoffroyi]
Sequence ID: XP_045356361.1 Length: 519
Range 1: 267 to 519

Score:457 bits(1177), Expect:9e-158,
Method:Compositional matrix adjust.,
Identities:233/253(92%), Positives:236/253(93%), Gaps:14/253(5%)

```
Query  1     MSGRGKGGKGLGKGGAKRHRKVLRDNIQGITKPAIRRLARRGGVKRISGLIYEETRGVLK  60
             MSGRGKGGKGLGKGGAKRHRKVLRDNIQGITKPAIRRLARRGGVKRISGLIYEETRGVLK
Sbjct  267   MSGRGKGGKGLGKGGAKRHRKVLRDNIQGITKPAIRRLARRGGVKRISGLIYEETRGVLK  326

Query  61    VFLENVIRDAVTYTEHAKRKTVTAMDVVYALKRQGRTLYGFGG--------------MAR  106
             VFLENVIRDAVTYTEHAKRKTVTAMDVVYALKRQGRTLYGFG               MAR
Sbjct  327   VFLENVIRDAVTYTEHAKRKTVTAMDVVYALKRQGRTLYGFGAERRTMSALSLALVVMAR  386

Query  107   TKQTARKSTGGKAPRKQLATKAARKSAPSTGGVKKPHRYRPGTVALREIRRYQKSTELLI  166
             TKQTARKSTGGKAPRKQLATKAARKSAP+TGGVKKPHRYRPGTVALREIRRYQKSTELLI
Sbjct  387   TKQTARKSTGGKAPRKQLATKAARKSAPATGGVKKPHRYRPGTVALREIRRYQKSTELLI  446

Query  167   RKLPFQRLVREIAQDFKTDLRFQSAAIGALQEASEAYLVGLFEDTNLCAIHAKRVTIMPK  226
             RKLPFQRLVREIAQDFKTDLRFQS+A+ ALQEA EAYLVGLFEDTNLCAIHAKRVTIMPK
Sbjct  447   RKLPFQRLVREIAQDFKTDLRFQSSAVMALQEACEAYLVGLFEDTNLCAIHAKRVTIMPK  506

Query  227   DIQLARRIRGERA  239
             DIQLARRIRGERA
Sbjct  507   DIQLARRIRGERA  519
```

Range 2: 1 to 136

Score:265 bits(678), Expect:2e-82,
Method:Compositional matrix adjust.,
Identities:131/136(96%), Positives:134/136(98%), Gaps:0/136(0%)

```
Query  104   MARTKQTARKSTGGKAPRKQLATKAARKSAPSTGGVKKPHRYRPGTVALREIRRYQKSTE  163
             MARTKQTARKSTGGKAPRKQLATKAARKSAP+TGGVKKPHRYRPGTVALREIRRYQKSTE
Sbjct  1     MARTKQTARKSTGGKAPRKQLATKAARKSAPATGGVKKPHRYRPGTVALREIRRYQKSTE  60

Query  164   LLIRKLPFQRLVREIAQDFKTDLRFQSAAIGALQEASEAYLVGLFEDTNLCAIHAKRVTI  223
             LLIRKLPFQRLVREIAQDFKTDLRFQS+A+ ALQEA EAYLVGLFEDTNLCAIHAKRVTI
```

```
Sbjct  61   LLIRKLPFQRLVREIAQDFKTDLRFQSSAVMALQEACEAYLVGLFEDTNLCAIHAKRVTI  120

Query  224  MPKDIQLARRIRGERA  239
            MPKDIQLARRIRGERA
Sbjct  121  MPKDIQLARRIRGERA  136
```

>LOW QUALITY PROTEIN: histone H3.v1-like [Ursus americanus]
Sequence ID: XP_045656319.1 Length: 563
Range 1: 1 to 253

Score:458 bits(1179), Expect:2e-157,
Method:Compositional matrix adjust.,
Identities:234/253(92%), Positives:237/253(93%), Gaps:14/253(5%)

```
Query  1    MSGRGKGGKGLGKGGAKRHRKVLRDNIQGITKPAIRRLARRGGVKRISGLIYEETRGVLK  60
            MSGRGKGGKGLGKGGAKRHRKVLRDNIQGITKPAIRRLARRGGVKRISGLIYEETRGVLK
Sbjct  1    MSGRGKGGKGLGKGGAKRHRKVLRDNIQGITKPAIRRLARRGGVKRISGLIYEETRGVLK  60

Query  61   VFLENVIRDAVTYTEHAKRKTVTAMDVVYALKRQGRTLYGFGG--------------MAR  106
            VFLENVIRDAVTYTEHAKRKTVTAMDVVYALKRQGRTLYGFGG              MAR
Sbjct  61   VFLENVIRDAVTYTEHAKRKTVTAMDVVYALKRQGRTLYGFGGXASTTHHVRPRPNAMAR  120

Query  107  TKQTARKSTGGKAPRKQLATKAARKSAPSTGGVKKPHRYRPGTVALREIRRYQKSTELLI  166
            TKQTARKSTGGKAPRKQLATKAARKSAP+TGGVKKPHRYRPGTVALREIRRYQKSTELLI
Sbjct  121  TKQTARKSTGGKAPRKQLATKAARKSAPATGGVKKPHRYRPGTVALREIRRYQKSTELLI  180

Query  167  RKLPFQRLVREIAQDFKTDLRFQSAAIGALQEASEAYLVGLFEDTNLCAIHAKRVTIMPK  226
            RKLPFQRLVREIAQDFKTDLRFQS+A+ ALQEA EAYLVGLFEDTNLCAIHAKRVTIMPK
Sbjct  181  RKLPFQRLVREIAQDFKTDLRFQSSAVMALQEACEAYLVGLFEDTNLCAIHAKRVTIMPK  240

Query  227  DIQLARRIRGERA  239
            DIQLARRIRGERA
Sbjct  241  DIQLARRIRGERA  253
```

Range 2: 305 to 440

Score:265 bits(677), Expect:9e-82,
Method:Compositional matrix adjust.,
Identities:131/136(96%), Positives:134/136(98%), Gaps:0/136(0%)

```
Query  104  MARTKQTARKSTGGKAPRKQLATKAARKSAPSTGGVKKPHRYRPGTVALREIRRYQKSTE  163
            MARTKQTARKSTGGKAPRKQLATKAARKSAP+TGGVKKPHRYRPGTVALREIRRYQKSTE
Sbjct  305  MARTKQTARKSTGGKAPRKQLATKAARKSAPATGGVKKPHRYRPGTVALREIRRYQKSTE  364

Query  164  LLIRKLPFQRLVREIAQDFKTDLRFQSAAIGALQEASEAYLVGLFEDTNLCAIHAKRVTI  223
            LLIRKLPFQRLVREIAQDFKTDLRFQS+A+ ALQEA EAYLVGLFEDTNLCAIHAKRVTI
Sbjct  365  LLIRKLPFQRLVREIAQDFKTDLRFQSSAVMALQEACEAYLVGLFEDTNLCAIHAKRVTI  424
```

```
Query  224  MPKDIQLARRIRGERA  239
            MPKDIQLARRIRGERA
Sbjct  425  MPKDIQLARRIRGERA  440
```

Range 3: 462 to 563

Score:204 bits(518), Expect:3e-58,
Method:Compositional matrix adjust.,
Identities:101/102(99%), Positives:101/102(99%), Gaps:0/102(0%)

```
Query  2    SGRGKGGKGLGKGGAKRHRKVLRDNIQGITKPAIRRLARRGGVKRISGLIYEETRGVLKV  61
             GRGKGGKGLGKGGAKRHRKVLRDNIQGITKPAIRRLARRGGVKRISGLIYEETRGVLKV
Sbjct  462  CGRGKGGKGLGKGGAKRHRKVLRDNIQGITKPAIRRLARRGGVKRISGLIYEETRGVLKV  521

Query  62   FLENVIRDAVTYTEHAKRKTVTAMDVVYALKRQGRTLYGFGG  103
            FLENVIRDAVTYTEHAKRKTVTAMDVVYALKRQGRTLYGFGG
Sbjct  522  FLENVIRDAVTYTEHAKRKTVTAMDVVYALKRQGRTLYGFGG  563
```

>LOW QUALITY PROTEIN: uncharacterized protein LOC129974918 [Argiope bruennichi]
Sequence ID: XP_055943683.1 Length: 344
Range 1: 1 to 240

Score:448 bits(1153), Expect:8e-157,
Method:Compositional matrix adjust.,
Identities:229/240(95%), Positives:232/240(96%), Gaps:3/240(1%)

```
Query  1    MSGRGKGGKGLGKGGAKRHRKVLRDNIQGITKPAIRRLARRGGVKRISGLIYEETRGVLK  60
            MSGRGKGGKGLGKGGAKRHRKVLRDNIQGITKPAIRRLARRGGVKRISGLIYEETRGVLK
Sbjct  1    MSGRGKGGKGLGKGGAKRHRKVLRDNIQGITKPAIRRLARRGGVKRISGLIYEETRGVLK  60

Query  61   VFLENVIRDAVTYTEHAKRKTVTAMDVVYALKRQGRTLYGF---GGMARTKQTARKSTGG  117
            VFLENVIRDAVTYTEHAKRKTVTAMDVVYALKRQGRTLY       MARTKQTARKSTGG
Sbjct  61   VFLENVIRDAVTYTEHAKRKTVTAMDVVYALKRQGRTLYVVXEXSVMARTKQTARKSTGG  120

Query  118  KAPRKQLATKAARKSAPSTGGVKKPHRYRPGTVALREIRRYQKSTELLIRKLPFQRLVRE  177
            KAPRKQLATKAARKSAP+TGGVKKPHRYRPGTVALREIRRYQKSTELLIRKLPFQRLVRE
Sbjct  121  KAPRKQLATKAARKSAPATGGVKKPHRYRPGTVALREIRRYQKSTELLIRKLPFQRLVRE  180

Query  178  IAQDFKTDLRFQSAAIGALQEASEAYLVGLFEDTNLCAIHAKRVTIMPKDIQLARRIRGE  237
            IAQDFKTDLRFQS+A+ ALQEASEAYLVGLFEDTNLCAIHAKRVTIMPKDIQLARRIRGE
Sbjct  181  IAQDFKTDLRFQSSAVMALQEASEAYLVGLFEDTNLCAIHAKRVTIMPKDIQLARRIRGE  240
```

Range 2: 242 to 344

Score:206 bits(524), Expect:2e-61,

```
Method:Compositional matrix adjust.,
Identities:103/103(100%), Positives:103/103(100%), Gaps:0/103(0%)

Query  1    MSGRGKGGKGLGKGGAKRHRKVLRDNIQGITKPAIRRLARRGGVKRISGLIYEETRGVLK  60
            MSGRGKGGKGLGKGGAKRHRKVLRDNIQGITKPAIRRLARRGGVKRISGLIYEETRGVLK
Sbjct  242  MSGRGKGGKGLGKGGAKRHRKVLRDNIQGITKPAIRRLARRGGVKRISGLIYEETRGVLK  301

Query  61   VFLENVIRDAVTYTEHAKRKTVTAMDVVYALKRQGRTLYGFGG  103
            VFLENVIRDAVTYTEHAKRKTVTAMDVVYALKRQGRTLYGFGG
Sbjct  302  VFLENVIRDAVTYTEHAKRKTVTAMDVVYALKRQGRTLYGFGG  344
```

```
>uncharacterized protein LOC128748062 [Synchiropus splendidus]
Sequence ID: XP_053702428.1 Length: 468
Range 1: 1 to 248

Score:452 bits(1162), Expect:3e-156,
Method:Compositional matrix adjust.,
Identities:231/248(93%), Positives:235/248(94%), Gaps:9/248(3%)

Query  1    MSGRGKGGKGLGKGGAKRHRKVLRDNIQGITKPAIRRLARRGGVKRISGLIYEETRGVLK  60
            MSGRGKGGKGLGKGGAKRHRKVLRDNIQGITKPAIRRLARRGGVKRISGLIYEETRGVLK
Sbjct  1    MSGRGKGGKGLGKGGAKRHRKVLRDNIQGITKPAIRRLARRGGVKRISGLIYEETRGVLK  60

Query  61   VFLENVIRDAVTYTEHAKRKTVTAMDVVYALKRQGRTL------YGFGG---MARTKQTA  111
            VFLENVIRDAVTYTEHAKRKTVTAMDVVYALKRQGRTL        F G   MARTKQTA
Sbjct  61   VFLENVIRDAVTYTEHAKRKTVTAMDVVYALKRQGRTLPAHTLFLAFNGRVEMARTKQTA  120

Query  112  RKSTGGKAPRKQLATKAARKSAPSTGGVKKPHRYRPGTVALREIRRYQKSTELLIRKLPF  171
            RKSTGGKAPRKQLATKAARKSAP+TGGVKKPHRYRPGTVALREIRRYQKSTELLIRKLPF
Sbjct  121  RKSTGGKAPRKQLATKAARKSAPATGGVKKPHRYRPGTVALREIRRYQKSTELLIRKLPF  180

Query  172  QRLVREIAQDFKTDLRFQSAAIGALQEASEAYLVGLFEDTNLCAIHAKRVTIMPKDIQLA  231
            QRLVREIAQDFKTDLRFQS+A+ ALQEASEAYLVGLFEDTNLCAIHAKRVTIMPKDIQLA
Sbjct  181  QRLVREIAQDFKTDLRFQSSAVMALQEASEAYLVGLFEDTNLCAIHAKRVTIMPKDIQLA  240

Query  232  RRIRGERA  239
            RRIRGER+
Sbjct  241  RRIRGERS  248
```

```
Range 2: 366 to 468

Score:208 bits(529), Expect:1e-60,
Method:Compositional matrix adjust.,
Identities:103/103(100%), Positives:103/103(100%), Gaps:0/103(0%)

Query  1    MSGRGKGGKGLGKGGAKRHRKVLRDNIQGITKPAIRRLARRGGVKRISGLIYEETRGVLK  60
            MSGRGKGGKGLGKGGAKRHRKVLRDNIQGITKPAIRRLARRGGVKRISGLIYEETRGVLK
Sbjct  366  MSGRGKGGKGLGKGGAKRHRKVLRDNIQGITKPAIRRLARRGGVKRISGLIYEETRGVLK  425
```

```
Query  61   VFLENVIRDAVTYTEHAKRKTVTAMDVVYALKRQGRTLYGFGG  103
            VFLENVIRDAVTYTEHAKRKTVTAMDVVYALKRQGRTLYGFGG
Sbjct  426  VFLENVIRDAVTYTEHAKRKTVTAMDVVYALKRQGRTLYGFGG  468
```

>uncharacterized protein LOC125908486 [Panthera uncia]
Sequence ID: XP_049467286.1 Length: 521
Range 1: 261 to 521

Score:454 bits(1167), Expect:3e-156,
Method:Compositional matrix adjust.,
Identities:233/261(89%), Positives:236/261(90%), Gaps:22/261(8%)

```
Query  1    MSGRGKGGKGLGKGGAKRHRKVLRDNIQGITKPAIRRLARRGGVKRISGLIYEETRGVLK  60
            MSGRGKGGKGLGKGGAKRHRKVLRDNIQGITKPAIRRLARRGGVKRISGLIYEETRGVLK
Sbjct  261  MSGRGKGGKGLGKGGAKRHRKVLRDNIQGITKPAIRRLARRGGVKRISGLIYEETRGVLK  320

Query  61   VFLENVIRDAVTYTEHAKRKTVTAMDVVYALKRQGRTLYGFGG-----------------  103
            VFLENVIRDAVTYTEHAKRKTVTAMDVVYALKRQGRTLYGFG
Sbjct  321  VFLENVIRDAVTYTEHAKRKTVTAMDVVYALKRQGRTLYGFGEIGGRGGKTLPVLNGLNK  380

Query  104  -----MARTKQTARKSTGGKAPRKQLATKAARKSAPSTGGVKKPHRYRPGTVALREIRRY  158
                 MARTKQTARKSTGGKAPRKQLATKAARKSAP+TGGVKKPHRYRPGTVALREIRRY
Sbjct  381  LALVVMARTKQTARKSTGGKAPRKQLATKAARKSAPATGGVKKPHRYRPGTVALREIRRY  440

Query  159  QKSTELLIRKLPFQRLVREIAQDFKTDLRFQSAAIGALQEASEAYLVGLFEDTNLCAIHA  218
            QKSTELLIRKLPFQRLVREIAQDFKTDLRFQS+A+ ALQEA EAYLVGLFEDTNLCAIHA
Sbjct  441  QKSTELLIRKLPFQRLVREIAQDFKTDLRFQSSAVMALQEACEAYLVGLFEDTNLCAIHA  500

Query  219  KRVTIMPKDIQLARRIRGERA  239
            KRVTIMPKDIQLARRIRGERA
Sbjct  501  KRVTIMPKDIQLARRIRGERA  521
```

Range 2: 1 to 136

Score:265 bits(677), Expect:3e-82,
Method:Compositional matrix adjust.,
Identities:131/136(96%), Positives:134/136(98%), Gaps:0/136(0%)

```
Query  104  MARTKQTARKSTGGKAPRKQLATKAARKSAPSTGGVKKPHRYRPGTVALREIRRYQKSTE  163
            MARTKQTARKSTGGKAPRKQLATKAARKSAP+TGGVKKPHRYRPGTVALREIRRYQKSTE
Sbjct  1    MARTKQTARKSTGGKAPRKQLATKAARKSAPATGGVKKPHRYRPGTVALREIRRYQKSTE  60

Query  164  LLIRKLPFQRLVREIAQDFKTDLRFQSAAIGALQEASEAYLVGLFEDTNLCAIHAKRVTI  223
            LLIRKLPFQRLVREIAQDFKTDLRFQS+A+ ALQEA EAYLVGLFEDTNLCAIHAKRVTI
Sbjct  61   LLIRKLPFQRLVREIAQDFKTDLRFQSSAVMALQEACEAYLVGLFEDTNLCAIHAKRVTI  120

Query  224  MPKDIQLARRIRGERA  239
```

```
             MPKDIQLARRIRGERA
Sbjct  121   MPKDIQLARRIRGERA  136
```

>uncharacterized protein LOC123826658 [Phyllostomus hastatus]
Sequence ID: XP_045706646.1 Length: 463
Range 1: 224 to 463

Score:451 bits(1160), Expect:5e-156,
Method:Compositional matrix adjust.,
Identities:228/240(95%), Positives:232/240(96%), Gaps:6/240(2%)

```
Query  6     KGGKGLGKGGAKRHRKVLRDNIQGITKPAIRRLARRGGVKRISGLIYEETRGVLKVFLEN  65
             +GGKGLGKGGAKRHRKVLRDNIQGITKPAIRRLARRGGVKRISGLIYEETRGVLKVFLEN
Sbjct  224   RGGKGLGKGGAKRHRKVLRDNIQGITKPAIRRLARRGGVKRISGLIYEETRGVLKVFLEN  283

Query  66    VIRDAVTYTEHAKRKTVTAMDVVYALKRQGRTLYGFGG------MARTKQTARKSTGGKA  119
             VIRDAVTYTEHAKRKTVTAMDVVYALKRQGRTLYGFGG      MARTKQTARKSTGGKA
Sbjct  284   VIRDAVTYTEHAKRKTVTAMDVVYALKRQGRTLYGFGGCCFRVDMARTKQTARKSTGGKA  343

Query  120   PRKQLATKAARKSAPSTGGVKKPHRYRPGTVALREIRRYQKSTELLIRKLPFQRLVREIA  179
             PRKQLATKAARKSAP+TGGVKKPHRYRPGTVALREIRRYQKSTELLIRKLPFQRLVREIA
Sbjct  344   PRKQLATKAARKSAPATGGVKKPHRYRPGTVALREIRRYQKSTELLIRKLPFQRLVREIA  403

Query  180   QDFKTDLRFQSAAIGALQEASEAYLVGLFEDTNLCAIHAKRVTIMPKDIQLARRIRGERA  239
             QDFKTDLRFQS+A+ ALQEA EAYLVGLFEDTNLCAIHAKRVTIMPKDIQLARRIRGERA
Sbjct  404   QDFKTDLRFQSSAVMALQEACEAYLVGLFEDTNLCAIHAKRVTIMPKDIQLARRIRGERA  463
```

Range 2: 1 to 220

Score:258 bits(658), Expect:4e-80,
Method:Compositional matrix adjust.,
Identities:148/237(62%), Positives:166/237(70%), Gaps:20/237(8%)

```
Query  1     MSGRGKGGKGLGKGGAKRHRKVLRDNIQGITKPA--IRRLARRGG-VKRISGLIYEETRG  57
             MSGRGK      +GG  R +   R +  G+  P   + RL R+G   +R+
Sbjct  1     MSGRGK------QGGKARAKAKTRSSRAGLQFPVGRVHRLLRKGNYAERVGAGAPVYLAA  54

Query  58    VLKVFLENVIRDAVTYTEHAKRKTVTAMDVVYALKRQGRTLYGFGGMARTKQTARKSTGG  117
             VL+     ++  A       K+  +    +  A+            MARTKQTARKSTGG
Sbjct  55    VLEYLTAEILELAGNAARDNKKTRIIPRHLQLAIL-----------MARTKQTARKSTGG  103

Query  118   KAPRKQLATKAARKSAPSTGGVKKPHRYRPGTVALREIRRYQKSTELLIRKLPFQRLVRE  177
             KAPRKQLATKAARKSAP+TGGVKKPHRYRPGTVALREIRRYQKSTELLIRKLPFQRLVRE
Sbjct  104   KAPRKQLATKAARKSAPATGGVKKPHRYRPGTVALREIRRYQKSTELLIRKLPFQRLVRE  163

Query  178   IAQDFKTDLRFQSAAIGALQEASEAYLVGLFEDTNLCAIHAKRVTIMPKDIQLARRI  234
             IAQDFKTDLRFQS+A+ ALQEA EAYLVGLFEDTNLCAIHAKRVTIMPKDIQLARRI
Sbjct  164   IAQDFKTDLRFQSSAVMALQEACEAYLVGLFEDTNLCAIHAKRVTIMPKDIQLARRI  220
```

>uncharacterized protein LOC121063867 [Cygnus olor]
Sequence ID: XP_040400708.1 Length: 687
Range 1: 1 to 243

Score:458 bits(1179), Expect:1e-155,
Method:Compositional matrix adjust.,
Identities:232/243(95%), Positives:235/243(96%), Gaps:6/243(2%)

```
Query  1    MSGRGKGGKGLGKGGAKRHRKVLRDNIQGITKPAIRRLARRGGVKRISGLIYEETRGVLK  60
            MSGRGKGGKGLGKGGAKRHRKVLRDNIQGITKPAIRRLARRGGVKRISGLIYEETRGVLK
Sbjct  1    MSGRGKGGKGLGKGGAKRHRKVLRDNIQGITKPAIRRLARRGGVKRISGLIYEETRGVLK  60

Query  61   VFLENVIRDAVTYTEHAKRKTVTAMDVVYALKRQGRTLYGFGG------MARTKQTARKS  114
            VFLENVIRDAVTYTEHAKRKTVTAMDVVYALKRQGRTLYGFG       MARTKQTARKS
Sbjct  61   VFLENVIRDAVTYTEHAKRKTVTAMDVVYALKRQGRTLYGFGEKALRSEMARTKQTARKS  120

Query  115  TGGKAPRKQLATKAARKSAPSTGGVKKPHRYRPGTVALREIRRYQKSTELLIRKLPFQRL  174
            TGGKAPRKQLATKAARKSAP+TGGVKKPHRYRPGTVALREIRRYQKSTELLIRKLPFQRL
Sbjct  121  TGGKAPRKQLATKAARKSAPATGGVKKPHRYRPGTVALREIRRYQKSTELLIRKLPFQRL  180

Query  175  VREIAQDFKTDLRFQSAAIGALQEASEAYLVGLFEDTNLCAIHAKRVTIMPKDIQLARRI  234
            VREIAQDFKTDLRFQS+A+ ALQEASEAYLVGLFEDTNLCAIHAKRVTIMPKDIQLARRI
Sbjct  181  VREIAQDFKTDLRFQSSAVMALQEASEAYLVGLFEDTNLCAIHAKRVTIMPKDIQLARRI  240

Query  235  RGE  237
            RGE
Sbjct  241  RGE  243
```

Range 2: 369 to 506

Score:268 bits(685), Expect:8e-82,
Method:Compositional matrix adjust.,
Identities:133/138(96%), Positives:136/138(98%), Gaps:0/138(0%)

```
Query  102  GGMARTKQTARKSTGGKAPRKQLATKAARKSAPSTGGVKKPHRYRPGTVALREIRRYQKS  161
            G MARTKQTARKSTGGKAPRKQLATKAARKSAP+TGGVKKPHRYRPGTVALREIRRYQKS
Sbjct  369  GEMARTKQTARKSTGGKAPRKQLATKAARKSAPATGGVKKPHRYRPGTVALREIRRYQKS  428

Query  162  TELLIRKLPFQRLVREIAQDFKTDLRFQSAAIGALQEASEAYLVGLFEDTNLCAIHAKRV  221
            TELLIRKLPFQRLVREIAQDFKTDLRFQS+A+ ALQEASEAYLVGLFEDTNLCAIHAKRV
Sbjct  429  TELLIRKLPFQRLVREIAQDFKTDLRFQSSAVMALQEASEAYLVGLFEDTNLCAIHAKRV  488

Query  222  TIMPKDIQLARRIRGERA  239
            TIMPKDIQLARRIRGERA
Sbjct  489  TIMPKDIQLARRIRGERA  506
```

```
Range 3: 585 to 687

Score:208 bits(529), Expect:5e-59,
Method:Compositional matrix adjust.,
Identities:103/103(100%), Positives:103/103(100%), Gaps:0/103(0%)

Query  1    MSGRGKGGKGLGKGGAKRHRKVLRDNIQGITKPAIRRLARRGGVKRISGLIYEETRGVLK  60
            MSGRGKGGKGLGKGGAKRHRKVLRDNIQGITKPAIRRLARRGGVKRISGLIYEETRGVLK
Sbjct  585  MSGRGKGGKGLGKGGAKRHRKVLRDNIQGITKPAIRRLARRGGVKRISGLIYEETRGVLK  644

Query  61   VFLENVIRDAVTYTEHAKRKTVTAMDVVYALKRQGRTLYGFGG  103
            VFLENVIRDAVTYTEHAKRKTVTAMDVVYALKRQGRTLYGFGG
Sbjct  645  VFLENVIRDAVTYTEHAKRKTVTAMDVVYALKRQGRTLYGFGG  687
```

```
>hypothetical protein EK904_001702 [Melospiza melodia maxima]
Sequence ID: KAF2974834.1 Length: 317
Range 1: 87 to 317

Score:444 bits(1143), Expect:1e-155,
Method:Compositional matrix adjust.,
Identities:226/239(95%), Positives:229/239(95%), Gaps:8/239(3%)

Query  1    MSGRGKGGKGLGKGGAKRHRKVLRDNIQGITKPAIRRLARRGGVKRISGLIYEETRGVLK  60
            MSGRGKGGKGLGKGGAKRHRKVLRDNIQGITKPAIRRLARRGGVKRISGLIYEETRGVLK
Sbjct  87   MSGRGKGGKGLGKGGAKRHRKVLRDNIQGITKPAIRRLARRGGVKRISGLIYEETRGVLK  146

Query  61   VFLENVIRDAVTYTEHAKRKTVTAMDVVYALKRQGRTLYGFGGMARTKQTARKSTGGKAP  120
            VFLENVIRDAVTYTEHAKRKTVTAMDVVYALKRQ         MARTKQTARKSTGGKAP
Sbjct  147  VFLENVIRDAVTYTEHAKRKTVTAMDVVYALKRQA--------MARTKQTARKSTGGKAP  198

Query  121  RKQLATKAARKSAPSTGGVKKPHRYRPGTVALREIRRYQKSTELLIRKLPFQRLVREIAQ  180
            RKQLATKAARKSAP+TGGVKKPHRYRPGTVALREIRRYQKSTELLIRKLPFQRLVREIAQ
Sbjct  199  RKQLATKAARKSAPATGGVKKPHRYRPGTVALREIRRYQKSTELLIRKLPFQRLVREIAQ  258

Query  181  DFKTDLRFQSAAIGALQEASEAYLVGLFEDTNLCAIHAKRVTIMPKDIQLARRIRGERA  239
            DFKTDLRFQS+A+ ALQEASEAYLVGLFEDTNLCAIHAKRVTIMPKDIQLARRIRGERA
Sbjct  259  DFKTDLRFQSSAVMALQEASEAYLVGLFEDTNLCAIHAKRVTIMPKDIQLARRIRGERA  317
```

```
>uncharacterized protein LOC129753383 [Uranotaenia lowii]
Sequence ID: XP_055605175.1 Length: 730
Range 1: 485 to 730

Score:457 bits(1177), Expect:6e-155,
Method:Compositional matrix adjust.,
Identities:232/246(94%), Positives:236/246(95%), Gaps:7/246(2%)
```

```
Query  1    MSGRGKGGKGLGKGGAKRHRKVLRDNIQGITKPAIRRLARRGGVKRISGLIYEETRGVLK  60
            M+GRGKGGKGLGKGGAKRHRKVLRDNIQGITKPAIRRLARRGGVKRISGLIYEETRGVLK
Sbjct  485  MTGRGKGGKGLGKGGAKRHRKVLRDNIQGITKPAIRRLARRGGVKRISGLIYEETRGVLK  544

Query  61   VFLENVIRDAVTYTEHAKRKTVTAMDVVYALKRQGRTLYG----FGG---MARTKQTARK  113
            VFLENVIRDAVTYTEHAKRKTVTAMDVVYALKRQGRTL      FGG   MARTKQTARK
Sbjct  545  VFLENVIRDAVTYTEHAKRKTVTAMDVVYALKRQGRTLPTACRVFGGRTTMARTKQTARK  604

Query  114  STGGKAPRKQLATKAARKSAPSTGGVKKPHRYRPGTVALREIRRYQKSTELLIRKLPFQR  173
            STGGKAPRKQLATKAARKSAP+TGGVKKPHRYRPGTVALREIRRYQKSTELLIRKLPFQR
Sbjct  605  STGGKAPRKQLATKAARKSAPATGGVKKPHRYRPGTVALREIRRYQKSTELLIRKLPFQR  664

Query  174  LVREIAQDFKTDLRFQSAAIGALQEASEAYLVGLFEDTNLCAIHAKRVTIMPKDIQLARR  233
            LVREIAQDFKTDLRFQS+A+ ALQEASEAYLVGLFEDTNLCAIHAKRVTIMPKDIQLARR
Sbjct  665  LVREIAQDFKTDLRFQSSAVMALQEASEAYLVGLFEDTNLCAIHAKRVTIMPKDIQLARR  724

Query  234  IRGERA  239
            IRGERA
Sbjct  725  IRGERA  730
```

Range 2: 1 to 134

Score:267 bits(682), Expect:5e-81,
Method:Compositional matrix adjust.,
Identities:130/134(97%), Positives:133/134(99%), Gaps:0/134(0%)

```
Query  104  MARTKQTARKSTGGKAPRKQLATKAARKSAPSTGGVKKPHRYRPGTVALREIRRYQKSTE  163
            MARTKQTARKSTGGKAPRKQLATKAARKSAP+TGGVKKPHRYRPGTVALREIRRYQKSTE
Sbjct  1    MARTKQTARKSTGGKAPRKQLATKAARKSAPATGGVKKPHRYRPGTVALREIRRYQKSTE  60

Query  164  LLIRKLPFQRLVREIAQDFKTDLRFQSAAIGALQEASEAYLVGLFEDTNLCAIHAKRVTI  223
            LLIRKLPFQRLVREIAQDFKTDLRFQS+A+ ALQEASEAYLVGLFEDTNLCAIHAKRVTI
Sbjct  61   LLIRKLPFQRLVREIAQDFKTDLRFQSSAVMALQEASEAYLVGLFEDTNLCAIHAKRVTI  120

Query  224  MPKDIQLARRIRGE  237
            MPKDIQLARRIRGE
Sbjct  121  MPKDIQLARRIRGE  134
```

>LOW QUALITY PROTEIN: uncharacterized protein LOC127042746 [Gopherus flavomarginatus]
Sequence ID: XP_050791933.1 Length: 743
Range 1: 1 to 250

Score:457 bits(1177), Expect:9e-155,
Method:Compositional matrix adjust.,
Identities:230/250(92%), Positives:233/250(93%), Gaps:12/250(4%)

```
Query  1    MSGRGKGGKGLGKGGAKRHRKVLRDNIQGITKPAIRRLARRGGVKRISGLIYEETRGVLK  60
```

```
            MSGRGKGGKGLGKGGAKRHRKVLRDNIQGITKPAIRRLARRGGVKRISGLIYEETRGVLK
Sbjct  1    MSGRGKGGKGLGKGGAKRHRKVLRDNIQGITKPAIRRLARRGGVKRISGLIYEETRGVLK  60

Query  61   VFLENVIRDAVTYTEHAKRKTVTAMDVVYALKRQGRTLYG------------FGGMARTK  108
            VFLENVIRDAVTYTEHAKRKTVTAMDVVYALKRQGRTL G               MARTK
Sbjct  61   VFLENVIRDAVTYTEHAKRKTVTAMDVVYALKRQGRTLPGRFHVPGAGRANXLCRMARTK  120

Query  109  QTARKSTGGKAPRKQLATKAARKSAPSTGGVKKPHRYRPGTVALREIRRYQKSTELLIRK  168
            QTARKSTGGKAPRKQLATKAARKSAP+TGGVKKPHRYRPGTVALREIRRYQKSTELLIRK
Sbjct  121  QTARKSTGGKAPRKQLATKAARKSAPATGGVKKPHRYRPGTVALREIRRYQKSTELLIRK  180

Query  169  LPFQRLVREIAQDFKTDLRFQSAAIGALQEASEAYLVGLFEDTNLCAIHAKRVTIMPKDI  228
            LPFQRLVREIAQDFKTDLRFQS+A+ ALQEASEAYLVGLFEDTNLCAIHAKRVTIMPKDI
Sbjct  181  LPFQRLVREIAQDFKTDLRFQSSAVMALQEASEAYLVGLFEDTNLCAIHAKRVTIMPKDI  240

Query  229  QLARRIRGER  238
            QLARRIRGER
Sbjct  241  QLARRIRGER  250
```

Range 2: 561 to 743

Score:278 bits(710), Expect:6e-85,
Method:Compositional matrix adjust.,
Identities:143/193(74%), Positives:158/193(81%), Gaps:14/193(7%)

```
Query  51   IYEETRGVLKVFLENVIR----DAVTYTEHAKRKTVTAMDVVYALKRQGRTLYGFGGMAR  106
            I  +  G++  F+ ++      +A     + KR T+T+ ++  AL R          MAR
Sbjct  561  ISSKAMGIMNSFVNDIFERIAGEASRLAHYNKRSTITSREIQTALVR----------MAR  610

Query  107  TKQTARKSTGGKAPRKQLATKAARKSAPSTGGVKKPHRYRPGTVALREIRRYQKSTELLI  166
            TKQTARKSTGGKAPRKQLATKAARKSAP+TGGVKKPHRYRPGTVALREIRRYQKSTELLI
Sbjct  611  TKQTARKSTGGKAPRKQLATKAARKSAPATGGVKKPHRYRPGTVALREIRRYQKSTELLI  670

Query  167  RKLPFQRLVREIAQDFKTDLRFQSAAIGALQEASEAYLVGLFEDTNLCAIHAKRVTIMPK  226
            RKLPFQRLVREIAQDFKTDLRFQS+A+ ALQEASEAYLVGLFEDTNLCAIHAKRVTIMPK
Sbjct  671  RKLPFQRLVREIAQDFKTDLRFQSSAVMALQEASEAYLVGLFEDTNLCAIHAKRVTIMPK  730

Query  227  DIQLARRIRGERA  239
            DIQLARRIRGERA
Sbjct  731  DIQLARRIRGERA  743
```

>uncharacterized protein LOC134722518 [Mytilus trossulus]
Sequence ID: XP_063442209.1 Length: 374
Range 1: 136 to 374

Score:444 bits(1143), Expect:1e-154,
Method:Compositional matrix adjust.,
Identities:226/239(95%), Positives:229/239(95%), Gaps:3/239(1%)

```
Query  4    RGKGGKGLGKGGAKRHRKVLRDNIQGITKPAIRRLARRGGVKRISGLIYEETRGVLKVFL  63
            RGKGGKGLGKGGAKRHRKVLRDNIQGITKPAIRRLARRGGVKRISGLIYEETRGVLKVFL
Sbjct  136  RGKGGKGLGKGGAKRHRKVLRDNIQGITKPAIRRLARRGGVKRISGLIYEETRGVLKVFL  195

Query  64   ENVIRDAVTYTEHAKRKTVTAMDVVYALKRQGRTLYGFGG---MARTKQTARKSTGGKAP  120
            ENVIRDAVTYTEHAKRKTVTAMDVVYALKRQGRT         MARTKQTARKSTGGKAP
Sbjct  196  ENVIRDAVTYTEHAKRKTVTAMDVVYALKRQGRTFKSSTELIIMARTKQTARKSTGGKAP  255

Query  121  RKQLATKAARKSAPSTGGVKKPHRYRPGTVALREIRRYQKSTELLIRKLPFQRLVREIAQ  180
             RKQLATKAARKSAP+TGGVKKPHRYRPGTVALREIRRYQKSTELLIRKLPFQRLVREIAQ
Sbjct  256  RKQLATKAARKSAPATGGVKKPHRYRPGTVALREIRRYQKSTELLIRKLPFQRLVREIAQ  315

Query  181  DFKTDLRFQSAAIGALQEASEAYLVGLFEDTNLCAIHAKRVTIMPKDIQLARRIRGERA  239
            DFKTDLRFQS+A+ ALQEASEAYLVGLFEDTNLCAIHAKRVTIMPKDIQLARRIRGERA
Sbjct  316  DFKTDLRFQSSAVMALQEASEAYLVGLFEDTNLCAIHAKRVTIMPKDIQLARRIRGERA  374
```

Range 2: 1 to 135

Score:266 bits(681), Expect:1e-84,
Method:Compositional matrix adjust.,
Identities:131/135(97%), Positives:134/135(99%), Gaps:0/135(0%)

```
Query  104  MARTKQTARKSTGGKAPRKQLATKAARKSAPSTGGVKKPHRYRPGTVALREIRRYQKSTE  163
            MARTKQTARKSTGGKAPRKQLATKAARKSAP+TGGVKKPHRYRPGTVALREIRRYQKSTE
Sbjct  1    MARTKQTARKSTGGKAPRKQLATKAARKSAPATGGVKKPHRYRPGTVALREIRRYQKSTE  60

Query  164  LLIRKLPFQRLVREIAQDFKTDLRFQSAAIGALQEASEAYLVGLFEDTNLCAIHAKRVTI  223
            LLIRKLPFQRLVREIAQDFKTDLRFQS+A+ ALQEASEAYLVGLFEDTNLCAIHAKRVTI
Sbjct  61   LLIRKLPFQRLVREIAQDFKTDLRFQSSAVMALQEASEAYLVGLFEDTNLCAIHAKRVTI  120

Query  224  MPKDIQLARRIRGER  238
            MPKDIQLARRIRGER
Sbjct  121  MPKDIQLARRIRGER  135
```

>uncharacterized protein LOC134934408 [Pseudophryne corroboree]
Sequence ID: XP_063785922.1 Length: 453
Range 1: 1 to 246

Score:444 bits(1143), Expect:1e-153,
Method:Compositional matrix adjust.,
Identities:230/246(93%), Positives:234/246(95%), Gaps:8/246(3%)

```
Query  1    MSGRGKGGKGLGKGGAKRHRKVLRDNIQGITKPAIRRLARRGGVKRISGLIYEETRGVLK  60
            MSGRGKGGKGLGKGGAKRHRKVLRDNIQGITKPAIRRLARRGGVKRISGLIYEETRGVLK
Sbjct  1    MSGRGKGGKGLGKGGAKRHRKVLRDNIQGITKPAIRRLARRGGVKRISGLIYEETRGVLK  60

Query  61   VFLENVIRDAVTYTEHAKRKTVTAMDVVYALKRQGRTL----YGF----GGMARTKQTAR  112
```

```
          VFLENVIRDAVTYTEHAKRKTVTAMDVVYALKRQGRTL    + F      MARTKQTAR
Sbjct  61   VFLENVIRDAVTYTEHAKRKTVTAMDVVYALKRQGRTLTAALHHFILQTLEMARTKQTAR  120

Query  113  KSTGGKAPRKQLATKAARKSAPSTGGVKKPHRYRPGTVALREIRRYQKSTELLIRKLPFQ  172
            KSTGGKAPRKQLATKAARKSAP+TGGVKKPHRYRPGTVALREIRRYQKSTELLIRKLPFQ
Sbjct  121  KSTGGKAPRKQLATKAARKSAPATGGVKKPHRYRPGTVALREIRRYQKSTELLIRKLPFQ  180

Query  173  RLVREIAQDFKTDLRFQSAAIGALQEASEAYLVGLFEDTNLCAIHAKRVTIMPKDIQLAR  232
            RLVREIAQDFKTDLRFQS+A+ ALQEASEAYLVGLFEDTNLCAIHAKRVTIMPKDIQLAR
Sbjct  181  RLVREIAQDFKTDLRFQSSAVMALQEASEAYLVGLFEDTNLCAIHAKRVTIMPKDIQLAR  240

Query  233  RIRGER  238
            RIRGER
Sbjct  241  RIRGER  246
```

Range 2: 351 to 453

Score:203 bits(516), Expect:5e-59,
Method:Compositional matrix adjust.,
Identities:101/103(98%), Positives:102/103(99%), Gaps:0/103(0%)

```
Query  1    MSGRGKGGKGLGKGGAKRHRKVLRDNIQGITKPAIRRLARRGGVKRISGLIYEETRGVLK  60
            MSGRGKGGKGLGKGGAKRHRKVL+DNIQGITKPAIRRLAR GGVKRISGLIYEETRGVLK
Sbjct  351  MSGRGKGGKGLGKGGAKRHRKVLQDNIQGITKPAIRRLAREGGVKRISGLIYEETRGVLK  410

Query  61   VFLENVIRDAVTYTEHAKRKTVTAMDVVYALKRQGRTLYGFGG  103
            VFLENVIRDAVTYTEHAKRKTVTAMDVVYALKRQGRTLYGFGG
Sbjct  411  VFLENVIRDAVTYTEHAKRKTVTAMDVVYALKRQGRTLYGFGG  453
```

>uncharacterized protein LOC121195787 [Toxotes jaculatrix]
Sequence ID: XP_040915384.1 Length: 589
Range 1: 1 to 245

Score:449 bits(1155), Expect:2e-153,
Method:Compositional matrix adjust.,
Identities:229/245(93%), Positives:232/245(94%), Gaps:7/245(2%)

```
Query  1    MSGRGKGGKGLGKGGAKRHRKVLRDNIQGITKPAIRRLARRGGVKRISGLIYEETRGVLK  60
            MSGRGKGGKGLGKGGAKRHRKVLRDNIQGITKPAIRRLARRGGVKRISGLIYEETRGVLK
Sbjct  1    MSGRGKGGKGLGKGGAKRHRKVLRDNIQGITKPAIRRLARRGGVKRISGLIYEETRGVLK  60

Query  61   VFLENVIRDAVTYTEHAKRKTVTAMDVVYALKRQGRTLYGF-------GGMARTKQTARK  113
            VFLENVIRDAVTYTEHAKRKTVTAMDVVYALKRQGRTL            MARTKQTARK
Sbjct  61   VFLENVIRDAVTYTEHAKRKTVTAMDVVYALKRQGRTLNTALHLQPEEAKMARTKQTARK  120

Query  114  STGGKAPRKQLATKAARKSAPSTGGVKKPHRYRPGTVALREIRRYQKSTELLIRKLPFQR  173
            STGGKAPRKQLATKAARKSAP+TGGVKKPHRYRPGTVALREIRRYQKSTELLIRKLPFQR
Sbjct  121  STGGKAPRKQLATKAARKSAPATGGVKKPHRYRPGTVALREIRRYQKSTELLIRKLPFQR  180
```

```
Query  174  LVREIAQDFKTDLRFQSAAIGALQEASEAYLVGLFEDTNLCAIHAKRVTIMPKDIQLARR  233
            LVREIAQDFKTDLRFQS+A+ ALQEASEAYLVGLFEDTNLCAIHAKRVTIMPKDIQLARR
Sbjct  181  LVREIAQDFKTDLRFQSSAVMALQEASEAYLVGLFEDTNLCAIHAKRVTIMPKDIQLARR  240

Query  234  IRGER  238
            IRGER
Sbjct  241  IRGER  245
```

Range 2: 454 to 589

Score:269 bits(687), Expect:5e-83,
Method:Compositional matrix adjust.,
Identities:132/136(97%), Positives:135/136(99%), Gaps:0/136(0%)

```
Query  104  MARTKQTARKSTGGKAPRKQLATKAARKSAPSTGGVKKPHRYRPGTVALREIRRYQKSTE  163
            MARTKQTARKSTGGKAPRKQLATKAARKSAP+TGGVKKPHRYRPGTVALREIRRYQKSTE
Sbjct  454  MARTKQTARKSTGGKAPRKQLATKAARKSAPATGGVKKPHRYRPGTVALREIRRYQKSTE  513

Query  164  LLIRKLPFQRLVREIAQDFKTDLRFQSAAIGALQEASEAYLVGLFEDTNLCAIHAKRVTI  223
            LLIRKLPFQRLVREIAQDFKTDLRFQS+A+ ALQEASEAYLVGLFEDTNLCAIHAKRVTI
Sbjct  514  LLIRKLPFQRLVREIAQDFKTDLRFQSSAVMALQEASEAYLVGLFEDTNLCAIHAKRVTI  573

Query  224  MPKDIQLARRIRGERA  239
            MPKDIQLARRIRGERA
Sbjct  574  MPKDIQLARRIRGERA  589
```

Range 3: 246 to 318

Score:149 bits(375), Expect:2e-37,
Method:Compositional matrix adjust.,
Identities:73/73(100%), Positives:73/73(100%), Gaps:0/73(0%)

```
Query  29   GITKPAIRRLARRGGVKRISGLIYEETRGVLKVFLENVIRDAVTYTEHAKRKTVTAMDVV  88
            GITKPAIRRLARRGGVKRISGLIYEETRGVLKVFLENVIRDAVTYTEHAKRKTVTAMDVV
Sbjct  246  GITKPAIRRLARRGGVKRISGLIYEETRGVLKVFLENVIRDAVTYTEHAKRKTVTAMDVV  305

Query  89   YALKRQGRTLYGF  101
            YALKRQGRTLYGF
Sbjct  306  YALKRQGRTLYGF  318
```

>uncharacterized protein LOC131703089 [Acipenser ruthenus]
Sequence ID: XP_058861937.1 Length: 422
Range 1: 1 to 248

Score:442 bits(1138), Expect:3e-153,
Method:Compositional matrix adjust.,
Identities:229/248(92%), Positives:232/248(93%), Gaps:10/248(4%)

```
Query  1    MSGRGKGGKGLGKGGAKRHRKVLRDNIQGITKPAIRRLARRGGVKRISGLIYEETRGVLK  60
            MSGRGKGGKGLGKGGAKRHRKVLRDNIQGITKPAIRRLARRGGVKRISGLIYEETRGVLK
Sbjct  1    MSGRGKGGKGLGKGGAKRHRKVLRDNIQGITKPAIRRLARRGGVKRISGLIYEETRGVLK  60

Query  61   VFLENVIRDAVTYTEHAKRKTVTAMDVVYALKRQGRTL----------YGFGGMARTKQT  110
            VFLENVIRDAVTYTEHAKRKTVTAMDVVYALKRQGRTL               MARTKQT
Sbjct  61   VFLENVIRDAVTYTEHAKRKTVTAMDVVYALKRQGRTLPSGRHHRPGRSASLKMARTKQT  120

Query  111  ARKSTGGKAPRKQLATKAARKSAPSTGGVKKPHRYRPGTVALREIRRYQKSTELLIRKLP  170
            ARKSTGGKAPRKQLATKAARKSAP+TGGVKKPHRYRPGTVALREIRRYQKSTELLIRKLP
Sbjct  121  ARKSTGGKAPRKQLATKAARKSAPATGGVKKPHRYRPGTVALREIRRYQKSTELLIRKLP  180

Query  171  FQRLVREIAQDFKTDLRFQSAAIGALQEASEAYLVGLFEDTNLCAIHAKRVTIMPKDIQL  230
            FQRLVREIAQDFKTDLRFQS+A+ ALQEASEAYLVGLFEDTNLCAIHAKRVTIMPKDIQL
Sbjct  181  FQRLVREIAQDFKTDLRFQSSAVMALQEASEAYLVGLFEDTNLCAIHAKRVTIMPKDIQL  240

Query  231  ARRIRGER  238
            ARRIRGER
Sbjct  241  ARRIRGER  248
```

Range 2: 320 to 422

Score:206 bits(524), Expect:2e-60,
Method:Compositional matrix adjust.,
Identities:103/103(100%), Positives:103/103(100%), Gaps:0/103(0%)

```
Query  1    MSGRGKGGKGLGKGGAKRHRKVLRDNIQGITKPAIRRLARRGGVKRISGLIYEETRGVLK  60
            MSGRGKGGKGLGKGGAKRHRKVLRDNIQGITKPAIRRLARRGGVKRISGLIYEETRGVLK
Sbjct  320  MSGRGKGGKGLGKGGAKRHRKVLRDNIQGITKPAIRRLARRGGVKRISGLIYEETRGVLK  379

Query  61   VFLENVIRDAVTYTEHAKRKTVTAMDVVYALKRQGRTLYGFGG  103
            VFLENVIRDAVTYTEHAKRKTVTAMDVVYALKRQGRTLYGFGG
Sbjct  380  VFLENVIRDAVTYTEHAKRKTVTAMDVVYALKRQGRTLYGFGG  422
```

>uncharacterized protein LOC125102583 [Lutra lutra]
Sequence ID: XP_047589869.1 Length: 542
Range 1: 291 to 542

Score:445 bits(1144), Expect:2e-152,
Method:Compositional matrix adjust.,
Identities:227/252(90%), Positives:231/252(91%), Gaps:18/252(7%)

```
Query  6    KGGKGLGKGGAKRHRKVLRDNIQGITKPAIRRLARRGGVKRISGLIYEETRGVLKVFLEN  65
            +GGKGLGKGGAKRHRKVLRDNIQGITKPAIRRLARRGGVKRISGLIYEETRGVLKVFLEN
```

```
Sbjct  291  QGGKGLGKGGAKRHRKVLRDNIQGITKPAIRRLARRGGVKRISGLIYEETRGVLKVFLEN  350

Query  66   VIRDAVTYTEHAKRKTVTAMDVVYALKRQGRTLYGFGG-------------------MART  107
            VIRDAVTYTEHAKRKTVTAMDVVYALKRQGRTLYGFG                   MART
Sbjct  351  VIRDAVTYTEHAKRKTVTAMDVVYALKRQGRTLYGFGVPGPRLCPLAALSCYLFFVMART  410

Query  108  KQTARKSTGGKAPRKQLATKAARKSAPSTGGVKKPHRYRPGTVALREIRRYQKSTELLIR  167
            KQTARKSTGGKAPRKQLATKAARKSAP+TGGVKKPHRYRPGTVALREIRRYQKSTELLIR
Sbjct  411  KQTARKSTGGKAPRKQLATKAARKSAPATGGVKKPHRYRPGTVALREIRRYQKSTELLIR  470

Query  168  KLPFQRLVREIAQDFKTDLRFQSAAIGALQEASEAYLVGLFEDTNLCAIHAKRVTIMPKD  227
            KLPFQRLVREIAQDFKTDLRFQS+A+ ALQEA EAYLVGLFEDTNLCAIHAKRVTIMPKD
Sbjct  471  KLPFQRLVREIAQDFKTDLRFQSSAVMALQEACEAYLVGLFEDTNLCAIHAKRVTIMPKD  530

Query  228  IQLARRIRGERA  239
            IQLARRIRGERA
Sbjct  531  IQLARRIRGERA  542
```

Range 2: 1 to 136

Score:265 bits(678), Expect:4e-82,
Method:Compositional matrix adjust.,
Identities:131/136(96%), Positives:134/136(98%), Gaps:0/136(0%)

```
Query  104  MARTKQTARKSTGGKAPRKQLATKAARKSAPSTGGVKKPHRYRPGTVALREIRRYQKSTE  163
            MARTKQTARKSTGGKAPRKQLATKAARKSAP+TGGVKKPHRYRPGTVALREIRRYQKSTE
Sbjct  1    MARTKQTARKSTGGKAPRKQLATKAARKSAPATGGVKKPHRYRPGTVALREIRRYQKSTE  60

Query  164  LLIRKLPFQRLVREIAQDFKTDLRFQSAAIGALQEASEAYLVGLFEDTNLCAIHAKRVTI  223
            LLIRKLPFQRLVREIAQDFKTDLRFQS+A+ ALQEA EAYLVGLFEDTNLCAIHAKRVTI
Sbjct  61   LLIRKLPFQRLVREIAQDFKTDLRFQSSAVMALQEACEAYLVGLFEDTNLCAIHAKRVTI  120

Query  224  MPKDIQLARRIRGERA  239
            MPKDIQLARRIRGERA
Sbjct  121  MPKDIQLARRIRGERA  136
```

>uncharacterized protein LOC134810327 [Pan troglodytes]
Sequence ID: XP_063669332.1 Length: 575
Range 1: 1 to 244

Score:445 bits(1144), Expect:5e-152,
Method:Compositional matrix adjust.,
Identities:229/244(94%), Positives:232/244(95%), Gaps:5/244(2%)

```
Query  1    MSGRGKGGKGLGKGGAKRHRKVLRDNIQGITKPAIRRLARRGGVKRISGLIYEETRGVLK  60
            MSGRGKGGKGLGKGGAKRHRKVLRDNIQGITKPAIRRLARRGGVKRISGLIYEETRGVLK
Sbjct  1    MSGRGKGGKGLGKGGAKRHRKVLRDNIQGITKPAIRRLARRGGVKRISGLIYEETRGVLK  60
```

```
Query  61   VFLENVIRDAVTYTEHAKRKTVTAMDVVYALKRQGR---TLYGFGG--MARTKQTARKST  115
            VFLENVIRDAVTYTEHAKRKTVTAMDVVYALKRQGR   TL       MARTKQTARKST
Sbjct  61   VFLENVIRDAVTYTEHAKRKTVTAMDVVYALKRQGRRFTTLLCVLSLQMARTKQTARKST  120

Query  116  GGKAPRKQLATKAARKSAPSTGGVKKPHRYRPGTVALREIRRYQKSTELLIRKLPFQRLV  175
            GGKAPRKQLATKAARKSAP+TGGVKKPHRYRPGTVALREIRRYQKSTELLIRKLPFQRLV
Sbjct  121  GGKAPRKQLATKAARKSAPATGGVKKPHRYRPGTVALREIRRYQKSTELLIRKLPFQRLV  180

Query  176  REIAQDFKTDLRFQSAAIGALQEASEAYLVGLFEDTNLCAIHAKRVTIMPKDIQLARRIR  235
            REIAQDFKTDLRFQS+A+ ALQEA EAYLVGLFEDTNLCAIHAKRVTIMPKDIQLARRIR
Sbjct  181  REIAQDFKTDLRFQSSAVMALQEACEAYLVGLFEDTNLCAIHAKRVTIMPKDIQLARRIR  240

Query  236  GERA  239
            GERA
Sbjct  241  GERA  244
```

Range 2: 473 to 575

Score:208 bits(530), Expect:6e-60,
Method:Compositional matrix adjust.,
Identities:103/103(100%), Positives:103/103(100%), Gaps:0/103(0%)

```
Query  1    MSGRGKGGKGLGKGGAKRHRKVLRDNIQGITKPAIRRLARRGGVKRISGLIYEETRGVLK  60
            MSGRGKGGKGLGKGGAKRHRKVLRDNIQGITKPAIRRLARRGGVKRISGLIYEETRGVLK
Sbjct  473  MSGRGKGGKGLGKGGAKRHRKVLRDNIQGITKPAIRRLARRGGVKRISGLIYEETRGVLK  532

Query  61   VFLENVIRDAVTYTEHAKRKTVTAMDVVYALKRQGRTLYGFGG  103
            VFLENVIRDAVTYTEHAKRKTVTAMDVVYALKRQGRTLYGFGG
Sbjct  533  VFLENVIRDAVTYTEHAKRKTVTAMDVVYALKRQGRTLYGFGG  575
```

>uncharacterized protein LOC124227098 [Equus quagga]
Sequence ID: XP_046496953.1 Length: 677
Range 1: 1 to 246

Score:448 bits(1153), Expect:6e-152,
Method:Compositional matrix adjust.,
Identities:230/247(93%), Positives:234/247(94%), Gaps:9/247(3%)

```
Query  1    MSGRGKGGKGLGKGGAKRHRKVLRDNIQGITKPAIRRLARRGGVKRISGLIYEETRGVLK  60
            MSGRGKGGKGLGKGGAKRHRKVLRDNIQGITKPAIRRLARRGGVKRISGLIYEETRGVLK
Sbjct  1    MSGRGKGGKGLGKGGAKRHRKVLRDNIQGITKPAIRRLARRGGVKRISGLIYEETRGVLK  60

Query  61   VFLENVIRDAVTYTEHAKRKTVTAMDVVYALKRQGRTLYGFG--------GMARTKQTAR  112
            VFLENVIRDAVTYTEHAKRKTVTAMDVVYALKRQGRTL+ F          MARTKQTAR
Sbjct  61   VFLENVIRDAVTYTEHAKRKTVTAMDVVYALKRQGRTLH-FPLILVLFRQAMARTKQTAR  119

Query  113  KSTGGKAPRKQLATKAARKSAPSTGGVKKPHRYRPGTVALREIRRYQKSTELLIRKLPFQ  172
            KSTGGKAPRKQLATKAARKSAP+TGGVKKPHRYRPGTVALREIRRYQKSTELLIRKLPFQ
```

```
Sbjct  120  KSTGGKAPRKQLATKAARKSAPATGGVKKPHRYRPGTVALREIRRYQKSTELLIRKLPFQ  179

Query  173  RLVREIAQDFKTDLRFQSAAIGALQEASEAYLVGLFEDTNLCAIHAKRVTIMPKDIQLAR  232
            RLVREIAQDFKTDLRFQS+A+ ALQEA EAYLVGLFEDTNLCAIHAKRVTIMPKDIQLAR
Sbjct  180  RLVREIAQDFKTDLRFQSSAVMALQEACEAYLVGLFEDTNLCAIHAKRVTIMPKDIQLAR  239

Query  233  RIRGERA  239
            RIRGERA
Sbjct  240  RIRGERA  246
```

Range 2: 575 to 677

Score:208 bits(530), Expect:4e-59,
Method:Compositional matrix adjust.,
Identities:103/103(100%), Positives:103/103(100%), Gaps:0/103(0%)

```
Query  1    MSGRGKGGKGLGKGGAKRHRKVLRDNIQGITKPAIRRLARRGGVKRISGLIYEETRGVLK  60
            MSGRGKGGKGLGKGGAKRHRKVLRDNIQGITKPAIRRLARRGGVKRISGLIYEETRGVLK
Sbjct  575  MSGRGKGGKGLGKGGAKRHRKVLRDNIQGITKPAIRRLARRGGVKRISGLIYEETRGVLK  634

Query  61   VFLENVIRDAVTYTEHAKRKTVTAMDVVYALKRQGRTLYGFGG  103
            VFLENVIRDAVTYTEHAKRKTVTAMDVVYALKRQGRTLYGFGG
Sbjct  635  VFLENVIRDAVTYTEHAKRKTVTAMDVVYALKRQGRTLYGFGG  677
```

>uncharacterized protein LOC128594725 [Nycticebus coucang]
Sequence ID: XP_053459600.1 Length: 454
Range 1: 1 to 230

Score:437 bits(1125), Expect:6e-151,
Method:Compositional matrix adjust.,
Identities:221/232(95%), Positives:225/232(96%), Gaps:2/232(0%)

```
Query  1    MSGRGKGGKGLGKGGAKRHRKVLRDNIQGITKPAIRRLARRGGVKRISGLIYEETRGVLK  60
            MSGRGKGGKGLGKGGAKRHRKVLRDNIQGITKPAIRRLARRGGVKRISGLIYEETRGVLK
Sbjct  1    MSGRGKGGKGLGKGGAKRHRKVLRDNIQGITKPAIRRLARRGGVKRISGLIYEETRGVLK  60

Query  61   VFLENVIRDAVTYTEHAKRKTVTAMDVVYALKRQGRTLYGFGGMARTKQTARKSTGGKAP  120
            VFLENVIRDAVTYTEHAKRKTVTAMDVVYALKRQGRTLYGFG +  T  TARKSTGGKAP
Sbjct  61   VFLENVIRDAVTYTEHAKRKTVTAMDVVYALKRQGRTLYGFGDLVDT--TARKSTGGKAP  118

Query  121  RKQLATKAARKSAPSTGGVKKPHRYRPGTVALREIRRYQKSTELLIRKLPFQRLVREIAQ  180
            RKQLATKAARKSAP+TGGVKKPHRYRPGTVALREIRRYQKSTELLIRKLPFQRLVREIAQ
Sbjct  119  RKQLATKAARKSAPATGGVKKPHRYRPGTVALREIRRYQKSTELLIRKLPFQRLVREIAQ  178

Query  181  DFKTDLRFQSAAIGALQEASEAYLVGLFEDTNLCAIHAKRVTIMPKDIQLAR  232
            DFKTDLRFQS+A+ ALQEA EAYLVGLFEDTNLCAIHAKRVTIMPKDIQLAR
Sbjct  179  DFKTDLRFQSSAVMALQEACEAYLVGLFEDTNLCAIHAKRVTIMPKDIQLAR  230
```

Range 2: 199 to 344

Score:221 bits(564), Expect:3e-66,
Method:Compositional matrix adjust.,
Identities:115/155(74%), Positives:123/155(79%), Gaps:9/155(5%)

```
Query  60   KVFLENVIRDAVTYTEHAKRKTVTAMDVVYALKRQGRTLYGFGGMARTKQTARKSTGGKA  119
            + +L  +  D      HAKR T+   D+          L     MARTKQTARKSTGGKA
Sbjct  199  EAYLVGLFEDTNLCAIHAKRVTIMPKDI---------QLARLFIMARTKQTARKSTGGKA  249

Query  120  PRKQLATKAARKSAPSTGGVKKPHRYRPGTVALREIRRYQKSTELLIRKLPFQRLVREIA  179
            PRKQLATKAARKSAP+TGGVKKPHRYRPGTVALREIRRYQKSTELLIRKLPFQRLVREIA
Sbjct  250  PRKQLATKAARKSAPATGGVKKPHRYRPGTVALREIRRYQKSTELLIRKLPFQRLVREIA  309

Query  180  QDFKTDLRFQSAAIGALQEASEAYLVGLFEDTNLC  214
            QDFKTDLRFQS+A+ ALQEA EAYLVGLFEDTNLC
Sbjct  310  QDFKTDLRFQSSAVMALQEACEAYLVGLFEDTNLC  344
```

Range 3: 352 to 454

Score:206 bits(524), Expect:4e-60,
Method:Compositional matrix adjust.,
Identities:103/103(100%), Positives:103/103(100%), Gaps:0/103(0%)

```
Query  1    MSGRGKGGKGLGKGGAKRHRKVLRDNIQGITKPAIRRLARRGGVKRISGLIYEETRGVLK  60
            MSGRGKGGKGLGKGGAKRHRKVLRDNIQGITKPAIRRLARRGGVKRISGLIYEETRGVLK
Sbjct  352  MSGRGKGGKGLGKGGAKRHRKVLRDNIQGITKPAIRRLARRGGVKRISGLIYEETRGVLK  411

Query  61   VFLENVIRDAVTYTEHAKRKTVTAMDVVYALKRQGRTLYGFGG  103
            VFLENVIRDAVTYTEHAKRKTVTAMDVVYALKRQGRTLYGFGG
Sbjct  412  VFLENVIRDAVTYTEHAKRKTVTAMDVVYALKRQGRTLYGFGG  454
```

>LOW QUALITY PROTEIN: uncharacterized protein LOC135232753 [Loxodonta africana]
Sequence ID: XP_064149686.1 Length: 689
Range 1: 1 to 247

Score:446 bits(1147), Expect:7e-151,
Method:Compositional matrix adjust.,
Identities:229/247(93%), Positives:233/247(94%), Gaps:8/247(3%)

```
Query  1    MSGRGKGGKGLGKGGAKRHRKVLRDNIQGITKPAIRRLARRGGVKRISGLIYEETRGVLK  60
            MSGRGKGGKGLGKGGAKRHRKVLRDNIQGITKPAIRRLARRGGVKRISGLIYEETRGVLK
Sbjct  1    MSGRGKGGKGLGKGGAKRHRKVLRDNIQGITKPAIRRLARRGGVKRISGLIYEETRGVLK  60

Query  61   VFLENVIRDAVTYTEHAKRKTVTAMDVVYALKRQGRTL--------YGFGGMARTKQTAR  112
```

```
           VFLENVIRDAVTYTEHAKRKTVTAMDVVYALKRQGRTL        +    MARTKQTAR
Sbjct  61  VFLENVIRDAVTYTEHAKRKTVTAMDVVYALKRQGRTLSXSLQCSSHDSLLMARTKQTAR  120

Query  113 KSTGGKAPRKQLATKAARKSAPSTGGVKKPHRYRPGTVALREIRRYQKSTELLIRKLPFQ  172
           KSTGGKAPRKQLATKAARKSAP+TGGVKKPHRYRPGTVALREIRRYQKSTELLIRKLPFQ
Sbjct  121 KSTGGKAPRKQLATKAARKSAPATGGVKKPHRYRPGTVALREIRRYQKSTELLIRKLPFQ  180

Query  173 RLVREIAQDFKTDLRFQSAAIGALQEASEAYLVGLFEDTNLCAIHAKRVTIMPKDIQLAR  232
           RLVREIAQDFKTDLRFQS+A+ ALQEA EAYLVGLFEDTNLCAIHAKRVTIMPKDIQLAR
Sbjct  181 RLVREIAQDFKTDLRFQSSAVMALQEACEAYLVGLFEDTNLCAIHAKRVTIMPKDIQLAR  240

Query  233 RIRGERA  239
           RIRGERA
Sbjct  241 RIRGERA  247
```

Range 2: 587 to 689

Score:207 bits(526), Expect:2e-58,
Method:Compositional matrix adjust.,
Identities:103/103(100%), Positives:103/103(100%), Gaps:0/103(0%)

```
Query  1   MSGRGKGGKGLGKGGAKRHRKVLRDNIQGITKPAIRRLARRGGVKRISGLIYEETRGVLK  60
           MSGRGKGGKGLGKGGAKRHRKVLRDNIQGITKPAIRRLARRGGVKRISGLIYEETRGVLK
Sbjct  587 MSGRGKGGKGLGKGGAKRHRKVLRDNIQGITKPAIRRLARRGGVKRISGLIYEETRGVLK  646

Query  61  VFLENVIRDAVTYTEHAKRKTVTAMDVVYALKRQGRTLYGFGG  103
           VFLENVIRDAVTYTEHAKRKTVTAMDVVYALKRQGRTLYGFGG
Sbjct  647 VFLENVIRDAVTYTEHAKRKTVTAMDVVYALKRQGRTLYGFGG  689
```

>uncharacterized protein LOC102471222 [Tupaia chinensis]
Sequence ID: XP_014448824.1 Length: 450
Range 1: 203 to 450

Score:436 bits(1122), Expect:2e-150,
Method:Compositional matrix adjust.,
Identities:224/248(90%), Positives:227/248(91%), Gaps:18/248(7%)

```
Query  10  GLGKGGAKRHRKVLRDNIQGITKPAIRRLARRGGVKRISGLIYEETRGVLKVFLENVIRD  69
           GLGKGGAKRHRKVLRDNIQGITKPAIRRLARRGGVKRISGLIYEETRGVLKVFLENVIRD
Sbjct  203 GLGKGGAKRHRKVLRDNIQGITKPAIRRLARRGGVKRISGLIYEETRGVLKVFLENVIRD  262

Query  70  AVTYTEHAKRKTVTAMDVVYALKRQGRTLYGFGG------------------MARTKQTA  111
           AVTYTEHAKRKTVTAMDVVYALKRQGRTLYGF G                  MARTKQTA
Sbjct  263 AVTYTEHAKRKTVTAMDVVYALKRQGRTLYGFVGRGVWGLLSLQLGSYFDTVMARTKQTA  322

Query  112 RKSTGGKAPRKQLATKAARKSAPSTGGVKKPHRYRPGTVALREIRRYQKSTELLIRKLPF  171
           RKSTGGKAPRKQLATKAARKSAP+TGGVKKPHRYRPGTVALREIRRYQKSTELLIRKLPF
Sbjct  323 RKSTGGKAPRKQLATKAARKSAPATGGVKKPHRYRPGTVALREIRRYQKSTELLIRKLPF  382
```

```
Query  172  QRLVREIAQDFKTDLRFQSAAIGALQEASEAYLVGLFEDTNLCAIHAKRVTIMPKDIQLA  231
            QRLVREIAQDFKTDLRFQS+A+ ALQEA EAYLVGLFEDTNLCAIHAKRVTIMPKDIQLA
Sbjct  383  QRLVREIAQDFKTDLRFQSSAVMALQEACEAYLVGLFEDTNLCAIHAKRVTIMPKDIQLA  442

Query  232  RRIRGERA  239
            RRIRGERA
Sbjct  443  RRIRGERA  450
```

Range 2: 1 to 136

Score:265 bits(678), Expect:3e-83,
Method:Compositional matrix adjust.,
Identities:131/136(96%), Positives:134/136(98%), Gaps:0/136(0%)

```
Query  104  MARTKQTARKSTGGKAPRKQLATKAARKSAPSTGGVKKPHRYRPGTVALREIRRYQKSTE  163
            MARTKQTARKSTGGKAPRKQLATKAARKSAP+TGGVKKPHRYRPGTVALREIRRYQKSTE
Sbjct  1    MARTKQTARKSTGGKAPRKQLATKAARKSAPATGGVKKPHRYRPGTVALREIRRYQKSTE  60

Query  164  LLIRKLPFQRLVREIAQDFKTDLRFQSAAIGALQEASEAYLVGLFEDTNLCAIHAKRVTI  223
            LLIRKLPFQRLVREIAQDFKTDLRFQS+A+ ALQEA EAYLVGLFEDTNLCAIHAKRVTI
Sbjct  61   LLIRKLPFQRLVREIAQDFKTDLRFQSSAVMALQEACEAYLVGLFEDTNLCAIHAKRVTI  120

Query  224  MPKDIQLARRIRGERA  239
            MPKDIQLARRIRGERA
Sbjct  121  MPKDIQLARRIRGERA  136
```

>uncharacterized protein LOC100664283 [Loxodonta africana]
Sequence ID: XP_064149664.1 Length: 790
Range 1: 211 to 456

Score:446 bits(1147), Expect:1e-149,
Method:Compositional matrix adjust.,
Identities:230/246(93%), Positives:233/246(94%), Gaps:7/246(2%)

```
Query  1    MSGRGKGGKGLGKGGAKRHRKVLRDNIQGITKPAIRRLARRGGVKRISGLIYEETRGVLK  60
            MSGRGKGGKGLGKGGAKRHRKVLRDNIQGITKPAIRRLARRGGVKRISGLIYEETRGVLK
Sbjct  211  MSGRGKGGKGLGKGGAKRHRKVLRDNIQGITKPAIRRLARRGGVKRISGLIYEETRGVLK  270

Query  61   VFLENVIRDAVTYTEHAKRKTVTAMDVVYALKRQGRTL-------YGFGGMARTKQTARK  113
            VFLENVIRDAVTYTEHAKRKTVTAMDVVYALKRQGRTL        G   MARTKQTARK
Sbjct  271  VFLENVIRDAVTYTEHAKRKTVTAMDVVYALKRQGRTLPSDLHHRAGRFSMARTKQTARK  330

Query  114  STGGKAPRKQLATKAARKSAPSTGGVKKPHRYRPGTVALREIRRYQKSTELLIRKLPFQR  173
            STGGKAPRKQLATKAARKSAP+TGGVKKPHRYRPGTVALREIRRYQKSTELLIRKLPFQR
Sbjct  331  STGGKAPRKQLATKAARKSAPATGGVKKPHRYRPGTVALREIRRYQKSTELLIRKLPFQR  390

Query  174  LVREIAQDFKTDLRFQSAAIGALQEASEAYLVGLFEDTNLCAIHAKRVTIMPKDIQLARR  233
```

```
           LVREIAQDFKTDLRFQS+A+ ALQEA EAYLVGLFEDTNLCAIHAKRVTIMPKDIQLARR
Sbjct  391  LVREIAQDFKTDLRFQSSAVMALQEACEAYLVGLFEDTNLCAIHAKRVTIMPKDIQLARR  450

Query  234  IRGERA  239
           IRGERA
Sbjct  451  IRGERA  456
```

Range 2: 688 to 790

Score:206 bits(525), Expect:7e-58,
Method:Compositional matrix adjust.,
Identities:103/103(100%), Positives:103/103(100%), Gaps:0/103(0%)

```
Query  1    MSGRGKGGKGLGKGGAKRHRKVLRDNIQGITKPAIRRLARRGGVKRISGLIYEETRGVLK  60
           MSGRGKGGKGLGKGGAKRHRKVLRDNIQGITKPAIRRLARRGGVKRISGLIYEETRGVLK
Sbjct  688  MSGRGKGGKGLGKGGAKRHRKVLRDNIQGITKPAIRRLARRGGVKRISGLIYEETRGVLK  747

Query  61   VFLENVIRDAVTYTEHAKRKTVTAMDVVYALKRQGRTLYGFGG  103
           VFLENVIRDAVTYTEHAKRKTVTAMDVVYALKRQGRTLYGFGG
Sbjct  748  VFLENVIRDAVTYTEHAKRKTVTAMDVVYALKRQGRTLYGFGG  790
```

>unnamed protein product [Lampetra fluviatilis]
Sequence ID: CAL5913199.1 Length: 1283
Range 1: 1 to 234

Score:457 bits(1177), Expect:4e-149,
Method:Compositional matrix adjust.,
Identities:228/234(97%), Positives:231/234(98%), Gaps:0/234(0%)

```
Query  1    MSGRGKGGKGLGKGGAKRHRKVLRDNIQGITKPAIRRLARRGGVKRISGLIYEETRGVLK  60
           MSGRGKGGKGLGKGGAKRHRKVLRDNIQGITKPAIRRLARRGGVKRISGLIYEETRGVLK
Sbjct  1    MSGRGKGGKGLGKGGAKRHRKVLRDNIQGITKPAIRRLARRGGVKRISGLIYEETRGVLK  60

Query  61   VFLENVIRDAVTYTEHAKRKTVTAMDVVYALKRQGRTLYGFGGMARTKQTARKSTGGKAP  120
           VFLENVIRDAVTYTEHAKRKTVTAMDVVYALKRQGRTLYGFG MARTKQTARKSTGGKAP
Sbjct  61   VFLENVIRDAVTYTEHAKRKTVTAMDVVYALKRQGRTLYGFGAMARTKQTARKSTGGKAP  120

Query  121  RKQLATKAARKSAPSTGGVKKPHRYRPGTVALREIRRYQKSTELLIRKLPFQRLVREIAQ  180
           RKQLATKAARKSAP+TGGVKKPHRYRPGTVALREIRRYQKSTELLIRKLPFQRLVREIAQ
Sbjct  121  RKQLATKAARKSAPATGGVKKPHRYRPGTVALREIRRYQKSTELLIRKLPFQRLVREIAQ  180

Query  181  DFKTDLRFQSAAIGALQEASEAYLVGLFEDTNLCAIHAKRVTIMPKDIQLARRI  234
           DFKTDLRFQS+A+ ALQEASEAYLV LFEDTNLCAIHAKRVTIMPKDIQLARRI
Sbjct  181  DFKTDLRFQSSAVMALQEASEAYLVALFEDTNLCAIHAKRVTIMPKDIQLARRI  234
```

Range 2: 237 to 470

Score:457 bits(1177), Expect:4e-149,
Method:Compositional matrix adjust.,
Identities:228/234(97%), Positives:231/234(98%), Gaps:0/234(0%)

```
Query  1    MSGRGKGGKGLGKGGAKRHRKVLRDNIQGITKPAIRRLARRGGVKRISGLIYEETRGVLK  60
            MSGRGKGGKGLGKGGAKRHRKVLRDNIQGITKPAIRRLARRGGVKRISGLIYEETRGVLK
Sbjct  237  MSGRGKGGKGLGKGGAKRHRKVLRDNIQGITKPAIRRLARRGGVKRISGLIYEETRGVLK  296

Query  61   VFLENVIRDAVTYTEHAKRKTVTAMDVVYALKRQGRTLYGFGGMARTKQTARKSTGGKAP  120
            VFLENVIRDAVTYTEHAKRKTVTAMDVVYALKRQGRTLYGFG MARTKQTARKSTGGKAP
Sbjct  297  VFLENVIRDAVTYTEHAKRKTVTAMDVVYALKRQGRTLYGFGAMARTKQTARKSTGGKAP  356

Query  121  RKQLATKAARKSAPSTGGVKKPHRYRPGTVALREIRRYQKSTELLIRKLPFQRLVREIAQ  180
            RKQLATKAARKSAP+TGGVKKPHRYRPGTVALREIRRYQKSTELLIRKLPFQRLVREIAQ
Sbjct  357  RKQLATKAARKSAPATGGVKKPHRYRPGTVALREIRRYQKSTELLIRKLPFQRLVREIAQ  416

Query  181  DFKTDLRFQSAAIGALQEASEAYLVGLFEDTNLCAIHAKRVTIMPKDIQLARRI  234
            DFKTDLRFQS+A+ ALQEASEAYLV LFEDTNLCAIHAKRVTIMPKDIQLARRI
Sbjct  417  DFKTDLRFQSSAVMALQEASEAYLVALFEDTNLCAIHAKRVTIMPKDIQLARRI  470
```

Range 3: 473 to 706

Score:457 bits(1177), Expect:4e-149,
Method:Compositional matrix adjust.,
Identities:228/234(97%), Positives:231/234(98%), Gaps:0/234(0%)

```
Query  1    MSGRGKGGKGLGKGGAKRHRKVLRDNIQGITKPAIRRLARRGGVKRISGLIYEETRGVLK  60
            MSGRGKGGKGLGKGGAKRHRKVLRDNIQGITKPAIRRLARRGGVKRISGLIYEETRGVLK
Sbjct  473  MSGRGKGGKGLGKGGAKRHRKVLRDNIQGITKPAIRRLARRGGVKRISGLIYEETRGVLK  532

Query  61   VFLENVIRDAVTYTEHAKRKTVTAMDVVYALKRQGRTLYGFGGMARTKQTARKSTGGKAP  120
            VFLENVIRDAVTYTEHAKRKTVTAMDVVYALKRQGRTLYGFG MARTKQTARKSTGGKAP
Sbjct  533  VFLENVIRDAVTYTEHAKRKTVTAMDVVYALKRQGRTLYGFGAMARTKQTARKSTGGKAP  592

Query  121  RKQLATKAARKSAPSTGGVKKPHRYRPGTVALREIRRYQKSTELLIRKLPFQRLVREIAQ  180
            RKQLATKAARKSAP+TGGVKKPHRYRPGTVALREIRRYQKSTELLIRKLPFQRLVREIAQ
Sbjct  593  RKQLATKAARKSAPATGGVKKPHRYRPGTVALREIRRYQKSTELLIRKLPFQRLVREIAQ  652

Query  181  DFKTDLRFQSAAIGALQEASEAYLVGLFEDTNLCAIHAKRVTIMPKDIQLARRI  234
            DFKTDLRFQS+A+ ALQEASEAYLV LFEDTNLCAIHAKRVTIMPKDIQLARRI
Sbjct  653  DFKTDLRFQSSAVMALQEASEAYLVALFEDTNLCAIHAKRVTIMPKDIQLARRI  706
```

Range 4: 709 to 942

Score:457 bits(1177), Expect:4e-149,
Method:Compositional matrix adjust.,

```
Identities:228/234(97%), Positives:231/234(98%), Gaps:0/234(0%)

Query  1    MSGRGKGGKGLGKGGAKRHRKVLRDNIQGITKPAIRRLARRGGVKRISGLIYEETRGVLK  60
            MSGRGKGGKGLGKGGAKRHRKVLRDNIQGITKPAIRRLARRGGVKRISGLIYEETRGVLK
Sbjct  709  MSGRGKGGKGLGKGGAKRHRKVLRDNIQGITKPAIRRLARRGGVKRISGLIYEETRGVLK  768

Query  61   VFLENVIRDAVTYTEHAKRKTVTAMDVVYALKRQGRTLYGFGGMARTKQTARKSTGGKAP  120
            VFLENVIRDAVTYTEHAKRKTVTAMDVVYALKRQGRTLYGFG MARTKQTARKSTGGKAP
Sbjct  769  VFLENVIRDAVTYTEHAKRKTVTAMDVVYALKRQGRTLYGFGAMARTKQTARKSTGGKAP  828

Query  121  RKQLATKAARKSAPSTGGVKKPHRYRPGTVALREIRRYQKSTELLIRKLPFQRLVREIAQ  180
            RKQLATKAARKSAP+TGGVKKPHRYRPGTVALREIRRYQKSTELLIRKLPFQRLVREIAQ
Sbjct  829  RKQLATKAARKSAPATGGVKKPHRYRPGTVALREIRRYQKSTELLIRKLPFQRLVREIAQ  888

Query  181  DFKTDLRFQSAAIGALQEASEAYLVGLFEDTNLCAIHAKRVTIMPKDIQLARRI  234
            DFKTDLRFQS+A+ ALQEASEAYLV LFEDTNLCAIHAKRVTIMPKDIQLARRI
Sbjct  889  DFKTDLRFQSSAVMALQEASEAYLVALFEDTNLCAIHAKRVTIMPKDIQLARRI  942
```

Range 5: 945 to 1178

Score:457 bits(1177), Expect:4e-149,
Method:Compositional matrix adjust.,
Identities:228/234(97%), Positives:231/234(98%), Gaps:0/234(0%)

```
Query  1     MSGRGKGGKGLGKGGAKRHRKVLRDNIQGITKPAIRRLARRGGVKRISGLIYEETRGVLK  60
             MSGRGKGGKGLGKGGAKRHRKVLRDNIQGITKPAIRRLARRGGVKRISGLIYEETRGVLK
Sbjct  945   MSGRGKGGKGLGKGGAKRHRKVLRDNIQGITKPAIRRLARRGGVKRISGLIYEETRGVLK  1004

Query  61    VFLENVIRDAVTYTEHAKRKTVTAMDVVYALKRQGRTLYGFGGMARTKQTARKSTGGKAP  120
             VFLENVIRDAVTYTEHAKRKTVTAMDVVYALKRQGRTLYGFG MARTKQTARKSTGGKAP
Sbjct  1005  VFLENVIRDAVTYTEHAKRKTVTAMDVVYALKRQGRTLYGFGAMARTKQTARKSTGGKAP  1064

Query  121   RKQLATKAARKSAPSTGGVKKPHRYRPGTVALREIRRYQKSTELLIRKLPFQRLVREIAQ  180
             RKQLATKAARKSAP+TGGVKKPHRYRPGTVALREIRRYQKSTELLIRKLPFQRLVREIAQ
Sbjct  1065  RKQLATKAARKSAPATGGVKKPHRYRPGTVALREIRRYQKSTELLIRKLPFQRLVREIAQ  1124

Query  181   DFKTDLRFQSAAIGALQEASEAYLVGLFEDTNLCAIHAKRVTIMPKDIQLARRI  234
             DFKTDLRFQS+A+ ALQEASEAYLV LFEDTNLCAIHAKRVTIMPKDIQLARRI
Sbjct  1125  DFKTDLRFQSSAVMALQEASEAYLVALFEDTNLCAIHAKRVTIMPKDIQLARRI  1178
```

Range 6: 1181 to 1283

Score:207 bits(527), Expect:3e-57,
Method:Compositional matrix adjust.,
Identities:103/103(100%), Positives:103/103(100%), Gaps:0/103(0%)

```
Query  1     MSGRGKGGKGLGKGGAKRHRKVLRDNIQGITKPAIRRLARRGGVKRISGLIYEETRGVLK  60
             MSGRGKGGKGLGKGGAKRHRKVLRDNIQGITKPAIRRLARRGGVKRISGLIYEETRGVLK
```

```
Sbjct  1181  MSGRGKGGKGLGKGGAKRHRKVLRDNIQGITKPAIRRLARRGGVKRISGLIYEETRGVLK  1240

Query  61    VFLENVIRDAVTYTEHAKRKTVTAMDVVYALKRQGRTLYGFGG  103
             VFLENVIRDAVTYTEHAKRKTVTAMDVVYALKRQGRTLYGFGG
Sbjct  1241  VFLENVIRDAVTYTEHAKRKTVTAMDVVYALKRQGRTLYGFGG  1283
```

>LOW QUALITY PROTEIN: uncharacterized protein LOC132316938 [Gavia stellata]
Sequence ID: XP_059672939.1 Length: 332
Range 1: 1 to 223

Score:427 bits(1098), Expect:1e-148,
Method:Compositional matrix adjust.,
Identities:216/223(97%), Positives:219/223(98%), Gaps:3/223(1%)

```
Query  1    MSGRGKGGKGLGKGGAKRHRKVLRDNIQGITKPAIRRLARRGGVKRISGLIYEETRGVLK  60
            MSGRGKGGKGLGKGGAKRHRKVLRDNIQGITKPAIRRLARRGGVKRISGLIYEETRGVLK
Sbjct  1    MSGRGKGGKGLGKGGAKRHRKVLRDNIQGITKPAIRRLARRGGVKRISGLIYEETRGVLK  60

Query  61   VFLENVIRDAVTYTEHAKRKTVTAMDVVYALKRQGRTLYGFGG---MARTKQTARKSTGG  117
            VFLENVIRDAVTYTEHAKRKTVTAMDVVYALKRQGRTLYGFGG   MARTKQTARKSTGG
Sbjct  61   VFLENVIRDAVTYTEHAKRKTVTAMDVVYALKRQGRTLYGFGGXSSMARTKQTARKSTGG  120

Query  118  KAPRKQLATKAARKSAPSTGGVKKPHRYRPGTVALREIRRYQKSTELLIRKLPFQRLVRE  177
            KAPRKQLATKAARKSAP+TGGVKKPHRYRPGTVALREIRRYQKSTELLIRKLPFQRLVRE
Sbjct  121  KAPRKQLATKAARKSAPATGGVKKPHRYRPGTVALREIRRYQKSTELLIRKLPFQRLVRE  180

Query  178  IAQDFKTDLRFQSAAIGALQEASEAYLVGLFEDTNLCAIHAKR  220
            IAQDFKTDLRFQS+A+ ALQEASEAYLVGLFEDTNLCAIHAKR
Sbjct  181  IAQDFKTDLRFQSSAVMALQEASEAYLVGLFEDTNLCAIHAKR  223
```

Range 2: 230 to 332

Score:206 bits(524), Expect:2e-61,
Method:Compositional matrix adjust.,
Identities:103/103(100%), Positives:103/103(100%), Gaps:0/103(0%)

```
Query  1    MSGRGKGGKGLGKGGAKRHRKVLRDNIQGITKPAIRRLARRGGVKRISGLIYEETRGVLK  60
            MSGRGKGGKGLGKGGAKRHRKVLRDNIQGITKPAIRRLARRGGVKRISGLIYEETRGVLK
Sbjct  230  MSGRGKGGKGLGKGGAKRHRKVLRDNIQGITKPAIRRLARRGGVKRISGLIYEETRGVLK  289

Query  61   VFLENVIRDAVTYTEHAKRKTVTAMDVVYALKRQGRTLYGFGG  103
            VFLENVIRDAVTYTEHAKRKTVTAMDVVYALKRQGRTLYGFGG
Sbjct  290  VFLENVIRDAVTYTEHAKRKTVTAMDVVYALKRQGRTLYGFGG  332
```

>uncharacterized protein LOC135242606 [Anguilla rostrata]

Sequence ID: XP_064169827.1 Length: 323
Range 1: 1 to 220

Score:424 bits(1089), Expect:3e-147,
Method:Compositional matrix adjust.,
Identities:214/220(97%), Positives:217/220(98%), Gaps:2/220(0%)

```
Query  1    MSGRGKGGKGLGKGGAKRHRKVLRDNIQGITKPAIRRLARRGGVKRISGLIYEETRGVLK  60
            MSGRGKGGKGLGKGGAKRHRKVLRDNIQGITKPAIRRLARRGGVKRISGLIYEETRGVLK
Sbjct  1    MSGRGKGGKGLGKGGAKRHRKVLRDNIQGITKPAIRRLARRGGVKRISGLIYEETRGVLK  60

Query  61   VFLENVIRDAVTYTEHAKRKTVTAMDVVYALKRQGRTLYGFGG--MARTKQTARKSTGGK  118
            VFLENVIRDAVTYTEHAKRKTVTAMDVVYALKRQGRTLYGFGG  MARTKQTARKSTGGK
Sbjct  61   VFLENVIRDAVTYTEHAKRKTVTAMDVVYALKRQGRTLYGFGGDAMARTKQTARKSTGGK  120

Query  119  APRKQLATKAARKSAPSTGGVKKPHRYRPGTVALREIRRYQKSTELLIRKLPFQRLVREI  178
            APRKQLATKAARKSAP+TGGVKKPHRYRPGTVALREIRRYQKSTELLIRKLPFQRLVREI
Sbjct  121  APRKQLATKAARKSAPATGGVKKPHRYRPGTVALREIRRYQKSTELLIRKLPFQRLVREI  180

Query  179  AQDFKTDLRFQSAAIGALQEASEAYLVGLFEDTNLCAIHA  218
            AQDFKTDLRFQS+A+ ALQEASEAYLVGLFEDTNLCAIHA
Sbjct  181  AQDFKTDLRFQSSAVMALQEASEAYLVGLFEDTNLCAIHA  220
```

Range 2: 221 to 323

Score:205 bits(522), Expect:2e-61,
Method:Compositional matrix adjust.,
Identities:103/103(100%), Positives:103/103(100%), Gaps:0/103(0%)

```
Query  1    MSGRGKGGKGLGKGGAKRHRKVLRDNIQGITKPAIRRLARRGGVKRISGLIYEETRGVLK  60
            MSGRGKGGKGLGKGGAKRHRKVLRDNIQGITKPAIRRLARRGGVKRISGLIYEETRGVLK
Sbjct  221  MSGRGKGGKGLGKGGAKRHRKVLRDNIQGITKPAIRRLARRGGVKRISGLIYEETRGVLK  280

Query  61   VFLENVIRDAVTYTEHAKRKTVTAMDVVYALKRQGRTLYGFGG  103
            VFLENVIRDAVTYTEHAKRKTVTAMDVVYALKRQGRTLYGFGG
Sbjct  281  VFLENVIRDAVTYTEHAKRKTVTAMDVVYALKRQGRTLYGFGG  323
```

>histone H3-like [Rhopalosiphum maidis]
Sequence ID: XP_026813666.1 Length: 244
Range 1: 18 to 244

Score:420 bits(1079), Expect:5e-147,
Method:Compositional matrix adjust.,
Identities:212/227(93%), Positives:216/227(95%), Gaps:5/227(2%)

```
Query  18   RHRKVLRDNIQGITKPAIRRLARRGGVKRISGLIYEETRGVLKVFLENVIRDAVTYTEHA  77
            RHRKVLRDNIQGITKPAIRRLARRGGVKRISGLIYEETRGVLKVFLENVIRDAVTYTEHA
Sbjct  18   RHRKVLRDNIQGITKPAIRRLARRGGVKRISGLIYEETRGVLKVFLENVIRDAVTYTEHA  77
```

```
Query  78   KRKTVTAMDVVYALKRQGRTL-----YGFGGMARTKQTARKSTGGKAPRKQLATKAARKS  132
            KRKTVTAMDVVYALKRQGRT      +    MARTKQTARKSTGGKAPRKQLATKAARKS
Sbjct  78   KRKTVTAMDVVYALKRQGRTFPSNRTHPNSTMARTKQTARKSTGGKAPRKQLATKAARKS  137

Query  133  APSTGGVKKPHRYRPGTVALREIRRYQKSTELLIRKLPFQRLVREIAQDFKTDLRFQSAA  192
            AP+TGGVKKPHRYRPGTVALREIRRYQKSTELLIRKLPFQRLVREIAQDFKTDLRFQS+A
Sbjct  138  APATGGVKKPHRYRPGTVALREIRRYQKSTELLIRKLPFQRLVREIAQDFKTDLRFQSSA  197

Query  193  IGALQEASEAYLVGLFEDTNLCAIHAKRVTIMPKDIQLARRIRGERA  239
            + ALQEASEAYLVGLFEDTNLCAIHAKRVTIMPKDIQLARRIRGERA
Sbjct  198  VMALQEASEAYLVGLFEDTNLCAIHAKRVTIMPKDIQLARRIRGERA  244
```

>unnamed protein product [Angiostrongylus costaricensis]
Sequence ID: VDM64356.1 Length: 357
Range 1: 1 to 236

Score:424 bits(1089), Expect:1e-146,
Method:Compositional matrix adjust.,
Identities:215/238(90%), Positives:218/238(91%), Gaps:15/238(6%)

```
Query  1    MSGRGKGGKGLGKGGAKRHRKVLRDNIQGITKPAIRRLARRGGVKRISGLIYEETRGVLK  60
            MSGRGKGGKGLGKGGAKRHRKVLRDNIQGITKPAIRRLARRGGVKRISGLIYEETRGVLK
Sbjct  1    MSGRGKGGKGLGKGGAKRHRKVLRDNIQGITKPAIRRLARRGGVKRISGLIYEETRGVLK  60

Query  61   VFLENVIRDAVTYTEHAKRKTVTAMDVVYALKRQGRTLYGFG-------------GMART  107
            VFLENVIRDAVTY EHAKRKTVTAMDVVYALKRQGRTLYGF               MART
Sbjct  61   VFLENVIRDAVTYCEHAKRKTVTAMDVVYALKRQGRTLYGFAEERATISFANNRSNMART  120

Query  108  KQTARKSTGGKAPRKQLATKAARKSAPSTGGVKKPHRYRPGTVALREIRRYQKSTELLIR  167
            KQTARKSTGGKAPRKQLATKAARKSAP+TGGVKKPHRYRPGTVALREIRRYQKSTELLIR
Sbjct  121  KQTARKSTGGKAPRKQLATKAARKSAPATGGVKKPHRYRPGTVALREIRRYQKSTELLIR  180

Query  168  KLPFQRLVREIAQDFKTDLRFQSAAIGALQEASEAYLVGLFEDTNLCAIHAKRVTIMP  225
            KLPFQRLVREIAQDFKTDLRFQS+A+ ALQEASEAYLVGLFEDTNLCAIHAK   IMP
Sbjct  181  KLPFQRLVREIAQDFKTDLRFQSSAVMALQEASEAYLVGLFEDTNLCAIHAK--PIMP  236
```

>hypothetical protein SFRURICE_015739, partial [Spodoptera frugiperda]
Sequence ID: KAF9809276.1 Length: 450
Range 1: 119 to 340

Score:426 bits(1095), Expect:2e-146,
Method:Compositional matrix adjust.,
Identities:216/233(93%), Positives:220/233(94%), Gaps:11/233(4%)

```
Query  7    GGKGLGKGGAKRHRKVLRDNIQGITKPAIRRLARRGGVKRISGLIYEETRGVLKVFLENV  66
             GKGLGKGGAKRHRKVLRDNIQGITKPAIRRLARRGGVKRISGLIYEETRGVLKVFLENV
Sbjct  119  SGKGLGKGGAKRHRKVLRDNIQGITKPAIRRLARRGGVKRISGLIYEETRGVLKVFLENV  178
```

```
Query  67   IRDAVTYTEHAKRKTVTAMDVVYALKRQGRTLYGFGGMARTKQTARKSTGGKAPRKQLAT  126
            IRDAVTYTEHAKRKTVTAMDVVYALKRQGRTLY           ARKSTGGKAPRKQLAT
Sbjct  179  IRDAVTYTEHAKRKTVTAMDVVYALKRQGRTLY-----------ARKSTGGKAPRKQLAT  227

Query  127  KAARKSAPSTGGVKKPHRYRPGTVALREIRRYQKSTELLIRKLPFQRLVREIAQDFKTDL  186
            KAARKSAP+TGGVKKPHRYRPGTVALREIRRYQKSTELLIRKLPFQRLVREIAQDFKTDL
Sbjct  228  KAARKSAPATGGVKKPHRYRPGTVALREIRRYQKSTELLIRKLPFQRLVREIAQDFKTDL  287

Query  187  RFQSAAIGALQEASEAYLVGLFEDTNLCAIHAKRVTIMPKDIQLARRIRGERA  239
            RFQS+A+ ALQEASEAYLVGLFEDTNLCAIHAKRVTIMPKDIQLARR+RGERA
Sbjct  288  RFQSSAVMALQEASEAYLVGLFEDTNLCAIHAKRVTIMPKDIQLARRVRGERA  340
```

>hypothetical protein DBR06_SOUSAS20010018 [Sousa chinensis]
Sequence ID: TEA21837.1 Length: 346
Range 1: 1 to 242

Score:420 bits(1080), Expect:1e-145,
Method:Compositional matrix adjust.,
Identities:215/242(89%), Positives:221/242(91%), Gaps:18/242(7%)

```
Query  1    MSGRGKGGKGLGKGGAKRHRKVLRDNIQGITKPAIRRLARRGGVKRISGLIYEETRGVLK  60
            MSGRGKGGKGLGKGGAKRHRKVLRDNIQGITKPAIRRLARRGGVKRISGLIYEETRGVLK
Sbjct  1    MSGRGKGGKGLGKGGAKRHRKVLRDNIQGITKPAIRRLARRGGVKRISGLIYEETRGVLK  60

Query  61   VFLENVIRDAVTYTEHAKRKTVTAMDVVYALKRQGRTLYGFGG-----------------  103
            VFLENVIRDAVTYTEHAKRKTVTAMDVVYALKRQGRTLYGFGG
Sbjct  61   VFLENVIRDAVTYTEHAKRKTVTAMDVVYALKRQGRTLYGFGGDLFLLICLLRFIFVWFL  120

Query  104  -MARTKQTARKSTGGKAPRKQLATKAARKSAPSTGGVKKPHRYRPGTVALREIRRYQKST  162
             MARTKQTARKSTGGKAPRKQLATKAARKSAP+TGGVKKPHRYRPGTVALREIRRYQKST
Sbjct  121  TMARTKQTARKSTGGKAPRKQLATKAARKSAPATGGVKKPHRYRPGTVALREIRRYQKST  180

Query  163  ELLIRKLPFQRLVREIAQDFKTDLRFQSAAIGALQEASEAYLVGLFEDTNLCAIHAKRVT  222
            ELLIRKLPFQRLVREIAQDFKTDLRFQS+A+ ALQEA EAYLVGLFEDTNLCAIHAKR +
Sbjct  181  ELLIRKLPFQRLVREIAQDFKTDLRFQSSAVMALQEACEAYLVGLFEDTNLCAIHAKRKS  240

Query  223  IM  224
            ++
Sbjct  241  LL  242
```

Range 2: 244 to 346

Score:205 bits(522), Expect:4e-61,
Method:Compositional matrix adjust.,
Identities:103/103(100%), Positives:103/103(100%), Gaps:0/103(0%)

```
Query  1    MSGRGKGGKGLGKGGAKRHRKVLRDNIQGITKPAIRRLARRGGVKRISGLIYEETRGVLK  60
```

```
          MSGRGKGGKGLGKGGAKRHRKVLRDNIQGITKPAIRRLARRGGVKRISGLIYEETRGVLK
Sbjct 244 MSGRGKGGKGLGKGGAKRHRKVLRDNIQGITKPAIRRLARRGGVKRISGLIYEETRGVLK 303

Query 61  VFLENVIRDAVTYTEHAKRKTVTAMDVVYALKRQGRTLYGFGG 103
          VFLENVIRDAVTYTEHAKRKTVTAMDVVYALKRQGRTLYGFGG
Sbjct 304 VFLENVIRDAVTYTEHAKRKTVTAMDVVYALKRQGRTLYGFGG 346
```

>histone H3-like centromeric protein CSE4 [Trematomus bernacchii]
Sequence ID: XP_033992940.1 Length: 283
Range 1: 1 to 283

Score:417 bits(1071), Expect:3e-145,
Method:Compositional matrix adjust.,
Identities:234/283(83%), Positives:238/283(84%), Gaps:44/283(15%)

```
Query 1   MSGRGKGGKGLGKGGAKRHRKVLRDNIQGITKPAIRRLARRGGVKRISGLIYEETRGVLK 60
          MSGRGKGGKGLGKGGAKRHRKVLRDNIQGITKPAIRRLARRGGVKRISGLIYEETRGVLK
Sbjct 1   MSGRGKGGKGLGKGGAKRHRKVLRDNIQGITKPAIRRLARRGGVKRISGLIYEETRGVLK 60

Query 61  VFLENVIRDAVTYTEHAKRKTVTAMDVVYALKRQGRTLYGFGG----------------- 103
          VFLENVIRDAVTYTEHAKRKTVTAMDVVYALKRQGRTLYGFGG
Sbjct 61  VFLENVIRDAVTYTEHAKRKTVTAMDVVYALKRQGRTLYGFGGLSCSVTCIHSTNQRRCR 120

Query 104 ---------------------------MARTKQTARKSTGGKAPRKQLATKAARKSAPST 136
                                     MARTKQTARKSTGGKAPRKQLATKAARKSAP+T
Sbjct 121 ACSAYIGDTDKSTALLLFQQRSRPSLTMARTKQTARKSTGGKAPRKQLATKAARKSAPAT 180

Query 137 GGVKKPHRYRPGTVALREIRRYQKSTELLIRKLPFQRLVREIAQDFKTDLRFQSAAIGAL 196
          GGVKKPHRYRPGTVALREIRRYQKSTELLIRKLPFQRLVREIAQDFKTDLRFQS+A+ AL
Sbjct 181 GGVKKPHRYRPGTVALREIRRYQKSTELLIRKLPFQRLVREIAQDFKTDLRFQSSAVMAL 240

Query 197 QEASEAYLVGLFEDTNLCAIHAKRVTIMPKDIQLARRIRGERA 239
          QE+SEAYLVGLFEDTNLCAIHAKRVTIMPKDIQLARRIRGERA
Sbjct 241 QESSEAYLVGLFEDTNLCAIHAKRVTIMPKDIQLARRIRGERA 283
```

>histone H3-like centromeric protein cpar-1 [Chroicocephalus ridibundus]
Sequence ID: XP_063181136.1 Length: 283
Range 1: 1 to 283

Score:416 bits(1069), Expect:6e-145,
Method:Compositional matrix adjust.,
Identities:235/283(83%), Positives:238/283(84%), Gaps:44/283(15%)

```
Query 1   MSGRGKGGKGLGKGGAKRHRKVLRDNIQGITKPAIRRLARRGGVKRISGLIYEETRGVLK 60
          MSGRGKGGKGLGKGGAKRHRKVLRDNIQGITKPAIRRLARRGGVKRISGLIYEETRGVLK
Sbjct 1   MSGRGKGGKGLGKGGAKRHRKVLRDNIQGITKPAIRRLARRGGVKRISGLIYEETRGVLK 60

Query 61  VFLENVIRDAVTYTEHAKRKTVTAMDVVYALKRQGRTLYGFGG----------------- 103
```

```
            VFLENVIRDAVTYTEHAKRKTVTAMDVVYALKRQGRTLYGFGG
Sbjct  61   VFLENVIRDAVTYTEHAKRKTVTAMDVVYALKRQGRTLYGFGGCDKIAMEGSGAEYWCWS  120

Query  104  ---------------------------MARTKQTARKSTGGKAPRKQLATKAARKSAPST  136
                                       MARTKQTARKSTGGKAPRKQLATKAARKSAP+T
Sbjct  121  FAVAAESPRYPQCFSQASLKQQTQGTRMARTKQTARKSTGGKAPRKQLATKAARKSAPAT  180

Query  137  GGVKKPHRYRPGTVALREIRRYQKSTELLIRKLPFQRLVREIAQDFKTDLRFQSAAIGAL  196
            GGVKKPHRYRPGTVALREIRRYQKSTELLIRKLPFQRLVREIAQDFKTDLRFQS+A+ AL
Sbjct  181  GGVKKPHRYRPGTVALREIRRYQKSTELLIRKLPFQRLVREIAQDFKTDLRFQSSAVMAL  240

Query  197  QEASEAYLVGLFEDTNLCAIHAKRVTIMPKDIQLARRIRGERA  239
            QEASEAYLVGLFEDTNLCAIHAKRVTIMPKDIQLARRIRGERA
Sbjct  241  QEASEAYLVGLFEDTNLCAIHAKRVTIMPKDIQLARRIRGERA  283
```

>histone H3-like centromeric protein A [Leucoraja erinaceus]
Sequence ID: XP_055487172.1 Length: 275
Range 1: 1 to 275

Score:415 bits(1066), Expect:2e-144,
Method:Compositional matrix adjust.,
Identities:234/275(85%), Positives:237/275(86%), Gaps:36/275(13%)

```
Query  1    MSGRGKGGKGLGKGGAKRHRKVLRDNIQGITKPAIRRLARRGGVKRISGLIYEETRGVLK  60
            MSGRGKGGKGLGKGGAKRHRKVLRDNIQGITKPAIRRLARRGGVKRISGLIYEETRGVLK
Sbjct  1    MSGRGKGGKGLGKGGAKRHRKVLRDNIQGITKPAIRRLARRGGVKRISGLIYEETRGVLK  60

Query  61   VFLENVIRDAVTYTEHAKRKTVTAMDVVYALKRQGRTLYGFGG-----------------  103
            VFLENV RDAVTYTEHAKRKTVTAMDVVYALKRQGRTLYGFGG
Sbjct  61   VFLENVSRDAVTYTEHAKRKTVTAMDVVYALKRQGRTLYGFGGVCRASPTPPGRPASDLL  120

Query  104  -------------------MARTKQTARKSTGGKAPRKQLATKAARKSAPSTGGVKKPHR  144
                               MARTKQTARKSTGGKAPRKQLATKAARKSAP+TGGVKKPHR
Sbjct  121  KQPPGVDKPAATRRDGTSRMARTKQTARKSTGGKAPRKQLATKAARKSAPATGGVKKPHR  180

Query  145  YRPGTVALREIRRYQKSTELLIRKLPFQRLVREIAQDFKTDLRFQSAAIGALQEASEAYL  204
            YRPGTVALREIRRYQKSTELLIRKLPFQRLVREIAQDFKTDLRFQS+A+ ALQEASEAYL
Sbjct  181  YRPGTVALREIRRYQKSTELLIRKLPFQRLVREIAQDFKTDLRFQSSAVMALQEASEAYL  240

Query  205  VGLFEDTNLCAIHAKRVTIMPKDIQLARRIRGERA  239
            VGLFEDTNLCAIHAKRVTIMPKDIQLARRIRGERA
Sbjct  241  VGLFEDTNLCAIHAKRVTIMPKDIQLARRIRGERA  275
```

>hypothetical protein EI555_001631, partial [Monodon monoceros]
Sequence ID: TKC35984.1 Length: 645
Range 1: 294 to 515

Score:428 bits(1100), Expect:2e-144,

Method:Compositional matrix adjust.,
Identities:214/222(96%), Positives:218/222(98%), Gaps:0/222(0%)

```
Query  1    MSGRGKGGKGLGKGGAKRHRKVLRDNIQGITKPAIRRLARRGGVKRISGLIYEETRGVLK  60
            MSGRGKGGKGLGKGGAKRHRKVLRDNIQGITKPAIRRLARRGGVKRISGLIYEETRGVLK
Sbjct  294  MSGRGKGGKGLGKGGAKRHRKVLRDNIQGITKPAIRRLARRGGVKRISGLIYEETRGVLK  353

Query  61   VFLENVIRDAVTYTEHAKRKTVTAMDVVYALKRQGRTLYGFGGMARTKQTARKSTGGKAP  120
            VFLENVIRDAVTYTEHAKRKTVTAMDVVYALKRQGRTLYGFG MARTKQTARKSTGGKAP
Sbjct  354  VFLENVIRDAVTYTEHAKRKTVTAMDVVYALKRQGRTLYGFGVMARTKQTARKSTGGKAP  413

Query  121  RKQLATKAARKSAPSTGGVKKPHRYRPGTVALREIRRYQKSTELLIRKLPFQRLVREIAQ  180
            RKQLATKAARKSAP+TGGVKKPHRYRPGTVALREIRRYQKSTELLIRKLPFQRLVREIAQ
Sbjct  414  RKQLATKAARKSAPATGGVKKPHRYRPGTVALREIRRYQKSTELLIRKLPFQRLVREIAQ  473

Query  181  DFKTDLRFQSAAIGALQEASEAYLVGLFEDTNLCAIHAKRVT  222
            DFKTDLRFQS+A+ ALQEA EAYLVGLFEDTNLCAIHAKR +
Sbjct  474  DFKTDLRFQSSAVMALQEACEAYLVGLFEDTNLCAIHAKRFS  515
```

Range 2: 132 to 267

Score:265 bits(677), Expect:5e-81,
Method:Compositional matrix adjust.,
Identities:131/136(96%), Positives:134/136(98%), Gaps:0/136(0%)

```
Query  104  MARTKQTARKSTGGKAPRKQLATKAARKSAPSTGGVKKPHRYRPGTVALREIRRYQKSTE  163
            MARTKQTARKSTGGKAPRKQLATKAARKSAP+TGGVKKPHRYRPGTVALREIRRYQKSTE
Sbjct  132  MARTKQTARKSTGGKAPRKQLATKAARKSAPATGGVKKPHRYRPGTVALREIRRYQKSTE  191

Query  164  LLIRKLPFQRLVREIAQDFKTDLRFQSAAIGALQEASEAYLVGLFEDTNLCAIHAKRVTI  223
            LLIRKLPFQRLVREIAQDFKTDLRFQS+A+ ALQEA EAYLVGLFEDTNLCAIHAKRVTI
Sbjct  192  LLIRKLPFQRLVREIAQDFKTDLRFQSSAVMALQEACEAYLVGLFEDTNLCAIHAKRVTI  251

Query  224  MPKDIQLARRIRGERA  239
            MPKDIQLARRIRGERA
Sbjct  252  MPKDIQLARRIRGERA  267
```

Range 3: 543 to 645

Score:207 bits(526), Expect:9e-59,
Method:Compositional matrix adjust.,
Identities:103/103(100%), Positives:103/103(100%), Gaps:0/103(0%)

```
Query  1    MSGRGKGGKGLGKGGAKRHRKVLRDNIQGITKPAIRRLARRGGVKRISGLIYEETRGVLK  60
            MSGRGKGGKGLGKGGAKRHRKVLRDNIQGITKPAIRRLARRGGVKRISGLIYEETRGVLK
Sbjct  543  MSGRGKGGKGLGKGGAKRHRKVLRDNIQGITKPAIRRLARRGGVKRISGLIYEETRGVLK  602

Query  61   VFLENVIRDAVTYTEHAKRKTVTAMDVVYALKRQGRTLYGFGG  103
```

```
           VFLENVIRDAVTYTEHAKRKTVTAMDVVYALKRQGRTLYGFGG
Sbjct  603  VFLENVIRDAVTYTEHAKRKTVTAMDVVYALKRQGRTLYGFGG  645
```

>histone H3-like centromeric protein CSE4 [Nothoprocta perdicaria]
Sequence ID: XP_025909233.1 Length: 281
Range 1: 1 to 281

Score:414 bits(1064), Expect:3e-144,
Method:Compositional matrix adjust.,
Identities:234/281(83%), Positives:237/281(84%), Gaps:42/281(14%)

```
Query  1    MSGRGKGGKGLGKGGAKRHRKVLRDNIQGITKPAIRRLARRGGVKRISGLIYEETRGVLK  60
            MSGRGKGGKGLGKGGAKRHRKVLRDNIQGITKPAIRRLARRGGVKRISGLIYEETRGVLK
Sbjct  1    MSGRGKGGKGLGKGGAKRHRKVLRDNIQGITKPAIRRLARRGGVKRISGLIYEETRGVLK  60

Query  61   VFLENVIRDAVTYTEHAKRKTVTAMDVVYALKRQGRTLYGF-------------------  101
            VFLENVIRDAVTYTEHAKRKTVTAMDVVYALKRQGRTLYGF
Sbjct  61   VFLENVIRDAVTYTEHAKRKTVTAMDVVYALKRQGRTLYGFGVTATDASQSRSRALHKSR  120

Query  102  -----------------------GGMARTKQTARKSTGGKAPRKQLATKAARKSAPSTGG  138
                                   G MARTKQTARKSTGGKAPRKQLATKAARKSAP+TGG
Sbjct  121  RRYRQRSFAGVPPEERQXQRGAPGTMARTKQTARKSTGGKAPRKQLATKAARKSAPATGG  180

Query  139  VKKPHRYRPGTVALREIRRYQKSTELLIRKLPFQRLVREIAQDFKTDLRFQSAAIGALQE  198
            VKKPHRYRPGTVALREIRRYQKSTELLIRKLPFQRLVREIAQDFKTDLRFQS+A+ ALQE
Sbjct  181  VKKPHRYRPGTVALREIRRYQKSTELLIRKLPFQRLVREIAQDFKTDLRFQSSAVMALQE  240

Query  199  ASEAYLVGLFEDTNLCAIHAKRVTIMPKDIQLARRIRGERA  239
            ASEAYLVGLFEDTNLCAIHAKRVTIMPKDIQLARRIRGERA
Sbjct  241  ASEAYLVGLFEDTNLCAIHAKRVTIMPKDIQLARRIRGERA  281
```

>uncharacterized protein LOC135085844 [Ostrinia nubilalis]
Sequence ID: XP_063836701.1 Length: 326
Range 1: 1 to 225

Score:416 bits(1069), Expect:3e-144,
Method:Compositional matrix adjust.,
Identities:212/225(94%), Positives:217/225(96%), Gaps:3/225(1%)

```
Query  1    MSGRGKGGKGLGKGGAKRHRKVLRDNIQGITKPAIRRLARRGGVKRISGLIYEETRGVLK  60
            M+GRGKGGKGLGKGGAKRHRKVLRDNIQGITKPAIRRLARRGGVKRISGLIYEETRGVLK
Sbjct  1    MTGRGKGGKGLGKGGAKRHRKVLRDNIQGITKPAIRRLARRGGVKRISGLIYEETRGVLK  60

Query  61   VFLENVIRDAVTYTEHAKRKTVTAMDVVYALKRQGRTLYG---FGGMARTKQTARKSTGG  117
            VFLENVIRDAVTYTEHAKRKTVTAMDVVYALKRQGRTLY       MARTKQTARKSTGG
Sbjct  61   VFLENVIRDAVTYTEHAKRKTVTAMDVVYALKRQGRTLYVVLRLRTMARTKQTARKSTGG  120

Query  118  KAPRKQLATKAARKSAPSTGGVKKPHRYRPGTVALREIRRYQKSTELLIRKLPFQRLVRE  177
```

```
           KAPRKQLATKAARKSAP+TGGVKKPHRYRPGTVALREIRRYQKSTELLIRKLPFQRLVRE
Sbjct  121  KAPRKQLATKAARKSAPATGGVKKPHRYRPGTVALREIRRYQKSTELLIRKLPFQRLVRE  180

Query  178  IAQDFKTDLRFQSAAIGALQEASEAYLVGLFEDTNLCAIHAKRVT  222
           IAQDFKTDLRFQS+A+ ALQEASEAYLVGLFEDTNLCAIHAKR+T
Sbjct  181  IAQDFKTDLRFQSSAVMALQEASEAYLVGLFEDTNLCAIHAKRMT  225
```

Range 2: 224 to 326

Score:205 bits(521), Expect:4e-61,
Method:Compositional matrix adjust.,
Identities:102/103(99%), Positives:103/103(100%), Gaps:0/103(0%)

```
Query  1    MSGRGKGGKGLGKGGAKRHRKVLRDNIQGITKPAIRRLARRGGVKRISGLIYEETRGVLK  60
           M+GRGKGGKGLGKGGAKRHRKVLRDNIQGITKPAIRRLARRGGVKRISGLIYEETRGVLK
Sbjct  224  MTGRGKGGKGLGKGGAKRHRKVLRDNIQGITKPAIRRLARRGGVKRISGLIYEETRGVLK  283

Query  61   VFLENVIRDAVTYTEHAKRKTVTAMDVVYALKRQGRTLYGFGG  103
           VFLENVIRDAVTYTEHAKRKTVTAMDVVYALKRQGRTLYGFGG
Sbjct  284  VFLENVIRDAVTYTEHAKRKTVTAMDVVYALKRQGRTLYGFGG  326
```

>uncharacterized protein LOC112664031 [Canis lupus dingo]
Sequence ID: XP_035556775.1 Length: 295
Range 1: 67 to 295

Score:414 bits(1065), Expect:4e-144,
Method:Compositional matrix adjust.,
Identities:212/229(93%), Positives:215/229(93%), Gaps:7/229(3%)

```
Query  18   RHRKVLRDNIQGITKPAIRRLARRGGVKRISGLIYEETRGVLKVFLENVIRDAVTYTEHA  77
           RHRKVLRDNIQGITKPAIRRLARRGGVKRISGLIYEETRGVLKVFLENVIRDAVTYTEHA
Sbjct  67   RHRKVLRDNIQGITKPAIRRLARRGGVKRISGLIYEETRGVLKVFLENVIRDAVTYTEHA  126

Query  78   KRKTVTAMDVVYALKRQGR-------TLYGFGGMARTKQTARKSTGGKAPRKQLATKAAR  130
           KRKTVTAMDVVYALKRQGR           G   MARTKQTARKSTGGKAPRKQLATKAAR
Sbjct  127  KRKTVTAMDVVYALKRQGRGAPTKMHVRSGRFAMARTKQTARKSTGGKAPRKQLATKAAR  186

Query  131  KSAPSTGGVKKPHRYRPGTVALREIRRYQKSTELLIRKLPFQRLVREIAQDFKTDLRFQS  190
           KSAP+TGGVKKPHRYRPGTVALREIRRYQKSTELLIRKLPFQRLVREIAQDFKTDLRFQS
Sbjct  187  KSAPATGGVKKPHRYRPGTVALREIRRYQKSTELLIRKLPFQRLVREIAQDFKTDLRFQS  246

Query  191  AAIGALQEASEAYLVGLFEDTNLCAIHAKRVTIMPKDIQLARRIRGERA  239
           +A+ ALQEASEAYLVGLFEDTNLCAIHAKRVTIMPKDIQLARRIRGERA
Sbjct  247  SAVMALQEASEAYLVGLFEDTNLCAIHAKRVTIMPKDIQLARRIRGERA  295
```

```
>uncharacterized protein LOC133242159 [Bos javanicus]
Sequence ID: XP_061264213.1 Length: 293
Range 1: 65 to 293

Score:414 bits(1064), Expect:5e-144,
Method:Compositional matrix adjust.,
Identities:212/229(93%), Positives:215/229(93%), Gaps:7/229(3%)

Query  18   RHRKVLRDNIQGITKPAIRRLARRGGVKRISGLIYEETRGVLKVFLENVIRDAVTYTEHA  77
            RHRKVLRDNIQGITKPAIRRLARRGGVKRISGLIYEETRGVLKVFLENVIRDAVTYTEHA
Sbjct  65   RHRKVLRDNIQGITKPAIRRLARRGGVKRISGLIYEETRGVLKVFLENVIRDAVTYTEHA  124

Query  78   KRKTVTAMDVVYALKRQGR-------TLYGFGGMARTKQTARKSTGGKAPRKQLATKAAR  130
            KRKTVTAMDVVYALKRQGR          G   MARTKQTARKSTGGKAPRKQLATKAAR
Sbjct  125  KRKTVTAMDVVYALKRQGRRAPTQLSCRRGRFAMARTKQTARKSTGGKAPRKQLATKAAR  184

Query  131  KSAPSTGGVKKPHRYRPGTVALREIRRYQKSTELLIRKLPFQRLVREIAQDFKTDLRFQS  190
            KSAP+TGGVKKPHRYRPGTVALREIRRYQKSTELLIRKLPFQRLVREIAQDFKTDLRFQS
Sbjct  185  KSAPATGGVKKPHRYRPGTVALREIRRYQKSTELLIRKLPFQRLVREIAQDFKTDLRFQS  244

Query  191  AAIGALQEASEAYLVGLFEDTNLCAIHAKRVTIMPKDIQLARRIRGERA  239
            +A+ ALQEASEAYLVGLFEDTNLCAIHAKRVTIMPKDIQLARRIRGERA
Sbjct  245  SAVMALQEASEAYLVGLFEDTNLCAIHAKRVTIMPKDIQLARRIRGERA  293
```

```
>uncharacterized protein LOC119817094 [Arvicola amphibius]
Sequence ID: XP_041911258.1 Length: 395
Range 1: 18 to 245

Score:417 bits(1071), Expect:2e-143,
Method:Compositional matrix adjust.,
Identities:211/228(93%), Positives:214/228(93%), Gaps:7/228(3%)

Query  18   RHRKVLRDNIQGITKPAIRRLARRGGVKRISGLIYEETRGVLKVFLENVIRDAVTYTEHA  77
            RHRKVLRDNIQGITKPAIRRLARRGGVKRISGLIYEETRGVLKVFLENVIRDAVTYTEHA
Sbjct  18   RHRKVLRDNIQGITKPAIRRLARRGGVKRISGLIYEETRGVLKVFLENVIRDAVTYTEHA  77

Query  78   KRKTVTAMDVVYALKRQGRTL-------YGFGGMARTKQTARKSTGGKAPRKQLATKAAR  130
            KRKTVTAMDVVYALKRQGR          G   MARTKQTARKSTGGKAPRKQLATKAAR
Sbjct  78   KRKTVTAMDVVYALKRQGRPAPLAVSVNSGSSDMARTKQTARKSTGGKAPRKQLATKAAR  137

Query  131  KSAPSTGGVKKPHRYRPGTVALREIRRYQKSTELLIRKLPFQRLVREIAQDFKTDLRFQS  190
            KSAP+TGGVKKPHRYRPGTVALREIRRYQKSTELLIRKLPFQRLVREIAQDFKTDLRFQS
Sbjct  138  KSAPATGGVKKPHRYRPGTVALREIRRYQKSTELLIRKLPFQRLVREIAQDFKTDLRFQS  197

Query  191  AAIGALQEASEAYLVGLFEDTNLCAIHAKRVTIMPKDIQLARRIRGER  238
            +A+ ALQEASEAYLVGLFEDTNLCAIHAKRVTIMPKDIQLARRIRGER
Sbjct  198  SAVMALQEASEAYLVGLFEDTNLCAIHAKRVTIMPKDIQLARRIRGER  245
```

Range 2: 208 to 395

Score:272 bits(696), Expect:1e-86,
Method:Compositional matrix adjust.,
Identities:141/188(75%), Positives:152/188(80%), Gaps:8/188(4%)

```
Query  60   KVFLENVIRDAVTYTEHAKRKTVTAMDVVYALKRQGRTLYG--------FGGMARTKQTA  111
            + +L  +  D      HAKR T+   D+  A + +G   +            MARTKQTA
Sbjct  208  EAYLVGLFEDTNLCAIHAKRVTIMPKDIQLARRIRGERDFSAVYLLRSLSPDMARTKQTA  267

Query  112  RKSTGGKAPRKQLATKAARKSAPSTGGVKKPHRYRPGTVALREIRRYQKSTELLIRKLPF  171
            RKSTGGKAPRKQLATKAARKSAP+TGGVKKPHRYRPGTVALREIRRYQKSTELLIRKLPF
Sbjct  268  RKSTGGKAPRKQLATKAARKSAPATGGVKKPHRYRPGTVALREIRRYQKSTELLIRKLPF  327

Query  172  QRLVREIAQDFKTDLRFQSAAIGALQEASEAYLVGLFEDTNLCAIHAKRVTIMPKDIQLA  231
            QRLVREIAQDFKTDLRFQS+A+ ALQEA EAYLVGLFEDTNLCAIHAKRVTIMPKDIQLA
Sbjct  328  QRLVREIAQDFKTDLRFQSSAVMALQEACEAYLVGLFEDTNLCAIHAKRVTIMPKDIQLA  387

Query  232  RRIRGERA  239
            RRIRGERA
Sbjct  388  RRIRGERA  395
```

>LOW QUALITY PROTEIN: uncharacterized protein LOC116544226 [Sapajus apella]
Sequence ID: XP_032126308.1 Length: 428
Range 1: 172 to 428

Score:418 bits(1074), Expect:2e-143,
Method:Compositional matrix adjust.,
Identities:215/257(84%), Positives:218/257(84%), Gaps:35/257(13%)

```
Query  18   RHRKVLRDNIQGITKPAIRRLARRGGVKRISGLIYEETRGVLKVFLENVIRDAVTYTEHA  77
            RHRKVLRDNIQGITKPAIRRLARRGGVKRISGLIYEETRGVLKVFLENVIRDAVTYTEHA
Sbjct  172  RHRKVLRDNIQGITKPAIRRLARRGGVKRISGLIYEETRGVLKVFLENVIRDAVTYTEHA  231

Query  78   KRKTVTAMDVVYALKRQGRTLYGFG-----------------------------------  102
            KRKTVTAMDVVYALKRQGRTLYGF
Sbjct  232  KRKTVTAMDVVYALKRQGRTLYGFDVTRHSLLSNQNARAAINTGMVNFFFSFAGSSWVYL  291

Query  103  GMARTKQTARKSTGGKAPRKQLATKAARKSAPSTGGVKKPHRYRPGTVALREIRRYQKST  162
             MARTKQTARKSTGGKAPRKQLATKAARKSAP+TGGVKKPHRYRPGTVALREIRRYQKST
Sbjct  292  AMARTKQTARKSTGGKAPRKQLATKAARKSAPATGGVKKPHRYRPGTVALREIRRYQKST  351

Query  163  ELLIRKLPFQRLVREIAQDFKTDLRFQSAAIGALQEASEAYLVGLFEDTNLCAIHAKRVT  222
            ELLIRKLPFQRLVREIAQDFKTDLRFQS+A+ ALQEA EAYLVGLFEDTNLCAIHAKRVT
Sbjct  352  ELLIRKLPFQRLVREIAQDFKTDLRFQSSAVMALQEACEAYLVGLFEDTNLCAIHAKRVT  411

Query  223  IMPKDIQLARRIRGERA  239
            IMPKDIQLARRIRGERA
Sbjct  412  IMPKDIQLARRIRGERA  428
```

Range 2: 1 to 136

Score:266 bits(680), Expect:7e-84,
Method:Compositional matrix adjust.,
Identities:131/136(96%), Positives:134/136(98%), Gaps:0/136(0%)

```
Query  104  MARTKQTARKSTGGKAPRKQLATKAARKSAPSTGGVKKPHRYRPGTVALREIRRYQKSTE  163
            MARTKQTARKSTGGKAPRKQLATKAARKSAP+TGGVKKPHRYRPGTVALREIRRYQKSTE
Sbjct  1    MARTKQTARKSTGGKAPRKQLATKAARKSAPATGGVKKPHRYRPGTVALREIRRYQKSTE  60

Query  164  LLIRKLPFQRLVREIAQDFKTDLRFQSAAIGALQEASEAYLVGLFEDTNLCAIHAKRVTI  223
            LLIRKLPFQRLVREIAQDFKTDLRFQS+A+ ALQEA EAYLVGLFEDTNLCAIHAKRVTI
Sbjct  61   LLIRKLPFQRLVREIAQDFKTDLRFQSSAVMALQEACEAYLVGLFEDTNLCAIHAKRVTI  120

Query  224  MPKDIQLARRIRGERA  239
            MPKDIQLARRIRGERA
Sbjct  121  MPKDIQLARRIRGERA  136
```

>uncharacterized protein LOC129781780 [Toxorhynchites rutilus septentrionalis]
Sequence ID: XP_055645334.1 Length: 321
Range 1: 1 to 216

Score:413 bits(1061), Expect:5e-143,
Method:Compositional matrix adjust.,
Identities:207/216(96%), Positives:212/216(98%), Gaps:0/216(0%)

```
Query  1    MSGRGKGGKGLGKGGAKRHRKVLRDNIQGITKPAIRRLARRGGVKRISGLIYEETRGVLK  60
            M+GRGKGGKGLGKGGAKRHRKVLRDNIQGITKPAIRRLARRGGVKRISGLIYEETRGVLK
Sbjct  1    MTGRGKGGKGLGKGGAKRHRKVLRDNIQGITKPAIRRLARRGGVKRISGLIYEETRGVLK  60

Query  61   VFLENVIRDAVTYTEHAKRKTVTAMDVVYALKRQGRTLYGFGGMARTKQTARKSTGGKAP  120
            VFLENVIRDAVTYTEHAKRKTVTAMDVVYALKRQGRTLYGFG MARTKQTARKSTGGKAP
Sbjct  61   VFLENVIRDAVTYTEHAKRKTVTAMDVVYALKRQGRTLYGFGAMARTKQTARKSTGGKAP  120

Query  121  RKQLATKAARKSAPSTGGVKKPHRYRPGTVALREIRRYQKSTELLIRKLPFQRLVREIAQ  180
            RKQLATKAARKSAP+TGGVKKPHRYRPGTVALREIRRYQKSTELLIRKLPFQRLVREIAQ
Sbjct  121  RKQLATKAARKSAPATGGVKKPHRYRPGTVALREIRRYQKSTELLIRKLPFQRLVREIAQ  180

Query  181  DFKTDLRFQSAAIGALQEASEAYLVGLFEDTNLCAI  216
            DFKTDLRFQS+A+ ALQEASEAYLVGLFEDTNL  +
Sbjct  181  DFKTDLRFQSSAVMALQEASEAYLVGLFEDTNLYIV  216
```

Range 2: 219 to 321

Score:204 bits(519), Expect:6e-61,
Method:Compositional matrix adjust.,

```
Identities:102/103(99%), Positives:103/103(100%), Gaps:0/103(0%)

Query  1    MSGRGKGGKGLGKGGAKRHRKVLRDNIQGITKPAIRRLARRGGVKRISGLIYEETRGVLK  60
            M+GRGKGGKGLGKGGAKRHRKVLRDNIQGITKPAIRRLARRGGVKRISGLIYEETRGVLK
Sbjct  219  MTGRGKGGKGLGKGGAKRHRKVLRDNIQGITKPAIRRLARRGGVKRISGLIYEETRGVLK  278

Query  61   VFLENVIRDAVTYTEHAKRKTVTAMDVVYALKRQGRTLYGFGG  103
            VFLENVIRDAVTYTEHAKRKTVTAMDVVYALKRQGRTLYGFGG
Sbjct  279  VFLENVIRDAVTYTEHAKRKTVTAMDVVYALKRQGRTLYGFGG  321
```

>uncharacterized protein LOC135181613 [Pogoniulus pusillus]
Sequence ID: XP_064010917.1 Length: 327
Range 1: 1 to 222

Score:412 bits(1060), Expect:7e-143,
Method:Compositional matrix adjust.,
Identities:209/222(94%), Positives:213/222(95%), Gaps:6/222(2%)

```
Query  1    MSGRGKGGKGLGKGGAKRHRKVLRDNIQGITKPAIRRLARRGGVKRISGLIYEETRGVLK  60
            MSGRGKGGKGLGKGGAKRHRKVLRDNIQGITKPAIRRLARRGGVKRISGLIYEETRGVLK
Sbjct  1    MSGRGKGGKGLGKGGAKRHRKVLRDNIQGITKPAIRRLARRGGVKRISGLIYEETRGVLK  60

Query  61   VFLENVIRDAVTYTEHAKRKTVTAMDVVYALKRQGRTLYGFGG------MARTKQTARKS  114
            VFLENVIRDAVTYTEHAKRKTVTAMDVVYALKRQGRTLYGFG       MARTKQTARKS
Sbjct  61   VFLENVIRDAVTYTEHAKRKTVTAMDVVYALKRQGRTLYGFGAVWCFYTMARTKQTARKS  120

Query  115  TGGKAPRKQLATKAARKSAPSTGGVKKPHRYRPGTVALREIRRYQKSTELLIRKLPFQRL  174
            TGGKAPRKQLATKAARKSAP+TGGVKKPHRYRPGTVALREIRRYQKSTELLIRKLPFQRL
Sbjct  121  TGGKAPRKQLATKAARKSAPATGGVKKPHRYRPGTVALREIRRYQKSTELLIRKLPFQRL  180

Query  175  VREIAQDFKTDLRFQSAAIGALQEASEAYLVGLFEDTNLCAI  216
            VREIAQDFKTDLRFQS+A+ ALQEASEAYLVGLFEDTNLC +
Sbjct  181  VREIAQDFKTDLRFQSSAVMALQEASEAYLVGLFEDTNLCIV  222
```

Range 2: 225 to 327

Score:206 bits(523), Expect:2e-61,
Method:Compositional matrix adjust.,
Identities:103/103(100%), Positives:103/103(100%), Gaps:0/103(0%)

```
Query  1    MSGRGKGGKGLGKGGAKRHRKVLRDNIQGITKPAIRRLARRGGVKRISGLIYEETRGVLK  60
            MSGRGKGGKGLGKGGAKRHRKVLRDNIQGITKPAIRRLARRGGVKRISGLIYEETRGVLK
Sbjct  225  MSGRGKGGKGLGKGGAKRHRKVLRDNIQGITKPAIRRLARRGGVKRISGLIYEETRGVLK  284

Query  61   VFLENVIRDAVTYTEHAKRKTVTAMDVVYALKRQGRTLYGFGG  103
            VFLENVIRDAVTYTEHAKRKTVTAMDVVYALKRQGRTLYGFGG
Sbjct  285  VFLENVIRDAVTYTEHAKRKTVTAMDVVYALKRQGRTLYGFGG  327
```

>uncharacterized protein LOC132221136 [Myotis daubentonii]
Sequence ID: XP_059531074.1 Length: 467
Range 1: 195 to 467

Score:418 bits(1074), Expect:7e-143,
Method:Compositional matrix adjust.,
Identities:234/273(86%), Positives:237/273(86%), Gaps:34/273(12%)

```
Query  1    MSGRGKGGKGLGKGGAKRHRKVLRDNIQGITKPAIRRLARRGGVKRISGLIYEETRGVLK  60
            MSGRGKGGKGLGKGGAKRHRKVLRDNIQGITKPAIRRLARRGGVKRISGLIYEETRGVLK
Sbjct  195  MSGRGKGGKGLGKGGAKRHRKVLRDNIQGITKPAIRRLARRGGVKRISGLIYEETRGVLK  254

Query  61   VFLENVIRDAVTYTEHAKRKTVTAMDVVYALKRQGRTLYGFGG-----------------  103
            VFLENVIRDAVTYTEHAKRKTVTAMDVVYALKRQGRTLYGFGG
Sbjct  255  VFLENVIRDAVTYTEHAKRKTVTAMDVVYALKRQGRTLYGFGGVGLCPHSSPNASLRSGP  314

Query  104  -----------------MARTKQTARKSTGGKAPRKQLATKAARKSAPSTGGVKKPHRYR  146
                             MARTKQTARKSTGGKAPRKQLATKAARKSAP+TGGVKKPHRYR
Sbjct  315  LPEPPGGRLSFTGEILSMARTKQTARKSTGGKAPRKQLATKAARKSAPATGGVKKPHRYR  374

Query  147  PGTVALREIRRYQKSTELLIRKLPFQRLVREIAQDFKTDLRFQSAAIGALQEASEAYLVG  206
            PGTVALREIRRYQKSTELLIRKLPFQRLVREIAQDFKTDLRFQS+A+ ALQEA EAYLVG
Sbjct  375  PGTVALREIRRYQKSTELLIRKLPFQRLVREIAQDFKTDLRFQSSAVMALQEACEAYLVG  434

Query  207  LFEDTNLCAIHAKRVTIMPKDIQLARRIRGERA  239
            LFEDTNLCAIHAKRVTIMPKDIQLARRIRGERA
Sbjct  435  LFEDTNLCAIHAKRVTIMPKDIQLARRIRGERA  467
```

Range 2: 36 to 171

Score:265 bits(677), Expect:8e-83,
Method:Compositional matrix adjust.,
Identities:131/136(96%), Positives:134/136(98%), Gaps:0/136(0%)

```
Query  104  MARTKQTARKSTGGKAPRKQLATKAARKSAPSTGGVKKPHRYRPGTVALREIRRYQKSTE  163
            MARTKQTARKSTGGKAPRKQLATKAARKSAP+TGGVKKPHRYRPGTVALREIRRYQKSTE
Sbjct  36   MARTKQTARKSTGGKAPRKQLATKAARKSAPATGGVKKPHRYRPGTVALREIRRYQKSTE  95

Query  164  LLIRKLPFQRLVREIAQDFKTDLRFQSAAIGALQEASEAYLVGLFEDTNLCAIHAKRVTI  223
            LLIRKLPFQRLVREIAQDFKTDLRFQS+A+ ALQEA EAYLVGLFEDTNLCAIHAKRVTI
Sbjct  96   LLIRKLPFQRLVREIAQDFKTDLRFQSSAVMALQEACEAYLVGLFEDTNLCAIHAKRVTI  155

Query  224  MPKDIQLARRIRGERA  239
            MPKDIQLARRIRGERA
Sbjct  156  MPKDIQLARRIRGERA  171
```

```
>uncharacterized protein LOC132999314 [Limanda limanda]
Sequence ID: XP_060924979.1 Length: 288
Range 1: 50 to 288

Score:410 bits(1054), Expect:2e-142,
Method:Compositional matrix adjust.,
Identities:212/239(89%), Positives:215/239(89%), Gaps:17/239(7%)

Query  18   RHRKVLRDNIQGITKPAIRRLARRGGVKRISGLIYEETRGVLKVFLENVIRDAVTYTEHA  77
            RHRKVLRDNIQGITKPAIRRLARRGGVKRISGLIYEETRGVLKVFLENVIRDAVTYTEHA
Sbjct  50   RHRKVLRDNIQGITKPAIRRLARRGGVKRISGLIYEETRGVLKVFLENVIRDAVTYTEHA  109

Query  78   KRKTVTAMDVVYALKRQG-----------------RTLYGF-GGMARTKQTARKSTGGKAP  120
            KRKTVTAMDVVYALKRQG                  T Y     MARTKQTARKSTGGKAP
Sbjct  110  KRKTVTAMDVVYALKRQGPPASPRLRLHKKRSATSTSYSLLVTMARTKQTARKSTGGKAP  169

Query  121  RKQLATKAARKSAPSTGGVKKPHRYRPGTVALREIRRYQKSTELLIRKLPFQRLVREIAQ  180
            RKQLATKAARKSAP+TGGVKKPHRYRPGTVALREIRRYQKSTELLIRKLPFQRLVREIAQ
Sbjct  170  RKQLATKAARKSAPATGGVKKPHRYRPGTVALREIRRYQKSTELLIRKLPFQRLVREIAQ  229

Query  181  DFKTDLRFQSAAIGALQEASEAYLVGLFEDTNLCAIHAKRVTIMPKDIQLARRIRGERA  239
            DFKTDLRFQS+A+ ALQEASEAYLVGLFEDTNLCAIHAKRVTIMPKDIQLARRIRGERA
Sbjct  230  DFKTDLRFQSSAVMALQEASEAYLVGLFEDTNLCAIHAKRVTIMPKDIQLARRIRGERA  288


>uncharacterized protein LOC110977866 [Acanthaster planci]
Sequence ID: XP_022088067.1 Length: 285
Range 1: 1 to 285

Score:409 bits(1051), Expect:3e-142,
Method:Compositional matrix adjust.,
Identities:232/285(81%), Positives:236/285(82%), Gaps:46/285(16%)

Query  1    MSGRGKGGKGLGKGGAKRHRKVLRDNIQGITKPAIRRLARRGGVKRISGLIYEETRGVLK  60
            MSGRGKGGKGLGKGGAKRHRKVLRDNIQGITKPAIRRLARRGGVKRISGLIYEETRGVLK
Sbjct  1    MSGRGKGGKGLGKGGAKRHRKVLRDNIQGITKPAIRRLARRGGVKRISGLIYEETRGVLK  60

Query  61   VFLENVIRDAVTYTEHAKRKTVTAMDVVYALKRQGRTLYGFGG-----------------  103
            VFLENVIRDAVTY EHAKRKTVTAMDVVYALKRQGRTLYGFG
Sbjct  61   VFLENVIRDAVTYCEHAKRKTVTAMDVVYALKRQGRTLYGFGDKHVRSALTLEKWREGFT  120

Query  104  -----------------------------MARTKQTARKSTGGKAPRKQLATKAARKSAP  134
                                         MARTKQTARKSTGGKAPRKQLATKAARKSAP
Sbjct  121  HPPITALTERTNGTVLRRPIRNKPKHQSTMARTKQTARKSTGGKAPRKQLATKAARKSAP  180

Query  135  STGGVKKPHRYRPGTVALREIRRYQKSTELLIRKLPFQRLVREIAQDFKTDLRFQSAAIG  194
            +TGGVKKPHRYRPGTVALREIRRYQKSTELLIRKLPFQRLVREIAQDFKT+LRFQS+A+
Sbjct  181  ATGGVKKPHRYRPGTVALREIRRYQKSTELLIRKLPFQRLVREIAQDFKTELRFQSSAVM  240

Query  195  ALQEASEAYLVGLFEDTNLCAIHAKRVTIMPKDIQLARRIRGERA  239
            ALQEASEAYLVGLFEDTNLCAIHAKRVTIMPKDIQLARRIRGERA
```

```
Sbjct  241  ALQEASEAYLVGLFEDTNLCAIHAKRVTIMPKDIQLARRIRGERA  285
```

>LOW QUALITY PROTEIN: uncharacterized protein LOC100485665 [Xenopus tropicalis]
Sequence ID: XP_031749188.1 Length: 430
Range 1: 18 to 249

Score:415 bits(1066), Expect:4e-142,
Method:Compositional matrix adjust.,
Identities:214/232(92%), Positives:217/232(93%), Gaps:10/232(4%)

```
Query  18   RHRKVLRDNIQGITKPAIRRLARRGGVKRISGLIYEETRGVLKVFLENVIRDAVTYTEHA  77
            RHRKVLRDNIQGITKPAIRRLARRGGVKRISGLIYEETRGVLKVFLENVIRDAVTYTEHA
Sbjct  18   RHRKVLRDNIQGITKPAIRRLARRGGVKRISGLIYEETRGVLKVFLENVIRDAVTYTEHA  77

Query  78   KRKTVTAMDVVYALKRQGRTLYGF----GG------MARTKQTARKSTGGKAPRKQLATK  127
            KRKTVTAMDVVYALKRQGR L       GG      MARTKQTARKSTGGKAPRKQLATK
Sbjct  78   KRKTVTAMDVVYALKRQGRQLPTALEVRGGPSIXFIMARTKQTARKSTGGKAPRKQLATK  137

Query  128  AARKSAPSTGGVKKPHRYRPGTVALREIRRYQKSTELLIRKLPFQRLVREIAQDFKTDLR  187
            AARKSAP+TGGVKKPHRYRPGTVALREIRRYQKSTELLIRKLPFQRLVREIAQDFKTDLR
Sbjct  138  AARKSAPATGGVKKPHRYRPGTVALREIRRYQKSTELLIRKLPFQRLVREIAQDFKTDLR  197

Query  188  FQSAAIGALQEASEAYLVGLFEDTNLCAIHAKRVTIMPKDIQLARRIRGERA  239
            FQS+A+ ALQEASEAYLVGLFEDTNLCAIHAKRVTIMPKDIQLARRIRGERA
Sbjct  198  FQSSAVMALQEASEAYLVGLFEDTNLCAIHAKRVTIMPKDIQLARRIRGERA  249
```

Range 2: 328 to 430

Score:174 bits(440), Expect:7e-48,
Method:Compositional matrix adjust.,
Identities:103/103(100%), Positives:103/103(100%), Gaps:0/103(0%)

```
Query  1    MSGRGKGGKGLGKGGAKRHRKVLRDNIQGITKPAIRRLARRGGVKRISGLIYEETRGVLK  60
            MSGRGKGGKGLGKGGAKRHRKVLRDNIQGITKPAIRRLARRGGVKRISGLIYEETRGVLK
Sbjct  328  MSGRGKGGKGLGKGGAKRHRKVLRDNIQGITKPAIRRLARRGGVKRISGLIYEETRGVLK  387

Query  61   VFLENVIRDAVTYTEHAKRKTVTAMDVVYALKRQGRTLYGFGG  103
            VFLENVIRDAVTYTEHAKRKTVTAMDVVYALKRQGRTLYGFGG
Sbjct  388  VFLENVIRDAVTYTEHAKRKTVTAMDVVYALKRQGRTLYGFGG  430
```

>unnamed protein product [Anisakis simplex]
Sequence ID: VDK57329.1 Length: 411
Range 1: 133 to 411

Score:414 bits(1063), Expect:4e-142,

```
Method:Compositional matrix adjust.,
Identities:233/279(84%), Positives:236/279(84%), Gaps:40/279(14%)

Query  1    MSGRGKGGKGLGKGGAKRHRKVLRDNIQGITKPAIRRLARRGGVKRISGLIYEETRGVLK  60
            MSGRGKGGKGLGKGGAKRHRKVLRDNIQGITKPAIRRLARRGGVKRISGLIYEETRGVLK
Sbjct  133  MSGRGKGGKGLGKGGAKRHRKVLRDNIQGITKPAIRRLARRGGVKRISGLIYEETRGVLK  192

Query  61   VFLENVIRDAVTYTEHAKRKTVTAMDVVYALKRQGRTLYGFGG-----------------  103
            VFLENVIRDAVTY EHAKRKTVTAMDVVYALKRQGRTLYGFG
Sbjct  193  VFLENVIRDAVTYCEHAKRKTVTAMDVVYALKRQGRTLYGFGASQKGRQFSGRCFGEIAY  252

Query  104  -----------------------MARTKQTARKSTGGKAPRKQLATKAARKSAPSTGGVK  140
                                   MARTKQTARKSTGGKAPRKQLATKAARKSAP+TGGVK
Sbjct  253  KKASQAVATHRSISFDALVLAEIMARTKQTARKSTGGKAPRKQLATKAARKSAPATGGVK  312

Query  141  KPHRYRPGTVALREIRRYQKSTELLIRKLPFQRLVREIAQDFKTDLRFQSAAIGALQEAS  200
            KPHRYRPGTVALREIRRYQKSTELLIRKLPFQRLVREIAQDFKTDLRFQS+A+ ALQEAS
Sbjct  313  KPHRYRPGTVALREIRRYQKSTELLIRKLPFQRLVREIAQDFKTDLRFQSSAVLALQEAS  372

Query  201  EAYLVGLFEDTNLCAIHAKRVTIMPKDIQLARRIRGERA  239
            EAYLVGLFEDTNLCAIHAKRVTIMPKDIQLARRIRGERA
Sbjct  373  EAYLVGLFEDTNLCAIHAKRVTIMPKDIQLARRIRGERA  411
```

```
>uncharacterized protein [Physeter macrocephalus]
Sequence ID: XP_023977603.2 Length: 355
Range 1: 18 to 245

Score:411 bits(1056), Expect:8e-142,
Method:Compositional matrix adjust.,
Identities:211/228(93%), Positives:215/228(94%), Gaps:7/228(3%)

Query  18   RHRKVLRDNIQGITKPAIRRLARRGGVKRISGLIYEETRGVLKVFLENVIRDAVTYTEHA  77
            RHRKVLRDNIQGITKPAIRRLARRGGVKRISGLIYEETRGVLKVFLENVIRDAVTYTEHA
Sbjct  18   RHRKVLRDNIQGITKPAIRRLARRGGVKRISGLIYEETRGVLKVFLENVIRDAVTYTEHA  77

Query  78   KRKTVTAMDVVYALKRQGRT----LYGFGG---MARTKQTARKSTGGKAPRKQLATKAAR  130
            KRKTVTAMDVVYALKRQGR     L+  G    MARTKQTARKSTGGKAPRKQLATKAAR
Sbjct  78   KRKTVTAMDVVYALKRQGRRPATGLWVCGQSLVMARTKQTARKSTGGKAPRKQLATKAAR  137

Query  131  KSAPSTGGVKKPHRYRPGTVALREIRRYQKSTELLIRKLPFQRLVREIAQDFKTDLRFQS  190
            KSAP+TGGVKKPHRYRPGTVALREIRRYQKSTELLIRKLPFQRLVREIAQDFKTDLRFQS
Sbjct  138  KSAPATGGVKKPHRYRPGTVALREIRRYQKSTELLIRKLPFQRLVREIAQDFKTDLRFQS  197

Query  191  AAIGALQEASEAYLVGLFEDTNLCAIHAKRVTIMPKDIQLARRIRGER  238
            +A+ ALQEA EAYLVGLFEDTNLCAIHAKRVTIMPKDIQLARRIRGER
Sbjct  198  SAVMALQEACEAYLVGLFEDTNLCAIHAKRVTIMPKDIQLARRIRGER  245
```

Range 2: 253 to 355

Score:172 bits(436), Expect:4e-48,
Method:Compositional matrix adjust.,
Identities:103/103(100%), Positives:103/103(100%), Gaps:0/103(0%)

```
Query  1    MSGRGKGGKGLGKGGAKRHRKVLRDNIQGITKPAIRRLARRGGVKRISGLIYEETRGVLK  60
            MSGRGKGGKGLGKGGAKRHRKVLRDNIQGITKPAIRRLARRGGVKRISGLIYEETRGVLK
Sbjct  253  MSGRGKGGKGLGKGGAKRHRKVLRDNIQGITKPAIRRLARRGGVKRISGLIYEETRGVLK  312

Query  61   VFLENVIRDAVTYTEHAKRKTVTAMDVVYALKRQGRTLYGFGG  103
            VFLENVIRDAVTYTEHAKRKTVTAMDVVYALKRQGRTLYGFGG
Sbjct  313  VFLENVIRDAVTYTEHAKRKTVTAMDVVYALKRQGRTLYGFGG  355
```

>LOW QUALITY PROTEIN: uncharacterized protein LOC112404117 [Neophocaena asiaeorientalis asiaeorientalis]
Sequence ID: XP_024607716.1 Length: 469
Range 1: 194 to 469

Score:414 bits(1065), Expect:1e-141,
Method:Compositional matrix adjust.,
Identities:233/276(84%), Positives:236/276(85%), Gaps:37/276(13%)

```
Query  1    MSGRGKGGKGLGKGGAKRHRKVLRDNIQGITKPAIRRLARRGGVKRISGLIYEETRGVLK  60
            MSGRGKGGKGLGKGGAKRHRKVLRDNIQGITKPAIRRLARRGGVKRISGLIYEETRGVLK
Sbjct  194  MSGRGKGGKGLGKGGAKRHRKVLRDNIQGITKPAIRRLARRGGVKRISGLIYEETRGVLK  253

Query  61   VFLENVIRDAVTYTEHAKRKTVTAMDVVYALKRQGRTLYGFGG-----------------  103
            VFLENVIRDAVTYTEHAKRKTVTAMDVVYALKRQGRTLYGFG
Sbjct  254  VFLENVIRDAVTYTEHAKRKTVTAMDVVYALKRQGRTLYGFGETELHGHLALCWDSATPK  313

Query  104  --------------------MARTKQTARKSTGGKAPRKQLATKAARKSAPSTGGVKKPH  143
                                MARTKQTARKSTGGKAPRKQLATKAARKSAP+TGGVKKPH
Sbjct  314  VFSCCQYLQRKRGKKSPKXVMARTKQTARKSTGGKAPRKQLATKAARKSAPATGGVKKPH  373

Query  144  RYRPGTVALREIRRYQKSTELLIRKLPFQRLVREIAQDFKTDLRFQSAAIGALQEASEAY  203
            RYRPGTVALREIRRYQKSTELLIRKLPFQRLVREIAQDFKTDLRFQS+A+ ALQEA EAY
Sbjct  374  RYRPGTVALREIRRYQKSTELLIRKLPFQRLVREIAQDFKTDLRFQSSAVMALQEACEAY  433

Query  204  LVGLFEDTNLCAIHAKRVTIMPKDIQLARRIRGERA  239
            LVGLFEDTNLCAIHAKRVTIMPKDIQLARRIRGERA
Sbjct  434  LVGLFEDTNLCAIHAKRVTIMPKDIQLARRIRGERA  469
```

Range 2: 1 to 136

Score:259 bits(661), Expect:2e-80,
Method:Compositional matrix adjust.,
Identities:128/136(94%), Positives:131/136(96%), Gaps:0/136(0%)

```
Query  104  MARTKQTARKSTGGKAPRKQLATKAARKSAPSTGGVKKPHRYRPGTVALREIRRYQKSTE  163
            MARTKQTARKSTGGKAPRKQLATKAARKSAP+TGGVKKPHRYRPGTVALREIRRYQKSTE
Sbjct  1    MARTKQTARKSTGGKAPRKQLATKAARKSAPATGGVKKPHRYRPGTVALREIRRYQKSTE  60

Query  164  LLIRKLPFQRLVREIAQDFKTDLRFQSAAIGALQEASEAYLVGLFEDTNLCAIHAKRVTI  223
            LLIRKLPFQRLVREIAQ FK DLRFQS+A+ ALQEA E YLVGLFEDTNLCAIHAKRVTI
Sbjct  61   LLIRKLPFQRLVREIAQKFKIDLRFQSSAVMALQEACEGYLVGLFEDTNLCAIHAKRVTI  120

Query  224  MPKDIQLARRIRGERA  239
            MPKDIQLARRIRGERA
Sbjct  121  MPKDIQLARRIRGERA  136
```

>uncharacterized protein LOC132497238 [Mesoplodon densirostris]
Sequence ID: XP_059966288.1 Length: 470
Range 1: 18 to 246

Score:414 bits(1065), Expect:2e-141,
Method:Compositional matrix adjust.,
Identities:210/229(92%), Positives:215/229(93%), Gaps:7/229(3%)

```
Query  18   RHRKVLRDNIQGITKPAIRRLARRGGVKRISGLIYEETRGVLKVFLENVIRDAVTYTEHA  77
            RHRKVLRDNIQGITKPAIRRLARRGGVKRISGLIYEETRGVLKVFLENVIRDAVTYTEHA
Sbjct  18   RHRKVLRDNIQGITKPAIRRLARRGGVKRISGLIYEETRGVLKVFLENVIRDAVTYTEHA  77

Query  78   KRKTVTAMDVVYALKRQGR-------TLYGFGGMARTKQTARKSTGGKAPRKQLATKAAR  130
            KRKTVTAMDVVYALKRQG+        + G   MARTKQTARKSTGGKAPRKQLATKAAR
Sbjct  78   KRKTVTAMDVVYALKRQGQRALSARHLMSGLSRMARTKQTARKSTGGKAPRKQLATKAAR  137

Query  131  KSAPSTGGVKKPHRYRPGTVALREIRRYQKSTELLIRKLPFQRLVREIAQDFKTDLRFQS  190
            KSAP+TGGVKKPHRYRPGTVALREIRRYQKSTELLIRKLPFQRLVREIAQDFKTDLRFQS
Sbjct  138  KSAPATGGVKKPHRYRPGTVALREIRRYQKSTELLIRKLPFQRLVREIAQDFKTDLRFQS  197

Query  191  AAIGALQEASEAYLVGLFEDTNLCAIHAKRVTIMPKDIQLARRIRGERA  239
            +A+ ALQEA EAYLVGLFEDTNLCAIHAKRVTIMPKDIQLARRIRGERA
Sbjct  198  SAVMALQEACEAYLVGLFEDTNLCAIHAKRVTIMPKDIQLARRIRGERA  246
```

Range 2: 335 to 470

Score:266 bits(679), Expect:3e-83,
Method:Compositional matrix adjust.,
Identities:131/136(96%), Positives:134/136(98%), Gaps:0/136(0%)

```
Query  104  MARTKQTARKSTGGKAPRKQLATKAARKSAPSTGGVKKPHRYRPGTVALREIRRYQKSTE  163
            MARTKQTARKSTGGKAPRKQLATKAARKSAP+TGGVKKPHRYRPGTVALREIRRYQKSTE
Sbjct  335  MARTKQTARKSTGGKAPRKQLATKAARKSAPATGGVKKPHRYRPGTVALREIRRYQKSTE  394

Query  164  LLIRKLPFQRLVREIAQDFKTDLRFQSAAIGALQEASEAYLVGLFEDTNLCAIHAKRVTI  223
            LLIRKLPFQRLVREIAQDFKTDLRFQS+A+ ALQEA EAYLVGLFEDTNLCAIHAKRVTI
```

```
Sbjct  395  LLIRKLPFQRLVREIAQDFKTDLRFQSSAVMALQEACEAYLVGLFEDTNLCAIHAKRVTI  454

Query  224  MPKDIQLARRIRGERA  239
            MPKDIQLARRIRGERA
Sbjct  455  MPKDIQLARRIRGERA  470
```

>LOW QUALITY PROTEIN: uncharacterized protein LOC117997531 [Mirounga leonina]
Sequence ID: XP_034841793.1 Length: 310
Range 1: 1 to 231

Score:408 bits(1048), Expect:3e-141,
Method:Compositional matrix adjust.,
Identities:209/231(90%), Positives:212/231(91%), Gaps:17/231(7%)

```
Query  1    MSGRGKGGKGLGKGGAKRHRKVLRDNIQGITKPAIRRLARRGGVKRISGLIYEETRGVLK  60
            MSGRGKGGKGLGKGGAKRHRKVLRDNIQGITKPAIRRLARRGGVKRISGLIYEETRGVLK
Sbjct  1    MSGRGKGGKGLGKGGAKRHRKVLRDNIQGITKPAIRRLARRGGVKRISGLIYEETRGVLK  60

Query  61   VFLENVIRDAVTYTEHAKRKTVTAMDVVYALKRQGRTLYGFGG-----------------  103
            VFLENVIRDAVTYTEHAKRKTVTAMDVVYALKRQGRTLYGFGG
Sbjct  61   VFLENVIRDAVTYTEHAKRKTVTAMDVVYALKRQGRTLYGFGGXARRAPRSSHLNSGALE  120

Query  104  MARTKQTARKSTGGKAPRKQLATKAARKSAPSTGGVKKPHRYRPGTVALREIRRYQKSTE  163
            MARTKQTARKSTGGKAPRKQLATKAARKSAP+TGGVKKPHRYRPGTVALREIRRYQKSTE
Sbjct  121  MARTKQTARKSTGGKAPRKQLATKAARKSAPATGGVKKPHRYRPGTVALREIRRYQKSTE  180

Query  164  LLIRKLPFQRLVREIAQDFKTDLRFQSAAIGALQEASEAYLVGLFEDTNLC  214
            LLIRKLPFQRLVREIAQDFKTDLRFQS+A+ ALQEA EAYLVGLFEDTNLC
Sbjct  181  LLIRKLPFQRLVREIAQDFKTDLRFQSSAVMALQEACEAYLVGLFEDTNLC  231
```

Range 2: 237 to 310

Score:134 bits(337), Expect:8e-34,
Method:Compositional matrix adjust.,
Identities:74/102(73%), Positives:74/102(72%), Gaps:28/102(27%)

```
Query  2    SGRGKGGKGLGKGGAKRHRKVLRDNIQGITKPAIRRLARRGGVKRISGLIYEETRGVLKV  61
            SGRGKGGKGLGKGGAKR                            ISGLIYEETRGVLKV
Sbjct  237  SGRGKGGKGLGKGGAKR----------------------------ISGLIYEETRGVLKV  268

Query  62   FLENVIRDAVTYTEHAKRKTVTAMDVVYALKRQGRTLYGFGG  103
            FLENVIRDAVTYTEHAKRKTVTAMDVVYALKRQGRTLYGFGG
Sbjct  269  FLENVIRDAVTYTEHAKRKTVTAMDVVYALKRQGRTLYGFGG  310
```

>LOW QUALITY PROTEIN: uncharacterized protein LOC132923226 [Rhopalosiphum padi]

```
Sequence ID: XP_060843068.1 Length: 623
Range 1: 18 to 246

Score:419 bits(1076), Expect:5e-141,
Method:Compositional matrix adjust.,
Identities:212/229(93%), Positives:217/229(94%), Gaps:7/229(3%)

Query  18   RHRKVLRDNIQGITKPAIRRLARRGGVKRISGLIYEETRGVLKVFLENVIRDAVTYTEHA  77
            RHRKVLRDNIQGITKPAIRRLARRGGVKRISGLIYEETRGVLKVFLENVIRDAVTYTEHA
Sbjct  18   RHRKVLRDNIQGITKPAIRRLARRGGVKRISGLIYEETRGVLKVFLENVIRDAVTYTEHA  77

Query  78   KRKTVTAMDVVYALKRQGRT-LYGF------GGMARTKQTARKSTGGKAPRKQLATKAAR  130
            KRKTVTAMDVVYALKRQGRT +  F        MARTKQTARKSTGGKAPRKQLATKAAR
Sbjct  78   KRKTVTAMDVVYALKRQGRTFILQFVXHTTKSKMARTKQTARKSTGGKAPRKQLATKAAR  137

Query  131  KSAPSTGGVKKPHRYRPGTVALREIRRYQKSTELLIRKLPFQRLVREIAQDFKTDLRFQS  190
            KSAP+TGGVKKPHRYRPGTVALREIRRYQKSTELLIRKLPFQRLVREIAQDFKTDLRFQS
Sbjct  138  KSAPATGGVKKPHRYRPGTVALREIRRYQKSTELLIRKLPFQRLVREIAQDFKTDLRFQS  197

Query  191  AAIGALQEASEAYLVGLFEDTNLCAIHAKRVTIMPKDIQLARRIRGERA  239
            +A+ ALQEASEAYLVGLFEDTNLCAIHAKRVTIMPKDIQLARRIRGER+
Sbjct  198  SAVMALQEASEAYLVGLFEDTNLCAIHAKRVTIMPKDIQLARRIRGERS  246



Range 2: 531 to 623

Score:174 bits(441), Expect:1e-46,
Method:Compositional matrix adjust.,
Identities:93/93(100%), Positives:93/93(100%), Gaps:0/93(0%)

Query  11   LGKGGAKRHRKVLRDNIQGITKPAIRRLARRGGVKRISGLIYEETRGVLKVFLENVIRDA  70
            LGKGGAKRHRKVLRDNIQGITKPAIRRLARRGGVKRISGLIYEETRGVLKVFLENVIRDA
Sbjct  531  LGKGGAKRHRKVLRDNIQGITKPAIRRLARRGGVKRISGLIYEETRGVLKVFLENVIRDA  590

Query  71   VTYTEHAKRKTVTAMDVVYALKRQGRTLYGFGG  103
            VTYTEHAKRKTVTAMDVVYALKRQGRTLYGFGG
Sbjct  591  VTYTEHAKRKTVTAMDVVYALKRQGRTLYGFGG  623



>uncharacterized protein LOC122754920 [Dromiciops gliroides]
Sequence ID: XP_043859287.1 Length: 606
Range 1: 18 to 246

Score:417 bits(1073), Expect:8e-141,
Method:Compositional matrix adjust.,
Identities:213/229(93%), Positives:216/229(94%), Gaps:8/229(3%)

Query  18   RHRKVLRDNIQGITKPAIRRLARRGGVKRISGLIYEETRGVLKVFLENVIRDAVTYTEHA  77
            RHRKVLRDNIQGITKPAIRRLARRGGVKRISGLIYEETRGVLKVFLENVIRDAVTYTEHA
Sbjct  18   RHRKVLRDNIQGITKPAIRRLARRGGVKRISGLIYEETRGVLKVFLENVIRDAVTYTEHA  77
```

```
Query  78   KRKTVTAMDVVYALKRQGRT-LYG-------FGGMARTKQTARKSTGGKAPRKQLATKAA  129
            KRKTVTAMDVVYALKRQGR  LY        F  MARTKQTARKSTGGKAPRKQLATKAA
Sbjct  78   KRKTVTAMDVVYALKRQGRLRLYSSAVTRPLFFVMARTKQTARKSTGGKAPRKQLATKAA  137

Query  130  RKSAPSTGGVKKPHRYRPGTVALREIRRYQKSTELLIRKLPFQRLVREIAQDFKTDLRFQ  189
            RKSAP+TGGVKKPHRYRPGTVALREIRRYQKSTELLIRKLPFQRLVREIAQDFKTDLRFQ
Sbjct  138  RKSAPATGGVKKPHRYRPGTVALREIRRYQKSTELLIRKLPFQRLVREIAQDFKTDLRFQ  197

Query  190  SAAIGALQEASEAYLVGLFEDTNLCAIHAKRVTIMPKDIQLARRIRGER  238
            S+A+ ALQEASEAYLVGLFEDTNLCAIHAKRVTIMPKDIQLARRIRGER
Sbjct  198  SSAVMALQEASEAYLVGLFEDTNLCAIHAKRVTIMPKDIQLARRIRGER  246
```

Range 2: 470 to 606

Score:269 bits(687), Expect:8e-83,
Method:Compositional matrix adjust.,
Identities:132/137(96%), Positives:135/137(98%), Gaps:0/137(0%)

```
Query  103  GMARTKQTARKSTGGKAPRKQLATKAARKSAPSTGGVKKPHRYRPGTVALREIRRYQKST  162
             MARTKQTARKSTGGKAPRKQLATKAARKSAP+TGGVKKPHRYRPGTVALREIRRYQKST
Sbjct  470  AMARTKQTARKSTGGKAPRKQLATKAARKSAPATGGVKKPHRYRPGTVALREIRRYQKST  529

Query  163  ELLIRKLPFQRLVREIAQDFKTDLRFQSAAIGALQEASEAYLVGLFEDTNLCAIHAKRVT  222
            ELLIRKLPFQRLVREIAQDFKTDLRFQS+A+ ALQEASEAYLVGLFEDTNLCAIHAKRVT
Sbjct  530  ELLIRKLPFQRLVREIAQDFKTDLRFQSSAVMALQEASEAYLVGLFEDTNLCAIHAKRVT  589

Query  223  IMPKDIQLARRIRGERA  239
            IMPKDIQLARRIRGERA
Sbjct  590  IMPKDIQLARRIRGERA  606
```

>uncharacterized protein LOC131703110 [Acipenser ruthenus]
Sequence ID: XP_058861958.1 Length: 369
Range 1: 18 to 249

Score:408 bits(1049), Expect:2e-140,
Method:Compositional matrix adjust.,
Identities:211/232(91%), Positives:216/232(93%), Gaps:10/232(4%)

```
Query  18   RHRKVLRDNIQGITKPAIRRLARRGGVKRISGLIYEETRGVLKVFLENVIRDAVTYTEHA  77
            RHRKVLRDNIQGITKPAIRRLARRGGVKRISGLIYEETRGVLKVFLENVIRDAVTYTEHA
Sbjct  18   RHRKVLRDNIQGITKPAIRRLARRGGVKRISGLIYEETRGVLKVFLENVIRDAVTYTEHA  77

Query  78   KRKTVTAMDVVYALKRQ-----GRTLYGFG-----GMARTKQTARKSTGGKAPRKQLATK  127
            KRKTVTAMDVVYALKRQ     GR  +  G      MARTKQTARKSTGGKAPRKQLATK
Sbjct  78   KRKTVTAMDVVYALKRQAQQADGRRHHRSGRSASLKMARTKQTARKSTGGKAPRKQLATK  137

Query  128  AARKSAPSTGGVKKPHRYRPGTVALREIRRYQKSTELLIRKLPFQRLVREIAQDFKTDLR  187
```

```
           AARKSAP+TGGVKKPHRYRPGTVALREIRRYQKSTELLIRKLPFQRLVREIAQDFKTDLR
Sbjct  138  AARKSAPATGGVKKPHRYRPGTVALREIRRYQKSTELLIRKLPFQRLVREIAQDFKTDLR  197

Query  188  FQSAAIGALQEASEAYLVGLFEDTNLCAIHAKRVTIMPKDIQLARRIRGERA  239
            FQS+A+ ALQEASEAYLVGLFEDTNLCAIHAKRVTIMPKDIQLARRIRGER+
Sbjct  198  FQSSAVMALQEASEAYLVGLFEDTNLCAIHAKRVTIMPKDIQLARRIRGERS  249
```

Range 2: 267 to 369

Score:173 bits(438), Expect:3e-48,
Method:Compositional matrix adjust.,
Identities:103/103(100%), Positives:103/103(100%), Gaps:0/103(0%)

```
Query  1    MSGRGKGGKGLGKGGAKRHRKVLRDNIQGITKPAIRRLARRGGVKRISGLIYEETRGVLK  60
            MSGRGKGGKGLGKGGAKRHRKVLRDNIQGITKPAIRRLARRGGVKRISGLIYEETRGVLK
Sbjct  267  MSGRGKGGKGLGKGGAKRHRKVLRDNIQGITKPAIRRLARRGGVKRISGLIYEETRGVLK  326

Query  61   VFLENVIRDAVTYTEHAKRKTVTAMDVVYALKRQGRTLYGFGG  103
            VFLENVIRDAVTYTEHAKRKTVTAMDVVYALKRQGRTLYGFGG
Sbjct  327  VFLENVIRDAVTYTEHAKRKTVTAMDVVYALKRQGRTLYGFGG  369
```

>uncharacterized protein LOC125352382 [Perognathus longimembris pacificus]
Sequence ID: XP_048203429.1 Length: 562
Range 1: 18 to 246

Score:415 bits(1067), Expect:2e-140,
Method:Compositional matrix adjust.,
Identities:212/229(93%), Positives:215/229(93%), Gaps:7/229(3%)

```
Query  18   RHRKVLRDNIQGITKPAIRRLARRGGVKRISGLIYEETRGVLKVFLENVIRDAVTYTEHA  77
            RHRKVLRDNIQGITKPAIRRLARRGGVKRISGLIYEETRGVLKVFLENVIRDAVTYTEHA
Sbjct  18   RHRKVLRDNIQGITKPAIRRLARRGGVKRISGLIYEETRGVLKVFLENVIRDAVTYTEHA  77

Query  78   KRKTVTAMDVVYALKRQGR-------TLYGFGGMARTKQTARKSTGGKAPRKQLATKAAR  130
            KRKTVTAMDVVYALKRQGR        L     MARTKQTARKSTGGKAPRKQLATKAAR
Sbjct  78   KRKTVTAMDVVYALKRQGRKGLTSIPLLSSSRAMARTKQTARKSTGGKAPRKQLATKAAR  137

Query  131  KSAPSTGGVKKPHRYRPGTVALREIRRYQKSTELLIRKLPFQRLVREIAQDFKTDLRFQS  190
            KSAP+TGGVKKPHRYRPGTVALREIRRYQKSTELLIRKLPFQRLVREIAQDFKTDLRFQS
Sbjct  138  KSAPATGGVKKPHRYRPGTVALREIRRYQKSTELLIRKLPFQRLVREIAQDFKTDLRFQS  197

Query  191  AAIGALQEASEAYLVGLFEDTNLCAIHAKRVTIMPKDIQLARRIRGERA  239
            +A+ ALQEASEAYLVGLFEDTNLCAIHAKRVTIMPKDIQLARRIRGERA
Sbjct  198  SAVMALQEASEAYLVGLFEDTNLCAIHAKRVTIMPKDIQLARRIRGERA  246
```

Range 2: 460 to 562

Score:174 bits(440), Expect:6e-47,
Method:Compositional matrix adjust.,
Identities:103/103(100%), Positives:103/103(100%), Gaps:0/103(0%)

```
Query  1    MSGRGKGGKGLGKGGAKRHRKVLRDNIQGITKPAIRRLARRGGVKRISGLIYEETRGVLK  60
            MSGRGKGGKGLGKGGAKRHRKVLRDNIQGITKPAIRRLARRGGVKRISGLIYEETRGVLK
Sbjct  460  MSGRGKGGKGLGKGGAKRHRKVLRDNIQGITKPAIRRLARRGGVKRISGLIYEETRGVLK  519

Query  61   VFLENVIRDAVTYTEHAKRKTVTAMDVVYALKRQGRTLYGFGG  103
            VFLENVIRDAVTYTEHAKRKTVTAMDVVYALKRQGRTLYGFGG
Sbjct  520  VFLENVIRDAVTYTEHAKRKTVTAMDVVYALKRQGRTLYGFGG  562
```

>uncharacterized protein LOC115336205 [Aquila chrysaetos chrysaetos]
Sequence ID: XP_029858695.1 Length: 384
Range 1: 154 to 384

Score:409 bits(1050), Expect:2e-140,
Method:Compositional matrix adjust.,
Identities:212/231(92%), Positives:215/231(93%), Gaps:9/231(3%)

```
Query  18   RHRKVLRDNIQGITKPAIRRLARRGGVKRISGLIYEETRGVLKVFLENVIRDAVTYTEHA  77
            RHRKVLRDNIQGITKPAIRRLARRGGVKRISGLIYEETRGVLKVFLENVIRDAVTYTEHA
Sbjct  154  RHRKVLRDNIQGITKPAIRRLARRGGVKRISGLIYEETRGVLKVFLENVIRDAVTYTEHA  213

Query  78   KRKTVTAMDVVYALKRQG----RTLYGFGG-----MARTKQTARKSTGGKAPRKQLATKA  128
            KRKTVTAMDVVYALKRQG    R L          MARTKQTARKSTGGKAPRKQLATKA
Sbjct  214  KRKTVTAMDVVYALKRQGPFKRRALIAVRVRLGRVMARTKQTARKSTGGKAPRKQLATKA  273

Query  129  ARKSAPSTGGVKKPHRYRPGTVALREIRRYQKSTELLIRKLPFQRLVREIAQDFKTDLRF  188
            ARKSAP+TGGVKKPHRYRPGTVALREIRRYQKSTELLIRKLPFQRLVREIAQDFKTDLRF
Sbjct  274  ARKSAPATGGVKKPHRYRPGTVALREIRRYQKSTELLIRKLPFQRLVREIAQDFKTDLRF  333

Query  189  QSAAIGALQEASEAYLVGLFEDTNLCAIHAKRVTIMPKDIQLARRIRGERA  239
            QS+A+ ALQEASEAYLVGLFEDTNLCAIHAKRVTIMPKDIQLARRIRGERA
Sbjct  334  QSSAVMALQEASEAYLVGLFEDTNLCAIHAKRVTIMPKDIQLARRIRGERA  384
```

>PREDICTED: uncharacterized protein LOC105513924 [Colobus angolensis palliatus]
Sequence ID: XP_011800353.1 Length: 581
Range 1: 330 to 581

Score:415 bits(1066), Expect:5e-140,
Method:Compositional matrix adjust.,
Identities:215/252(85%), Positives:218/252(86%), Gaps:30/252(11%)

```
Query  18   RHRKVLRDNIQGITKPAIRRLARRGGVKRISGLIYEETRGVLKVFLENVIRDAVTYTEHA  77
            RHRKVLRDNIQGITKPAIRRLARRGGVKRISGLIYEETRGVLKVFLENVIRDAVTYTEHA
```

```
Sbjct  330   RHRKVLRDNIQGITKPAIRRLARRGGVKRISGLIYEETRGVLKVFLENVIRDAVTYTEHA  389

Query  78    KRKTVTAMDVVYALKRQGRTLYGF-----------GGM-------------------ART  107
             KRKTVTAMDVVYALKRQGRTLY             GGM                   ART
Sbjct  390   KRKTVTAMDVVYALKRQGRTLYAIKTILHFLTQSRGGMSPFHQQIKLGANSCAYLAMART  449

Query  108   KQTARKSTGGKAPRKQLATKAARKSAPSTGGVKKPHRYRPGTVALREIRRYQKSTELLIR  167
             KQTARKSTGGKAPRKQLATKAARKSAP+TGGVKKPHRYRPGTVALREIRRYQKSTELLIR
Sbjct  450   KQTARKSTGGKAPRKQLATKAARKSAPATGGVKKPHRYRPGTVALREIRRYQKSTELLIR  509

Query  168   KLPFQRLVREIAQDFKTDLRFQSAAIGALQEASEAYLVGLFEDTNLCAIHAKRVTIMPKD  227
             KLPFQRLVREIAQDFKTDLRFQS+A+ ALQEA EAYLVGLFEDTNLCAIHAKRVTIMPKD
Sbjct  510   KLPFQRLVREIAQDFKTDLRFQSSAVMALQEACEAYLVGLFEDTNLCAIHAKRVTIMPKD  569

Query  228   IQLARRIRGERA  239
             IQLARRIRGERA
Sbjct  570   IQLARRIRGERA  581
```

Range 2: 1 to 135

Score:265 bits(676), Expect:2e-81,
Method:Compositional matrix adjust.,
Identities:130/135(96%), Positives:133/135(98%), Gaps:0/135(0%)

```
Query  104   MARTKQTARKSTGGKAPRKQLATKAARKSAPSTGGVKKPHRYRPGTVALREIRRYQKSTE  163
             MARTKQTARKSTGGKAPRKQLATKAARKSAP+TGGVKKPHRYRPGTVALREIRRYQKSTE
Sbjct  1     MARTKQTARKSTGGKAPRKQLATKAARKSAPATGGVKKPHRYRPGTVALREIRRYQKSTE  60

Query  164   LLIRKLPFQRLVREIAQDFKTDLRFQSAAIGALQEASEAYLVGLFEDTNLCAIHAKRVTI  223
             LLIRKLPFQRLVREIAQDFKTDLRFQS+A+ ALQEA EAYLVGLFEDTNLCAIHAKRVTI
Sbjct  61    LLIRKLPFQRLVREIAQDFKTDLRFQSSAVMALQEACEAYLVGLFEDTNLCAIHAKRVTI  120

Query  224   MPKDIQLARRIRGER  238
             MPKDIQLARRIRGER
Sbjct  121   MPKDIQLARRIRGER  135
```

>uncharacterized protein LOC100442896 isoform X2 [Pongo abelii]
Sequence ID: XP_002816573.3 Length: 406
Range 1: 78 to 300

Score:408 bits(1048), Expect:7e-140,
Method:Compositional matrix adjust.,
Identities:207/223(93%), Positives:211/223(94%), Gaps:7/223(3%)

```
Query  1     MSGRGKGGKGLGKGGAKRHRKVLRDNIQGITKPAIRRLARRGGVKRISGLIYEETRGVLK  60
             MSGRGKGGKGLGKGGAKRHRKVLRDNIQGITKPAIRRLARRGGVKRISGLIYEETRGVLK
Sbjct  78    MSGRGKGGKGLGKGGAKRHRKVLRDNIQGITKPAIRRLARRGGVKRISGLIYEETRGVLK  137
```

```
Query  61   VFLENVIRDAVTYTEHAKRKTVTAMDVVYALKRQGRTLYGF-------GGMARTKQTARK  113
            VFLENVIRDAVTYTEHAKRKTVTAMDVVYALKRQGRTLYGF         MARTKQTARK
Sbjct  138  VFLENVIRDAVTYTEHAKRKTVTAMDVVYALKRQGRTLYGFDHMRSGRSSMARTKQTARK  197

Query  114  STGGKAPRKQLATKAARKSAPSTGGVKKPHRYRPGTVALREIRRYQKSTELLIRKLPFQR  173
            STGGKAPRKQLATKAARKSAP+TGGVKKPHRYRPGTVALREIRRYQKSTELLIRKLPFQR
Sbjct  198  STGGKAPRKQLATKAARKSAPATGGVKKPHRYRPGTVALREIRRYQKSTELLIRKLPFQR  257

Query  174  LVREIAQDFKTDLRFQSAAIGALQEASEAYLVGLFEDTNLCAI  216
            LVREIAQDFKTDLRFQS+A+ ALQEA EAYLVGLFEDTNLC +
Sbjct  258  LVREIAQDFKTDLRFQSSAVMALQEACEAYLVGLFEDTNLCCL  300
```

Range 2: 304 to 406

Score:206 bits(523), Expect:2e-60,
Method:Compositional matrix adjust.,
Identities:103/103(100%), Positives:103/103(100%), Gaps:0/103(0%)

```
Query  1    MSGRGKGGKGLGKGGAKRHRKVLRDNIQGITKPAIRRLARRGGVKRISGLIYEETRGVLK  60
            MSGRGKGGKGLGKGGAKRHRKVLRDNIQGITKPAIRRLARRGGVKRISGLIYEETRGVLK
Sbjct  304  MSGRGKGGKGLGKGGAKRHRKVLRDNIQGITKPAIRRLARRGGVKRISGLIYEETRGVLK  363

Query  61   VFLENVIRDAVTYTEHAKRKTVTAMDVVYALKRQGRTLYGFGG  103
            VFLENVIRDAVTYTEHAKRKTVTAMDVVYALKRQGRTLYGFGG
Sbjct  364  VFLENVIRDAVTYTEHAKRKTVTAMDVVYALKRQGRTLYGFGG  406
```

>hypothetical protein E2320_003247 [Naja naja]
Sequence ID: KAG8140557.1 Length: 369
Range 1: 127 to 369

Score:406 bits(1043), Expect:1e-139,
Method:Compositional matrix adjust.,
Identities:211/243(87%), Positives:214/243(88%), Gaps:21/243(8%)

```
Query  18   RHRKVLRDNIQGITKPAIRRLARRGGVKRISGLIYEETRGVLKVFLENVIRDAVTYTEHA  77
            RHRKVLRDNIQGITKPAIRRLARRGGVKRISGLIYEETRGVLKVFLENVIRDAVTYTEHA
Sbjct  127  RHRKVLRDNIQGITKPAIRRLARRGGVKRISGLIYEETRGVLKVFLENVIRDAVTYTEHA  186

Query  78   KRKTVTAMDVVYALKRQGRTL---------------------YGFGGMARTKQTARKSTG  116
            KRKTVTAMDVVYALKRQ  TL                          MARTKQTARKSTG
Sbjct  187  KRKTVTAMDVVYALKRQDGTLPIKYPPARQPNSFTRTFRQRNCLVWKMARTKQTARKSTG  246

Query  117  GKAPRKQLATKAARKSAPSTGGVKKPHRYRPGTVALREIRRYQKSTELLIRKLPFQRLVR  176
            GKAPRKQLATKAARKSAP+TGGVKKPHRYRPGTVALREIRRYQKSTELLIRKLPFQRLVR
Sbjct  247  GKAPRKQLATKAARKSAPATGGVKKPHRYRPGTVALREIRRYQKSTELLIRKLPFQRLVR  306

Query  177  EIAQDFKTDLRFQSAAIGALQEASEAYLVGLFEDTNLCAIHAKRVTIMPKDIQLARRIRG  236
            EIAQDFKTDLRFQS+A+ ALQEASEAYLVGLFEDTNLCAIHAKRVTIMPKDIQLARRIRG
```

```
Sbjct  307  EIAQDFKTDLRFQSSAVMALQEASEAYLVGLFEDTNLCAIHAKRVTIMPKDIQLARRIRG  366

Query  237  ERA  239
            ERA
Sbjct  367  ERA  369
```

Range 2: 1 to 103

Score:173 bits(439), Expect:2e-48,
Method:Compositional matrix adjust.,
Identities:103/103(100%), Positives:103/103(100%), Gaps:0/103(0%)

```
Query  1    MSGRGKGGKGLGKGGAKRHRKVLRDNIQGITKPAIRRLARRGGVKRISGLIYEETRGVLK  60
            MSGRGKGGKGLGKGGAKRHRKVLRDNIQGITKPAIRRLARRGGVKRISGLIYEETRGVLK
Sbjct  1    MSGRGKGGKGLGKGGAKRHRKVLRDNIQGITKPAIRRLARRGGVKRISGLIYEETRGVLK  60

Query  61   VFLENVIRDAVTYTEHAKRKTVTAMDVVYALKRQGRTLYGFGG  103
            VFLENVIRDAVTYTEHAKRKTVTAMDVVYALKRQGRTLYGFGG
Sbjct  61   VFLENVIRDAVTYTEHAKRKTVTAMDVVYALKRQGRTLYGFGG  103
```

>uncharacterized protein LOC130830811 [Hippopotamus amphibius kiboko]
Sequence ID: XP_057554119.1 Length: 738
Range 1: 1 to 272

Score:418 bits(1074), Expect:2e-139,
Method:Compositional matrix adjust.,
Identities:234/272(86%), Positives:237/272(87%), Gaps:33/272(12%)

```
Query  1    MSGRGKGGKGLGKGGAKRHRKVLRDNIQGITKPAIRRLARRGGVKRISGLIYEETRGVLK  60
            MSGRGKGGKGLGKGGAKRHRKVLRDNIQGITKPAIRRLARRGGVKRISGLIYEETRGVLK
Sbjct  1    MSGRGKGGKGLGKGGAKRHRKVLRDNIQGITKPAIRRLARRGGVKRISGLIYEETRGVLK  60

Query  61   VFLENVIRDAVTYTEHAKRKTVTAMDVVYALKRQGRTLYGFG------------------  102
            VFLENVIRDAVTYTEHAKRKTVTAMDVVYALKRQGRTLYGFG
Sbjct  61   VFLENVIRDAVTYTEHAKRKTVTAMDVVYALKRQGRTLYGFGGLTGIAHNPIRVSLFLYK  120

Query  103  ---------------GMARTKQTARKSTGGKAPRKQLATKAARKSAPSTGGVKKPHRYRP  147
                           GMARTKQTARKSTGGKAPRKQLATKAARKSAP+TGGVKKPHRYRP
Sbjct  121  YKSNASCIVFLFWKQGMARTKQTARKSTGGKAPRKQLATKAARKSAPATGGVKKPHRYRP  180

Query  148  GTVALREIRRYQKSTELLIRKLPFQRLVREIAQDFKTDLRFQSAAIGALQEASEAYLVGL  207
            GTVALREIRRYQKSTELLIRKLPFQRLVREIAQDFKTDLRFQS+A+ ALQEA EAYLVGL
Sbjct  181  GTVALREIRRYQKSTELLIRKLPFQRLVREIAQDFKTDLRFQSSAVMALQEACEAYLVGL  240

Query  208  FEDTNLCAIHAKRVTIMPKDIQLARRIRGERA  239
            FEDTNLCAIHAKRVTIMPKDIQLARRIRGERA
Sbjct  241  FEDTNLCAIHAKRVTIMPKDIQLARRIRGERA  272
```

```
Range 2: 636 to 738

Score:208 bits(530), Expect:8e-59,
Method:Compositional matrix adjust.,
Identities:103/103(100%), Positives:103/103(100%), Gaps:0/103(0%)

Query  1    MSGRGKGGKGLGKGGAKRHRKVLRDNIQGITKPAIRRLARRGGVKRISGLIYEETRGVLK  60
            MSGRGKGGKGLGKGGAKRHRKVLRDNIQGITKPAIRRLARRGGVKRISGLIYEETRGVLK
Sbjct  636  MSGRGKGGKGLGKGGAKRHRKVLRDNIQGITKPAIRRLARRGGVKRISGLIYEETRGVLK  695

Query  61   VFLENVIRDAVTYTEHAKRKTVTAMDVVYALKRQGRTLYGFGG  103
            VFLENVIRDAVTYTEHAKRKTVTAMDVVYALKRQGRTLYGFGG
Sbjct  696  VFLENVIRDAVTYTEHAKRKTVTAMDVVYALKRQGRTLYGFGG  738



>uncharacterized protein LOC129543310 [Moschus berezovskii]
Sequence ID: XP_055262377.1 Length: 418
Range 1: 177 to 418

Score:407 bits(1046), Expect:2e-139,
Method:Compositional matrix adjust.,
Identities:210/242(87%), Positives:214/242(88%), Gaps:20/242(8%)

Query  18   RHRKVLRDNIQGITKPAIRRLARRGGVKRISGLIYEETRGVLKVFLENVIRDAVTYTEHA  77
            RHRKVLRDNIQGITKPAIRRLARRGGVKRISGLIYEETRGVLKVFLENVIRDAVTYTEHA
Sbjct  177  RHRKVLRDNIQGITKPAIRRLARRGGVKRISGLIYEETRGVLKVFLENVIRDAVTYTEHA  236

Query  78   KRKTVTAMDVVYALKRQGRT-------LYGFG-------------GMARTKQTARKSTGG  117
            KRKTVTAMDVVYALKR           LY +G              MARTKQTARKSTGG
Sbjct  237  KRKTVTAMDVVYALKRIAHNPIRVILVLYKYGSKPPSLIPLSCRIAMARTKQTARKSTGG  296

Query  118  KAPRKQLATKAARKSAPSTGGVKKPHRYRPGTVALREIRRYQKSTELLIRKLPFQRLVRE  177
            KAPRKQLATKAARKSAP+TGGVKKPHRYRPGTVALREIRRYQKSTELLIRKLPFQRLVRE
Sbjct  297  KAPRKQLATKAARKSAPATGGVKKPHRYRPGTVALREIRRYQKSTELLIRKLPFQRLVRE  356

Query  178  IAQDFKTDLRFQSAAIGALQEASEAYLVGLFEDTNLCAIHAKRVTIMPKDIQLARRIRGE  237
            IAQDFKTDLRFQS+A+ ALQEA EAYLVGLFEDTNLCAIHAKRVTIMPKDIQLARRIRGE
Sbjct  357  IAQDFKTDLRFQSSAVMALQEACEAYLVGLFEDTNLCAIHAKRVTIMPKDIQLARRIRGE  416

Query  238  RA  239
            RA
Sbjct  417  RA  418



Range 2: 1 to 136

Score:266 bits(680), Expect:6e-84,
```

Method:Compositional matrix adjust.,
Identities:131/136(96%), Positives:134/136(98%), Gaps:0/136(0%)

```
Query  104  MARTKQTARKSTGGKAPRKQLATKAARKSAPSTGGVKKPHRYRPGTVALREIRRYQKSTE  163
            MARTKQTARKSTGGKAPRKQLATKAARKSAP+TGGVKKPHRYRPGTVALREIRRYQKSTE
Sbjct  1    MARTKQTARKSTGGKAPRKQLATKAARKSAPATGGVKKPHRYRPGTVALREIRRYQKSTE  60

Query  164  LLIRKLPFQRLVREIAQDFKTDLRFQSAAIGALQEASEAYLVGLFEDTNLCAIHAKRVTI  223
            LLIRKLPFQRLVREIAQDFKTDLRFQS+A+ ALQEA EAYLVGLFEDTNLCAIHAKRVTI
Sbjct  61   LLIRKLPFQRLVREIAQDFKTDLRFQSSAVMALQEACEAYLVGLFEDTNLCAIHAKRVTI  120

Query  224  MPKDIQLARRIRGERA  239
            MPKDIQLARRIRGERA
Sbjct  121  MPKDIQLARRIRGERA  136
```

>uncharacterized protein LOC101170117 [Oryzias latipes]
Sequence ID: XP_023818559.1 Length: 329
Range 1: 104 to 329

Score:402 bits(1033), Expect:9e-139,
Method:Compositional matrix adjust.,
Identities:206/226(91%), Positives:209/226(92%), Gaps:15/226(6%)

```
Query  29   GITKPAIRRLARRGGVKRISGLIYEETRGVLKVFLENVIRDAVTYTEHAKRKTVTAMDVV  88
            GITKPAIRRLARRGGVKRISGLIYEETRGVLKVFLENVIRDAVTYTEHAKRKTVTAMDVV
Sbjct  104  GITKPAIRRLARRGGVKRISGLIYEETRGVLKVFLENVIRDAVTYTEHAKRKTVTAMDVV  163

Query  89   YALKRQGRTLYGFG---------------GMARTKQTARKSTGGKAPRKQLATKAARKSA  133
            YALKRQGRTLYGFG                MARTKQTARKSTGGKAPRKQLATKAARKSA
Sbjct  164  YALKRQGRTLYGFGESFVLRFRTTLACPPTMARTKQTARKSTGGKAPRKQLATKAARKSA  223

Query  134  PSTGGVKKPHRYRPGTVALREIRRYQKSTELLIRKLPFQRLVREIAQDFKTDLRFQSAAI  193
            P+TGGVKKPHRYRPGTVALREIRRYQKSTELLIRKLPFQRLVREIAQDFKTDLRFQS+A+
Sbjct  224  PATGGVKKPHRYRPGTVALREIRRYQKSTELLIRKLPFQRLVREIAQDFKTDLRFQSSAV  283

Query  194  GALQEASEAYLVGLFEDTNLCAIHAKRVTIMPKDIQLARRIRGERA  239
             ALQEASEAYLVGLFEDTNLCAIHAKRVTIMPKDIQLARRIRGERA
Sbjct  284  MALQEASEAYLVGLFEDTNLCAIHAKRVTIMPKDIQLARRIRGERA  329
```

Range 2: 1 to 103

Score:172 bits(436), Expect:2e-48,
Method:Compositional matrix adjust.,
Identities:103/103(100%), Positives:103/103(100%), Gaps:0/103(0%)

```
Query  1    MSGRGKGGKGLGKGGAKRHRKVLRDNIQGITKPAIRRLARRGGVKRISGLIYEETRGVLK  60
            MSGRGKGGKGLGKGGAKRHRKVLRDNIQGITKPAIRRLARRGGVKRISGLIYEETRGVLK
Sbjct  1    MSGRGKGGKGLGKGGAKRHRKVLRDNIQGITKPAIRRLARRGGVKRISGLIYEETRGVLK  60
```

```
Query  61   VFLENVIRDAVTYTEHAKRKTVTAMDVVYALKRQGRTLYGFGG  103
            VFLENVIRDAVTYTEHAKRKTVTAMDVVYALKRQGRTLYGFGG
Sbjct  61   VFLENVIRDAVTYTEHAKRKTVTAMDVVYALKRQGRTLYGFGG  103
```

>uncharacterized protein LOC119435142 [Dermacentor silvarum]
Sequence ID: XP_037558005.2 Length: 371
Range 1: 45 to 265

Score:403 bits(1036), Expect:2e-138,
Method:Compositional matrix adjust.,
Identities:206/221(93%), Positives:210/221(95%), Gaps:6/221(2%)

```
Query  1    MSGRGKGGKGLGKGGAKRHRKVLRDNIQGITKPAIRRLARRGGVKRISGLIYEETRGVLK  60
            MSGRGKGGKGLGKGGAKRHRKVLRDNIQGITKPAIRRLARRGGVKRISGLIYEETRGVLK
Sbjct  45   MSGRGKGGKGLGKGGAKRHRKVLRDNIQGITKPAIRRLARRGGVKRISGLIYEETRGVLK  104

Query  61   VFLENVIRDAVTYTEHAKRKTVTAMDVVYALKRQGRTLYGF------GGMARTKQTARKS  114
            VFLENVIRDAVTYTEHAKRKTVTAMDVVYALKRQGRTLY +        MARTKQTARKS
Sbjct  105  VFLENVIRDAVTYTEHAKRKTVTAMDVVYALKRQGRTLYVWTLPSPSSAMARTKQTARKS  164

Query  115  TGGKAPRKQLATKAARKSAPSTGGVKKPHRYRPGTVALREIRRYQKSTELLIRKLPFQRL  174
            TGGKAPRKQLATKAARKSAP+TGGVKKPHRYRPGTVALREIRRYQKSTELLIRKLPFQRL
Sbjct  165  TGGKAPRKQLATKAARKSAPATGGVKKPHRYRPGTVALREIRRYQKSTELLIRKLPFQRL  224

Query  175  VREIAQDFKTDLRFQSAAIGALQEASEAYLVGLFEDTNLCA  215
            VREIAQDFKTDLRFQS+A+ ALQEASEAYLVGLFEDTNL A
Sbjct  225  VREIAQDFKTDLRFQSSAVMALQEASEAYLVGLFEDTNLSA  265
```

Range 2: 269 to 371

Score:207 bits(527), Expect:1e-61,
Method:Compositional matrix adjust.,
Identities:103/103(100%), Positives:103/103(100%), Gaps:0/103(0%)

```
Query  1    MSGRGKGGKGLGKGGAKRHRKVLRDNIQGITKPAIRRLARRGGVKRISGLIYEETRGVLK  60
            MSGRGKGGKGLGKGGAKRHRKVLRDNIQGITKPAIRRLARRGGVKRISGLIYEETRGVLK
Sbjct  269  MSGRGKGGKGLGKGGAKRHRKVLRDNIQGITKPAIRRLARRGGVKRISGLIYEETRGVLK  328

Query  61   VFLENVIRDAVTYTEHAKRKTVTAMDVVYALKRQGRTLYGFGG  103
            VFLENVIRDAVTYTEHAKRKTVTAMDVVYALKRQGRTLYGFGG
Sbjct  329  VFLENVIRDAVTYTEHAKRKTVTAMDVVYALKRQGRTLYGFGG  371
```

>uncharacterized protein LOC130348372 [Hyla sarda]
Sequence ID: XP_056410747.1 Length: 445

```
Range 1: 18 to 241

Score:405 bits(1041), Expect:3e-138,
Method:Compositional matrix adjust.,
Identities:206/224(92%), Positives:212/224(94%), Gaps:7/224(3%)

Query  18   RHRKVLRDNIQGITKPAIRRLARRGGVKRISGLIYEETRGVLKVFLENVIRDAVTYTEHA  77
            RHRKVLRDNIQGITKPAIRRLARRGGVKRISGLIYEETRGVLKVFLENVIRDAVTYTEHA
Sbjct  18   RHRKVLRDNIQGITKPAIRRLARRGGVKRISGLIYEETRGVLKVFLENVIRDAVTYTEHA  77

Query  78   KRKTVTAMDVVYALKRQGR----TLYGFGG---MARTKQTARKSTGGKAPRKQLATKAAR  130
            KRKTVTAMDVVYALKRQG+     ++   G   MARTKQTARKSTGGKAPRKQLATKAAR
Sbjct  78   KRKTVTAMDVVYALKRQGQRASALMFLSAGRKIMARTKQTARKSTGGKAPRKQLATKAAR  137

Query  131  KSAPSTGGVKKPHRYRPGTVALREIRRYQKSTELLIRKLPFQRLVREIAQDFKTDLRFQS  190
            KSAP+TGGVKKPHRYRPGTVALREIRRYQKSTELLIRKLPFQRLVREIAQDFKTDLRFQS
Sbjct  138  KSAPATGGVKKPHRYRPGTVALREIRRYQKSTELLIRKLPFQRLVREIAQDFKTDLRFQS  197

Query  191  AAIGALQEASEAYLVGLFEDTNLCAIHAKRVTIMPKDIQLARRI  234
            +A+ ALQEASEAYLVGLFEDTNLCAIHAKRVTIMPKDIQLARRI
Sbjct  198  SAVMALQEASEAYLVGLFEDTNLCAIHAKRVTIMPKDIQLARRI  241


Range 2: 343 to 445

Score:174 bits(440), Expect:8e-48,
Method:Compositional matrix adjust.,
Identities:103/103(100%), Positives:103/103(100%), Gaps:0/103(0%)

Query  1    MSGRGKGGKGLGKGGAKRHRKVLRDNIQGITKPAIRRLARRGGVKRISGLIYEETRGVLK  60
            MSGRGKGGKGLGKGGAKRHRKVLRDNIQGITKPAIRRLARRGGVKRISGLIYEETRGVLK
Sbjct  343  MSGRGKGGKGLGKGGAKRHRKVLRDNIQGITKPAIRRLARRGGVKRISGLIYEETRGVLK  402

Query  61   VFLENVIRDAVTYTEHAKRKTVTAMDVVYALKRQGRTLYGFGG  103
            VFLENVIRDAVTYTEHAKRKTVTAMDVVYALKRQGRTLYGFGG
Sbjct  403  VFLENVIRDAVTYTEHAKRKTVTAMDVVYALKRQGRTLYGFGG  445


>uncharacterized protein LOC124227097 [Equus quagga]
Sequence ID: XP_046496952.1 Length: 606
Range 1: 18 to 246

Score:409 bits(1051), Expect:2e-137,
Method:Compositional matrix adjust.,
Identities:209/229(91%), Positives:212/229(92%), Gaps:8/229(3%)

Query  18   RHRKVLRDNIQGITKPAIRRLARRGGVKRISGLIYEETRGVLKVFLENVIRDAVTYTEHA  77
            RHRKVLRDNIQGITKPAIRRLARRGGVKRISGLIYEETRGVLKVFLENVIRDAVTYTEHA
Sbjct  18   RHRKVLRDNIQGITKPAIRRLARRGGVKRISGLIYEETRGVLKVFLENVIRDAVTYTEHA  77
```

```
Query  78   KRKTVTAMDVVYALKRQGRTLYG--------FGGMARTKQTARKSTGGKAPRKQLATKAA  129
            KRKTVTAMDVVYALKRQGR               MARTKQTARKSTGGKAPRKQLATKAA
Sbjct  78   KRKTVTAMDVVYALKRQGRLSPSVLTCVPSRLSEMARTKQTARKSTGGKAPRKQLATKAA  137

Query  130  RKSAPSTGGVKKPHRYRPGTVALREIRRYQKSTELLIRKLPFQRLVREIAQDFKTDLRFQ  189
            RKSAP+TGGVKKPHRYRPGTVALREIRRYQKSTELLIRKLPFQRLVREIAQDFKTDLRFQ
Sbjct  138  RKSAPATGGVKKPHRYRPGTVALREIRRYQKSTELLIRKLPFQRLVREIAQDFKTDLRFQ  197

Query  190  SAAIGALQEASEAYLVGLFEDTNLCAIHAKRVTIMPKDIQLARRIRGER  238
            S+A+ ALQEA EAYLVGLFEDTNLCAIHAKRVTIMPKDIQLARRIRGER
Sbjct  198  SSAVMALQEACEAYLVGLFEDTNLCAIHAKRVTIMPKDIQLARRIRGER  246
```

Range 2: 316 to 497

Score:266 bits(681), Expect:6e-82,
Method:Compositional matrix adjust.,
Identities:139/189(74%), Positives:154/189(81%), Gaps:7/189(3%)

```
Query  50   LIYEETRGVLKVFLENVIRDAVTYTEHAKRKTVTAMDVVYALKRQGRTLYGFGGMARTKQ  109
            ++  + R + +  LE+V+ + V       R     +D++       + L  F  MARTKQ
Sbjct  316  VVMRQARRLARDALEDVVDERVHDAHGLGRDAGVGVDLL-------QHLVHFLTMARTKQ  368

Query  110  TARKSTGGKAPRKQLATKAARKSAPSTGGVKKPHRYRPGTVALREIRRYQKSTELLIRKL  169
            TARKSTGGKAPRKQLATKAARKSAP+TGGVKKPHRYRPGTVALREIRRYQKSTELLIRKL
Sbjct  369  TARKSTGGKAPRKQLATKAARKSAPATGGVKKPHRYRPGTVALREIRRYQKSTELLIRKL  428

Query  170  PFQRLVREIAQDFKTDLRFQSAAIGALQEASEAYLVGLFEDTNLCAIHAKRVTIMPKDIQ  229
            PFQRLVREIAQDFKTDLRFQS+A+ ALQEA EAYLVGLFEDTNLCAIHAKRVTIMPKDIQ
Sbjct  429  PFQRLVREIAQDFKTDLRFQSSAVMALQEACEAYLVGLFEDTNLCAIHAKRVTIMPKDIQ  488

Query  230  LARRIRGER  238
            LARRIRGER
Sbjct  489  LARRIRGER  497
```

Range 3: 504 to 606

Score:172 bits(437), Expect:4e-46,
Method:Compositional matrix adjust.,
Identities:103/103(100%), Positives:103/103(100%), Gaps:0/103(0%)

```
Query  1    MSGRGKGGKGLGKGGAKRHRKVLRDNIQGITKPAIRRLARRGGVKRISGLIYEETRGVLK  60
            MSGRGKGGKGLGKGGAKRHRKVLRDNIQGITKPAIRRLARRGGVKRISGLIYEETRGVLK
Sbjct  504  MSGRGKGGKGLGKGGAKRHRKVLRDNIQGITKPAIRRLARRGGVKRISGLIYEETRGVLK  563

Query  61   VFLENVIRDAVTYTEHAKRKTVTAMDVVYALKRQGRTLYGFGG  103
            VFLENVIRDAVTYTEHAKRKTVTAMDVVYALKRQGRTLYGFGG
Sbjct  564  VFLENVIRDAVTYTEHAKRKTVTAMDVVYALKRQGRTLYGFGG  606
```

>LOW QUALITY PROTEIN: uncharacterized protein LOC109913959 [Rhincodon typus]
Sequence ID: XP_048448253.1 Length: 476
Range 1: 260 to 476

Score:404 bits(1039), Expect:2e-137,
Method:Compositional matrix adjust.,
Identities:204/224(91%), Positives:211/224(94%), Gaps:7/224(3%)

```
Query  16   AKRHRKVLRDNIQGITKPAIRRLARRGGVKRISGLIYEETRGVLKVFLENVIRDAVTYTE  75
            AKRHRKVLRDNIQGITKPAIRRLARRGGVKRISGLIYEETRGVLKVFLENVIRDAVTYTE
Sbjct  260  AKRHRKVLRDNIQGITKPAIRRLARRGGVKRISGLIYEETRGVLKVFLENVIRDAVTYTE  319

Query  76   HAKRKTVTAMDVVYALKRQGRTLYGFGGMARTKQTARKSTGGKAPRKQLATKAARKSAPS  135
            HAKRKTVTAMDV        +++    GMARTKQTARKSTGGKAPRKQLATKAARKSAP+
Sbjct  320  HAKRKTVTAMDV-------QQSVCVLQGMARTKQTARKSTGGKAPRKQLATKAARKSAPA  372

Query  136  TGGVKKPHRYRPGTVALREIRRYQKSTELLIRKLPFQRLVREIAQDFKTDLRFQSAAIGA  195
            TGGVKKPHRYRPGTVALREIRRYQKSTELLIRKLPFQRLVREIAQDFKTDLRFQ++A+ A
Sbjct  373  TGGVKKPHRYRPGTVALREIRRYQKSTELLIRKLPFQRLVREIAQDFKTDLRFQNSAVMA  432

Query  196  LQEASEAYLVGLFEDTNLCAIHAKRVTIMPKDIQLARRIRGERA  239
            LQEASEAYLVGLFEDTNLCAIHAKRVTIMPKDIQLARRIRGERA
Sbjct  433  LQEASEAYLVGLFEDTNLCAIHAKRVTIMPKDIQLARRIRGERA  476
```

Range 2: 1 to 135

Score:265 bits(677), Expect:9e-83,
Method:Compositional matrix adjust.,
Identities:130/135(96%), Positives:133/135(98%), Gaps:0/135(0%)

```
Query  104  MARTKQTARKSTGGKAPRKQLATKAARKSAPSTGGVKKPHRYRPGTVALREIRRYQKSTE  163
            MARTKQTARKSTGGKAPRKQLATKAARKSAP+TGGVKKPHRYRPGTVALREIRRYQKSTE
Sbjct  1    MARTKQTARKSTGGKAPRKQLATKAARKSAPATGGVKKPHRYRPGTVALREIRRYQKSTE  60

Query  164  LLIRKLPFQRLVREIAQDFKTDLRFQSAAIGALQEASEAYLVGLFEDTNLCAIHAKRVTI  223
            LLIRKLPFQRLVREIAQDFKTDLRF S+A+ ALQEASEAYLVGLFEDTNLCAIHAKRVTI
Sbjct  61   LLIRKLPFQRLVREIAQDFKTDLRFXSSAVMALQEASEAYLVGLFEDTNLCAIHAKRVTI  120

Query  224  MPKDIQLARRIRGER  238
            MPKDIQLARRIRGER
Sbjct  121  MPKDIQLARRIRGER  135
```

>uncharacterized protein LOC133095661 [Eubalaena glacialis]
Sequence ID: XP_061053392.1 Length: 419
Range 1: 136 to 419

```
Score:402 bits(1032), Expect:3e-137,
Method:Compositional matrix adjust.,
Identities:229/284(81%), Positives:233/284(82%), Gaps:45/284(15%)

Query  1    MSGRGKGGKGLGKGGAKRHRKVLRDNIQGITKPAIRRLARRGGVKRISGLIYEETRGVLK  60
            MSGRGKGGKGLGKGGAKRHRKVLRDNIQGITKPAIRRLARRGGVKRISGLIYEETRGVLK
Sbjct  136  MSGRGKGGKGLGKGGAKRHRKVLRDNIQGITKPAIRRLARRGGVKRISGLIYEETRGVLK  195

Query  61   VFLENVIRDAVTYTEHAKRKTVTAMDVVYALKRQGRTLYG--------------------  100
            VFLENVIRDAVTYTEHAKRKTVTAMDVVYALKRQGRTL
Sbjct  196  VFLENVIRDAVTYTEHAKRKTVTAMDVVYALKRQGRTLNKPRTNSVGGEGRGRRGRQQSI  255

Query  101  -------------------------FGGMARTKQTARKSTGGKAPRKQLATKAARKSAPS  135
                                     +  MARTKQTARKSTGGKAPRKQLATKAARKSAP+
Sbjct  256  VQSRREGPYIYWDDWLVLLLLNNWRYLCMARTKQTARKSTGGKAPRKQLATKAARKSAPA  315

Query  136  TGGVKKPHRYRPGTVALREIRRYQKSTELLIRKLPFQRLVREIAQDFKTDLRFQSAAIGA  195
            TGGVKKPHRYRPGTVALREIRRYQKSTELLIRKLPFQRLVREIAQDFKTDLRFQS+A+ A
Sbjct  316  TGGVKKPHRYRPGTVALREIRRYQKSTELLIRKLPFQRLVREIAQDFKTDLRFQSSAVMA  375

Query  196  LQEASEAYLVGLFEDTNLCAIHAKRVTIMPKDIQLARRIRGERA  239
            LQEA EAYLVGLFEDTNLCAIHAKRVTIMPKDIQLARRIRGERA
Sbjct  376  LQEACEAYLVGLFEDTNLCAIHAKRVTIMPKDIQLARRIRGERA  419



Range 2: 1 to 135

Score:264 bits(675), Expect:4e-83,
Method:Compositional matrix adjust.,
Identities:130/135(96%), Positives:133/135(98%), Gaps:0/135(0%)

Query  104  MARTKQTARKSTGGKAPRKQLATKAARKSAPSTGGVKKPHRYRPGTVALREIRRYQKSTE  163
            MARTKQTARKSTGGKAPRKQLATKAARKSAP+TGGVKKPHRYRPGTVALREIRRYQKSTE
Sbjct  1    MARTKQTARKSTGGKAPRKQLATKAARKSAPATGGVKKPHRYRPGTVALREIRRYQKSTE  60

Query  164  LLIRKLPFQRLVREIAQDFKTDLRFQSAAIGALQEASEAYLVGLFEDTNLCAIHAKRVTI  223
            LLIRKLPFQRLVREIAQDFKTDLRFQS+A+ ALQEA EAYLVGLFEDTNLCAIHAKRVTI
Sbjct  61   LLIRKLPFQRLVREIAQDFKTDLRFQSSAVMALQEACEAYLVGLFEDTNLCAIHAKRVTI  120

Query  224  MPKDIQLARRIRGER  238
            MPKDIQLARRIRGER
Sbjct  121  MPKDIQLARRIRGER  135



>LOW QUALITY PROTEIN: uncharacterized protein LOC131481407 [Ochotona princeps]
Sequence ID: XP_058526226.1 Length: 693
Range 1: 18 to 248

Score:411 bits(1056), Expect:4e-137,
```

```
Method:Compositional matrix adjust.,
Identities:210/231(91%), Positives:213/231(92%), Gaps:10/231(4%)

Query  18   RHRKVLRDNIQGITKPAIRRLARRGGVKRISGLIYEETRGVLKVFLENVIRDAVTYTEHA  77
            RHRKVLRDNIQGITKPAIRRLARRGGVKRISGLIYEETRGVLKVFLENVIRDAVTYTEHA
Sbjct  18   RHRKVLRDNIQGITKPAIRRLARRGGVKRISGLIYEETRGVLKVFLENVIRDAVTYTEHA  77

Query  78   KRKTVTAMDVVYALKRQGRTLYGF----------GGMARTKQTARKSTGGKAPRKQLATK  127
            KRKTVTAMDVVYALKRQGRT                MARTKQTARKSTGGKAPRKQLATK
Sbjct  78   KRKTVTAMDVVYALKRQGRTAPNVLSLSLXLLQASQMARTKQTARKSTGGKAPRKQLATK  137

Query  128  AARKSAPSTGGVKKPHRYRPGTVALREIRRYQKSTELLIRKLPFQRLVREIAQDFKTDLR  187
            AARKSAP+TGGVKKPHRYRPGTVALREIRRYQKSTELLIRKLPFQRLVREIAQDFKTDLR
Sbjct  138  AARKSAPATGGVKKPHRYRPGTVALREIRRYQKSTELLIRKLPFQRLVREIAQDFKTDLR  197

Query  188  FQSAAIGALQEASEAYLVGLFEDTNLCAIHAKRVTIMPKDIQLARRIRGER  238
            FQS+A+ ALQEA EAYLVGLFEDTNLCAIHAKRVTIMPKDIQLARRIRGER
Sbjct  198  FQSSAVMALQEACEAYLVGLFEDTNLCAIHAKRVTIMPKDIQLARRIRGER  248



Range 2: 405 to 560

Score:266 bits(680), Expect:6e-81,
Method:Compositional matrix adjust.,
Identities:133/156(85%), Positives:141/156(90%), Gaps:3/156(1%)

Query  86   DVVYALKRQGRTLYGFGG---MARTKQTARKSTGGKAPRKQLATKAARKSAPSTGGVKKP  142
             ++Y  + + R   G+     MARTKQTARKSTGGKAPRKQLATKAARKSAP+TGGVKKP
Sbjct  405  SLLYKWREKSRWPSGYSDLLVMARTKQTARKSTGGKAPRKQLATKAARKSAPATGGVKKP  464

Query  143  HRYRPGTVALREIRRYQKSTELLIRKLPFQRLVREIAQDFKTDLRFQSAAIGALQEASEA  202
            HRYRPGTVALREIRRYQKSTELLIRKLPFQRLVREIAQDFKTDLRFQS+A+ ALQEA EA
Sbjct  465  HRYRPGTVALREIRRYQKSTELLIRKLPFQRLVREIAQDFKTDLRFQSSAVMALQEACEA  524

Query  203  YLVGLFEDTNLCAIHAKRVTIMPKDIQLARRIRGER  238
            YLVGLFEDTNLCAIHAKRVTIMPKDIQLARRIRGER
Sbjct  525  YLVGLFEDTNLCAIHAKRVTIMPKDIQLARRIRGER  560



Range 3: 591 to 693

Score:172 bits(437), Expect:8e-46,
Method:Compositional matrix adjust.,
Identities:103/103(100%), Positives:103/103(100%), Gaps:0/103(0%)

Query  1    MSGRGKGGKGLGKGGAKRHRKVLRDNIQGITKPAIRRLARRGGVKRISGLIYEETRGVLK  60
            MSGRGKGGKGLGKGGAKRHRKVLRDNIQGITKPAIRRLARRGGVKRISGLIYEETRGVLK
Sbjct  591  MSGRGKGGKGLGKGGAKRHRKVLRDNIQGITKPAIRRLARRGGVKRISGLIYEETRGVLK  650

Query  61   VFLENVIRDAVTYTEHAKRKTVTAMDVVYALKRQGRTLYGFGG  103
```

```
            VFLENVIRDAVTYTEHAKRKTVTAMDVVYALKRQGRTLYGFGG
Sbjct  651  VFLENVIRDAVTYTEHAKRKTVTAMDVVYALKRQGRTLYGFGG  693
```

>uncharacterized protein LOC102093168 [Columba livia]
Sequence ID: XP_064908964.1 Length: 423
Range 1: 18 to 238

Score:401 bits(1031), Expect:5e-137,
Method:Compositional matrix adjust.,
Identities:206/221(93%), Positives:210/221(95%), Gaps:7/221(3%)

```
Query  18   RHRKVLRDNIQGITKPAIRRLARRGGVKRISGLIYEETRGVLKVFLENVIRDAVTYTEHA  77
            RHRKVLRDNIQGITKPAIRRLARRGGVKRISGLIYEETRGVLKVFLENVIRDAVTYTEHA
Sbjct  18   RHRKVLRDNIQGITKPAIRRLARRGGVKRISGLIYEETRGVLKVFLENVIRDAVTYTEHA  77

Query  78   KRKTVTAMDVVYALKRQGR----TLY---GFGGMARTKQTARKSTGGKAPRKQLATKAAR  130
            KRKTVTAMDVVYALKRQGR    +LY   G   MARTKQTARKSTGGKAPRKQLATKAAR
Sbjct  78   KRKTVTAMDVVYALKRQGRRASTSLYCRRGRTAMARTKQTARKSTGGKAPRKQLATKAAR  137

Query  131  KSAPSTGGVKKPHRYRPGTVALREIRRYQKSTELLIRKLPFQRLVREIAQDFKTDLRFQS  190
            KSAP+TGGVKKPHRYRPGTVALREIRRYQKSTELLIRKLPFQRLVREIAQDFKTDLRFQS
Sbjct  138  KSAPATGGVKKPHRYRPGTVALREIRRYQKSTELLIRKLPFQRLVREIAQDFKTDLRFQS  197

Query  191  AAIGALQEASEAYLVGLFEDTNLCAIHAKRVTIMPKDIQLA  231
            +A+ ALQEASEAYLVGLFEDTNLCAIHAKRVTIMPKDIQLA
Sbjct  198  SAVMALQEASEAYLVGLFEDTNLCAIHAKRVTIMPKDIQLA  238
```

Range 2: 331 to 423

Score:172 bits(435), Expect:3e-47,
Method:Compositional matrix adjust.,
Identities:93/93(100%), Positives:93/93(100%), Gaps:0/93(0%)

```
Query  11   LGKGGAKRHRKVLRDNIQGITKPAIRRLARRGGVKRISGLIYEETRGVLKVFLENVIRDA  70
            LGKGGAKRHRKVLRDNIQGITKPAIRRLARRGGVKRISGLIYEETRGVLKVFLENVIRDA
Sbjct  331  LGKGGAKRHRKVLRDNIQGITKPAIRRLARRGGVKRISGLIYEETRGVLKVFLENVIRDA  390

Query  71   VTYTEHAKRKTVTAMDVVYALKRQGRTLYGFGG  103
            VTYTEHAKRKTVTAMDVVYALKRQGRTLYGFGG
Sbjct  391  VTYTEHAKRKTVTAMDVVYALKRQGRTLYGFGG  423
```

>uncharacterized protein LOC134934338 [Pseudophryne corroboree]
Sequence ID: XP_063785865.1 Length: 344
Range 1: 1 to 239

Score:398 bits(1022), Expect:9e-137,
Method:Compositional matrix adjust.,
Identities:207/239(87%), Positives:211/239(88%), Gaps:23/239(9%)

```
Query  1    MSGRGKGGKGLGKGGAKRHRKVLRDNIQGITKPAIRRLARRGGVKRISGLIYEETRGVLK  60
            MSGRGKGGKGLGKGGAKRHRKVLRDNIQGITKPAIRRLARRGGVKRISGLIYEETRGVLK
Sbjct  1    MSGRGKGGKGLGKGGAKRHRKVLRDNIQGITKPAIRRLARRGGVKRISGLIYEETRGVLK  60

Query  61   VFLENVIRDAVTYTEHAKRKTVTAMDVVYALKRQGRTLYGFG------------------  102
            VFLENVIRDAVTYTEHAKRKTVTAMDVVYALKRQGRTLYGFG
Sbjct  61   VFLENVIRDAVTYTEHAKRKTVTAMDVVYALKRQGRTLYGFGETLAAAPVHFILASFYYS  120

Query  103  -----GMARTKQTARKSTGGKAPRKQLATKAARKSAPSTGGVKKPHRYRPGTVALREIRR  157
                  MARTKQTARKSTGGKAPRKQLATKAARKSAP+TGGVKKPHRYRPGTVALREIRR
Sbjct  121  SVEYRKMARTKQTARKSTGGKAPRKQLATKAARKSAPATGGVKKPHRYRPGTVALREIRR  180

Query  158  YQKSTELLIRKLPFQRLVREIAQDFKTDLRFQSAAIGALQEASEAYLVGLFEDTNLCAI  216
            YQKSTELLIRKLPFQRLVREIAQDFKTDLRFQS+A+ ALQEASEA LVGLFEDTNL  +
Sbjct  181  YQKSTELLIRKLPFQRLVREIAQDFKTDLRFQSSAVMALQEASEANLVGLFEDTNLAIL  239
```

Range 2: 242 to 344

Score:206 bits(524), Expect:2e-61,
Method:Compositional matrix adjust.,
Identities:103/103(100%), Positives:103/103(100%), Gaps:0/103(0%)

```
Query  1    MSGRGKGGKGLGKGGAKRHRKVLRDNIQGITKPAIRRLARRGGVKRISGLIYEETRGVLK  60
            MSGRGKGGKGLGKGGAKRHRKVLRDNIQGITKPAIRRLARRGGVKRISGLIYEETRGVLK
Sbjct  242  MSGRGKGGKGLGKGGAKRHRKVLRDNIQGITKPAIRRLARRGGVKRISGLIYEETRGVLK  301

Query  61   VFLENVIRDAVTYTEHAKRKTVTAMDVVYALKRQGRTLYGFGG  103
            VFLENVIRDAVTYTEHAKRKTVTAMDVVYALKRQGRTLYGFGG
Sbjct  302  VFLENVIRDAVTYTEHAKRKTVTAMDVVYALKRQGRTLYGFGG  344
```

>uncharacterized protein LOC121274916 [Carcharodon carcharias]
Sequence ID: XP_041038229.1 Length: 874
Range 1: 18 to 247

Score:415 bits(1067), Expect:1e-136,
Method:Compositional matrix adjust.,
Identities:211/230(92%), Positives:216/230(93%), Gaps:8/230(3%)

```
Query  18   RHRKVLRDNIQGITKPAIRRLARRGGVKRISGLIYEETRGVLKVFLENVIRDAVTYTEHA  77
            RHRKVLRDNIQGITKPAIRRLARRGGVKRISGLIYEETRGVLKVFLENVIRDAVTYTEHA
Sbjct  18   RHRKVLRDNIQGITKPAIRRLARRGGVKRISGLIYEETRGVLKVFLENVIRDAVTYTEHA  77

Query  78   KRKTVTAMDVVYALKRQGRTLYG--------FGGMARTKQTARKSTGGKAPRKQLATKAA  129
            KRKTVTAMDVVYALKRQG+ L+         F  MARTKQTARKSTGGKAPRKQLATKAA
```

```
Sbjct  78   KRKTVTAMDVVYALKRQGQRLHHSQSLCSTVFVIMARTKQTARKSTGGKAPRKQLATKAA  137

Query  130  RKSAPSTGGVKKPHRYRPGTVALREIRRYQKSTELLIRKLPFQRLVREIAQDFKTDLRFQ  189
            RKSAP+TGGVKKPHRYRPGTVALREIRRYQKSTELLIRKLPFQRLVREIAQDFKTDLRFQ
Sbjct  138  RKSAPATGGVKKPHRYRPGTVALREIRRYQKSTELLIRKLPFQRLVREIAQDFKTDLRFQ  197

Query  190  SAAIGALQEASEAYLVGLFEDTNLCAIHAKRVTIMPKDIQLARRIRGERA  239
            S+A+ ALQEASEAYLVGLFEDTNLCAIHAKRVTIMPKDIQLARRIRGE A
Sbjct  198  SSAVMALQEASEAYLVGLFEDTNLCAIHAKRVTIMPKDIQLARRIRGEGA  247
```

Range 2: 651 to 874

Score:399 bits(1025), Expect:2e-130,
Method:Compositional matrix adjust.,
Identities:203/224(91%), Positives:207/224(92%), Gaps:3/224(1%)

```
Query  19   HRKVLRDNIQGITKPAIRRLARRGGVKRISGLIYEETRGVLKVFLENVIRDAVTYTEHAK  78
            HRKVLRDNIQGITKPAIRRLARRGGVKRISGLIYEETRGVLKVFLENVIRDAVTYTEHAK
Sbjct  651  HRKVLRDNIQGITKPAIRRLARRGGVKRISGLIYEETRGVLKVFLENVIRDAVTYTEHAK  710

Query  79   RKTVTAMDVVYALK---RQGRTLYGFGGMARTKQTARKSTGGKAPRKQLATKAARKSAPS  135
            RKTVTAMDV    +    Q      F  MARTKQTARKSTGGKAPRKQLATKAARKSAP+
Sbjct  711  RKTVTAMDVTDTERLHHSQSLCSTAFVIMARTKQTARKSTGGKAPRKQLATKAARKSAPA  770

Query  136  TGGVKKPHRYRPGTVALREIRRYQKSTELLIRKLPFQRLVREIAQDFKTDLRFQSAAIGA  195
            TGGVKKPHRYRPGTVALREIRRYQKSTELLIRKLPFQRLVREIAQDFKTDLRFQS+A+ A
Sbjct  771  TGGVKKPHRYRPGTVALREIRRYQKSTELLIRKLPFQRLVREIAQDFKTDLRFQSSAVMA  830

Query  196  LQEASEAYLVGLFEDTNLCAIHAKRVTIMPKDIQLARRIRGERA  239
            LQEASEAYLVGLFEDTNLCAIHAKRVTIMPKDIQLARRIRGERA
Sbjct  831  LQEASEAYLVGLFEDTNLCAIHAKRVTIMPKDIQLARRIRGERA  874
```

>uncharacterized protein LOC118903524 [Balaenoptera musculus]
Sequence ID: XP_036724574.1 Length: 473
Range 1: 190 to 473

Score:402 bits(1033), Expect:1e-136,
Method:Compositional matrix adjust.,
Identities:229/284(81%), Positives:233/284(82%), Gaps:45/284(15%)

```
Query  1    MSGRGKGGKGLGKGGAKRHRKVLRDNIQGITKPAIRRLARRGGVKRISGLIYEETRGVLK  60
            MSGRGKGGKGLGKGGAKRHRKVLRDNIQGITKPAIRRLARRGGVKRISGLIYEETRGVLK
Sbjct  190  MSGRGKGGKGLGKGGAKRHRKVLRDNIQGITKPAIRRLARRGGVKRISGLIYEETRGVLK  249

Query  61   VFLENVIRDAVTYTEHAKRKTVTAMDVVYALKRQGRTL----------------------  98
            VFLENVIRDAVTYTEHAKRKTVTAMDVVYALKRQGRTL
Sbjct  250  VFLENVIRDAVTYTEHAKRKTVTAMDVVYALKRQGRTLNKPRTNSVGGEGRGRRGRQQSI  309
```

```
Query  99   -----------------------YGFGGMARTKQTARKSTGGKAPRKQLATKAARKSAPS  135
                                   +    MARTKQTARKSTGGKAPRKQLATKAARKSAP+
Sbjct  310  VQSRREGPYIYWDDWFLLLLLNNWRCLCMARTKQTARKSTGGKAPRKQLATKAARKSAPA  369

Query  136  TGGVKKPHRYRPGTVALREIRRYQKSTELLIRKLPFQRLVREIAQDFKTDLRFQSAAIGA  195
            TGGVKKPHRYRPGTVALREIRRYQKSTELLIRKLPFQRLVREIAQDFKTDLRFQS+A+ A
Sbjct  370  TGGVKKPHRYRPGTVALREIRRYQKSTELLIRKLPFQRLVREIAQDFKTDLRFQSSAVMA  429

Query  196  LQEASEAYLVGLFEDTNLCAIHAKRVTIMPKDIQLARRIRGERA  239
            LQEA EAYLVGLFEDTNLCAIHAKRVTIMPKDIQLARRIRGERA
Sbjct  430  LQEACEAYLVGLFEDTNLCAIHAKRVTIMPKDIQLARRIRGERA  473
```

Range 2: 1 to 135

Score:263 bits(673), Expect:3e-82,
Method:Compositional matrix adjust.,
Identities:130/135(96%), Positives:133/135(98%), Gaps:0/135(0%)

```
Query  104  MARTKQTARKSTGGKAPRKQLATKAARKSAPSTGGVKKPHRYRPGTVALREIRRYQKSTE  163
            MARTKQTARKSTGGKAPRKQLATKAARKSAP+TGGVKKPHRYRPGTVALREIRRYQKSTE
Sbjct  1    MARTKQTARKSTGGKAPRKQLATKAARKSAPATGGVKKPHRYRPGTVALREIRRYQKSTE  60

Query  164  LLIRKLPFQRLVREIAQDFKTDLRFQSAAIGALQEASEAYLVGLFEDTNLCAIHAKRVTI  223
            LLIRKLPFQRLVREIAQDFKTDLRFQS+A+ ALQEA EAYLVGLFEDTNLCAIHAKRVTI
Sbjct  61   LLIRKLPFQRLVREIAQDFKTDLRFQSSAVMALQEACEAYLVGLFEDTNLCAIHAKRVTI  120

Query  224  MPKDIQLARRIRGER  238
            MPKDIQLARRIRGER
Sbjct  121  MPKDIQLARRIRGER  135
```

>uncharacterized protein LOC114233576 [Eptesicus fuscus]
Sequence ID: XP_054568212.1 Length: 553
Range 1: 18 to 243

Score:405 bits(1040), Expect:1e-136,
Method:Compositional matrix adjust.,
Identities:206/226(91%), Positives:210/226(92%), Gaps:9/226(3%)

```
Query  18   RHRKVLRDNIQGITKPAIRRLARRGGVKRISGLIYEETRGVLKVFLENVIRDAVTYTEHA  77
            RHRKVLRDNIQGITKPAIRRLARRGGVKRISGLIYEETRGVLKVFLENVIRDAVTYTEHA
Sbjct  18   RHRKVLRDNIQGITKPAIRRLARRGGVKRISGLIYEETRGVLKVFLENVIRDAVTYTEHA  77

Query  78   KRKTVTAMDVVYALKRQGR-------TLYGFG--GMARTKQTARKSTGGKAPRKQLATKA  128
            KRKTVTAMDVVYALKRQGR         + F    MARTKQTARKSTGGKAPRKQLATKA
Sbjct  78   KRKTVTAMDVVYALKRQGRRASPLTHPQFNFSSFAMARTKQTARKSTGGKAPRKQLATKA  137

Query  129  ARKSAPSTGGVKKPHRYRPGTVALREIRRYQKSTELLIRKLPFQRLVREIAQDFKTDLRF  188
            ARKSAP+TGGVKKPHRYRPGTVALREIRRYQKSTELLIRKLPFQRLVREIAQDFKTDLRF
```

```
Sbjct  138  ARKSAPATGGVKKPHRYRPGTVALREIRRYQKSTELLIRKLPFQRLVREIAQDFKTDLRF  197

Query  189  QSAAIGALQEASEAYLVGLFEDTNLCAIHAKRVTIMPKDIQLARRI  234
            QS+A+ ALQEA EAYLVGLFEDTNLCAIHAKRVTIMPKDIQLARRI
Sbjct  198  QSSAVMALQEACEAYLVGLFEDTNLCAIHAKRVTIMPKDIQLARRI  243
```

Range 2: 310 to 445

Score:266 bits(681), Expect:2e-82,
Method:Compositional matrix adjust.,
Identities:131/136(96%), Positives:134/136(98%), Gaps:0/136(0%)

```
Query  104  MARTKQTARKSTGGKAPRKQLATKAARKSAPSTGGVKKPHRYRPGTVALREIRRYQKSTE  163
            MARTKQTARKSTGGKAPRKQLATKAARKSAP+TGGVKKPHRYRPGTVALREIRRYQKSTE
Sbjct  310  MARTKQTARKSTGGKAPRKQLATKAARKSAPATGGVKKPHRYRPGTVALREIRRYQKSTE  369

Query  164  LLIRKLPFQRLVREIAQDFKTDLRFQSAAIGALQEASEAYLVGLFEDTNLCAIHAKRVTI  223
            LLIRKLPFQRLVREIAQDFKTDLRFQS+A+ ALQEA EAYLVGLFEDTNLCAIHAKRVTI
Sbjct  370  LLIRKLPFQRLVREIAQDFKTDLRFQSSAVMALQEACEAYLVGLFEDTNLCAIHAKRVTI  429

Query  224  MPKDIQLARRIRGERA  239
            MPKDIQLARRIRGERA
Sbjct  430  MPKDIQLARRIRGERA  445
```

Range 3: 451 to 553

Score:174 bits(441), Expect:4e-47,
Method:Compositional matrix adjust.,
Identities:103/103(100%), Positives:103/103(100%), Gaps:0/103(0%)

```
Query  1    MSGRGKGGKGLGKGGAKRHRKVLRDNIQGITKPAIRRLARRGGVKRISGLIYEETRGVLK  60
            MSGRGKGGKGLGKGGAKRHRKVLRDNIQGITKPAIRRLARRGGVKRISGLIYEETRGVLK
Sbjct  451  MSGRGKGGKGLGKGGAKRHRKVLRDNIQGITKPAIRRLARRGGVKRISGLIYEETRGVLK  510

Query  61   VFLENVIRDAVTYTEHAKRKTVTAMDVVYALKRQGRTLYGFGG  103
            VFLENVIRDAVTYTEHAKRKTVTAMDVVYALKRQGRTLYGFGG
Sbjct  511  VFLENVIRDAVTYTEHAKRKTVTAMDVVYALKRQGRTLYGFGG  553
```

>uncharacterized protein LOC104861624 [Fukomys damarensis]
Sequence ID: XP_010621402.1 Length: 625
Range 1: 18 to 245

Score:407 bits(1045), Expect:2e-136,
Method:Compositional matrix adjust.,
Identities:210/228(92%), Positives:213/228(93%), Gaps:7/228(3%)

```
Query  18   RHRKVLRDNIQGITKPAIRRLARRGGVKRISGLIYEETRGVLKVFLENVIRDAVTYTEHA  77
            RHRKVLRDNIQGITKPAIRRLARRGGVKRISGLIYEETRGVLKVFLENVIRDAVTYTEHA
Sbjct  18   RHRKVLRDNIQGITKPAIRRLARRGGVKRISGLIYEETRGVLKVFLENVIRDAVTYTEHA  77

Query  78   KRKTVTAMDVVYALKRQGR-------TLYGFGGMARTKQTARKSTGGKAPRKQLATKAAR  130
            KRKTVTAMDVVYALKRQGR          G   MARTKQTARKSTGGKAPRKQLATKAAR
Sbjct  78   KRKTVTAMDVVYALKRQGRRAPQAICVSSGKQVMARTKQTARKSTGGKAPRKQLATKAAR  137

Query  131  KSAPSTGGVKKPHRYRPGTVALREIRRYQKSTELLIRKLPFQRLVREIAQDFKTDLRFQS  190
            KSAP+TGGVKKPHRYRPGTVALREIRRYQKSTELLIRKLPFQRLVREIAQDFKTDLRFQS
Sbjct  138  KSAPATGGVKKPHRYRPGTVALREIRRYQKSTELLIRKLPFQRLVREIAQDFKTDLRFQS  197

Query  191  AAIGALQEASEAYLVGLFEDTNLCAIHAKRVTIMPKDIQLARRIRGER  238
            +A+ ALQEA EAYLVGLFEDTNLCAIHAKRVTIMPKDIQLARRIRGER
Sbjct  198  SAVMALQEACEAYLVGLFEDTNLCAIHAKRVTIMPKDIQLARRIRGER  245
```

Range 2: 533 to 625

Score:166 bits(420), Expect:1e-43,
Method:Compositional matrix adjust.,
Identities:91/93(98%), Positives:91/93(97%), Gaps:0/93(0%)

```
Query  11   LGKGGAKRHRKVLRDNIQGITKPAIRRLARRGGVKRISGLIYEETRGVLKVFLENVIRDA  70
            LGKGGAKRHRKVLRDNIQGITKPAIRRLA RGGVKRISGLIYEETRGVLKVFLENV RDA
Sbjct  533  LGKGGAKRHRKVLRDNIQGITKPAIRRLALRGGVKRISGLIYEETRGVLKVFLENVTRDA  592

Query  71   VTYTEHAKRKTVTAMDVVYALKRQGRTLYGFGG  103
            VTYTEHAKRKTVTAMDVVYALKRQGRTLYGFGG
Sbjct  593  VTYTEHAKRKTVTAMDVVYALKRQGRTLYGFGG  625
```

>histone H3-like centromeric protein CSE4, partial [Equus quagga]
Sequence ID: XP_046507372.1 Length: 213
Range 1: 1 to 213

Score:388 bits(996), Expect:6e-135,
Method:Compositional matrix adjust.,
Identities:199/213(93%), Positives:202/213(94%), Gaps:5/213(2%)

```
Query  1    MSGRGKGGKGLGKGGAKRHRKVLRDNIQGITKPAIRRLARRGGVKRISGLIYEETRGVLK  60
            MSGRGKGGKGLGKGGAKRHRKVLRDNIQGITKPAIRRLARRGGVKRISGLIYEETRGVLK
Sbjct  1    MSGRGKGGKGLGKGGAKRHRKVLRDNIQGITKPAIRRLARRGGVKRISGLIYEETRGVLK  60

Query  61   VFLENVIRDAVTYTEHAKRKTVTAMDVVYALKRQGRTLYGFGG-----MARTKQTARKST  115
            VFLENVIRDAVTYTEHAKRKTVTAMDVVYALKRQGRTLY          MARTKQTARKST
Sbjct  61   VFLENVIRDAVTYTEHAKRKTVTAMDVVYALKRQGRTLYVLSSWLCFTMARTKQTARKST  120

Query  116  GGKAPRKQLATKAARKSAPSTGGVKKPHRYRPGTVALREIRRYQKSTELLIRKLPFQRLV  175
```

```
                GGKAPRKQLATKAARKSAP+TGGVKKPHRYRPGTVALREIRRYQKSTELLIRKLPFQRLV
Sbjct  121  GGKAPRKQLATKAARKSAPATGGVKKPHRYRPGTVALREIRRYQKSTELLIRKLPFQRLV  180

Query  176  REIAQDFKTDLRFQSAAIGALQEASEAYLVGLF  208
            REIAQDFKTDLRFQS+A+ ALQEA EAYLVGLF
Sbjct  181  REIAQDFKTDLRFQSSAVMALQEACEAYLVGLF  213
```

>uncharacterized protein LOC122544485 [Chiloscyllium plagiosum]
Sequence ID: XP_043539660.1 Length: 384
Range 1: 148 to 384

Score:394 bits(1012), Expect:1e-134,
Method:Compositional matrix adjust.,
Identities:205/237(86%), Positives:210/237(88%), Gaps:15/237(6%)

```
Query  18   RHRKVLRDNIQGITKPAIRRLARRGGVKRISGLIYEETRGVLKVFLENVIRDAVTYTEHA  77
            RHRKVLRDNIQGITKPAIRRLARRGGVKRISGLIYEETRGVLKVFLENVIRDAVTYTEHA
Sbjct  148  RHRKVLRDNIQGITKPAIRRLARRGGVKRISGLIYEETRGVLKVFLENVIRDAVTYTEHA  207

Query  78   KRKTVTAMDV---VYALKRQGRTLYG------------FGGMARTKQTARKSTGGKAPRK  122
            KRKTVTAMDV      L R+ R+                   MARTKQTARKSTGGKAPRK
Sbjct  208  KRKTVTAMDVGGISVPLNREHRSSLSIDSSSLCVCYRELKRMARTKQTARKSTGGKAPRK  267

Query  123  QLATKAARKSAPSTGGVKKPHRYRPGTVALREIRRYQKSTELLIRKLPFQRLVREIAQDF  182
            QLATKAARKSAP+TGGVKKPHRYRPGTVALREIRRYQKSTELLIRKLPFQRLVREIAQDF
Sbjct  268  QLATKAARKSAPATGGVKKPHRYRPGTVALREIRRYQKSTELLIRKLPFQRLVREIAQDF  327

Query  183  KTDLRFQSAAIGALQEASEAYLVGLFEDTNLCAIHAKRVTIMPKDIQLARRIRGERA  239
            KTDLRFQS+A+ ALQEASEAYLVGLFEDTNLCAIHAKRVTIMPKDIQLARRIRGERA
Sbjct  328  KTDLRFQSSAVMALQEASEAYLVGLFEDTNLCAIHAKRVTIMPKDIQLARRIRGERA  384
```

Range 2: 1 to 132

Score:258 bits(659), Expect:4e-81,
Method:Compositional matrix adjust.,
Identities:126/132(95%), Positives:130/132(98%), Gaps:0/132(0%)

```
Query  104  MARTKQTARKSTGGKAPRKQLATKAARKSAPSTGGVKKPHRYRPGTVALREIRRYQKSTE  163
            MARTKQTA KSTGGKAPRKQLATKAARKSAP+TGGVKKPHRYRPGTVALREIRRYQKSTE
Sbjct  1    MARTKQTAHKSTGGKAPRKQLATKAARKSAPATGGVKKPHRYRPGTVALREIRRYQKSTE  60

Query  164  LLIRKLPFQRLVREIAQDFKTDLRFQSAAIGALQEASEAYLVGLFEDTNLCAIHAKRVTI  223
            LLIRKLPFQRLVREIAQDFKTDLRFQS+A+ ALQEASEAY+VGLFEDTNLCAIHAKRVTI
Sbjct  61   LLIRKLPFQRLVREIAQDFKTDLRFQSSAVMALQEASEAYMVGLFEDTNLCAIHAKRVTI  120

Query  224  MPKDIQLARRIR  235
            MPKDIQLARRIR
Sbjct  121  MPKDIQLARRIR  132
```

>LOW QUALITY PROTEIN: uncharacterized protein LOC133339839 [Lethenteron reissneri]
Sequence ID: XP_061403957.1 Length: 701
Range 1: 18 to 245

Score:404 bits(1039), Expect:2e-134,
Method:Compositional matrix adjust.,
Identities:208/229(91%), Positives:211/229(92%), Gaps:8/229(3%)

```
Query  18   RHRKVLRDNIQGITKPAIRRLARRGGVKRISGLIYEETRGVLKVFLENVIRDAVTYTEHA  77
            RHRKVLRDNIQGITKPAIRRLARRGGVKRISGLIYEETR VLKVFLENVIRDAVTYTEHA
Sbjct  18   RHRKVLRDNIQGITKPAIRRLARRGGVKRISGLIYEETR-VLKVFLENVIRDAVTYTEHA  76

Query  78   KRKTVTAMDVVYALKRQGRTLY-------GFGGMARTKQTARKSTGGKAPRKQLATKAAR  130
            KRKTVTAMDVVYALKRQG           G   MARTKQTARKSTGGKAPRKQLATKAAR
Sbjct  77   KRKTVTAMDVVYALKRQGHRSATAAPRRAGQAPMARTKQTARKSTGGKAPRKQLATKAAR  136

Query  131  KSAPSTGGVKKPHRYRPGTVALREIRRYQKSTELLIRKLPFQRLVREIAQDFKTDLRFQS  190
            KSAP+TGGVK PHRYRPGTVALREIRRYQKSTELLIRKLPFQRLVREIAQDFKTDLRFQS
Sbjct  137  KSAPATGGVKXPHRYRPGTVALREIRRYQKSTELLIRKLPFQRLVREIAQDFKTDLRFQS  196

Query  191  AAIGALQEASEAYLVGLFEDTNLCAIHAKRVTIMPKDIQLARRIRGERA  239
            +A+ ALQEASEAYLV LFEDTNLCAIHAKRVTIMPKDIQLARRIRGERA
Sbjct  197  SAVMALQEASEAYLVALFEDTNLCAIHAKRVTIMPKDIQLARRIRGERA  245
```

Range 2: 599 to 701

Score:174 bits(441), Expect:2e-46,
Method:Compositional matrix adjust.,
Identities:103/103(100%), Positives:103/103(100%), Gaps:0/103(0%)

```
Query  1    MSGRGKGGKGLGKGGAKRHRKVLRDNIQGITKPAIRRLARRGGVKRISGLIYEETRGVLK  60
            MSGRGKGGKGLGKGGAKRHRKVLRDNIQGITKPAIRRLARRGGVKRISGLIYEETRGVLK
Sbjct  599  MSGRGKGGKGLGKGGAKRHRKVLRDNIQGITKPAIRRLARRGGVKRISGLIYEETRGVLK  658

Query  61   VFLENVIRDAVTYTEHAKRKTVTAMDVVYALKRQGRTLYGFGG  103
            VFLENVIRDAVTYTEHAKRKTVTAMDVVYALKRQGRTLYGFGG
Sbjct  659  VFLENVIRDAVTYTEHAKRKTVTAMDVVYALKRQGRTLYGFGG  701
```

>uncharacterized protein LOC119039157 [Artibeus jamaicensis]
Sequence ID: XP_036987727.2 Length: 426
Range 1: 195 to 426

Score:394 bits(1013), Expect:4e-134,

Method:Compositional matrix adjust.,
Identities:207/234(88%), Positives:211/234(90%), Gaps:14/234(5%)

```
Query  18   RHRKVLRDNIQGITKPAIRRLARRGGVKRISGLIYEETRGVLKVFLENVIRDAVTYTEHA  77
            RHRKVLRDNIQGITKPAIRRLARRGGVKRISGLIYEETRGVLKVFLENVIRDAVTYTEHA
Sbjct  195  RHRKVLRDNIQGITKPAIRRLARRGGVKRISGLIYEETRGVLKVFLENVIRDAVTYTEHA  254

Query  78   KRKTVTAMDVV-------YALK--RQGRTLY---GFGGMARTKQTARKSTGGKAPRKQLA  125
            KRKTVTAMDV        +  K   QGR L     F  MARTKQTARKSTGGKAPRKQLA
Sbjct  255  KRKTVTAMDVTPLPATITFPPKGPFQGRPLSLRKSF--MARTKQTARKSTGGKAPRKQLA  312

Query  126  TKAARKSAPSTGGVKKPHRYRPGTVALREIRRYQKSTELLIRKLPFQRLVREIAQDFKTD  185
            TKAARKSAP+TGGVKKPHRYRPGTVALREIRRYQKSTELLIRKLPFQRLVREIAQDFKTD
Sbjct  313  TKAARKSAPATGGVKKPHRYRPGTVALREIRRYQKSTELLIRKLPFQRLVREIAQDFKTD  372

Query  186  LRFQSAAIGALQEASEAYLVGLFEDTNLCAIHAKRVTIMPKDIQLARRIRGERA  239
            LRFQS+A+ ALQEA EAYLVGLFEDTNLCAIHAKRVTIMPKDIQLARRIRGERA
Sbjct  373  LRFQSSAVMALQEACEAYLVGLFEDTNLCAIHAKRVTIMPKDIQLARRIRGERA  426
```

Range 2: 1 to 135

Score:265 bits(677), Expect:2e-83,
Method:Compositional matrix adjust.,
Identities:130/135(96%), Positives:133/135(98%), Gaps:0/135(0%)

```
Query  104  MARTKQTARKSTGGKAPRKQLATKAARKSAPSTGGVKKPHRYRPGTVALREIRRYQKSTE  163
            MARTKQTARKSTGGKAPRKQLATKAARKSAP+TGGVKKPHRYRPGTVALREIRRYQKSTE
Sbjct  1    MARTKQTARKSTGGKAPRKQLATKAARKSAPATGGVKKPHRYRPGTVALREIRRYQKSTE  60

Query  164  LLIRKLPFQRLVREIAQDFKTDLRFQSAAIGALQEASEAYLVGLFEDTNLCAIHAKRVTI  223
            LLIRKLPFQRLVREIAQDFKTDLRFQS+A+ ALQEA EAYLVGLFEDTNLCAIHAKRVTI
Sbjct  61   LLIRKLPFQRLVREIAQDFKTDLRFQSSAVMALQEACEAYLVGLFEDTNLCAIHAKRVTI  120

Query  224  MPKDIQLARRIRGER  238
            MPKDIQLARRIRGER
Sbjct  121  MPKDIQLARRIRGER  135
```

>uncharacterized protein LOC116787389 [Chiroxiphia lanceolata]
Sequence ID: XP_032544847.1 Length: 502
Range 1: 18 to 238

Score:396 bits(1018), Expect:5e-134,
Method:Compositional matrix adjust.,
Identities:203/221(92%), Positives:206/221(93%), Gaps:7/221(3%)

```
Query  18   RHRKVLRDNIQGITKPAIRRLARRGGVKRISGLIYEETRGVLKVFLENVIRDAVTYTEHA  77
            RHRKVLRDNIQGITKPAIRRLARRGGVKRISGLIYEETRGVLKVFLENVIRDAVTYTEHA
Sbjct  18   RHRKVLRDNIQGITKPAIRRLARRGGVKRISGLIYEETRGVLKVFLENVIRDAVTYTEHA  77
```

```
Query  78   KRKTVTAMDVVYALKRQGRTLYGF-------GGMARTKQTARKSTGGKAPRKQLATKAAR  130
            KRKTVTAMDVVYALKRQGR              MARTKQTARKSTGGKAPRKQLATKAAR
Sbjct  78   KRKTVTAMDVVYALKRQGRRPPELLHLRAAPQDMARTKQTARKSTGGKAPRKQLATKAAR  137

Query  131  KSAPSTGGVKKPHRYRPGTVALREIRRYQKSTELLIRKLPFQRLVREIAQDFKTDLRFQS  190
            KSAP+TGGVKKPHRYRPGTVALREIRRYQKSTELLIRKLPFQRLVREIAQDFKTDLRFQS
Sbjct  138  KSAPATGGVKKPHRYRPGTVALREIRRYQKSTELLIRKLPFQRLVREIAQDFKTDLRFQS  197

Query  191  AAIGALQEASEAYLVGLFEDTNLCAIHAKRVTIMPKDIQLA  231
            +A+ ALQEASEAYLVGLFEDTNLCAIHAKRVTIMPKDIQLA
Sbjct  198  SAVMALQEASEAYLVGLFEDTNLCAIHAKRVTIMPKDIQLA  238
```

Range 2: 410 to 502

Score:172 bits(437), Expect:7e-47,
Method:Compositional matrix adjust.,
Identities:93/93(100%), Positives:93/93(100%), Gaps:0/93(0%)

```
Query  11   LGKGGAKRHRKVLRDNIQGITKPAIRRLARRGGVKRISGLIYEETRGVLKVFLENVIRDA  70
            LGKGGAKRHRKVLRDNIQGITKPAIRRLARRGGVKRISGLIYEETRGVLKVFLENVIRDA
Sbjct  410  LGKGGAKRHRKVLRDNIQGITKPAIRRLARRGGVKRISGLIYEETRGVLKVFLENVIRDA  469

Query  71   VTYTEHAKRKTVTAMDVVYALKRQGRTLYGFGG  103
            VTYTEHAKRKTVTAMDVVYALKRQGRTLYGFGG
Sbjct  470  VTYTEHAKRKTVTAMDVVYALKRQGRTLYGFGG  502
```

>uncharacterized protein LOC122150844 [Orycteropus afer afer]
Sequence ID: XP_042638117.1 Length: 358
Range 1: 132 to 358

Score:388 bits(997), Expect:8e-133,
Method:Compositional matrix adjust.,
Identities:199/227(88%), Positives:205/227(90%), Gaps:5/227(2%)

```
Query  18   RHRKVLRDNIQGITKPAIRRLARRGGVKRISGLIYEETRGVLKVFLENVIRDAVTYTEHA  77
            R  +VLRDNIQGITKPAIRRLARRGGVKRISGLIYEETRGVLKVFLENVIRDAVTYTEHA
Sbjct  132  RGERVLRDNIQGITKPAIRRLARRGGVKRISGLIYEETRGVLKVFLENVIRDAVTYTEHA  191

Query  78   KRKTVTAMDVVYALKRQG-----RTLYGFGGMARTKQTARKSTGGKAPRKQLATKAARKS  132
            KRKTVTAMD     +R       + L     MARTKQTARKSTGGKAPRKQLATKAARKS
Sbjct  192  KRKTVTAMDATVFSRRSAGGALVKRLLISRSMARTKQTARKSTGGKAPRKQLATKAARKS  251

Query  133  APSTGGVKKPHRYRPGTVALREIRRYQKSTELLIRKLPFQRLVREIAQDFKTDLRFQSAA  192
            AP+TGGVKKPHRYRPGTVALREIRRYQKSTELLIRKLPFQRLVREIAQDFKTDLRFQS+A
Sbjct  252  APATGGVKKPHRYRPGTVALREIRRYQKSTELLIRKLPFQRLVREIAQDFKTDLRFQSSA  311

Query  193  IGALQEASEAYLVGLFEDTNLCAIHAKRVTIMPKDIQLARRIRGERA  239
```

```
            + ALQEA EAYLVGLFEDTNLCAIHAKRVTIMPKDIQLARRIRGERA
Sbjct  312  VMALQEACEAYLVGLFEDTNLCAIHAKRVTIMPKDIQLARRIRGERA  358
```

Range 2: 1 to 135

Score:265 bits(678), Expect:2e-84,
Method:Compositional matrix adjust.,
Identities:130/135(96%), Positives:133/135(98%), Gaps:0/135(0%)

```
Query  104  MARTKQTARKSTGGKAPRKQLATKAARKSAPSTGGVKKPHRYRPGTVALREIRRYQKSTE  163
            MARTKQTARKSTGGKAPRKQLATKAARKSAP+TGGVKKPHRYRPGTVALREIRRYQKSTE
Sbjct  1    MARTKQTARKSTGGKAPRKQLATKAARKSAPATGGVKKPHRYRPGTVALREIRRYQKSTE  60

Query  164  LLIRKLPFQRLVREIAQDFKTDLRFQSAAIGALQEASEAYLVGLFEDTNLCAIHAKRVTI  223
            LLIRKLPFQRLVREIAQDFKTDLRFQS+A+ ALQEA EAYLVGLFEDTNLCAIHAKRVTI
Sbjct  61   LLIRKLPFQRLVREIAQDFKTDLRFQSSAVMALQEACEAYLVGLFEDTNLCAIHAKRVTI  120

Query  224  MPKDIQLARRIRGER  238
            MPKDIQLARRIRGER
Sbjct  121  MPKDIQLARRIRGER  135
```

>hypothetical protein [Acipenser sinensis]
Sequence ID: MGH0180577.1 Length: 223
Range 1: 18 to 223

Score:381 bits(978), Expect:5e-132,
Method:Compositional matrix adjust.,
Identities:196/222(88%), Positives:200/222(90%), Gaps:16/222(7%)

```
Query  18   RHRKVLRDNIQGITKPAIRRLARRGGVKRISGLIYEETRGVLKVFLENVIRDAVTYTEHA  77
            RHRKVLRDNIQGITKPAIRRLARRGGVKRISGLIYEETRGVLKVFLENVIRDAVTYTEHA
Sbjct  18   RHRKVLRDNIQGITKPAIRRLARRGGVKRISGLIYEETRGVLKVFLENVIRDAVTYTEHA  77

Query  78   KRKTVTAMDVVYALKRQGRTLYGFGGMARTKQTARKSTGGKAPRKQLATKAARKSAPSTG  137
            KRKTVTAMDVVYALKRQGRTLYGFG              G+A    L   AARKSAP+TG
Sbjct  78   KRKTVTAMDVVYALKRQGRTLYGFG------------VRGQA----LRVTAARKSAPATG  121

Query  138  GVKKPHRYRPGTVALREIRRYQKSTELLIRKLPFQRLVREIAQDFKTDLRFQSAAIGALQ  197
            GVKKPHRYRPGTVALREIRRYQKSTELLIRKLPFQRLVREIAQDFKTDLRFQS+A+ ALQ
Sbjct  122  GVKKPHRYRPGTVALREIRRYQKSTELLIRKLPFQRLVREIAQDFKTDLRFQSSAVMALQ  181

Query  198  EASEAYLVGLFEDTNLCAIHAKRVTIMPKDIQLARRIRGERA  239
            EASEAYLVGLFEDTNLCAIHAKRVTIMPKDIQLARRIRGERA
Sbjct  182  EASEAYLVGLFEDTNLCAIHAKRVTIMPKDIQLARRIRGERA  223
```

```
>histone H3.1, partial [Galemys pyrenaicus]
Sequence ID: KAG8511098.1 Length: 640
Range 1: 411 to 640

Score:395 bits(1014), Expect:2e-131,
Method:Compositional matrix adjust.,
Identities:202/230(88%), Positives:207/230(90%), Gaps:8/230(3%)

Query  18   RHRKVLRDNIQGITKPAIRRLARRGGVKRISGLIYEETRGVLKVFLENVIRDAVTYTEHA  77
            RHRKVLRDNIQGITKPAIRRLARRGGVKRISGLIYEETRGVLKVFLENVIRDAVTYTEHA
Sbjct  411  RHRKVLRDNIQGITKPAIRRLARRGGVKRISGLIYEETRGVLKVFLENVIRDAVTYTEHA  470

Query  78   KRKTVTAMDVVYALKRQGRTLYG--------FGGMARTKQTARKSTGGKAPRKQLATKAA  129
            KRKTVTAMD      + G   +         F  MARTKQTARKSTGGKAPRKQLATKAA
Sbjct  471  KRKTVTAMDDARGSIKAGTAAWALIPHRSSLFQAMARTKQTARKSTGGKAPRKQLATKAA  530

Query  130  RKSAPSTGGVKKPHRYRPGTVALREIRRYQKSTELLIRKLPFQRLVREIAQDFKTDLRFQ  189
            RKSAP+TGGVKKPHRYRPGTVALREIRRYQKSTELLIRKLPFQRLVREIAQDFKTDLRFQ
Sbjct  531  RKSAPATGGVKKPHRYRPGTVALREIRRYQKSTELLIRKLPFQRLVREIAQDFKTDLRFQ  590

Query  190  SAAIGALQEASEAYLVGLFEDTNLCAIHAKRVTIMPKDIQLARRIRGERA  239
            S+A+ ALQEA EAYLVGLFEDTNLCAIHAKRVTIMPKDIQLARRIRGERA
Sbjct  591  SSAVMALQEACEAYLVGLFEDTNLCAIHAKRVTIMPKDIQLARRIRGERA  640



Range 2: 246 to 385

Score:267 bits(682), Expect:8e-82,
Method:Compositional matrix adjust.,
Identities:132/140(94%), Positives:135/140(96%), Gaps:0/140(0%)

Query  100  GFGGMARTKQTARKSTGGKAPRKQLATKAARKSAPSTGGVKKPHRYRPGTVALREIRRYQ  159
            G   MARTKQTARKSTGGKAPRKQLATKAARKSAP+TGGVKKPHRYRPGTVALREIRRYQ
Sbjct  246  GSSAMARTKQTARKSTGGKAPRKQLATKAARKSAPATGGVKKPHRYRPGTVALREIRRYQ  305

Query  160  KSTELLIRKLPFQRLVREIAQDFKTDLRFQSAAIGALQEASEAYLVGLFEDTNLCAIHAK  219
            KSTELLIRKLPFQRLVREIAQDFKTDLRFQS+A+ ALQEA EAYLVGLFEDTNLCAIHAK
Sbjct  306  KSTELLIRKLPFQRLVREIAQDFKTDLRFQSSAVMALQEACEAYLVGLFEDTNLCAIHAK  365

Query  220  RVTIMPKDIQLARRIRGERA  239
            RVTIMPKDIQLARRIRGERA
Sbjct  366  RVTIMPKDIQLARRIRGERA  385



>uncharacterized protein LOC106064839 [Biomphalaria glabrata]
Sequence ID: XP_055879400.1 Length: 366
Range 1: 1 to 260

Score:382 bits(980), Expect:4e-130,
Method:Compositional matrix adjust.,
```

```
Identities:216/260(83%), Positives:220/260(84%), Gaps:38/260(14%)

Query  1    MSGRGKGGKGLGKGGAKRHRKVLRDNIQGITKPAIRRLARRGGVKRISGLIYEETRGVLK  60
            MSGRGKGGKGLGKGGAKRHRKVLRDNIQGITKPAIRRLARRGGVKRISGLIYEETRGVLK
Sbjct  1    MSGRGKGGKGLGKGGAKRHRKVLRDNIQGITKPAIRRLARRGGVKRISGLIYEETRGVLK  60

Query  61   VFLENVIRDAVTYTEHAKRKTVTAMDVVYALKRQGRTLYGFGG-----------------  103
            VFLENVIRDAVTYTEHAKRKTVTAMDVVYALKRQGRTLYGFGG
Sbjct  61   VFLENVIRDAVTYTEHAKRKTVTAMDVVYALKRQGRTLYGFGGKGPIGASSFFSVARRAY  120

Query  104  ---------------------MARTKQTARKSTGGKAPRKQLATKAARKSAPSTGGVKKP  142
                                 MARTKQTARKSTGGKAPRKQLATKAARKSAP+TGGVKKP
Sbjct  121  KSLLRARVSSFFLVIDKQVDTMARTKQTARKSTGGKAPRKQLATKAARKSAPATGGVKKP  180

Query  143  HRYRPGTVALREIRRYQKSTELLIRKLPFQRLVREIAQDFKTDLRFQSAAIGALQEASEA  202
            HRYRPGTVALREIRRYQKSTELLIRKLPFQRLVREIAQDFKTDLRFQS+A+ ALQEASEA
Sbjct  181  HRYRPGTVALREIRRYQKSTELLIRKLPFQRLVREIAQDFKTDLRFQSSAVMALQEASEA  240

Query  203  YLVGLFEDTNLCAIHAKRVT  222
            YLVGLFEDTNLCAIHAKR+
Sbjct  241  YLVGLFEDTNLCAIHAKRLN  260
```

Range 2: 264 to 366

Score:206 bits(524), Expect:4e-61,
Method:Compositional matrix adjust.,
Identities:103/103(100%), Positives:103/103(100%), Gaps:0/103(0%)

```
Query  1    MSGRGKGGKGLGKGGAKRHRKVLRDNIQGITKPAIRRLARRGGVKRISGLIYEETRGVLK  60
            MSGRGKGGKGLGKGGAKRHRKVLRDNIQGITKPAIRRLARRGGVKRISGLIYEETRGVLK
Sbjct  264  MSGRGKGGKGLGKGGAKRHRKVLRDNIQGITKPAIRRLARRGGVKRISGLIYEETRGVLK  323

Query  61   VFLENVIRDAVTYTEHAKRKTVTAMDVVYALKRQGRTLYGFGG  103
            VFLENVIRDAVTYTEHAKRKTVTAMDVVYALKRQGRTLYGFGG
Sbjct  324  VFLENVIRDAVTYTEHAKRKTVTAMDVVYALKRQGRTLYGFGG  366
```

>uncharacterized protein LOC112983655 [Dromaius novaehollandiae]
Sequence ID: XP_064367322.1 Length: 329
Range 1: 18 to 222

Score:380 bits(976), Expect:4e-130,
Method:Compositional matrix adjust.,
Identities:192/205(94%), Positives:196/205(95%), Gaps:1/205(0%)

```
Query  18   RHRKVLRDNIQGITKPAIRRLARRGGVKRISGLIYEETRGVLKVFLENVIRDAVTYTEHA  77
            RHRKVLRDNIQGITKPAIRRLARRGGVKRISGLIYEETRGVLKVFLENVIRDAVTYTEHA
Sbjct  18   RHRKVLRDNIQGITKPAIRRLARRGGVKRISGLIYEETRGVLKVFLENVIRDAVTYTEHA  77
```

```
Query  78   KRKTVTAMDVVYALKRQGR-TLYGFGGMARTKQTARKSTGGKAPRKQLATKAARKSAPST  136
            KRKTVTAMDVVYALKRQGR +      MARTKQTARKSTGGKAPRKQLATKAARKSAP+T
Sbjct  78   KRKTVTAMDVVYALKRQGRRSPSALRAMARTKQTARKSTGGKAPRKQLATKAARKSAPAT  137

Query  137  GGVKKPHRYRPGTVALREIRRYQKSTELLIRKLPFQRLVREIAQDFKTDLRFQSAAIGAL  196
            GGVKKPHRYRPGTVALREIRRYQKSTELLIRKLPFQRLVREIAQDFKTDLRFQS+A+ AL
Sbjct  138  GGVKKPHRYRPGTVALREIRRYQKSTELLIRKLPFQRLVREIAQDFKTDLRFQSSAVMAL  197

Query  197  QEASEAYLVGLFEDTNLCAIHAKRV  221
            QEASEAYLVGLFEDTNLCAIHAKR
Sbjct  198  QEASEAYLVGLFEDTNLCAIHAKRC  222
```

Range 2: 227 to 329

Score:172 bits(437), Expect:1e-48,
Method:Compositional matrix adjust.,
Identities:103/103(100%), Positives:103/103(100%), Gaps:0/103(0%)

```
Query  1    MSGRGKGGKGLGKGGAKRHRKVLRDNIQGITKPAIRRLARRGGVKRISGLIYEETRGVLK  60
            MSGRGKGGKGLGKGGAKRHRKVLRDNIQGITKPAIRRLARRGGVKRISGLIYEETRGVLK
Sbjct  227  MSGRGKGGKGLGKGGAKRHRKVLRDNIQGITKPAIRRLARRGGVKRISGLIYEETRGVLK  286

Query  61   VFLENVIRDAVTYTEHAKRKTVTAMDVVYALKRQGRTLYGFGG  103
            VFLENVIRDAVTYTEHAKRKTVTAMDVVYALKRQGRTLYGFGG
Sbjct  287  VFLENVIRDAVTYTEHAKRKTVTAMDVVYALKRQGRTLYGFGG  329
```
